# The “Missing Link”, Allostery and Synergism—Hosting of Metal Cations by Regular and Partial *Cone* Calix[4]arene Isomers

**DOI:** 10.1021/acsorginorgau.5c00095

**Published:** 2025-11-12

**Authors:** Matija Modrušan, Nikola Cindro, Marija Cvetnić, Andrea Usenik, Slavica Petrović, Jakov Borovec, Katarina Leko, Karla Kukina Gradečak, Vladimir Stilinović, Gordan Horvat, Tomica Hrenar, Josip Požar, Vladislav Tomišić

**Affiliations:** Department of Chemistry, Faculty of Science, University of Zagreb, Horvatovac 102a, 10000 Zagreb, Croatia

**Keywords:** calixarenes, atropisomerism, complexation thermodynamics, solvation, solvent inclusion, allosteric effect, cooperativity

## Abstract

The influence of the tetra-*O*-2-oxopropyl-substituted calix­[4]­arene conformation on its binding affinity toward first- and second-group metal cations, as well as on the solvent molecule (acetonitrile or methanol) inclusion in the calixarene hydrophobic cavity, was investigated experimentally and computationally. Misorientation of one monomeric subunit in the partial *cone* ligand (**L**_**p**_) led to incomplete cation desolvation and significantly reduced its cation-binding ability compared to the regular *cone* isomer (**L**_**c**_). Aromatic ring inversion also precluded solvent inclusion in the calixarene *basket* of both free and complexed **L**_**p**_ (in contrast to **L**_**c**_), which considerably affected the complex stabilities and highlighted the pronounced cooperative allosteric effect of this process on the cation binding. Comprehensive structural and energetic studies, carried out by classical molecular dynamics simulations and quantum chemical calculations, showed that inclusion of acetonitrile within the complexes was favored over methanol, whereby the nitrile group of the solvent coordinated the second-group cations. Conversely, the methyl group of included acetonitrile or methanol molecule faced the alkali metal cations in the corresponding adducts. Molecular and crystal structures of free **L**_**p**_, as well as sodium, calcium, and barium complexes of **L**_**c**_ with included acetonitrile, were determined by single-crystal X-ray diffraction. The orientations of solvent molecules within the calixarene cavity in the solid state closely matched computational predictions, further supporting conclusions drawn from the experimental data. Overall, this work presents a particularly detailed account of the thermodynamic and structural aspects of chelate, macrocyclic, and medium effects on the cation-hosting processes, providing valuable insights into the driving forces governing supramolecular recognition in solution.

## Graphical abstract

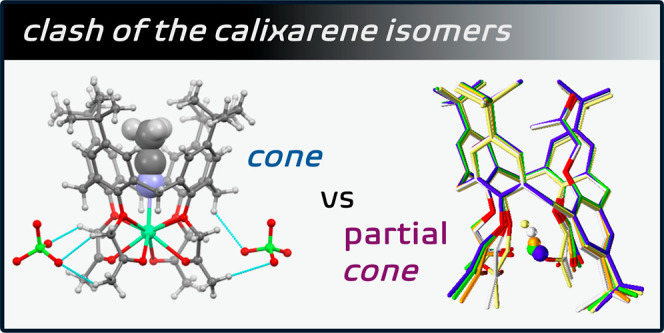

## Introduction

The chelate and macrocyclic effects have been recognized as two main cornerstones of supramolecular chemistry since the pioneering work of Pedersen,^1^ Cramer,^2^ and Lehn.^3^ The subsequent endeavors demonstrated that combination of these effects enabled design and preparation of efficient, selective, and even specific receptors.^4–6^ On the other hand, the early investigations of cyclodextrin hosting properties^2,7,8^ pointed out that solvent reorganization could be an ally, even the primary driving force for the largely unspecific complexation of nonpolar species.^9–11^ Once the calorimetric data on ion hosting became more abundant, the entropically favorable desolvation of high-charge-density guests was identified as a potent driving force on its own.^12–15^ The awareness that efficient recognition can be accomplished through hydrogen bonding^16^ provided further impetus to the development of supramolecular chemistry which in the earlier days almost entirely relied on the affinities established in classical coordination chemistry. Along came the discovery of π–π interactions^17,18^ and halogen bonding.^19^ The so-called superchaotropic effect^20,21^ has been recently added to the list of phenomena on which the efficient hosting in solution can be based.

The established principles of supramolecular recognition and the understanding of underlying binding thermodynamics paved the way for the preparation of next-generation receptors such as cyclophanes,^22^ cucurbiturils,^9,23–25^ and calixarenes,^26–28^ the latter being probably the most widely used macrocyclic scaffolds for preparation of a range of ionic binders^26,27,29,30^ and receptors for neutral species.^31^ In the calixarene derivatives designed and synthesized for these purposes, binding groups are typically introduced at the lower (narrower) and/or upper (wider) rim of the parent compound.^32,33^

Although most investigations involving calixarene derivatives have focused on their synthesis and functionalization for the development of various functional materials (e.g., biomimetics, sensors, extractants), studies devoted to detailed thermodynamic analyses of their binding reactions should be emphasized. By providing a complete thermodynamic characterization of the binding events (i.e., the determination of *K*° and Δ_r_ *H*°, therefore also Δ_r_ *G*° and Δ_r_ *S*°),^14,31,34–43^ a valuable insight into the factors governing the complexation processes can be obtained. Some of these investigations offer a detailed and thermodynamically exact account of the solvent involvement in the binding event.^39,41,44^ This is achieved by determining the standard thermodynamic transfer functions (Δ_t_ *X*°(solvent 1 → solvent 2), *X* = *G*, *H*, *S*) of the reactants and products,^45–48^ which enable the dissection of the differences between the standard reaction quantities among the solvents of interest. Such approach quantifies the solvent preferences toward a range of receptors’ functionalities^35,36,38^ and stresses the importance of competing interactions with the medium.^37,41–44,49,50^

In our previous work,^42^ we have investigated the solvent effect on the extent of cation-binding reactions of a simple 2-oxopropyl-substituted calix[4]­arene (**L**_**c**_, Figure 1) and discussed how the inclusion of a solvent molecule inside the hydrophobic calixarene cavity reflects on its complexation thermodynamics. During repeated synthesis of **L**_**c**_, its partial *cone* atropisomer (**L**_**p**_, Figure 1) was obtained from the reaction mixture, which provided an ideal opportunity to explore the thermodynamic consequences of the “missing link” by carrying out comparative studies of the **L**_**p**_ and **L**_**c**_ hosting abilities.

**Figure 1:**
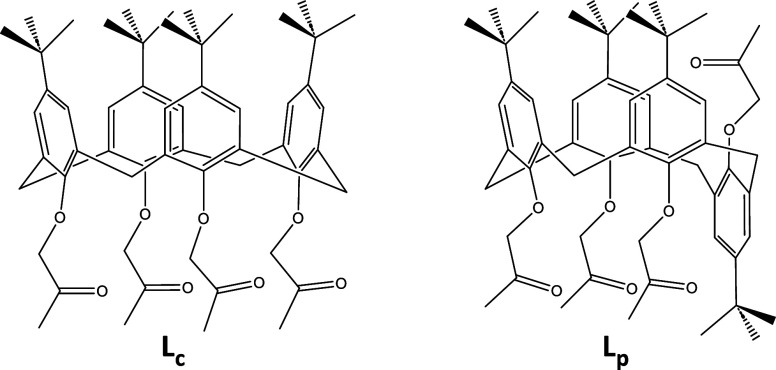
Structures of investigated calix[4]­arenes: *cone* (**L**_**c**_) and partial *cone* derivative (**L**_**p**_).

The related study, involving the complexation of Hg^2+^ with calix[4]­arenes containing variable number of functionalized phenolic groups in acetonitrile, was reported by Danil de Namor et al.^37^ Remarkably, both enthalpic and entropic contributions of calixarene pendant arms to the corresponding reaction quantities were found to be additive. If this holds in general, a similar relation between the standard reaction quantities of cation binding with **L**_**c**_ and **L**_**p**_ could be expected. However, it should be stressed that the investigations presented in ref 37 dealt with ligands in *cone* conformation. Such orientation of subunits enables cation coordination with all phenolic (ether) oxygen atoms and does not interfere with solvent inclusion into the electron-rich calixarene *basket*, which is particularly favorable in the case of acetonitrile.^34,38,39,41,43,51–53^ By contrast, one monomeric subunit of **L**_**p**_ cannot participate in cation coordination and, apart from that, the affinity of free **L**_**p**_ and its complexes for solvent molecules should be reduced compared to its *cone* counterpart.

It is well known that the lower rim calix[4]­arene derivatives with carbonyl-containing groups efficiently bind alkali and alkaline earth metal cations in a range of solvents.^14,35,39,40,48,49,51,54–57^ Considering this fact, and the investigations concerning the pendant functionality contribution to the calixarene coordination reactions,^37^ we opted for comparative thermodynamic, solid-state, and computational studies of the first- and second-group cation complexation reactions with **L**_**p**_ and **L**_**c**_ in acetonitrile and methanol. The solvent choice was based on our earlier investigations of the medium influence on the **L**_**c**_ affinity for alkali metal cations.^42^ Briefly, thermodynamically beneficial inclusion of both solvent molecules (especially MeCN) within the Na**L**_**c**_^+^ cavity was observed, and among the eight examined liquids, acetonitrile was the best medium for cation complexation followed by methanol. The research carried out herein provides a particularly detailed insight into the thermodynamic effects of the “missing link” and calixarene conformation on the cation binding and related solvent molecule inclusion process, thus enabling the evaluation of pendant-arm contribution to the hosting reactions. The findings derived from the thermodynamic experimental studies are supported by crystal structures, as well as by the results of classical molecular dynamics simulations and quantum chemical calculations.

## Results and Discussion

### Synthesis

Synthesis of the tetra-*O*-2-oxopropyl-substituted calix[4]­arene in *cone* conformation is a well-known procedure.^55^ Although **L**_**c**_ is the major product in this reaction and is usually isolated from a crude reaction mixture by crystallization, **L**_**p**_ is obtained to some extent in the reaction as well. By using column chromatography of mother liquor after isolation of **L**_**c**_ and subsequently crystallization of enriched fraction, this “side product” can be isolated as a pure compound (Scheme 1). The mixture of calix[4]­arene tetraketone derivatives in regular and partial *cone* conformation was obtained with yields of 22% and 3%, respectively. The structure of **L**_**c**_ was confirmed by comparing the ^1^H NMR spectrum with the literature one (Figure S1),^55^ whereas **L**_**p**_ was characterized using ^1^H and ^13^C NMR spectroscopy as well as high-resolution mass spectrometry (Section S1.1.1, Figure S2 and S3, Table S1).

**Scheme 1:**
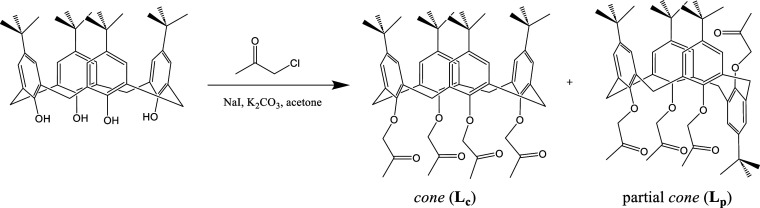
Synthesis of *Cone* (**L**_**c**_) and Partial *Cone* (**L**_**p**_) Calix[4]­arene Ketone Derivatives

### Crystal Structures of Free **L**_**p**_ and **L**_**c**_ Complexes (Solvent Adducts)

Ligand **L**_**p**_ was crystallized from acetonitrile as a solvate. The conformation of **L**_**p**_ in the crystal structure is a partial *cone*, with one of the aromatic rings oriented in the opposite direction than the others (Figure 2). The three rings of the same orientation form a rather regular *cone* with similar dihedral angles between the mean plane of the aromatic rings and the mean planes of the four macrocycle methylene carbon atoms (65.1°, 64.4°, and 68.5°). The inverted aromatic ring is almost parallel to the one opposite to it, with a dihedral angle of ca. 4.1°. The 2-oxopropoxy substituent of the inverted aromatic ring thus protrudes into the (partial) cavity formed by the other three rings. This group is disordered over two orientations, with the majority (81%) of molecules found to have the terminal methyl group positioned in the partial *cone* cavity, apparently forming weak C–H···π hydrogen-bonding contacts with C–H···(ring centroid) distances of ca. 3.62 Å and 3.69 Å, which are comparable to equivalent contact distances in solvates with acetonitrile where the methyl group of the solvent was found to be included in the cavity of a (symmetrical) calix[4]­arene *cone* (the mean distance in 287 crystal structures deposited in the Cambridge Crystallographic Database^58^ which comprise such a contact being 3.635 Å with a standard deviation of 0.162 Å). In the remaining 19% of the molecules, the carbonyl oxygen atom is positioned at the mouth of the cavity and does not appear to participate in any significant intramolecular contacts (the distance toward the *tert*-butyl methyl atoms being in the range 4–4.5 Å, considerably above the usual lengths of C–H···O hydrogen-bonding contacts (generally below 3.2 Å)). Regardless of the orientation of the 2-oxopropoxy substituent of the inverted aromatic ring, its positioning precludes inclusion of the solvent in the calixarene cavity. Instead, the (rather severely disordered) acetonitrile molecules occupy the voids left between the molecules of **L**_**p**_ due to inefficient crystal packing.

**Figure 2:**
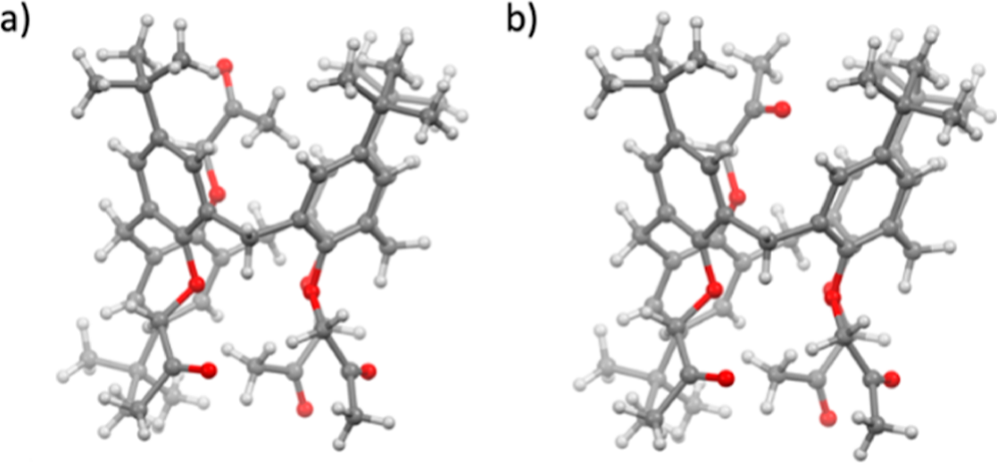
Molecular structure of **L**_**p**_ in the crystal structure of **L**_**p**_·MeCN showing the conformation corresponding to a) the major component (81%) and b) the minor component (19%) of the disordered 2-oxopropoxy substituent.

Crystallization of **L**_**c**_ with Ca­(ClO_4_)_2_ in acetonitrile yielded crystals of an acetonitrile solvate of the calixarene complex, [Ca**L**_**c**_MeCN]­(ClO_4_)_2_·2MeCN (Figure 3a). The cation is positioned between the mean plane of the ether and the mean plane of the carboxyl oxygen atoms of the **L**_**c**_ molecule, being considerably closer to the former (0.814 Å) than to the latter (1.533 Å). Consequently, while all the Ca–O bonds fall in a relatively narrow range (2.43–2.54 Å), the bonds with the ether oxygen atoms are somewhat shorter (2.43–2.47 Å) than those with the carbonyl atoms (2.45–2.54 Å). The eight oxygen atoms of **L**_**c**_ form a square antiprism which is capped by a nitrogen atom from an acetonitrile molecule placed within the calixarene *cone* (with the nitrogen atom 1.77 Å from the mean plane of the ether oxygen atoms), forming a Ca–N bond of 2.581(2) Å. The calixarene *cone* is of approximate *C*_4_ symmetry with only slight flattening, with the dihedral angles of one pair of opposite aromatic rings and the mean plane of the four macrocycle methylene carbon atoms (64.3°, 67.2°) being negligibly smaller than those of the other pair (68.6°, 68.9°). The perchlorate anions bind to the [Ca**L**_**c**_MeCN]^2+^ cations via multiple C–H···O hydrogen-bonding contacts, interconnecting them into a 3D structure. The crystal structure additionally contains noncoordinated acetonitrile molecules which also bind to the perchlorate anions via C–H···O contacts.

**Figure 3:**
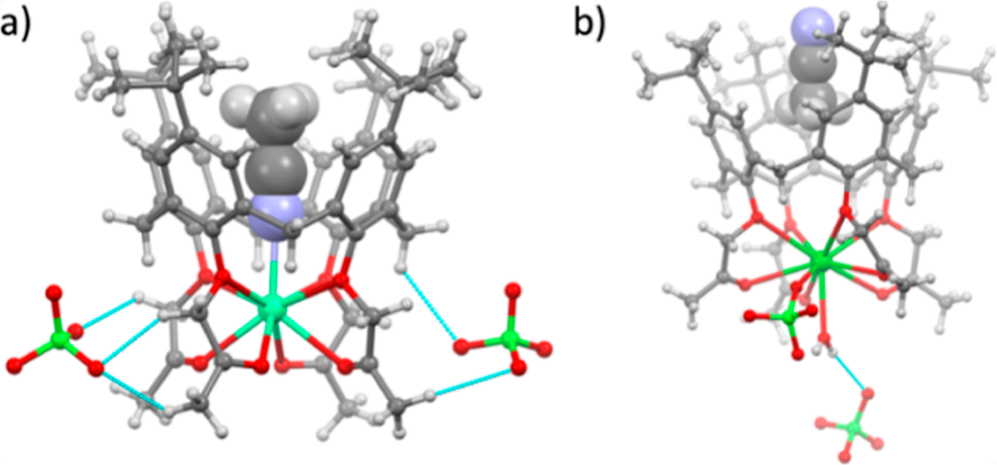
a) Molecular structure of a [Ca**L**_**c**_MeCN]­(ClO_4_)_2_ unit in the crystal structure of [Ca**L**_**c**_MeCN]­(ClO_4_)_2_·2MeCN. b) Molecular structure of one of the symmetrically independent [Ba­(ClO_4_)**L**_**c**_(H_2_O)]­(ClO_4_) units (with the acetonitrile molecule included in the calixarene cavity) in the crystal structure of [Ba­(ClO_4_)**L**_**c**_(H_2_O)]­(ClO_4_)·2.5MeCN·2H_2_O. The MeCN molecules in the calixarene cavities are depicted as a space-filling model (radii shown as 0.5 *r*_vdW_), and the minor components of the disordered (*t*-butyl and perchlorate) groups have been omitted for clarity.

Crystallization of **L**_**c**_ with Ba­(ClO_4_)_2_ in acetonitrile also yielded a crystal of a 1:1 complex of approximate formula [Ba­(ClO_4_)**L**_**c**_(H_2_O)]­(ClO_4_)·2.5MeCN·2H_2_O (unfortunately, due to extreme disorder, the exact content of solvent in the structure could not be unequivocally determined). The asymmetric unit comprises two [Ba­(ClO_4_)**L**_**c**_(H_2_O)]^+^ complex cations (Figure 3b) independent by symmetry. The larger radius of barium as compared to the calcium cation allows for a larger coordination sphere, which is in the case of the barium complex extended by a water molecule and a perchlorate anion, making the overall coordination number 10. The size of the perchlorate also affects the position of the cation within the binding site, placing it closer to the plane of the carbonyl (0.798 Å and 0.955 Å in the two independent molecules) than to the ether oxygen atoms (1.532 Å and 1.536 Å), which reflects also on the bond lengths with the ether oxygen atoms being longer (in the ca. 2.83–2.93 Å range in both complex cations) than the ones with the carbonyl oxygen atoms (in the ca. 2.72–2.83 Å range). This positioning of the barium cation also precludes coordination of the acetonitrile molecule through the calixarene cavity. An acetonitrile molecule is, however, present in the cavities of the *cones* of both complex cations, although here the methyl group penetrates into the *cone*, forming a series of C–H···C­(π) contacts with the phenyl rings of the *cone*. This makes for much more “shallow” penetration, with the bulk of the molecule in the level of the *tert*-butyl groups (the distances of the centroid of the MeCN molecule from the mean plane of the ether oxygen atoms; *d*(centroid_MeCN_–plane_ether‑O_), in the two independent complex cations are 4.576 and 4.614 Å as opposed to 3.834 Å in [Ca**L**_**c**_MeCN]^2+^).

It is interesting to compare the calcium and barium complexes obtained in this study with the previously reported cadmium and lead complexes with the same calixarene ligand.^53^ It is immediately obvious that there are striking similarities on the one hand between the Ca^2+^ and Cd^2+^ and somewhat less between Ba^2+^ and Pb^2+^ complexes, indicating the dominating effect of the radius of the central ion. Ionic radius of Ca^2+^ is only slightly larger (1.00 Å) than that of Cd^2+^ (0.95 Å).^59^ Both Ca^2+^ and Cd^2+^ form M**L**_**c**_MeCN^2+^ complex cations with the acetonitrile molecule within the calixarene *cone* coordinating the metal cation. The Cd–N­(MeCN) bond is shorter (2.428(2) Å) than the Ca–N­(MeCN) bond (see above). This is in accord with a slightly smaller ionic radius allowing it to penetrate more closely to the mean plane of the ether oxygen atoms (by 0.65 Å) than the calcium ion, as well as *softer* character of cadmium which makes the coordination of a *softer* base (nitrogen) more favorable. Position of the MeCN molecule within the *cone* is almost identical in both complexes (nitrogen atom in Cd**L**_**c**_MeCN^2+^ being positioned 1.78 Å, and the centroid of the MeCN molecule 3.856 Å, from the mean plane of the ether oxygen atoms). The only significant difference between the two complexes is the coordination number; unlike in Ca**L**_**c**_MeCN^2+^, in Cd**L**_**c**_MeCN^2+^, only three carbonyl oxygen atoms coordinate the central ion, resulting in the overall coordination number 8. On the other hand, a larger difference between the radii^59^ of Ba^2+^ (1.35 Å) and Pb^2+^ (1.19 Å) cations is reflected with a larger difference between the ensuing complexes with **L**_**c**_: while both cations bind to all eight oxygen atoms of **L**_**c**_, in the case of Pb^2+^, neither counterion nor solvent molecule enters the coordination sphere, making the overall coordination number of Pb^2+^ eight. The lead cation is also more equidistantly placed between the mean planes of the ether oxygen and the carboxyl oxygen atoms (1.219 Å vs 1.459 Å) than the barium cation, although it is still sufficiently removed from the plane of the ether oxygen atoms to allow for inclusion of acetonitrile in the calixarene *cone*—the acetonitrile molecule included in the cavity penetrates with the methyl group and is positioned similarly as in the [Ba­(ClO_4_)**L**_**c**_(H_2_O)]^+^ complex (*d*(centroid_MeCN_–plane_ether‑O_) = 4.688 Å in the lead complex, compared to 4.576 Å and 4.614 Å in the barium complex).

Crystallization of **L**_**c**_ with an excess of NaClO_4_ yielded a quite unexpected product in the solid state. Rather than the expected 1:1 complex obtained with other cations and detected in the solution, the crystallized material was found to be a 1:2 complex comprising asymmetric tetranuclear [Na_4_(ClO_4_)_2_ **L**_**c**2_(H_2_O)_3_]^2+^ cations (overall formula of the obtained crystals being [Na_4_(ClO_4_)_2_ **L**_**c**2_(H_2_O)_3_]­(ClO_4_)_2_·6MeCN; Figure 4). Each of the two calixarene ligands coordinates two sodium cations, one binds in a classical fashion to the calixarene binding site via the four ether and three carbonyl oxygen atoms (a capped-octahedral coordination), while the other is outside of the binding site coordinated only by three carbonyl oxygen atoms. The coordination sphere of the *outer* sodium atoms is a distorted octahedron and is completed by perchlorate anions and water molecules: one sodium binds two water molecules and a perchlorate which bridges to the second *outer* sodium ion which also binds a single water molecule and another perchlorate anion. Two remaining perchlorate anions bind with the [Na_4_(ClO_4_)_2_ **L**_**c**2_(H_2_O)_3_]^2+^ cation via O–H/···O hydrogen bonds with the two water molecules coordinating the former *outer* sodium ion. Out of six acetonitrile molecules, two are contained within the calixarene *cone* cavities with the methyl group penetrating into the *cone*, forming a series of C–H···C­(π) contacts with the phenyl rings of the *cone*. While the tetranuclear complex obtained in the solid state can hardly be taken as a proper representative of the complex present in the solution, the latter aspect (i.e., the inclusion and orientation of the acetonitrile molecule) is probably a good indicator of the state in solution, as the general shape and electrostatics of the calixarene *cone* can be expected to be similar in both cases.

**Figure 4:**
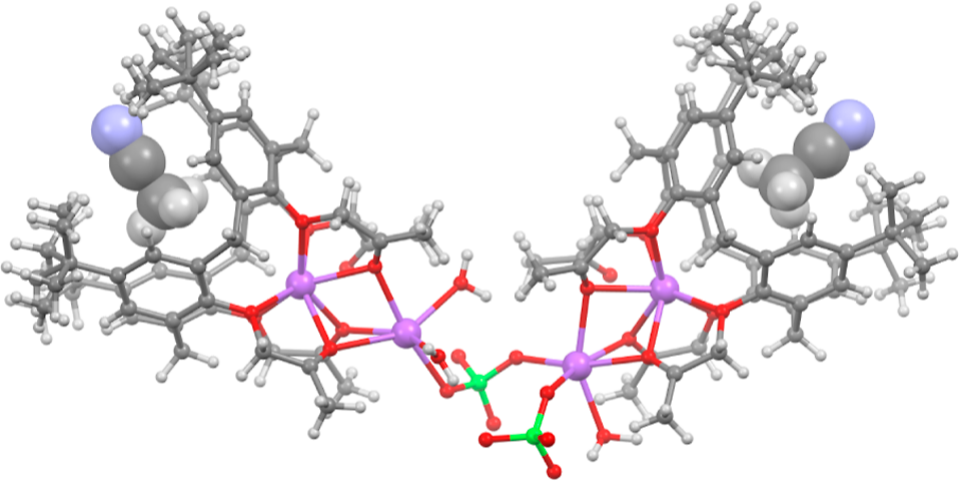
Molecular structure of a [Na_4_(ClO_4_)_2_ **L**_**c**2_(H_2_O)_3_]^2+^ cation with the two MeCN molecules included in calixarene cavities present in the crystal structure of [Na_4_(ClO_4_)_2_ **L**_**c**2_(H_2_O)_3_]­(ClO_4_)_2_·6MeCN.

### Molecular Dynamics Simulations of **L**_**p**_ and **L**_**c**_ in MeCN and MeOH

According to the results of MD simulations, the hydrophobic *basket* of the partial *cone* calixarene derivative **L**_**p**_ is vacant for the most part of the simulation in acetonitrile (the compound forms an adduct with the solvent molecule during only ca. 2% of the simulation time; Table S3 and Figure S13). Two conformations of solvent-free **L**_**p**_ are detected. In the first, the methyl group of the upper rim pendant arm faces the hydrophobic calixarene cavity (77% of the simulation time, Table S9 and Figure S15 (left)), whereas in the second (23%, Table S9 and Figure S15 (right)), this position is occupied by the corresponding carbonyl oxygen atom, leaving the adjacent –CH_3_ group exposed to the solvent. Noteworthily, the distribution of the two orientations closely mirrors that in the crystal structure of the **L**_**p**_ solvate (81:19).

The seldom observed inclusion of the MeCN molecule within **L**_**p**_ resulted in its more opened structure (carbonyl and methyl group of the inverted pendant arm are parallel to the opposite phenyl ring) compared to the solvent-free counterpart resulting in the *cone*-like conformation of the **L**_**p**_MeCN adduct.

The results of **L**_**p**_ simulations in methanol are similar to those in acetonitrile. The solvent-free ligand is again the dominant form with two orientations of the methyl group of the misplaced functionality (Table S4 and Figure S17). The MeOH molecule occupies the calixarene *basket* merely 1% of the total simulation time (Table S4 and Figure S16), whereby the carbonyl oxygen atom of the inverted pendant arm forms a hydrogen bond with the hydroxy group of the included solvent (Figure S17).

The MD investigations of **L**_**c**_ in acetonitrile reveal the expected,^34,38,39,41,43,51–53^ much higher affinity of the calixarene *basket* for MeCN. The acetonitrile molecule occupies the hydrophobic cavity of **L**_**c**_ 62% of the simulation time (Table S3 and Figure S13), whereby the small number of the exchanged solvent molecules additionally indicates the favorability of the realized interactions. As can be seen in Table S3, the mean distances between the *para*-C atoms of the opposite phenyl rings of the ligand are comparable (*C*_4_ conformation of the *basket*) during the period in which the cavity was occupied, whereas in the solvent-free **L**_**c**_, the difference between the corresponding mean values equals ≈3 Å (*C*_2_ conformation; Figure S14). The more rigid conformation of the adduct *basket* is also reflected in the deviations between the methylene bridge torsion angles (Table S3).

Although the inclusion of MeOH molecule into the **L**_**c**_ hydrophobic cavity is observed, the adduct is present only 9% of the simulation time (Table S4, Figures S16 and S17), indicating that the receptor favors acetonitrile over methanol. Namely, the MD simulations suggest that the strongly polar hydroxyl group of methanol prefers to remain exposed to the solvent (i.e., forms hydrogen bonds with the MeOH molecules).

### Complexation Properties of **L**_**p**_ and **L**_**c**_

#### Binding of Alkali Metal Cations

The results of microcalorimetric and spectrophotometric investigations of alkali metal cation complexation by **L**_**p**_ at 25 °C are presented in Section S2.4 of the Supporting Information. The standard thermodynamic complexation reaction parameters (*K*°, Δ_r_ *H*°, and hence Δ_r_ *G*° and Δ_r_ *S*°), obtained by processing the titration curves according to the 1:1 binding model, are listed in Table 1. The corresponding data for the first-group cations hosted by **L**_**c**_ were published in our previous paper.^42^ The thermodynamic reaction parameters for both receptors in acetonitrile are compared in Figure 5. As can be seen, **L**_**c**_ is a superior receptor for smaller alkali metal cations, which is due to the more exothermic complexation reactions with this ligand. The differences in reaction enthalpies and entropies decrease with the cation size for both receptors, resulting in rather similar stability constants for Rb^+^ complexes. Apart from being a better receptor for the alkali metal cations, the *cone* receptor is also the more selective one.

**Table 1:** Thermodynamic Parameters for Complexation of **L**_**p**_ and **L**_**c**_ (Reference 42) with Alkali Metal Cations in MeCN and MeOH at 25 °C^d^

		log *K*°		Δ_r_ *G*°/kJ mol^–1^		Δ_r_ *H*°/kJ mol^–1^		Δ_r_ *S*°/J K^–1^ mol^–1^	
solvent	M^+^	**L**_**p**_	**L**_**c**_	**L**_**p**_	**L**_**c**_	**L**_**p**_	**L**_**c**_	**L**_**p**_	**L**_**c**_
MeCN	Li^+^	5.39(5)^b^	7.19	–30.8(3)^b^	–41.0	–18.2(8)^b^	–37.6	42(3)^b^	11.6
	Na^+^	5.91(2)^b^	9.31	–33.8(1)^b^	–53.1	–33.0(2)^b^	–66.7	2.4(4)^b^	–45.6
	K^+^	4.20(4)^a^	5.02	–24.0(2)^a^	–28.7	–25.7(3)^b^	–43.0	–11(3)^b^	–48.2
		4.10(2)^b^		–23.4(2)^b^					
	Rb^+^	2.64(2)^a^	2.27	–15.1(1)^a^	–12.9	–20.2(3)^b^	–27.8	–19(1)^b^	–53
		2.52(1)^b^		–14.36(2)^b^					
									
MeOH	Na^+^	3.62(3)^a^	5.46	–20.7(2)^a^	–31.16	–16.2(5)^b^	–45.8	13(2)^b^	–48
		3.54(2)^b^		–20.2(1)^b^					
	K^+^	3.27(1)^a^	3.22	–18.66(6)^a^	–18.36	–22.9(3)^b^	–24.3	–16(1)^b^	–18
		3.18(1)^b^		–18.13(2)^b^					
	Rb^+^	2.06(2)^a^		–11.8(1)^a^		–^c^		–^c^	

a Determined spectrophotometrically.

b Determined microcalorimetrically.

c Could not be determined.

d Uncertainties of the last digit are given in parentheses as standard errors of the mean (*N* = 3).

**Figure 5:**
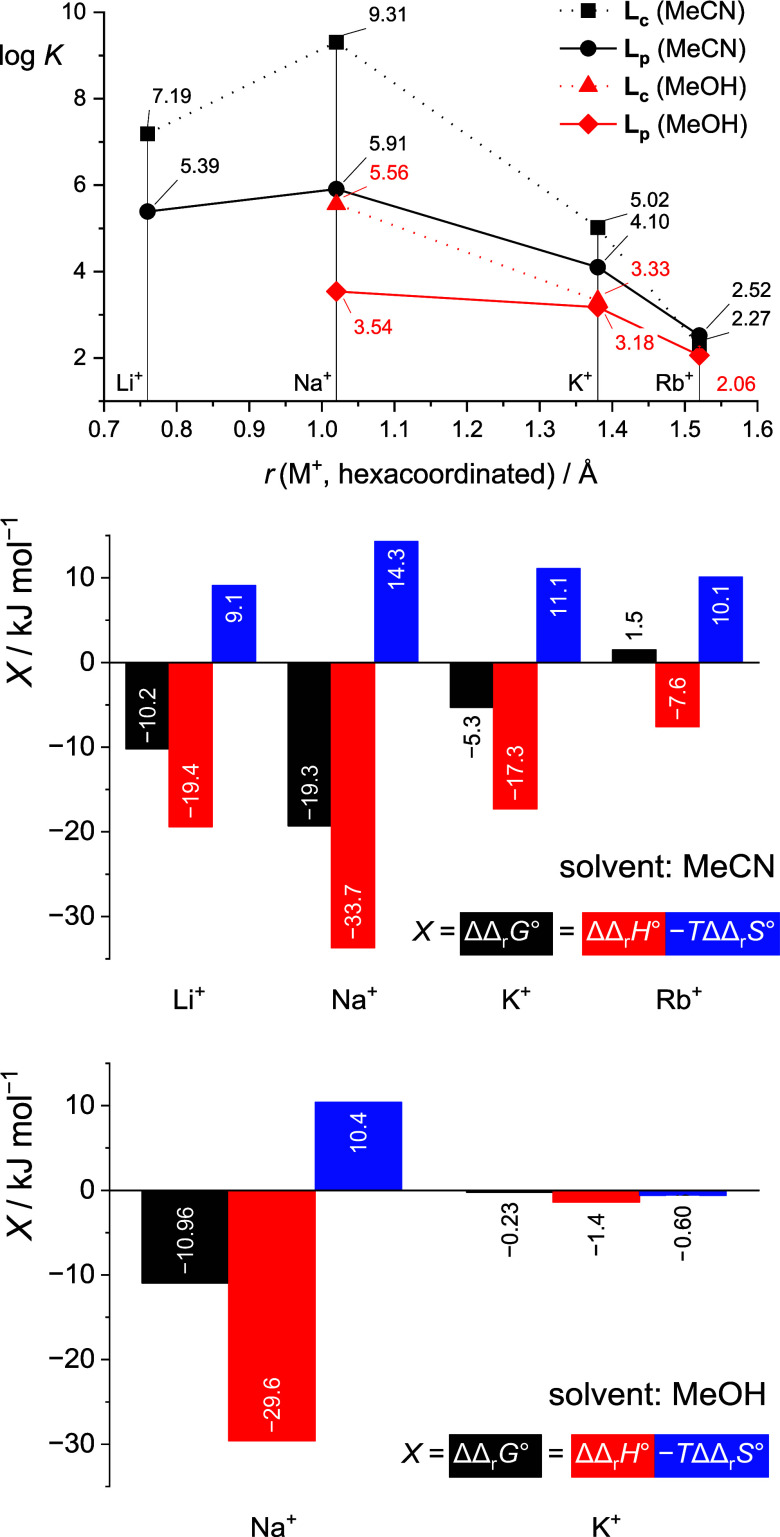
Dependence of stability constants of **L**_**p**_ and **L**_**c**_ complexes with alkali metal cations in acetonitrile and methanol on the cation radius and the difference in thermodynamic parameters of alkali metal cation (M^+^) complexation in acetonitrile and methanol between **L**_**c**_ and **L**_**p**_ defined as ΔΔ_r_ *X*° = Δ_r_ *X*°(M**L**_**c**_^+^) – Δ_r_ *X*°(M**L**_**p**_^+^). Ionic radii from ref 59; values for M**L**_**c**_^+^ from ref 42.

There are several factors which contribute to the systematically lower Δ_r_ *H*° and Δ_r_ *S*° values for the complexation of the *cone* relative to the partial *cone* atropisomer in acetonitrile: the extent of cation desolvation, the enthalpic and entropic contributions of the binding-site organization (i.e., accompanying conformational changes), and the complex and ligand affinities for MeCN molecule inclusion. The latter process is cooperative and synergistic with the metal ion binding, as both promote one another through the appropriate preorganization of the calixarene structure. The results of MD simulations (Section S2.3 in the Supporting Information) indicate that complexation of alkali metal cations by **L**_**p**_ (contrary to alkaline earth metal ones, see later) results in almost complete desolvation, as observed for analogous reactions with **L**_**c**_. Based on this finding alone, one would expect similar complexation entropies for cation binding with both receptors. As for the entropic effect of solvent inclusion within the receptor and corresponding complexes, we have previously established that the free **L**_**c**_ does not bind MeCN in chloroform.^42^ On the other hand, the aromatic *basket* of its sodium complex exhibits a moderate affinity for acetonitrile (log *K* = 1.59).^42^ The solvent inclusion is strongly exothermic (Δ_r_ *H*° = −30 kJ mol^–1^), yet accompanied with quite negative entropy changes (*T*Δ_r_ *S*° = −20.3 kJ mol^–1^). With this in mind, we examined the reactions of **L**_**p**_ and its sodium complex with MeCN in CDCl_3_. No ^1^H NMR spectral changes upon **L**_**p**_ and Na**L**_**p**_^**+**^ titration with acetonitrile in deuterated chloroform could be observed (Figure S82). In line with these results, according to MD simulations, Na^+^, Rb^+^, and Cs^+^ form only solvent-free-type complexes with **L**_**p**_, whereas in the case of Li^+^ and K^+^, the inclusion of an acetonitrile molecule was observed only during a small percentage of the simulation time (Table S7, Figures S26 and S32). Conversely, the alkali metal cations form three types of complexes with **L**_**c**_: one in which the acetonitrile molecule is oriented with the methyl group toward the cation (denoted as M**L**_**c**_MeCN^+^), the other with the nitrile group facing the cation (M**L**_**c**_MeCN′^+^), and the third without the included solvent molecule, M**L**_c_*^+^ (Figure S24). The first type was the most prevalent one in all cases, being present in 87–99% of the simulation time.

Concerning the differences among the **L**_**c**_ and **L**_**p**_ complexation enthalpies (Table 1 and Figure 5), the realized stronger host–guest interactions, combined with concomitant exothermic and allosteric inclusion of the MeCN within the M**L**_**c**_^+^ complexes, favor alkali metal cation coordination with the *cone* atropisomer. In other words, the exploitation of chelate and macrocyclic effects to their full relies on establishing more favorable host–guest and complex–solvent interactions at the expense of reaction entropy.

The decrease of differences in complex stability constants among M**L**_**c**_^+^ and M**L**_**p**_^+^ with the cation radius, as well as the corresponding reaction enthalpies and entropies, could be linked with the fact that the M**L**_**c**_^+^ complex affinities for MeCN decrease with the increase in cation size, while M**L**_**p**_^+^ species do not bind acetonitrile.

With the exception of Li^+^, the stability trends of **L**_**p**_ and **L**_**c**_ complexes observed in acetonitrile also hold in methanol (Table 1 and Figure 5). The extremely low affinity of both receptors for the smallest alkali metal cation is in agreement with numerous previous investigations^14,15,39,41,42,48,57,60^ and can be explained by a particularly favorable lithium cation solvation in MeOH (Δ_t_ *G*°(MeCN → MeOH) = −25 kJ mol^–1^, ref 47). As in acetonitrile, the binding of Na^+^ with the *cone* calixarene results in far lower complexation enthalpy compared to the partial *cone* receptor, whereas the opposite holds for accompanying entropy changes (Figure 5). Furthermore, the differences between Δ_r_ *H*°, and to a lower extent Δ_r_ *S*°, for both receptors again become lower as the cation size increases, leading to similar affinities of both ligands for K^+^.

The results of MD simulations suggest that Na^+^, K^+^, and Rb^+^ complexes with **L**_**p**_ form adducts with the MeOH molecule only during a very short, almost negligible, time (Table S11, Figures S38 and S41). In accordance, the formation of such an adduct with Na**L**_**p**_^+^ in chloroform was not observed experimentally. Apart from that, the carbonyl group of the inverted pendant arm forms hydrogen bonds with methanol molecules solvating the upper macrocyclic rim.

Molecular dynamics analyses further indicate that the M**L**_**c**_MeOH^+^ adducts are the predominant complex species in methanol, although in a smaller proportion than the M**L**_**c**_MeCN^+^ adducts in acetonitrile, and with more than 30 exchanged MeOH molecules during the simulation (Table S10). The formation of such species (specifically Na**L**_**c**_MeOH^+^) was confirmed experimentally in CDCl_3_,^42^ even though the affinity of the complex for this solvent (log *K*(Na**L**_**c**_MeOH^+^) = 0.7) is considerably lower than for acetonitrile (log *K*(Na**L**_**c**_MeCN^+^) = 1.59).

To investigate whether the missing-link influence on the calix[4]­arene complexation properties depends on the cation charge, we decided to include the second-group cations in the study. The corresponding results are presented in the next Section.

#### Binding of Alkaline Earth Metal Cations

UV spectral changes were observed only upon addition of a large excess (up to 1000 equiv) of alkaline earth metal salts to methanol solutions of **L**_**p**_ and **L**_**c**_. However, the complex stabilities were too low for reliable determination of the corresponding equilibrium constants (Figures S70–S77). In contrast, both **L**_**p**_ and **L**_**c**_ exhibit moderate-to-high affinity for the second-group cations in acetonitrile (Figures S57–S69). This difference is most likely because of the much stronger cation solvation in MeOH compared to MeCN (e.g., Δ_t_ *G*°(MeOH → MeCN)/kJ mol^–1^ = 42 (Ca^2+^), 40 (Ba^2+^)).^47^ The complex stability constants in acetonitrile (1:1 stoichiometry) and standard thermodynamic complexation parameters are listed in Table 2 and additionally are presented in Figure 6.

**Table 2:** Thermodynamic Parameters for Complexation of **L**_**p**_ and **L**_**c**_ with Alkaline Earth Metal Cations in MeCN at 25 °C^f^

	log *K*°		Δ_r_ *G*°/kJ mol^–1^		Δ_r_ *H*°/kJ mol^–1^		Δ_r_ *S*°/J K^–1^ mol^–1^	
M^2+^	**L**_**p**_	**L**_**c**_	**L**_**p**_	**L**_**c**_	**L**_**p**_	**L**_**c**_	**L**_**p**_	**L**_**c**_
Mg^2+^	2.64(4)^a^	3.35(5)^a^	–15.1(3)^a^	–19.1(5)^a^	–^e^	–^e^	–^e^	–^e^
Ca^2+^	3.17(4)^a^	12.16^b^	–18.2(2)^a^	–69.41^b^	–^e^	–49.0(3)^d^	–^e^	14^b^
						–65.3^b^		
Sr^2+^	2.73(3)^a^	9.22(3)^a^ ^,^ ^c^	–15.6(2)^a^	–52.6(3)^c^	–^e^	–31.9(2)^d^	–^e^	69(1)^d^
Ba^2+^	3.74(3)^a^	6.11(4)^d^	–21.3(4)^a^	–34.87(5)^d^	–9.5(4)^d^	–30.6(5)^d^	38(2)^d^	14(2)^d^
	3.65(1)^d^		–20.81(7)^d^					

a Determined spectrophotometrically.

b From ref 53.

c Determined by competitive titrations.

d Determined microcalorimetrically.

e Could not be determined.

f Uncertainties of the last digit are given in parentheses as standard errors of the mean (*N* = 3).

**Figure 6:**
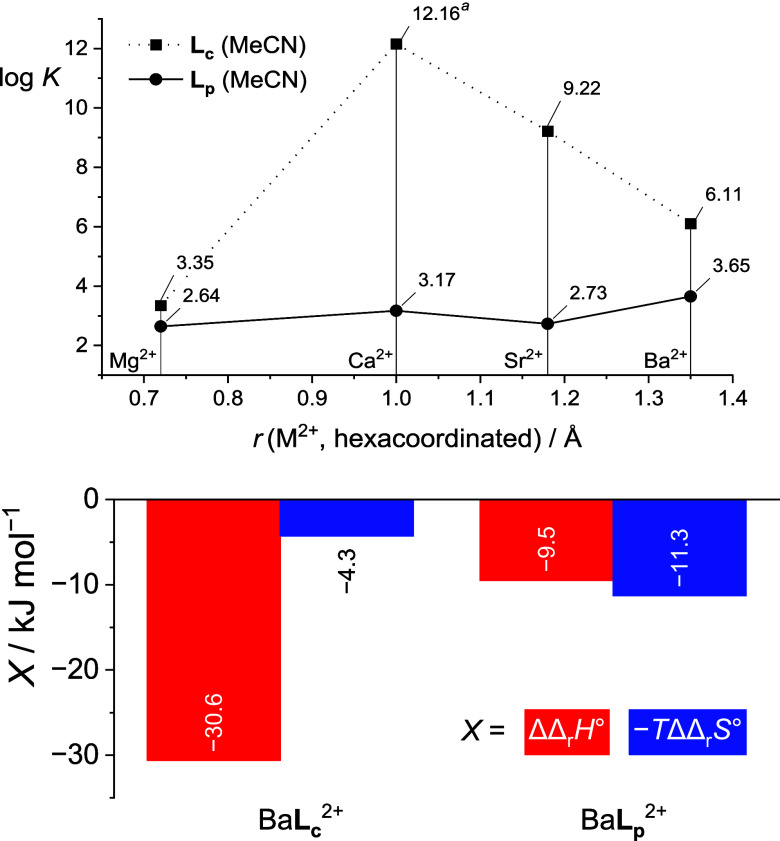
Dependence of stability constants of **L**_**p**_ and **L**_**c**_ complexes with alkaline earth metal cations in acetonitrile on the cation radius and the difference in the enthalpic and entropic contributions to Δ_r_ *G*°(Ba**L** ^2+^; **L** = **L**_**c**_, **L**_**p**_) defined as ΔΔ_r_ *X*° = Δ_r_ *X*°(M**L**_**c**_^+^) – Δ_r_ *X*°(M**L**_**p**_^+^). ^a^From ref 53. Ionic radii from ref 59.

The partial *cone* calix[4]­arene is clearly an inferior host compared to **L**_**c**_ for the second-group cations. The results of MD simulations indicate that Mg^2+^, and to a certain extent Sr^2+^, are coordinated by three carbonyl oxygen atoms of **L**_**p**_, while Ca^2+^ and Ba^2+^ are bound by two of them (Table S8 and Figure S33). The cations remain partially solvated with MeCN molecules and are positioned outside the commonly expected binding site. The effect of incomplete desolvation on the cation coordination is clearly seen by a comparison of the complex MD structures obtained in acetonitrile and in vacuo (Figures 7, S33, and S93). Although the inclusion of the MeCN molecule within the hydrophobic M**L**_**p**_^2+^ cavity was observed in all cases, the complexes were predominantly solvent-free (4% of the simulation time was the longest period in which the MeCN occupied the cavity of Ca**L**_**p**_^2+^, Table S8 and Figure S33). By contrast, simulations showed that the alkaline earth metal cation complexes with **L**_**c**_ formed adducts with acetonitrile molecules, which were the predominant species (present during nearly the whole of the simulation time for all cations). Both the coordination of Mg^2+^ by the nitrile group of the solvent (1% of the simulation time) and its inclusion with the methyl group facing the cation (98%) were observed, whereas only the former type of adduct was detected for the other divalent cations (Figure S25). The formation of such species (specifically Ca**L**_**c**_MeCN′^2+^) was also observed in the solid state and by DFT calculations (see later). Intriguingly, the smallest Mg^2+^ was coordinated by four carbonyl oxygen atoms and positioned at the “edge” of the lower-rim substituents, which prevented its interaction with the included solvent. On the other hand, the placement of calcium, strontium, and barium cations closer to the plane defined by ether oxygen atoms resulted in much higher coordination numbers, approaching nine (including coordination by included MeCN) in the case of largest Ba^2+^. All cations interacted with, on average, one additional acetonitrile molecule at the periphery of the pendant arms (Table S6).

**Figure 7:**
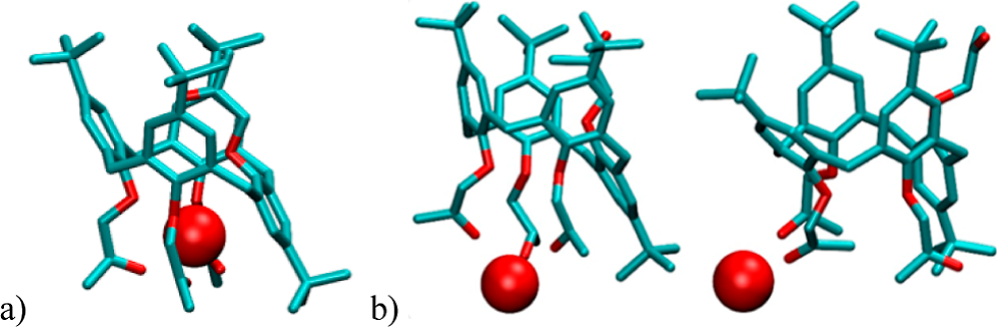
Representative structures of Sr**L**_p_*^2+^ obtained by MD simulations: a) in vacuo and b) in MeCN at 25 °C. Hydrogen atoms of receptors and cation-solvating MeCN molecules are omitted for clarity.

In agreement with the MD results, the alkaline earth metal cation complexes with **L**_**p**_ exhibited a much lower affinity for the inclusion of MeCN in chloroform compared to M**L**_**c**_^2+^. The equilibrium constant for the formation of the Ca**L**_**p**_^2+^ adduct with MeCN could not be determined experimentally, while the log *K*(Ca**L**_**c**_MeCN′^2+^) obtained by means of ^1^H NMR spectroscopy (Figure S85) and ITC (Figure S86) equaled 2.59 and 2.80, respectively. We can therefore conclude that the notably higher affinity of **L**_**c**_ for the second-group cations compared to the first-group ones stems from the more favorable interactions between ether and carbonyl oxygen atoms and doubly charged cations, as well as the higher affinity of its complexes for the inclusion of the MeCN molecule, which also participates in cation coordination. This more than compensates for the predictably entropically unfavorable conformational changes associated with the cation binding and those related to the solvent inclusion.

The data listed in Table 2 further reveal that the more symmetrical **L**_**c**_ prefers Ca^2+^ over other alkaline earth metal cations for enthalpic reasons. This cation has a similar ionic radius to Na^+^ (*r*(Na^+^) = 102 pm, *r*(Ca^2+^) = 100 pm) for coordination number 6 (ref 59) and is hence most compatible with the binding-site size of calix[4]­arenes in *cone* conformation. Interestingly, **L**_**p**_ most strongly binds much larger Ba^2+^, although it should be stressed that the affinities of this ligand for all alkaline earth metal cations are moderate and comparable.

The dependence of *cone* calixarene complex stability constants on the cation radius is qualitatively similar as in the case of alkali metal ions (Figures 5 and 6). By comparing the Δ_r_ *H*° values for the binding of alkali and alkaline earth metal cations of similar radii (Tables 1 and 2), it can be noticed that the binding of the former is more exothermic. Consequently, the **L**_**c**_ preference for the alkaline earth metal cations additionally arises from particularly entropically beneficial cation complexation. Such notable influence of the cation charge density on the complexation thermodynamics can be explained by large differences in the corresponding standard solvation enthalpies and entropies. Specifically, the particularly demanding removal of MeCN molecules from the solvation spheres of second-group cations (e.g., Δ_sol_ *H*°(Na^+^, MeCN) = −429 kJ mol^–1^ Δ_sol_ *H*°(Ca^2+^, MeCN) = −1583 kJ mol^–1^; refs 47 and 59 ) leads to their less exothermic coordination. On the other hand, the desolvation of alkaline earth metal cations is a strongly entropically favored process (Δ_sol_ *S*°(Na^+^, MeCN) = −232 J K^–1^ mol^–1^, Δ_sol_ *S*°(Ca^2+^, MeCN) = −491 J K^–1^ mol^–1^, ref 47), resulting in higher complexation entropies.

Another important contribution to the differences in **L**_**c**_ affinity for the first- and second-group cations concerns the complex tendency to bind MeCN molecules. As previously noted, the stabilities of M**L**_**c**_^2+^ and M**L**_**c**_^+^ adducts with acetonitrile in chloroform differ by an order of magnitude. The thermodynamic parameters for MeCN inclusion inside the cavity of Ca**L**_**c**_^2+^ are presented in Figure 8. The formation of the Ca**L**_**c**_MeCN^2+^ adduct is considerably more exothermic than that of Na**L**_**c**_MeCN^+^. This is in line with Ca^2+^ coordination by the solvent nitrile group, in contrast to its inclusion with the methyl group facing the sodium cation in the latter species, as observed in the solid state (Figures 3a and 4), during MD simulations (Tables S5 and S6, Figures S24 and S25), and by DFT calculations (see the next Section). The MeCN inclusion is significantly less pronounced in the case of Li**L**_**c**_^+^ and even more so for Ba**L**_**c**_^2+^ (log *K* = 1.17 and 0.63, respectively). Although slight ^1^H NMR spectral changes were observed, Ca**L**_**c**_^2+^ and Ba**L**_**c**_^2+^ formed very weak adducts with MeOH in chloroform, so their stability constants could not be determined (log *K* <0.5).

**Figure 8:**
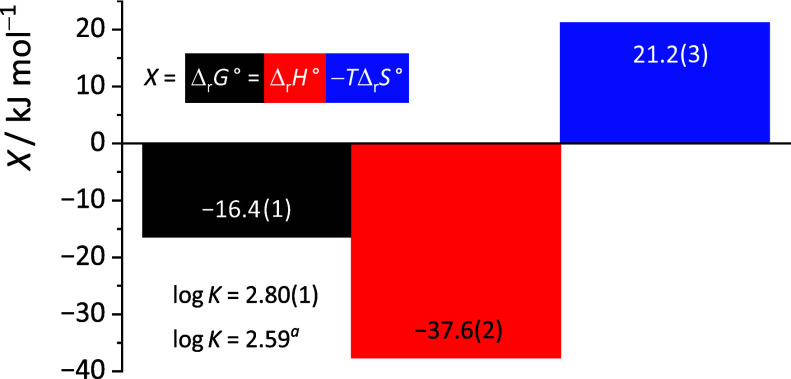
Thermodynamic parameters of MeCN inclusion into the hydrophobic cavity of Ca**L**_**c**_^2+^ at 25 °C determined calorimetrically. Uncertainties of the last digit are given in parentheses as standard errors of the mean (*N* = 3). ^a^Determined by means of ^1^H NMR.

#### Quantum Chemical Studies

The optimized structures of **L**_**c**_ and **L**_**p**_ conformers obtained by DFT calculations are shown in Figures 9 and S93. In line with the experimental and MD findings, the results of these calculations indicate that the inclusion of the MeCN or MeOH molecule in the hydrophobic cavity of **L**_**c**_, with the solvent methyl group pointing toward the lower rim, is a thermodynamically favorable process, more so in the case of MeCN (Tables S17 and S19). On the other hand, the Gibbs energies for the processes of solvent inclusion in the partial *cone* of **L**_**p**_ were found to be positive or close to zero. This is in accordance with the experimental thermodynamic studies and is largely a consequence of the orientation of the 2-oxopropoxy group of the inverted aromatic ring (Figure 9), which prevents inclusion of the solvent molecule, as also observed in the crystal and MD-obtained structure of **L**_**p**_.

**Figure 9:**
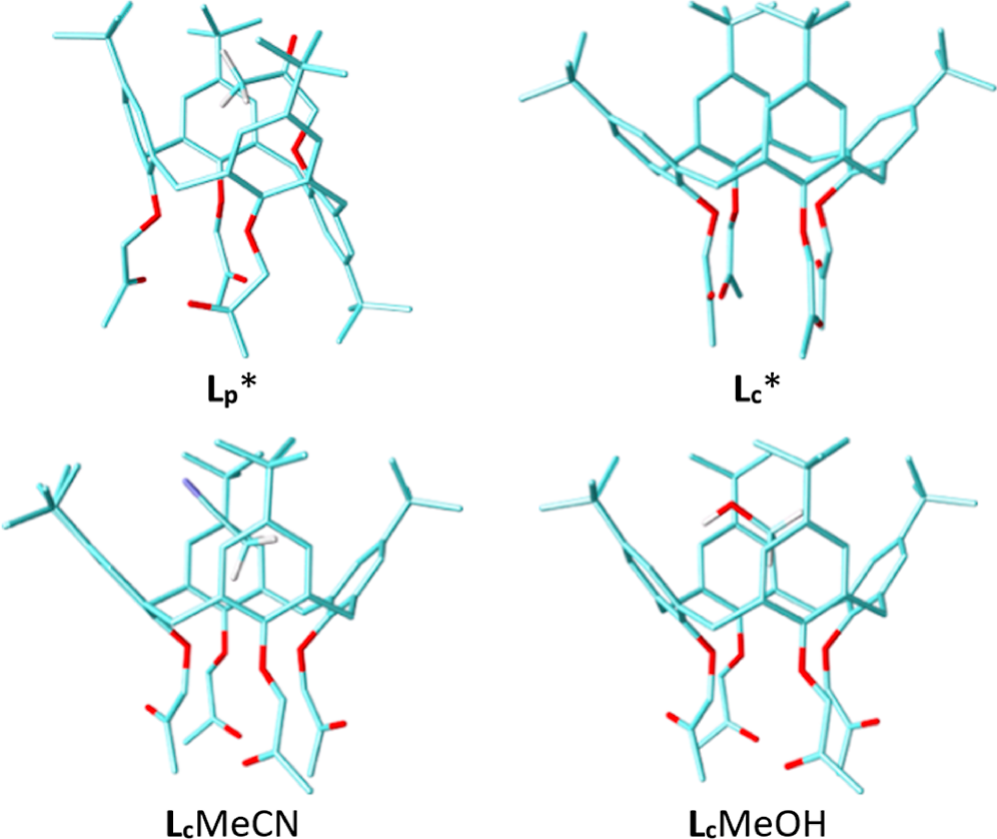
Optimized geometries of **L**_**p**_, **L**_**c**_, and adducts of **L**_**c**_ with MeCN and MeOH calculated by the B3LYP-D3BJ/def2-SVP method.

To further elucidate the structural and energetic features of **L**_**c**_ complexes with alkali and alkaline earth metal cations, a detailed quantum chemical analysis was carried out. In this respect, particular attention was paid to the orientation of the included solvent molecule and its interaction with the complexed cation. Overlays of the optimized structures of the **L**_**c**_ complex species with and without MeCN or MeOH inside the calixarene *basket* are shown in Figures 10 and S92, and the corresponding characteristic distances defined by the cation and solvent positions are given in Tables S24–S26.

**Figure 10:**
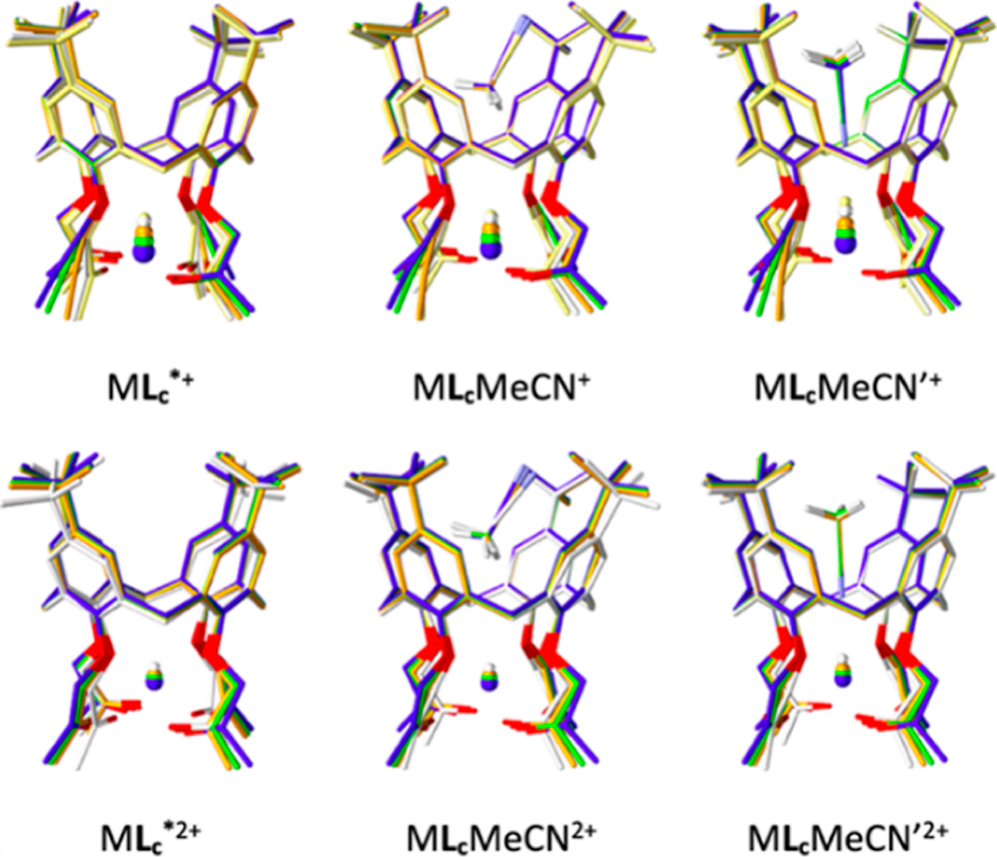
Optimized geometries of **L**_**c**_ complexes with alkali and alkaline earth cations and the corresponding adducts with MeCN calculated by the B3LYP-D3BJ/def2-SVP method. Li^+^ (orange), Na^+^ and Mg^2+^ (white), K^+^ and Ca^2+^ (orange), Rb^+^ and Sr^2+^ (green), Cs^+^ and Ba^2+^ (blue).

As expected,^52,61^ in all cases, the smaller cations are positioned closer to the least-squares-fit plane through the ether oxygen atoms, whereby the inclusion of the acetonitrile or methanol molecule with the methyl group pointing toward the metal ion in M**L**_**c**_S^+^ and M**L**_**c**_S^2+^ species (M denotes the first- or second-group cation and S stands for solvent) does not significantly alter these positions (Figure 10 and Table S24). However, when the solvent molecule is oriented with the –CN or –OH group facing the cation in M**L**_**c**_S′^+^ and M**L**_**c**_S′^2+^ adducts (Figures 10 and S92), metal ions are shifted in the direction of the plane by 0.1–0.5 Å (Table S24), due to the stronger cation–solvent interaction. That leads to the cation coordination by the included solvent (particularly in the case of divalent cations) and is also evidenced by the position of the MeCN or MeOH molecule located much closer to the ether–oxygen plane (for more than 1.5 Å; Table S24). The alkaline earth metal cations are somewhat farther from the ether oxygen atoms and closer to carbonyl ones than the alkali metal ions of comparable sizes (Mg^2+^ vs Li^+^, Ca^2+^ vs Na^+^, Ba^2+^ vs K^+^, Tables S25 and S26). However, in all cases, cations are placed slightly closer to the ether than carbonyl oxygens, as observed in the crystal structure of the Ca**L**_**c**_MeCN′^2+^ complex described above. The calculated Ca^2+^-(coordinating N) distance (2.52 Å) is in excellent agreement with that in the solid state (2.58 Å).

Considering the energetics of the systems studied, the computational results regarding the orientation of the solvent molecule included in the calixarene *basket* of M**L**_**c**_^+^ and M**L**_**c**_^2+^ complexes are completely in accordance with the experimental findings. From the calculated binding Gibbs energies (Tables S17, S19, S21, and S23) for the reactions

1 $$M{L_{c}}^{z}+S⇌ML_{c}S^{z}$$

2 $$M{L_{c}}^{z}+S⇌ML_{c}S\prime^{z}$$

the corresponding values for the process

3 $$ML_{c}S^{z}⇌ML_{c}S\prime^{z}$$

can be obtained (*z* denotes charge number). As seen from the data listed in Table 3, the reorientation of the MeCN or MeOH molecule from the position in which the methyl group faces the cation to the opposite one, in which the cation is coordinated by the –CN or –OH group, is slightly favorable for complexes of smaller alkali metal cations. Conversely, for all alkaline earth metal ions, the corresponding changes in Gibbs energies are much lower (by more than 50 kJ mol^–1^ for cations of comparable radii in the case of MeCN, and more than 20 kJ mol^–1^ for MeOH), indicating substantially higher preference of the second-group cations for coordination by the included solvent molecule. In almost all cases, the differences in Δ_r_ *G* values for processes (3) are mostly determined by the favorable enthalpy changes resulting from cation coordination by the –CN/–OH group (Table 3), since the orientation of the included solvent molecule does not significantly alter the adduct entropy.

**Table 3:** Standard Gibbs Free Energies, Enthalpies, and Entropies of Solvent Reorientation in the M**L**_**c**_^*z*^ Complexes (M**L**_**c**_S^*z*^ ⇌ M**L**_**c**_S′^*z*^; S = MeCN or MeOH, *z* Denotes the Charge Number) Calculated at the B3LYP-D3BJ/def2-SVP Level of the Theory

	Δ_r_ *G*°/kJ mol^–1^		Δ_r_ *H*°/kJ mol^–1^		Δ_r_ *S*°/J K^–1^ mol^–1^	
M^*z*^	MeCN	MeOH	MeCN	MeOH	MeCN	MeOH
Li^+^	–7.14	–1.93	–7.32	–2.21	–0.61	–0.96
Na^+^	–5.42	–5.15	–5.76	–4.24	–1.15	3.04
K^+^	1.43	–6.23	2.14	–1.85	2.39	14.71
Rb^+^	5.90	–7.20	5.96	0.69	0.19	26.49
						
Cs^+^	9.98	0.80	11.19	–2.21	4.07	5.46
Mg^2+^	–73.20	–40.81	–80.82	–46.58	–25.56	–19.34
Ca^2+^	–58.55	–30.30	–62.85	–36.31	–14.42	–20.18
Sr^2+^	–57.14	–30.64	–59.75	–36.85	–8.74	–20.82
Ba^2+^	–48.42	–28.51	–52.57	–33.05	–13.93	–15.23

The inclusion of both MeCN and MeOH within the **L**_**c**_ *basket* is energetically far more favorable for the complex species (reactions (1) and (2)) than for the free ligand, as indicated by the considerably lower corresponding Δ_r_ *H* and Δ_r_ *G* values (Tables S17, S19, S21, and S23), and occurs to a much larger extent in the case of divalent cation complexes. Moreover, the M**L**_**c**_MeCN′^2+^ adducts are thermodynamically more stable than M**L**_**c**_MeOH′^2+^ species (Tables S21 and S23; e.g., Δ_r_ *G*(Ca**L**_**c**_MeCN′^2+^) = −72 kJ mol^–1^, Δ_r_ *G*(Ca**L**_**c**_MeOH′^2+^) = −54 kJ mol^–1^). These findings support conclusions drawn from the MD simulations and experimental results, further underscoring the synergistic interplay between solvent inclusion and cation binding.

#### Thermodynamic Functions for Transfer of Ligands and Complexes from MeOH to MeCN

In our previous investigations of the alkali metal cation complexation with **L**_**c**_,^42^ the solvent effect on complexation thermodynamics was thoroughly discussed through standard thermodynamic functions of the ligand, cation, and complex transfer from methanol to acetonitrile (Δ_t_ *X*°(MeOH → MeCN), *X* = *G*, *H*, *S*). These values were obtained by determining the receptor solubilities (hence standard solution Gibbs energies) and standard solution enthalpies and were combined with the literature data for the free-cation transfer^47^ to calculate the transfer Gibbs energies, enthalpies, and entropies of the complexes. The relation between Δ_r_ *X*° and Δ_t_ *X*° for the two solvents is given by the following equation

$$\begin{array}{l} \Delta_{r}X^{{}^{\circ}}\left(MeCN\right)-\Delta_{r}X^{{}^{\circ}}\left(MeOH\right)=\Delta_{t}X^{{}^{\circ}}\left(ML^{+},\;MeOH\to MeCN\right)\\ -\Delta_{t}X^{{}^{\circ}}\left(M^{+},\;MeOH\to MeCN\right)\\ -\Delta_{t}X^{{}^{\circ}}\left(L,\;MeOH\to MeCN\right) \end{array}$$ 4

The analysis of the obtained data revealed that MeCN was a far better complexation medium due to particularly exergonic transfer of complex species from methanol to this solvent,^42^ and in the case of Li^+^ and Na^+^, the stronger free-cation solvation in methanol.^47^ The thermodynamically beneficial product solvation in acetonitrile (e.g., Δ_t_ *G*°(K**L**_**c**_^+^, MeOH → MeCN) = −16.2 kJ mol^–1^) was rationalized by the higher affinity of **L**_**c**_ complexes for MeCN inclusion compared to the free receptor.^42^ The analogous dissection of thermodynamic data was carried out in the present work for the reactions of alkali metal cations with the partial *cone* derivative **L**_**p**_. Its solubilities and solution enthalpies are given in Table S13, whereas standard thermodynamic functions of its transfer from methanol to acetonitrile are listed in Table 4. As can be seen, the standard Gibbs energy of **L**_**p**_ transfer among the two solvents is relatively low. The differences in the cation binding affinities in explored media are hence mostly determined by the free cation and complex transfer Gibbs energies (Table 4). The complete thermodynamic analysis of the solvent influence on the complexation of the most strongly bound Na^+^ is presented in Schemes 2, S1, and S2, and in Schemes S3–S6 for the other cations. The larger complex stability in acetonitrile is due to the endergonic Na^+^ and exergonic Na**L**_**p**_^+^ transfer from MeOH to MeCN. Given the fact that the sign of the Δ_t_ *G*°(cation) changes with increasing radius (Table 4), the complexation Gibbs energies in methanol and acetonitrile corresponding to larger cations become similar. This in turn results in comparable **L**_**p**_ binding affinities for Rb^+^ in both solvents (Table 1 and Figure 5). The **L**_**p**_ transfer Gibbs energy is larger than that of its *cone* counterpart (Δ_t_ *G*°(MeOH → MeCN)/kJ mol^–1^ = −1.9 (**L**_**p**_); −4.5 (**L**_**c**_)^42^). The reduced solvent influence on the stability of alkali metal cation complexes with the partial *cone* derivative is therefore almost completely a consequence of the less exergonic transfers of the M**L**_**p**_^**+**^ species from methanol to acetonitrile. For instance, the value of Δ_t_ *G*°(MeOH → MeCN) amounts to −8.5 kJ mol^–1^ for Na**L**_**p**_^+^ and that of Na**L**_**c**_^+^ to as high as −18.9 kJ mol^–1^ (ref 42). The experimentally and computationally observed more pronounced inclusion of the acetonitrile compared to methanol into the cavity of alkali metal cation complexes with the *cone* receptor vs weak affinity of their partial *cone* analogues for solvent molecules (Sections S2.3, S2.4, S2.8, and S2.10 in the Supporting Information) provides rationale for this finding.

**Table 4:** Thermodynamic Functions of Transfer from Methanol to Acetonitrile for Alkali and Alkaline Earth Metal Cations, **L**_**p**_, and Their Complexes^a^

	Δ_t_ *G*°/kJ mol^–1^	Δ_t_ *H*°/kJ mol^–1^	Δ_t_ *S*°/J K^–1^ mol^–1^
Li^+^	21	13.7	–24
Na^+^	7	7.4	1
K^+^	–2	–3.9	–6
Rb^+^	–4	–8.1	–14
Cs^+^	–3	–11.9	–30
			
Ca^2+^	42	71.6	101
Ba^2+^	40	53.0	50
			
**L**_**p**_	–1.9	–9.2	–24.8
Na**L**_**p**_^+^	–8.5	–18.6	–34
K**L**_**p**_^+^	–9.2	–15.9	–26
Rb**L**_**p**_^+^	–8.5	–^b^	–^b^

a Values for M^+^ and M^2^^+^ from ref 47.

b Not determined.

**Scheme 2:**
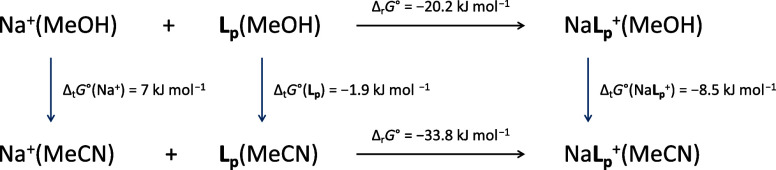
Thermodynamic Cycle Explaining the Difference in Δ_r_ *G*° for Complexation of Na^+^ with **L**_**p**_ in MeOH and MeCN

## Conclusions

Comparative thermodynamic and computational investigations of **L**_**p**_ and **L**_**c**_ affinities for first- and second-group cations provided detailed insights into the enthalpic and entropic effects of the inverted subunit in **L**_**p**_ on the cation coordination, tendency of free and complexed ligands to form solvent adducts, and the allosteric impact of this process, as well as the conformational changes accompanying the cation-binding reactions.

The significantly lower stabilities of the partial *cone* atropisomer complexes were shown to stem from the markedly less exothermic cation coordination and the absence of enthalpically favorable solvent inclusion within the calixarene *basket*. In contrast, the complexation with the *cone* counterpart resulted in notably lower Δ_r_ *S*° values, reflecting a larger entropic loss upon cation binding due to the conformational rigidification and the pronounced preference of **L**_**c**_ complexes for solvent inclusion. The stability of the acetonitrile adduct of Ca**L**_**c**_^2+^ was considerably higher than that of the Na**L**_**c**_^+^ analogue, predominantly due to the more exothermic formation of the former. This was consistent with previous studies,^51,52^ which proposed that in the complexes of another calix[4]­arene derivative, the alkaline earth metal cations were coordinated by the MeCN nitrile group, while the methyl group of the included solvent molecule faced the alkali metal ions. Indeed, such orientations of acetonitrile molecules within the **L**_**c**_ cavity were observed here in the crystal structures of its calcium and sodium complexes. Moreover, the existence of such adducts in solution and gas phase was corroborated by molecular dynamics simulations and quantum chemical calculations. The computational results also provided deeper insights into the structural and energetic aspects behind the complexation thermodynamics of all studied systems.

Apart from being the more efficient receptor, the *cone* isomer also exhibited greater selectivity, which was largely attributed to the increased flexibility of the partial *cone* derivative, i.e., its size-adaptable binding site, resulting in only partial cation desolvation, particularly in the case of alkaline earth metal cations. The *cone* receptor demonstrated markedly higher affinity for divalent cations over monovalent ones of comparable radii (with the exception of Mg^2+^ vs Li^+^), while **L**_**p**_ preferred the less solvated alkali metal cations. Both receptors showed a peak affinity for Na^+^ among the first-group cations in both acetonitrile and methanol. However, **L**_**c**_ formed the most stable complex with Ca^2+^, while **L**_**p**_ favored Ba^2+^ in acetonitrile. Complexation of alkaline earth metal cations in methanol was barely detectable due to their particularly strong solvation in this solvent.

Acetonitrile proved to be a better reaction medium in all cases. The thermodynamic transfer functions of monovalent cations, ligands, and their complexes between methanol and acetonitrile indicated that the greater solvent impact on **L**_**c**_ complexation arose from the more pronounced tendency of its complexes to form MeCN adducts. Conversely, no solvent inclusion within the **L**_**p**_ complexes could be observed regardless of the cation involved.

The pronounced cooperative effect of specific calixarene–solvent interactions can be regarded as both allosteric and synergistic since cation binding and solvent inclusion mutually reinforce each other through the preorganization of the calixarene structure, resulting in enhanced stabilization of the ternary complex formed.

The influence of the inverted pendant arm in **L**_**p**_ on the cation binding differs considerably from the additive pendant arm contributions previously reported for Hg^2+^ complexation with *cone* calix[4]­arenes in acetonitrile.^37^ The absence of cation coordination by the phenolic oxygen atom of the inverted arm coupled by the related inability of **L**_**p**_ complexes to form solvent adducts gives rise to a more intricate binding behavior than would be expected by considering only a simple additive model. The range of phenomena reported herein, which define the receptor properties of the studied macrocycles, underscores the importance of comprehensive and integrated thermodynamic, structural, and computational investigations of host–guest systems to elucidate the key factors governing noncovalent supramolecular interactions.

## Experimental Section

### Synthesis of 5,11,17,23-Tetra-*p*-*tert*-butyl- 25,26,27,28-tetrapropanone-calix[4]arene (**L**_**p**_ and **L**_**c**_)

In a 100 cm^3^ round-bottom flask, *tert*-butylcalix­[4]­arene (4.00 g, 6.2 mmol), potassium carbonate (4.00 g, 29 mmol), and sodium iodide (900 mg, 6.0 mmol) were suspended in 50 cm^3^ of dry acetone. To the stirred mixture, chloroacetone (5.0 cm^3^, 63 mmol) was added dropwise, and the mixture was heated using an oil bath under reflux for 48 h under argon. Acetone was evaporated and the residue was portioned between DCM and water. The organic layer (white suspension) was separated, and the water layer was extracted with additional DCM. Organic layers were combined and evaporated without drying. The residue was dissolved in 100 cm^3^ of boiling acetone and filtered to remove unreacted *tert*-butylcalix­[4]­arene. The filtrate was evaporated and dissolved in 100 cm^3^ boiling benzene. After 2 days, the mixture was filtered giving 1.2 g (22%) of white crystalline solid **L**_**c**_ (^1^H NMR spectrum of the compound is presented in Figure S1). Filtrate was evaporated, and the residue was purified using column chromatography (silica, EtOAc/acetone). Fractions containing **L**_**p**_ were combined and evaporated. The compound was further purified by crystallization from acetonitrile. Isolation yielded 180 mg (3%) of white crystalline solid **L**_**p**_.

### Characterization of **L**_**p**_

^1^H and ^13^C spectra of **L**_**p**_ and assignation of ^1^H and ^13^C NMR peaks are presented in Table S1 and Figures S2 and S3. Details of ^1^H and ^13^C NMR spectra and HRMS analysis of **L**_**p**_ are provided in the Supporting Information.

### Determination of Crystal Structures

The crystal and molecular structures of the free **L**_**p**_ and three **L**_**c**_ complexes were determined by single-crystal X-ray diffraction. In all cases, the crystals were obtained by evaporation of solvent from the acetonitrile solution of a ligand or complex.

Diffraction measurements for **L**_**p**_·MeCN were performed on a Xcalibur Kappa CCD X-ray diffractometer, while the remaining crystals were measured using an Oxford Diffraction XtaLAB Synergy CCD X-ray diffractometer.^62^ The structures were solved by the direct methods using SHELXS or SHELXT and refined and SHELXL programs.^62^ The structural refinement was performed on *F* ^2^ by using all data. The C–H hydrogen atoms were placed in calculated positions and treated as riding on their parent atoms with some exceptions which will be listed below. All calculations were performed and the drawings were prepared using WinGX crystallographic suite of programs.^63^ The crystal data and measurement details are given in Table S1. Further details are available from the Cambridge Crystallographic Data Centre^58^ with quotation numbers 2442046 and 2470446–2470448.

In the structure of **L**_**p**_·MeCN, one of the terminal acetyl groups was modeled as disordered over two orientations with 18:19 occupancy. The solvent molecule was also disordered over two positions (55:45 occupancy); however, here the methyl hydrogen atoms could not be modeled in a meaningful way and were therefore left out of the structural model. A number of restraints on bond lengths and angles, as well as atomic displacement parameters, were necessary in order to successfully model the disorder. Additionally, several of the terminal *tert*-butyl and 2-oxopropyl groups also appear to be disordered (as evidenced by large ADP-s); however, attempts to model this disorder did not yield any improvement and were therefore abandoned.

The crystal of [Ba­(ClO_4_)**L**_**c**_(H_2_O)]­(ClO_4_)·2.5MeCN·2H_2_O was found to be a twin with a component ratio ca. 82:18. In addition, there was also a severe disorder of *tert*-butyl groups, perchlorate anions, and solvent molecules. For this reason, multiple restraints on bond lengths and angles as well as atomic displacement parameters (generally for the peripheral parts of the molecule as well as the solvent) had to be imposed. Also, the severity of the disorder has precluded modeling of hydrogen atoms on noncoordinated water molecules (as they could not either be located from the electron density map, or unequivocally inferred from a potential hydrogen-bonding network). The hydrogen atoms of the coordinated water molecules also could not be located form the electron density map but were treated as riding on parent oxygen atoms, and their direction was determined based on the presence of potential hydrogen-bond acceptors.

In the structure of [Na_4_(ClO_4_)_2_ **L**_**c**2_(H_2_O)_3_]­(ClO_4_)_2_·6MeCN, three (out of 8 symmetrically independent) *t*-butyl groups were modeled as disordered over two positions. Another two *t*-butyl groups show signs of potential disorder; however, as the attempt to model them as “split” over two orientations did not seem to benefit the overall quality of the model, the attempt was abandoned. One of the solvent acetonitrile molecules was also modeled as disordered over two positions, which required stringent restraints on bond lengths and angles. The hydrogen atoms on water molecules were located from the electron density map and freely refined, with exception of one water molecule where one of the O–H distances and the H–O–H angle had to be fixed.

### Physicochemical Studies

#### Materials

The salts used for investigation of **L**_**c**_ and **L**_**p**_ complexation properties were LiClO_4_ (Sigma-Aldrich, 99.99%), NaClO_4_ (Sigma-Aldrich, 98+%), KClO_4_ (Merck, p.a.), KCl (Gram-mol, 99.5%), RbCl (Sigma-Aldrich, ≥99.8%), RbI (Sigma-Aldrich, 99.9%), CsI (Merck, 99.5%), Mg­(ClO_4_)_2_·6H_2_O (Sigma-Aldrich, 99.99%), magnesium triflate (Mg­(trf)_2_, Sigma-Aldrich, 99.9%), Ca­(ClO_4_)_2_·4H_2_O (Sigma-Aldrich, 98+%), calcium triflate (Ca­(trf)_2_, Sigma-Aldrich, 99.9%), Sr­(ClO_4_)_2_·3H_2_O (Merck, p.a.), Ba­(ClO_4_)_2_ (Fluka, ≥98%), and barium triflate (Ba­(trf)_2_, Sigma-Aldrich, 98%). Perchlorate and triflate salts were used due to the inertness with respect to ion pairing and the fact that these ions do not absorb UV light in the investigated spectral range. All solutions were prepared by direct weighing and dissolution of the ligands and salts in volumetric flasks.

The solvents used for microcalorimetric and spectrophotometric investigations were acetonitrile (J. T. Baker, HPLC Gradient grade), used without further purification, methanol (J. T. Baker, HPLC Gradient grade), which was distilled prior to use, and CHCl_3_ (CARLO ERBA, RPE-For analysis-ISO). Solvents used for NMR investigations were CD_3_CN (Eurisotop, 99.8% D), CD_3_OD (Eurisotop, 99.8% D), and CDCl_3_ (Eurisotop, 99.8% D).

18-crown-6 (Sigma-Aldrich, 99%) and BaCl_2_ (Sigma-Aldrich, 99.9%) were used for calorimeter calibration.

#### Cation Complexation

The complexation thermodynamics of alkali and alkaline earth metal cations with **L**_**p**_ and **L**_**c**_ was investigated at 25 °C by means of a MicroCal VP-ITC titration calorimeter. For this purpose, the heat effects resulting from automatized addition of alkali or alkaline earth metal cation salts (*c* = 2 × 10^–4^ to 8 × 10^–2^ mol dm^–3^) into the ligand solution (*c*_0_ = 1 × 10^–4^ to 3 × 10^–4^ mol dm^–3^, *V*_0_ = 1.45 cm^3^) were recorded. The obtained enthalpy changes were corrected for titrant dilution and processed using the MicroCal OriginPro 7.0 and OriginPro 7.5 programs. The instrument reliability was verified by carrying out the titrations of 18-crown-6 by barium chloride in water at 25.0 °C. The obtained results (log *K* = 3.76, Δ_r_ *H* = −31.53 kJ mol^–1^) were in very good agreement with the literature values (log *K* = 3.77, Δ_r_ *H* = −31.42 kJ mol^–1^).^64^

The cation binding by the partial and regular *cone* calixarene was also investigated spectrophotometrically. Spectral changes of receptor solutions (*c*_0_ = 1 × 10^–4^ to 2 × 10^–4^ mol dm^–3^, *V*_0_ = 2.3 cm^3^) were recorded upon stepwise addition of the cation salt solutions (*c* = 2 × 10^–3^ mol dm^–3^ to 3 × 10^–2^ mol dm^–3^) into the quartz cell (Hellma, Suprasil QX, *l* = 1 cm) by means of an Agilent Cary 60 spectrophotometer at (25.0 ± 0.1) °C. Absorbances were collected at 1 nm intervals, with an integration time of 0.2 s, and the data were processed using the HypSpec program.^65^

#### Dissolution Calorimetry and Ligand Solubility

Dissolution enthalpies of the partial *cone* receptor in acetonitrile and methanol at 25.0 °C were determined by means of a TAM IV (TA Instruments) dissolution microcalorimeter. **L**_**p**_ (1.6–8.4 mg) was weighed directly into the sample cartridges (*V* = 40 μL). Following thermal equilibration, the cartridges were introduced into the calorimetric cell containing 16 cm^3^ of solvent under constant stirring (ν = 60 rpm), and the resulting passive mode heat flow was recorded (5 s intervals). The enthalpy changes obtained by integration of the calorimetric signals were corrected for blank (introduction of an empty cartridge into the solvent), and the dissolution enthalpies were determined by dividing the corresponding values with the amount of dissolved calixarene.

Solubilities of **L**_**p**_ in methanol and acetonitrile were determined spectrophotometrically. For this purpose, the suspensions of the solid macrocycle were prepared and left to equilibrate overnight at 25.0 °C while constantly agitated using a magnetic stirrer. After the equilibrium was reached, aliquots of saturated solutions were carefully sampled, and their concentrations were determined by means of an Agilent Cary 60 spectrophotometer equipped with a thermostatting device (ϑ = (25.0 ± 0.1) °C). The molar absorption coefficients of **L**_**p**_ were determined by measuring the absorbances of solutions containing known amounts of the receptor.

#### Solvent Inclusion Studies

The solvent inclusion within the hydrophobic cavity of **L**_**p**_ as well as **L**_**c**_ and **L**_**p**_ complexes was investigated by means of ^1^H NMR spectroscopy in CDCl_3_ at 25.0 °C using TMS as an internal standard. The proton spectra of corresponding solutions (*c*_0_ ≈ 5 × 10^–4^ to 6 × 10^–3^ mol dm^–3^, *V*_0_ = 0.5 cm^3^) were recorded upon stepwise addition of MeCN and MeOH (*c* = 0.8–6 mol dm^–3^) dissolved in CDCl_3_ using a Bruker AVANCE III HD 400 MHz/54 mm Ascend spectrometer equipped with a 5 mm PA BBI 1H/D-BB probe head with *z*-gradient and automated tuning and matching accessory. Overall, 64K data points were used, with a spectral width of 20 ppm and recycle delay of 1.0 and 16 scans. The data were processed by means of the HypNMR program.^65^

The solvent inclusion into the *basket* of Ca**L**_**c**_^2+^ was further explored by isothermal titration calorimetry using a MicroCal VP-ITC titration calorimeter. The heat effects were recorded upon the addition of MeCN dissolved in CHCl_3_ into the solution of Ca**L**_**c**_^2+^. The obtained enthalpy changes were corrected for titrant dilution. The data analysis was carried out using Microcal OriginPro 7.0 and OriginPro 7.5 programs.

#### Computational Investigations of Receptors and Their Complexes

The molecular dynamics simulations were carried out by means of a GROMACS package (version 2021.7).^66–71^ Intramolecular and nonbonded intermolecular interactions were modeled by the CHARMM36 (Chemistry at HARvard Macromolecular Mechanics) force field.^72^ The initial conformation of **L**_**c**_ was a squashed *cone*, and that of **L**_**p**_ was a partial *cone*. The complex simulations were initiated by placing the cation in the center of the binding site between the ether and carbonyl oxygen atoms, energy minimization, and *NVT* production simulation in vacuo. The obtained complexes were solvated in a cubic box of a previously equilibrated solvent with a periodic boundary condition.

An energy minimization procedure was again performed, followed by a 50.5 ns of *NpT* production simulation with a Parrinello–Rahman barostat,^73,74^ with the time constant of 1 ps. The pressure (1 bar) and temperature (298 K) were maintained constant during the simulation. The first 0.5 ns of production simulation was discarded during data analysis. The integrator used for the propagation and the temperature control was stochastic dynamics algorithm^75^ with a time step of 1 fs. The cutoff radius for nonbonded van der Waals and short-range Coulomb interactions was set to 15 Å. Long-range Coulomb interactions were treated by the Ewald method.^76^ Average molecular structures of calixarene–cation complexes were obtained by principal component analysis (PCA) on a coordination matrix whose rows contained distances between the metal cation, ether, and carbonyl oxygen atoms. Angles between metal cations and carbonyl groups were added to the coordination matrix, as well. The chosen structures were closest to the centroids of the most populated clusters in space defined by the first two principal components. The coordination matrix of free ligands was constructed of distances between phenol and carbonyl oxygen atoms and the geometric center of phenol oxygen atoms. The angles between this geometric center and carbonyl groups were also used. Figures of molecular structures were created using VMD software.^77^

In quantum chemical studies, optimizations of geometries for all investigated complexes were performed using the hybrid functional B3LYP^78,79^ with the D3 version of Grimme’s dispersion correction^80^ and Becke-Johnson damping in combination with the def2-SVP^81,82^ basis set. The initial geometries of complexes with and without the solvent molecule for the optimization procedure were assembled from the first optimized structure and reoptimized. To confirm that the obtained geometries were local minima, harmonic frequency calculations were performed and analyzed.^83,84^ The reaction standard enthalpies, entropies, and Gibbs energies were calculated at *T* = 298.15 K and *p* = 101,325 Pa. From these values, appropriate standard enthalpies, entropies, and Gibbs energies of binding were estimated.^85^ All quantum chemical calculations were carried out using the Gaussian 16 program package.^86^ The least-squares fit plane through ether oxygen atoms of the ligands was determined by using the advanced regression module implemented in our code ***moonee***.^87,88^

## Supplementary Material



## Data Availability

The data underlying this study are available in the published article and its Supporting Information.

## Notes

Characterization of compounds **L**_**c**_ and **L**_**p**_ (^1^H and ^13^C NMR, HRMS); spectrophotometric, calorimetric, and ^1^H NMR titrations; additional details regarding crystal structures; results of MD simulations and DFT calculations; thermodynamic dissolution parameters of **L**_**c**_ and **L**_**p**_; and additional thermodynamic cycles (PDF)
