# Supplementary material for: The “Missing Link”, Allostery and Synergism—Hosting of Metal Cations by Regular and Partial Cone Calix[4]arene Isomers
Source: ACS Org Inorg Au. 2025 Nov 12;6(1):88–103. doi: 10.1021/acsorginorgau.5c00095 (PMC12879178; doi:10.1021/acsorginorgau.5c00095)
Supplement: Supplementary file 1 [file gg5c00095_si_001.pdf]

The “missing link”, allostery and synergism – hosting of metal cations by regular and partial *cone* calix[4]arene isomers

Matija Modrušan, Nikola Cindro, Marija Cvetnić, Andrea Usenik, Slavica Petrović, Jakov Borovec, Katarina Leko, Karla Kukina Gradečak, Vladimir Stilinović, Gordan Horvat, Tomica Hrenar, Josip Požar* and Vladislav Tomišić*

Department of Chemistry, Faculty of Science, University of Zagreb, Horvatovac 102a, 10000 Zagreb, Croatia

Supporting Information

Table of contents

S1. Experimental 3

S1.1. Synthesis of 5,11,17,23-tetra-*p*-*tert*-butyl-25,26,27,28-tetrapropanone-calix[4]arene (**L$_p$** and **L$_c$**) 3

S1.1.1. Characterization of **L$_p$** 4

S2. Results 8

S2.1. Crystal structures 8

S2.2. MD simulations of free **L$_c$** and **L$_p$** 13

S2.2.1. Solvent: acetonitrile 13

S2.2.2. Solvent: methanol 16

S2.3. MD simulations of **L$_c$** and **L$_p$** complexes 18

S2.3.1. Solvent: acetonitrile 18

S2.3.2. Solvent: methanol 35

S2.4. Alkali metal cation complexation 44

S2.4.1. Solvent: acetonitrile 44

S2.4.2. Solvent: methanol 49

S2.5. Alkaline earth metal cation complexation 52

S2.5.1. Solvent: acetonitrile 52

S2.5.2. Solvent: methanol 59

S2.6. Dissolution calorimetry 63

S2.7. Solubility measurements 64

S2.8. NMR and ITC investigations of solvent inclusion into $\mathbf{L_c}$, $\mathbf{L_p}$, and their complexes 65

S2.9. Solvent effect on the thermodynamics of cation complexation 72

S2.10. Quantum chemical studies 74

References 177

S1. Experimental

S1.1. Synthesis of 5,11,17,23-tetra-*p*-*tert*-butyl-25,26,27,28-tetrapropanone-calix[4]arene ($\mathbf{L_p}$ and $\mathbf{L_c}$)

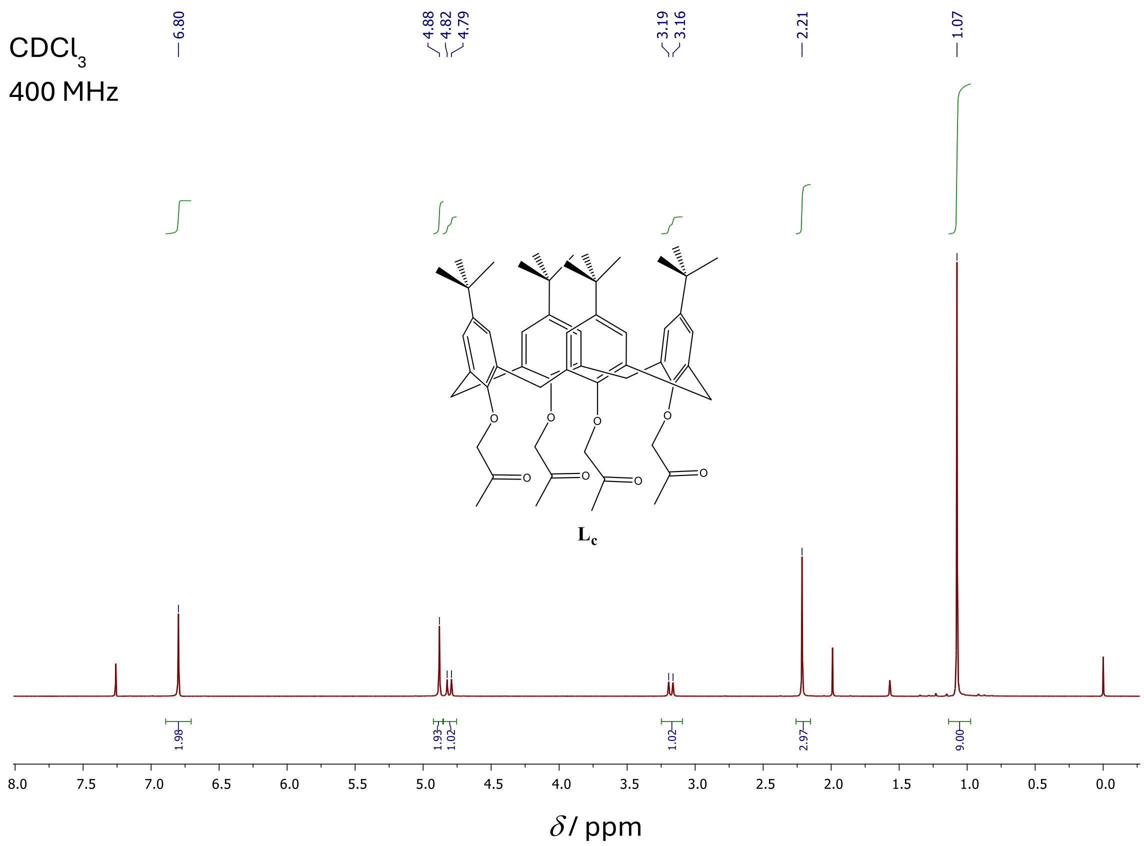

Figure S1. ^{1}H NMR spectrum of calixarene $\mathbf{L_c}$. Proton chemical shifts are consistent with the previously reported ones.[1]

S1.1.1. Characterization of $\mathbf{L_p}$

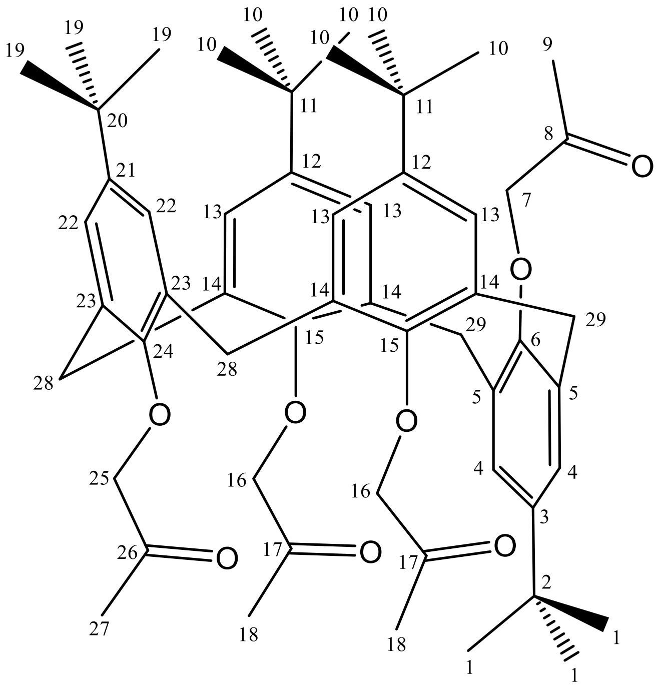

Figure S2. Structure of $\mathbf{L_p}$ with numbered proton and carbon atoms.

Table S1. Assignation of ^{1}H and ^{13}C NMR peaks to the proton and carbon atoms according to Figure S2.

proton/carbon no.	δ(^{1}H NMR) / ppm	δ(^{13}C NMR) / ppm
1	1.35	31.5
2	/	34.1
3	/	146.2
4	6.80	125.6
5	/	133.0
6	/	152.6
7	3.12	73.0
8	/	205.5
9	-0.43	24.7
10	1.15	31.3
11	/	34.0
12	/	146.3
13	7.15	125.5<br>126.4
14	/	131.9<br>135.0
15	/	153.3
16	4.40	77.0
17	/	206.1
18	1.86	26.1
19	1.23	31.6
20	/	34.1
21	/	146.4
22	7.18	126.5
23	/	134.3
24	/	153.3
25	4.74	79.8
26	/	208.2
27	1.94	26.0
28	4.96<br>3.31	30.7
29	4.00<br>3.83	39.2

a)

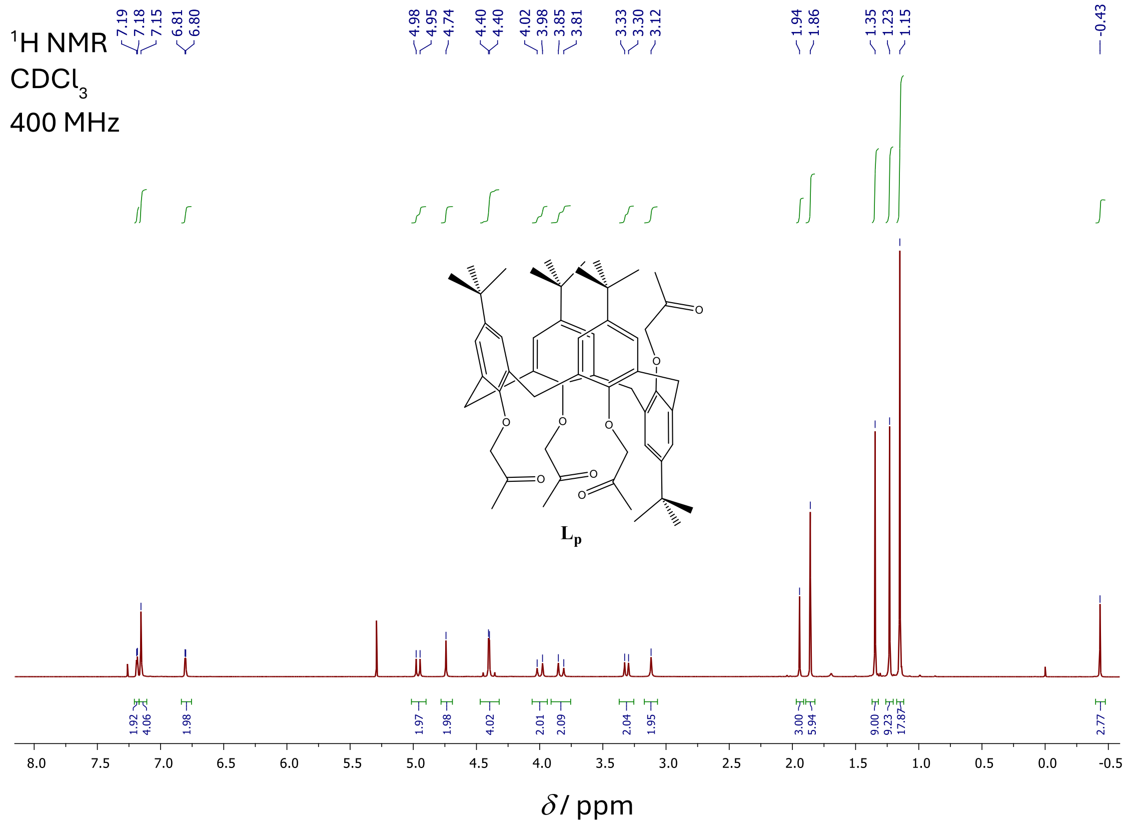

b)

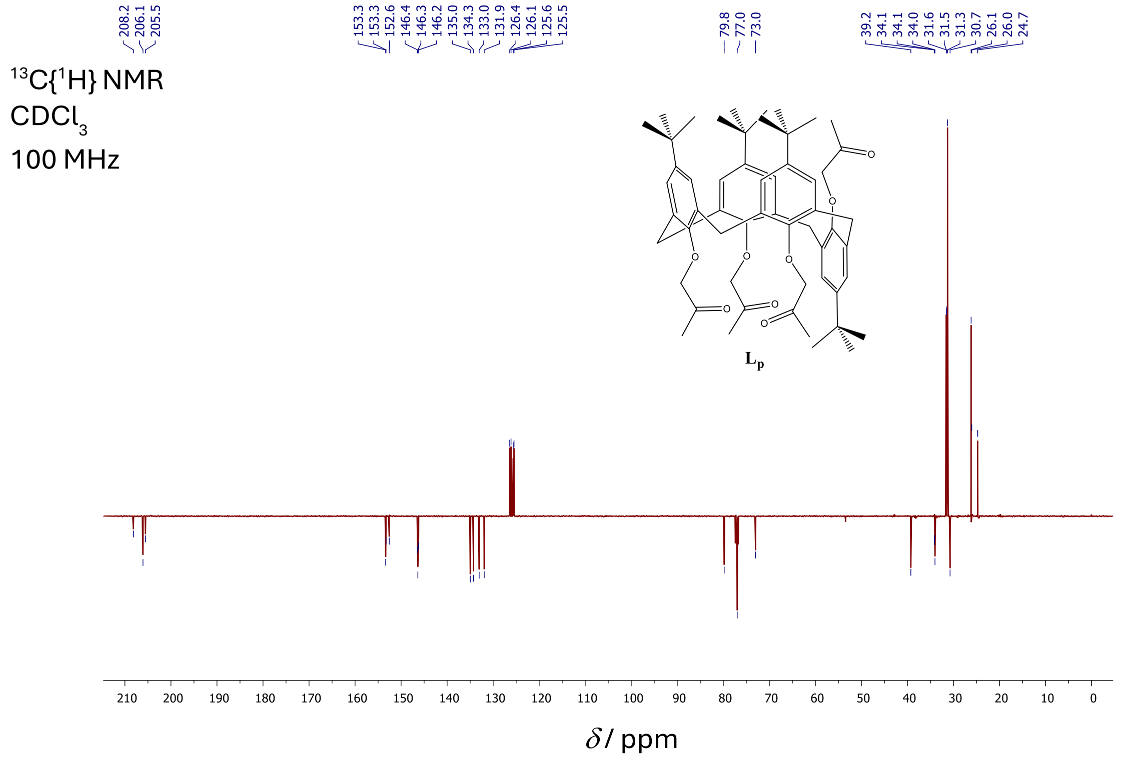

Figure S3. a) ^{1}H NMR and b) ^{13}C NMR spectra of $\mathbf{L_p}$ in $CDCl_3$ at 25.0 °C.

^{1}H NMR ($CDCl_3$, 400 MHz), δ / ppm: 7.18 (d, 2H, J = 2.2 Hz), 7.15 (s, 4H), 6.80 (d, 2H, J = 2.2 Hz), 4.96 (d, 2H, J = 12.5 Hz), 4.74 (s, 2H), 4.40 (s, 4H), 4.00 (d, 2H, J = 17.2 Hz), 3.83 (d, 2H, J = 17.2 Hz), 3.31 (d, 2H, J = 12.5 Hz), <u>3.12</u> (s, 2H), 1.94 (s, 3H), <u>1.86</u> (s, 6H), <u>1.35</u> (s, 9H), 1.23 (s, 9H), <u>1.15</u> (s, 18H), −0.43 (s, 3H).

^{13}C{^{1}H} NMR ($CDCl_3$, 100 MHz), δ / ppm: 208.2, 206.1, 205.5, 153.3, 153.3, 152.6, 146.4, 146.3, 146.2, 135.0, 134.3, 133.0, 131.9, 126.4, 126.1, 125.6, 125.5, 79.8, 77.0, 73.0, 39.2, 34.1, 34.1, 34.0, 31.6, 31.5, 31.3, 30.7, 26.1, 26.0, 24.7

HRMS (ESI): m/z: $[M + Na]^+$ Calcd for $C_{56}H_{72}O_8Na$: 895.5125, found 895.5131.

S2. Results

S2.1. Crystal structures

Table S2. The crystal data and measurement details for $\mathbf{L_p}$ · MeCN and $\mathbf{L_c}$ complex adducts.

	$\mathbf{L_p}$ · MeCN	[Ca$\mathbf{L_c}$MeCN]$(ClO_4)_2$ · 2MeCN	[Ba(ClO_4)$\mathbf{L_c}$(H_2O)](ClO_4) · 2.5MeCN · 2H_2O	[$Na_4(ClO_4)_2$$\mathbf{L_{c2}}$$(H_2O)_3$]$(ClO_4)_2$ · 6MeCN
Molecular formula	$C_{58}H_{75}NO_8$	$C_{62}H_{81}CaCl_2N_3O_{16}$	$C_{61}H_{82}BaCl_2N_{2.5}O_{19}$	$C_{124}H_{168}Na_2Cl_4N_6O_{35}$
M_r	914.19	1235.27	1362.53	2536.39
Crystal system	Monoclinic	Triclinic	Monoclinic	Triclinic
Space group	*C c*	*P* –1	*P* $2_1/c$	*P* –1
a / Å	13.416(2)	12.56289(14)	13.01310(10)	16.6090(2)
b / Å	21.9653(19)	12.63722(14)	51.7357(5)	20.6540(3)
c / Å	18.5788(16)	20.2080(2)	20.6515(2)	22.3603(3)
α / °	90	93.8826(8)	90	67.2880(10)
β / °	90.601(9)	93.3537(8)	90.1550(10)	69.2410(10)
γ / °	90	92.3740(9)	90	82.997(2)
V / Å^3	5474.7(12)	3192.05(6)	13903.4(2)	6615.39(17)
Z	4	2	8	2
ρ_{calc} / g cm^{-3}	1.105	1.285	1.302	1.273
T / K	293(2)	170.0(2)	100.0(2)	100.0(2)
λ(MoK_α) / Å,	0.71073	1.54184	1.54184	1.54184
μ / mm^{-1}	0.072	2.180	5.709	1.586
F(000)	1964	1312	5660	2692
θ range/ °	4−25	4−70	2.3−75	2.8−76.8
hkl range	−15, 15; −26, 26; −22, 22	−15, 15; −15, 5; −19, 24	−15, 16; −62, 64; −25, 23	−20, 20; −25, 25; −24, 28
N(reflections, measured)	23555	46362	436962	93716
N(reflections, independent)	9800	11706	28263	26636
N(reflections, $I > 4\sigma(I)$)	4951	11427	24355	24373
Number of parameters	663	776	1632	1737
$\Delta\rho_{max}$, $\Delta\rho_{min}$ / e Å^{-3}	0.287, −0.199	1.113, −1.019	1.859, −3.558	1.945, −0.985
$R[F^2>4\sigma(F^2)]$	0.0634	0.0691	0.1122	0.0604
$wR(F^2)$	0.1682	0.1901	0.3162	0.1678
Goodness-of-fit, *S*	0.896	1.034	1.076	1.048

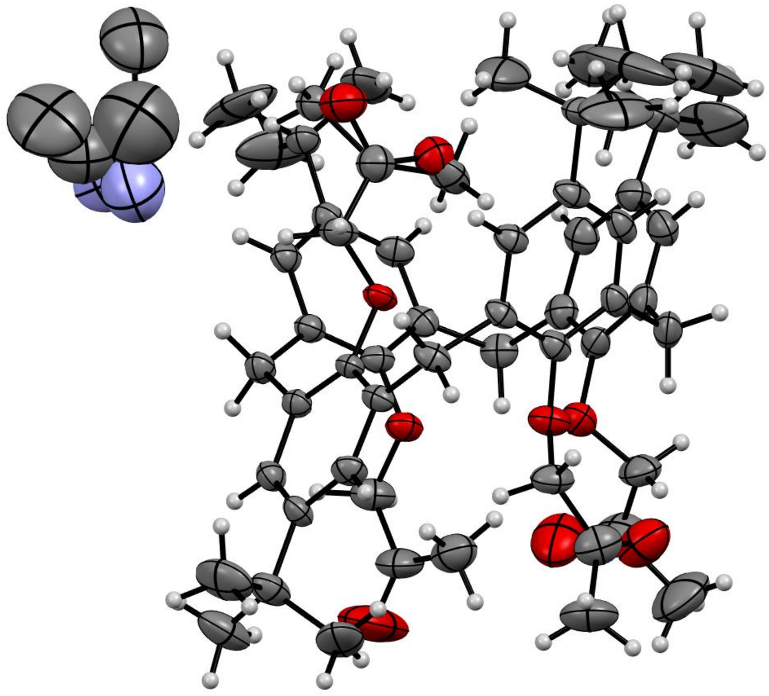

Figure S4. ORTEP plot of the asymmetric unit in the crystal structure of $\mathbf{L_p}$ · MeCN. The displacement ellipsoids are shown with 30 % probability, and hydrogen atoms are shown as small spheres of arbitrary radii.

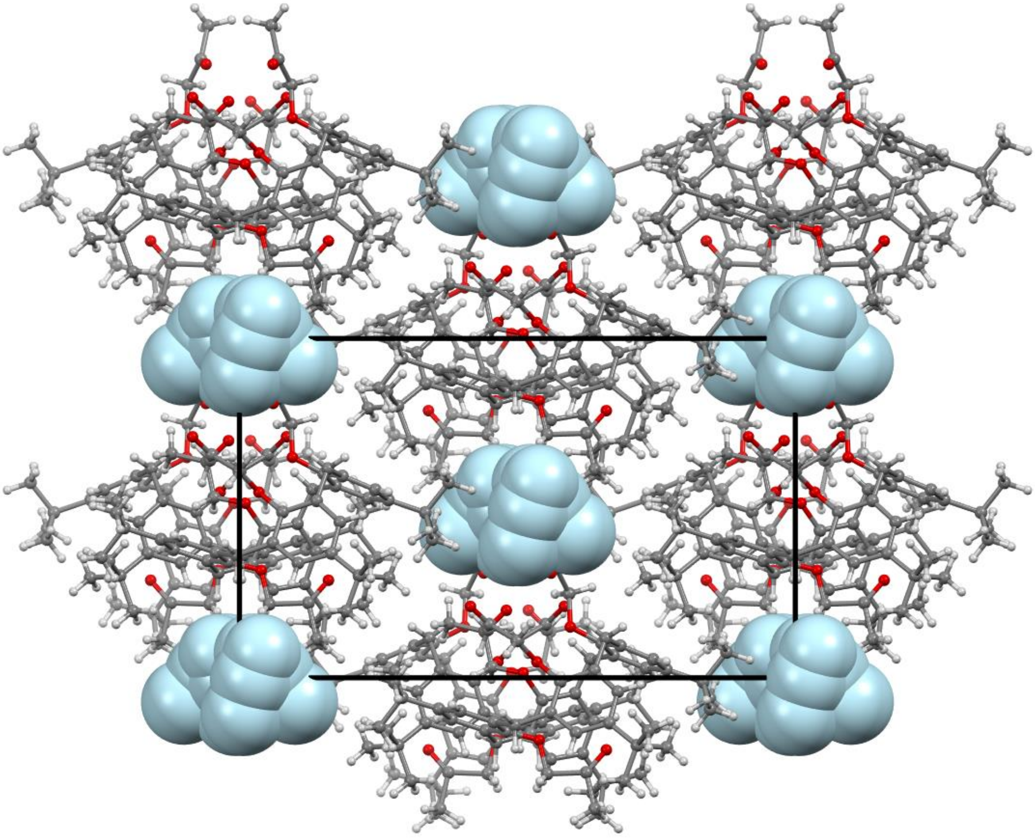

Figure S5. Crystal packing of $\mathbf{L_p}$ · MeCN viewed along the crystallographic *c* axis. The disordered acetonitrile molecules are shown as light blue space-filling models.

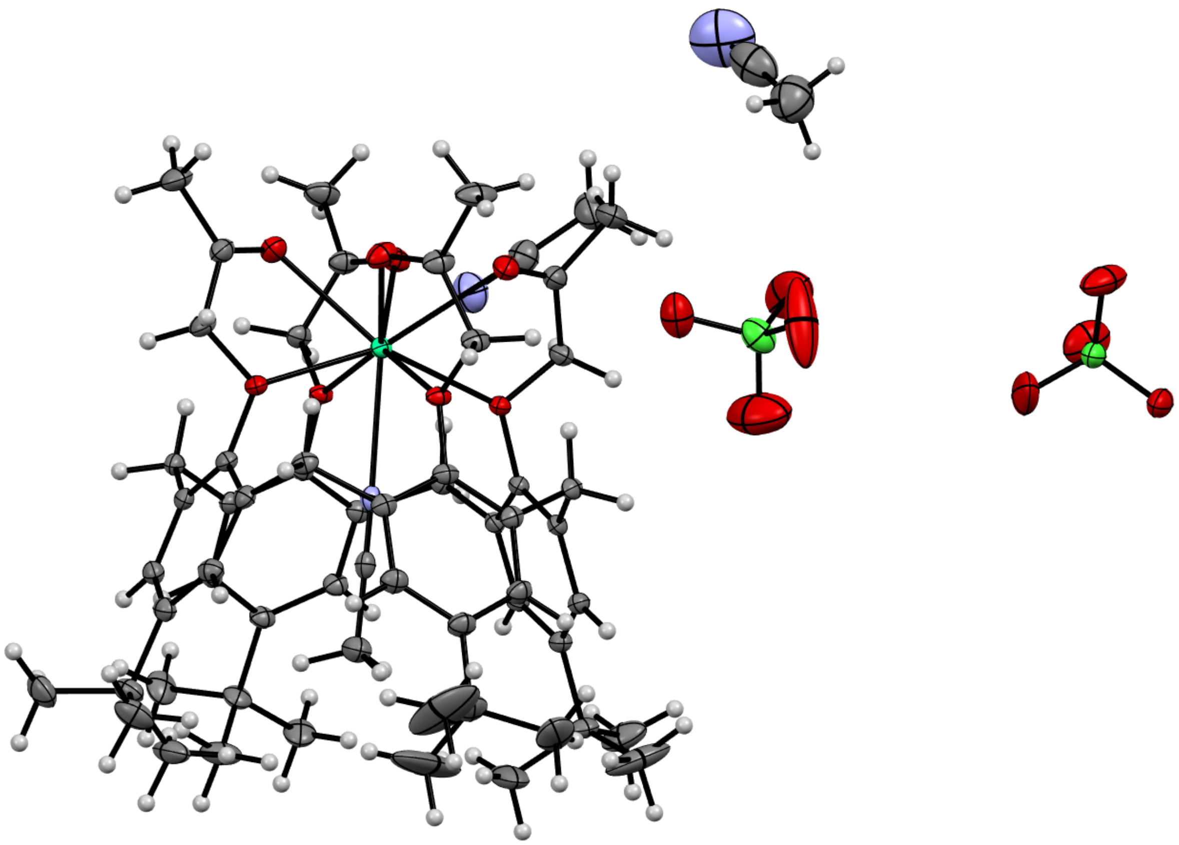

Figure S6. ORTEP plot of the asymmetric unit in the crystal structure of [Ca$\mathbf{L}_c$MeCN](ClO_4)$_2$ · 2MeCN. The displacement ellipsoids are shown with 30 % probability, and hydrogen atoms are shown as small spheres of arbitrary radii.

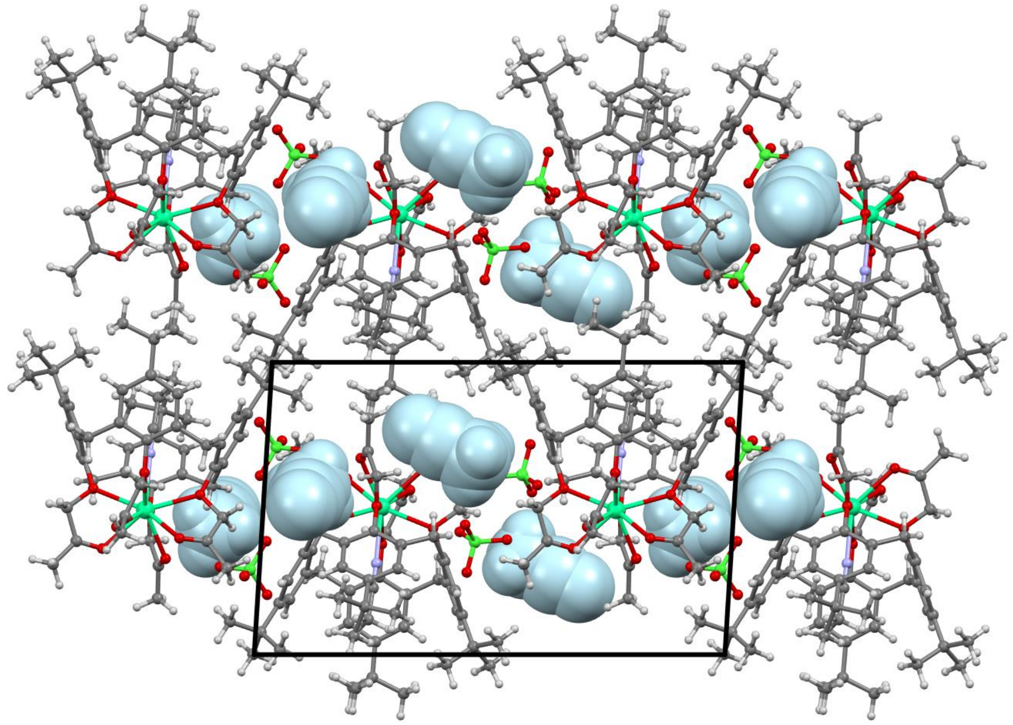

Figure S7. Crystal packing of [Ca$\mathbf{L}_c$MeCN](ClO_4)$_2$ · 2MeCN viewed along the crystallographic *b* axis. The uncoordinated acetonitrile molecules are shown as light blue space-filling models.

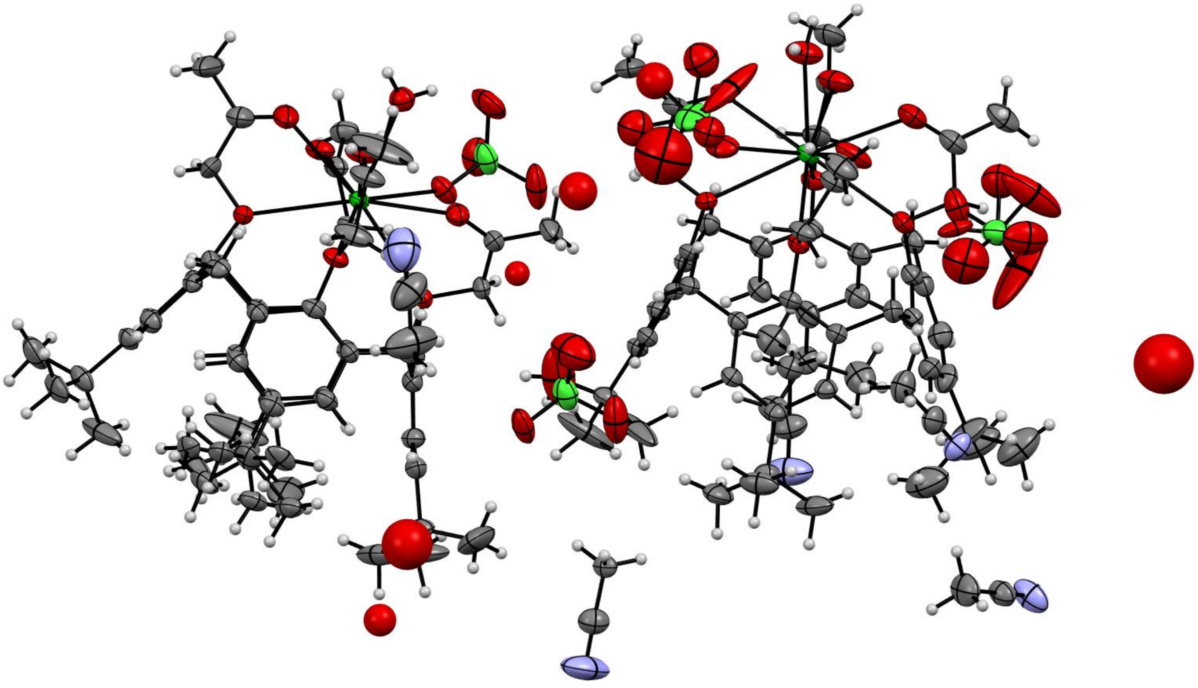

Figure S8. ORTEP plot of the asymmetric unit in the crystal structure of $[Ba(ClO_4)\mathbf{L_c}(H_2O)](ClO_4) \cdot 2.5MeCN \cdot 2H_2O$. The displacement ellipsoids are shown with 30 % probability, the isotopically refined oxygen atoms as spheres with 30 % probability, and hydrogen atoms are shown as small spheres of arbitrary radii.

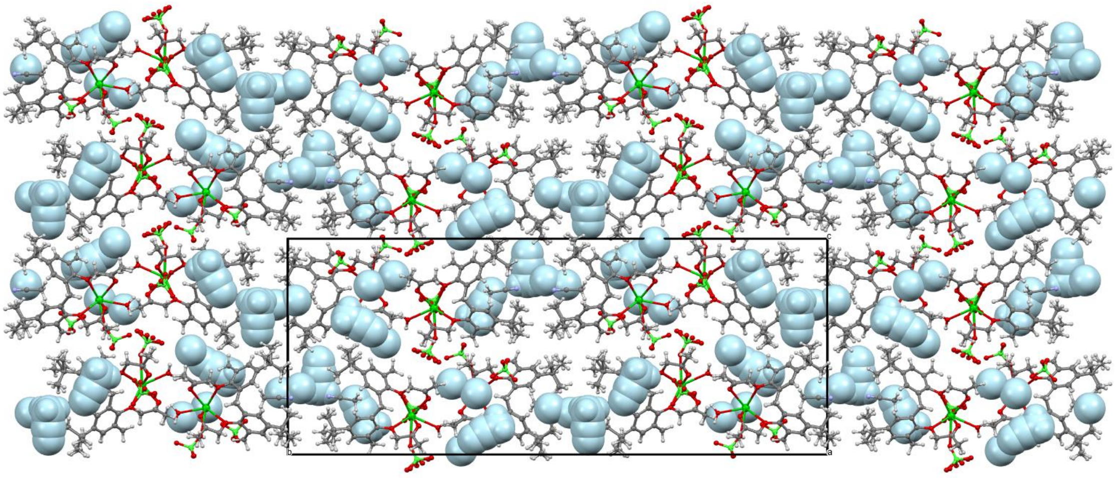

Figure S9. Crystal packing of $[Ba(ClO_4)\mathbf{L_c}(H_2O)](ClO_4) \cdot 2.5MeCN \cdot 2H_2O$ viewed along the crystallographic *a* axis. The uncoordinated water molecules as well as acetonitrile molecules not included in the calixarene cone are shown as light blue space-filling models.

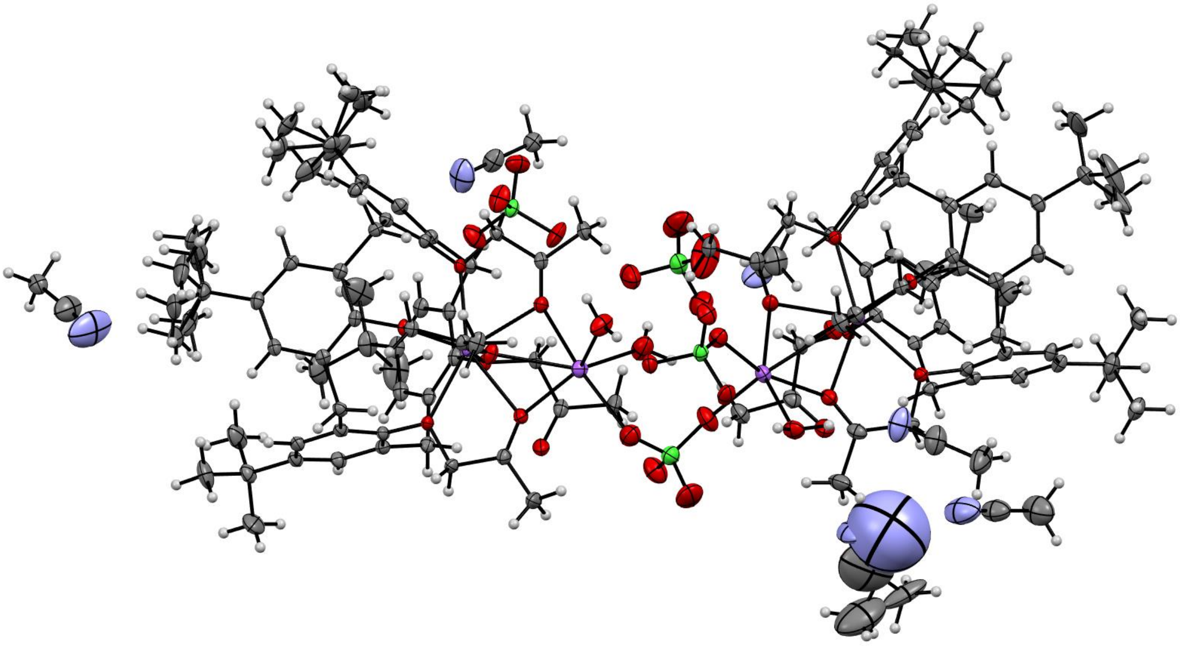

Figure S10. ORTEP plot of the asymmetric unit in the crystal structure of $[Na_4(ClO_4)_2\mathbf{L_{c2}}(H_2O)_3](ClO_4)_2 \cdot 6MeCN$. The displacement ellipsoids are shown with 50 % probability, and hydrogen atoms are shown as small spheres of arbitrary radii.

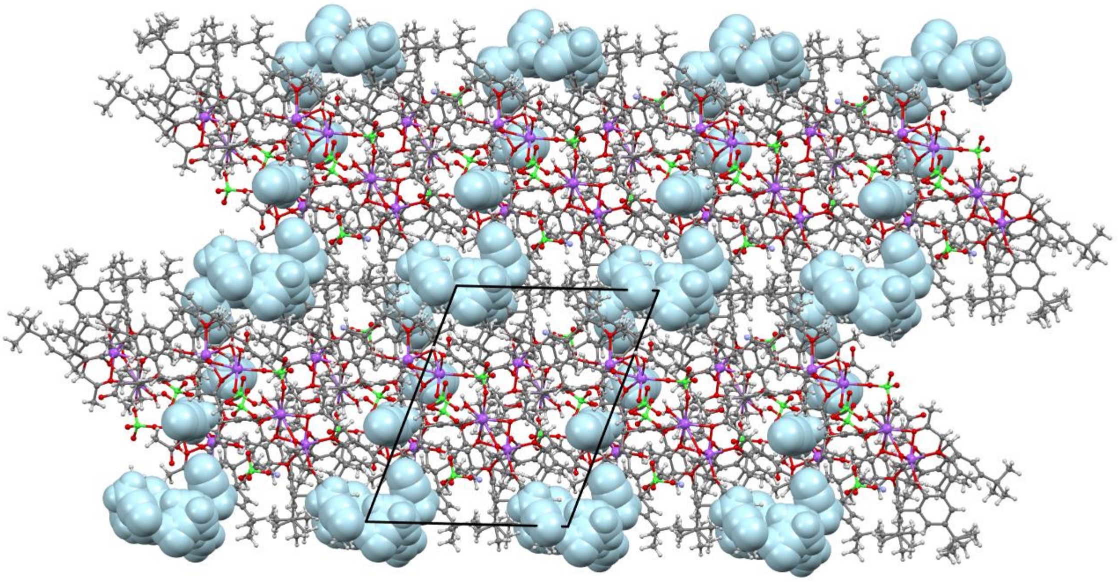

Figure S11. Crystal packing of $[Na_4(ClO_4)_2\mathbf{L_{c2}}(H_2O)_3](ClO_4)_2 \cdot 6MeCN$ viewed along the crystallographic *b* axis. The acetonitrile molecules not included in the calixarene cone are shown as light blue space-filling models.

S2.2. MD simulations of free **L**$_\mathbf{c}$ and **L**$_\mathbf{p}$

S2.2.1. Solvent: acetonitrile

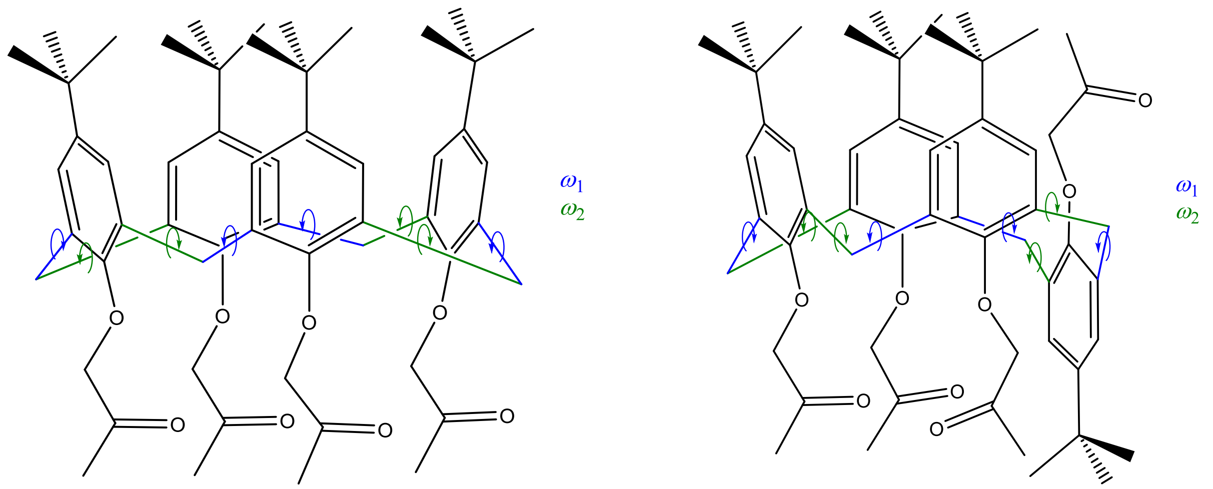

Figure S12. Structures of calixarenes **L**$_\mathbf{c}$ and **L**$_\mathbf{p}$ with the definition of ω_1 and ω_2 torsion angles.

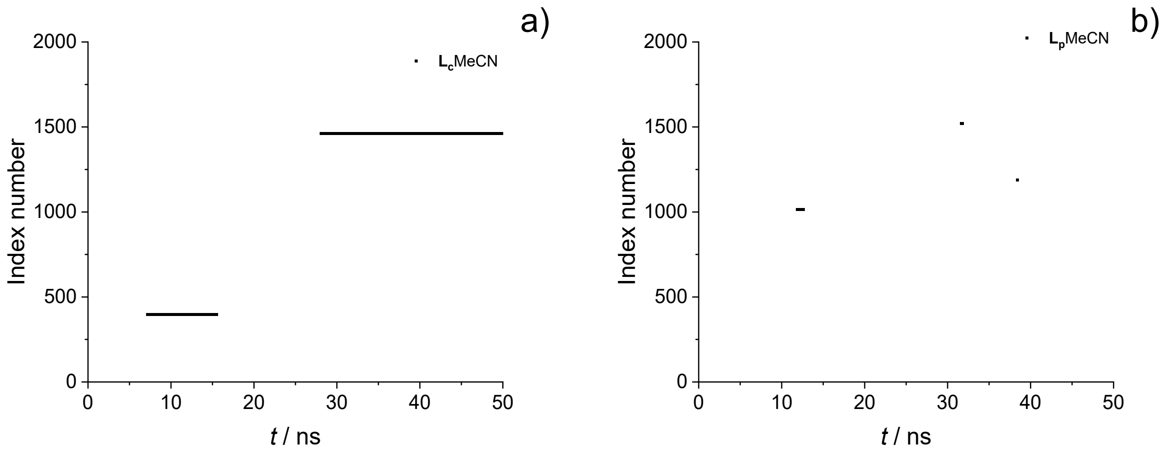

Figure S13. Index number of acetonitrile molecules that occupy hydrophobic cavities of a) **L**$_\mathbf{c}$MeCN and b) **L**$_\mathbf{p}$MeCN during MD simulations in acetonitrile at 25 °C.

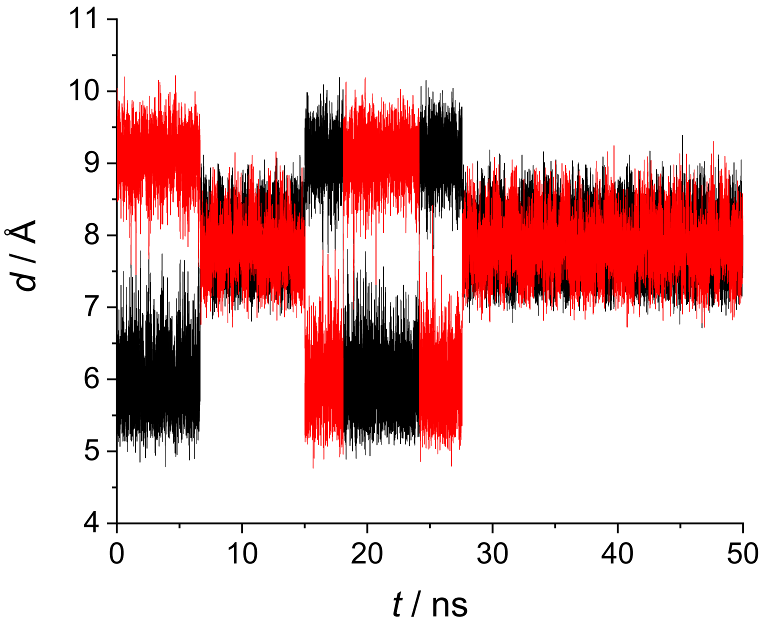

Figure S14. Distances between the opposing phenyl carbon atoms that are directly connected to the *tert*-butyl group during the MD simulation of $\mathbf{L_c}$ in MeCN.

Table S3. Energies of interactions of free $\mathbf{L_c}$ and $\mathbf{L_p}$ with solvent molecules in acetonitrile, occurrence time ratio of different chemical species, and structural parameters of calixarene *basket* in free receptors obtained by MD simulations in acetonitrile at 25 °C.

	Free $\mathbf{L_c}$		Free $\mathbf{L_p}$	
	$\mathbf{L_c}$MeCN	$\mathbf{L_c}$*	$\mathbf{L_p}$MeCN	$\mathbf{L_p}$*
E(**L**–MeCN) / kJ mol^{-1}	–489	–443	–482	–439
E(**L**–MeCN$_{incl}$) / kJ mol^{-1}	–45	/	–43	/
t_{total} / ns	50		50	
t / t_{total}	0.615	0.385	0.017	0.983
N(MeCN$_{incl}$)	2	/	3	/
$\bar{d}$ / Å	7.91	7.04	7.35	6.63
	7.84	8.04		
σ(d) / Å	0.36	1.52	0.28	0.88
	0.35	1.52		
|d – d_{ref}| / Å	0.29	1.66	0.51	1.26
	0.28	1.46		
ω_1 (σ(ω_1)) / °	82 (8)	75 (15)	79 (8)	72 (10)
	81 (8)	84 (15)	91 (9)	90 (11)
	82 (8)	76 (15)	131 (9)	123 (13)
	81 (8)	84 (15)	–124 (9)	–129 (13)
ω_2 (σ(ω_2)) / °	–81 (8)	–84 (15)	–90 (9)	–90 (11)
	–82 (8)	–76 (15)	–79 (8)	–73 (11)
	–81 (8)	–84 (15)	125 (9)	129 (13)
	–82 (8)	–75 (15)	–132 (9)	–123 (12)

$\bar{d}$ denotes average distance between opposing aryl carbon atoms connected to the *tert*-butyl groups and d_{ref} = 7.85 Å corresponds to C_{4v} *cone* conformation.
ω_1, ω_2 denotes two dihedral angles along the methylene bridge – benzyl covalent bond (Figure S2)
N(MeCN$_{incl}$) denotes the number of different acetonitrile molecules found in calixarene *basket*

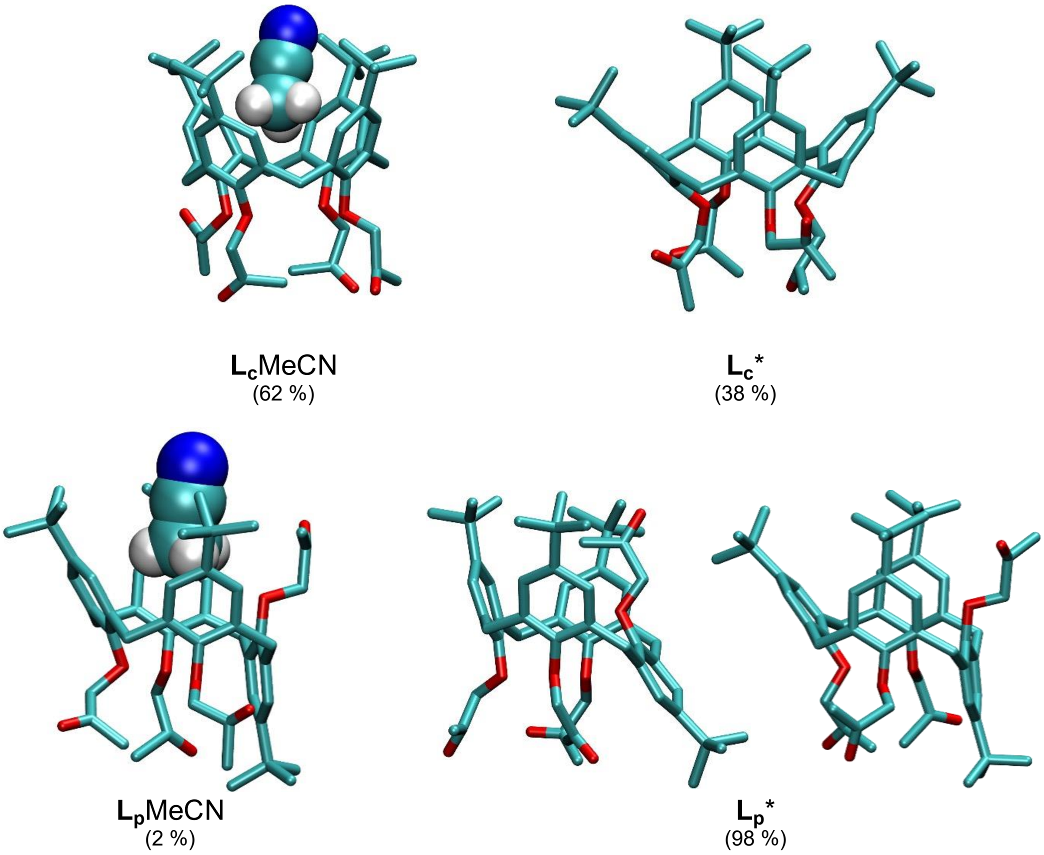

Figure S15. Representative structures of $\mathbf{L_c}$ and $\mathbf{L_p}$ and their MeCN adducts obtained by MD simulations at 25 °C. Hydrogen atoms of receptors are omitted for clarity. Numbers in parentheses represent percentages of total simulation time in which the species existed.

S2.2.2. Solvent: methanol

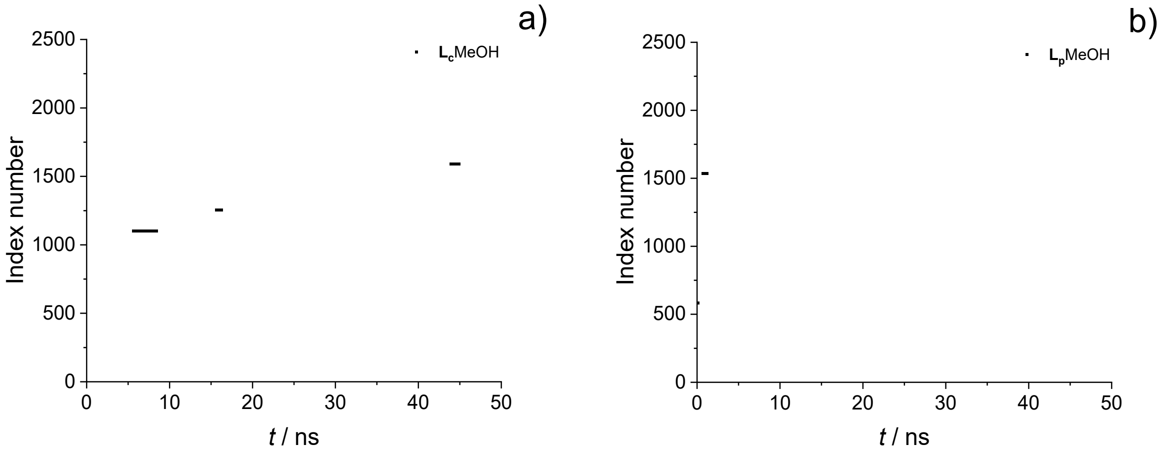

Figure S16. Index number of methanol molecules that occupy hydrophobic cavities of a) **L**$_c$MeOH and b) **L**$_p$MeOH during MD simulations in methanol at 25 °C.

Table S4. Energies of interactions of free **L**$_c$ and **L**$_p$ with solvent molecules in methanol, occurrence time ratio of different chemical species, and structural parameters of calixarene *basket* in the free receptors obtained by MD simulations in methanol at 25 °C.

	**L**$_c$MeOH	**L**$_c$*	**L**$_p$MeOH	**L**$_p$*
E(**L**–MeOH) / kJ mol^{-1}	–499	–455	–496	–451
E(**L**–MeOH$_{incl}$) / kJ mol^{-1}	–38	/	–49	/
t_{total} / ns	50		50	
t / t_{total}	0.088	0.912	0.010	0.990
N(MeOH$_{incl}$)	4	/	2	/
$\bar{d}$ / Å	7.85<br>7.89	7.47<br>7.62	7.34	6.47
$\sigma(d)$ / Å	0.37<br>0.38	1.59<br>1.59	0.25	0.84
$\lvert d - d_{ref}\rvert$ / Å	0.31<br>0.30	1.56<br>1.53	0.52	1.42
ω_1 ($\sigma(\omega_1)$) / °	82 (8)<br>82 (8)<br>81 (8)<br>82 (8)	79 (16)<br>81 (16)<br>79 (16)<br>81 (16)	78 (7)<br>91 (7)<br>132 (9)<br>–123 (8)	71 (10)<br>92 (11)<br>121 (12)<br>–127 (12)
ω_2 ($\sigma(\omega_2)$) / °	–82 (8)<br>–81 (8)<br>–82 (8)<br>–82 (8)	–81 (16)<br>–79 (16)<br>–81 (16)<br>–79 (16)	–92 (8)<br>–78 (7)<br>122 (9)<br>–130 (9)	–92 (11)<br>–71 (10)<br>127 (12)<br>–121 (12)

$\bar{d}$ denotes average distance between opposing aryl carbon atoms connected to the *tert*-butyl groups and d_{ref} = 7.85 Å corresponds to C_{4v} *cone* conformation.
ω_1, ω_2 denotes two dihedral angles along the methylene bridge – benzyl covalent bond
N(MeCN$_{incl}$) denotes the number of different acetonitrile molecules found in calixarene *basket*

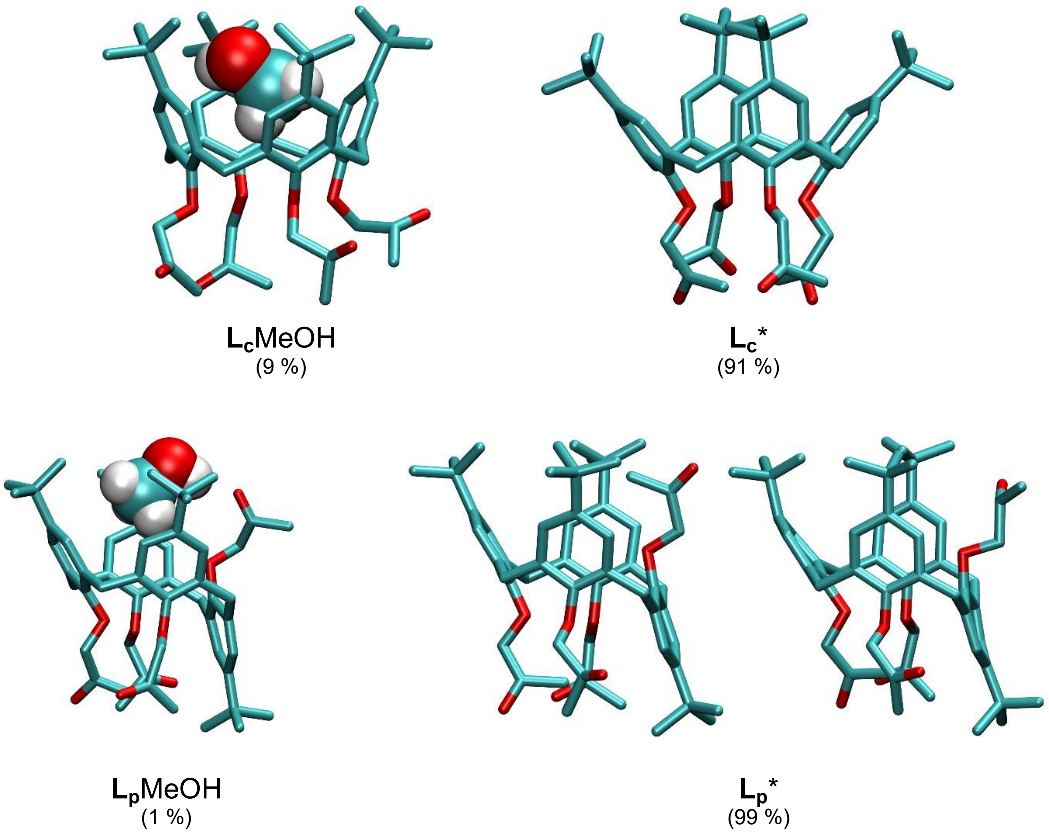

Figure S17. Representative structures of $\mathbf{L_c}$ and $\mathbf{L_p}$ and their MeOH adducts obtained by MD simulations at 25 °C. Hydrogen atoms of receptors are omitted for clarity. Numbers in parentheses represent percentages of total simulation time in which the species existed.

S2.3. MD simulations of $\mathbf{L}_c$ and $\mathbf{L}_p$ complexes

S2.3.1. Solvent: acetonitrile

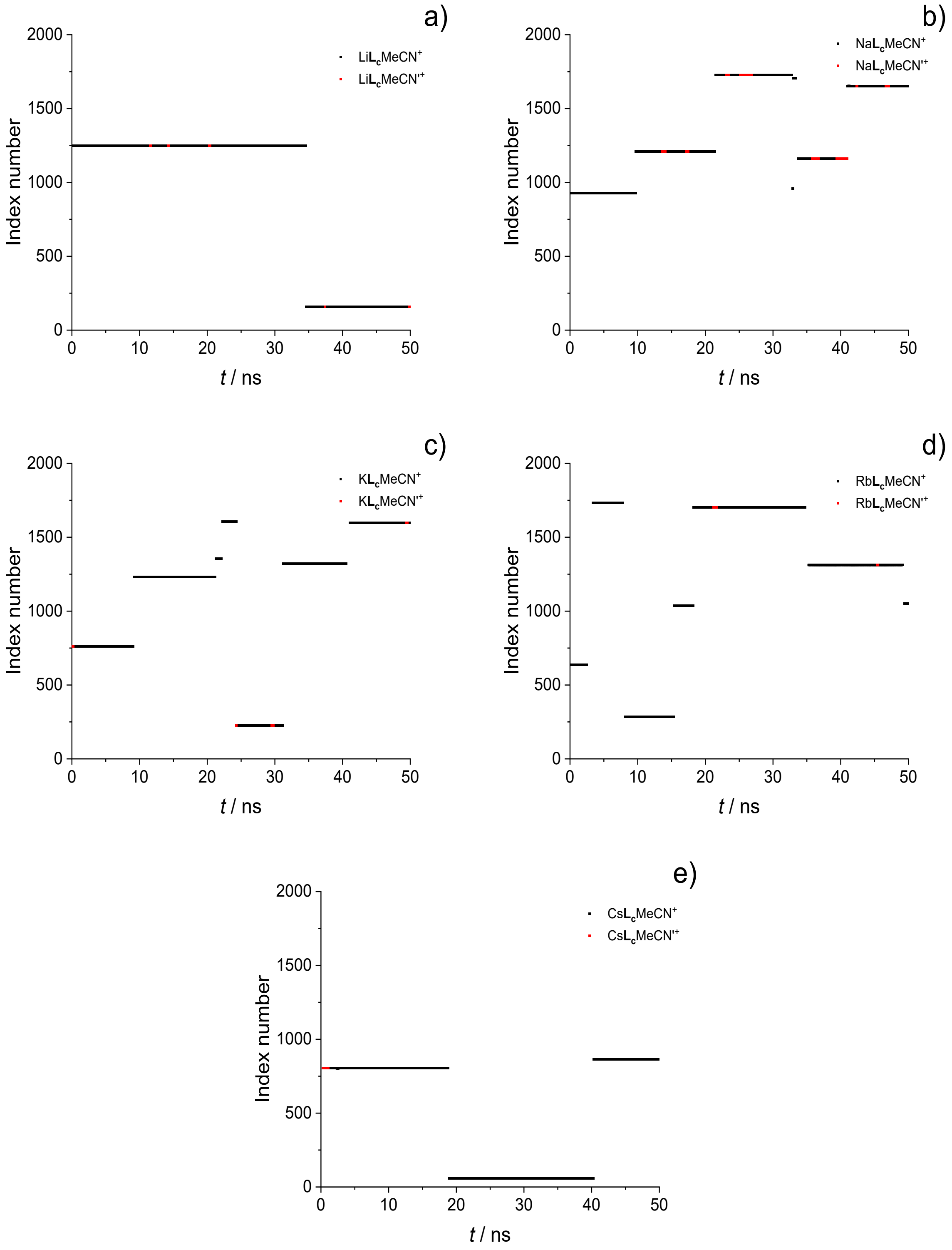

Figure S18. Index number of acetonitrile molecules that occupy hydrophobic cavities of a) Li$\mathbf{L}_c$MeCN$^+$ and Li$\mathbf{L}_c$MeCN’$^+$, b) Na$\mathbf{L}_c$MeCN$^+$ and Na$\mathbf{L}_c$MeCN’$^+$, c) K$\mathbf{L}_c$MeCN$^+$ and K$\mathbf{L}_c$MeCN’$^+$, d) Rb$\mathbf{L}_c$MeCN$^+$ and Rb$\mathbf{L}_c$MeCN’$^+$, and e) Cs$\mathbf{L}_c$MeCN$^+$ and Cs$\mathbf{L}_c$MeCN’$^+$ during MD simulations in acetonitrile at 25 °C.

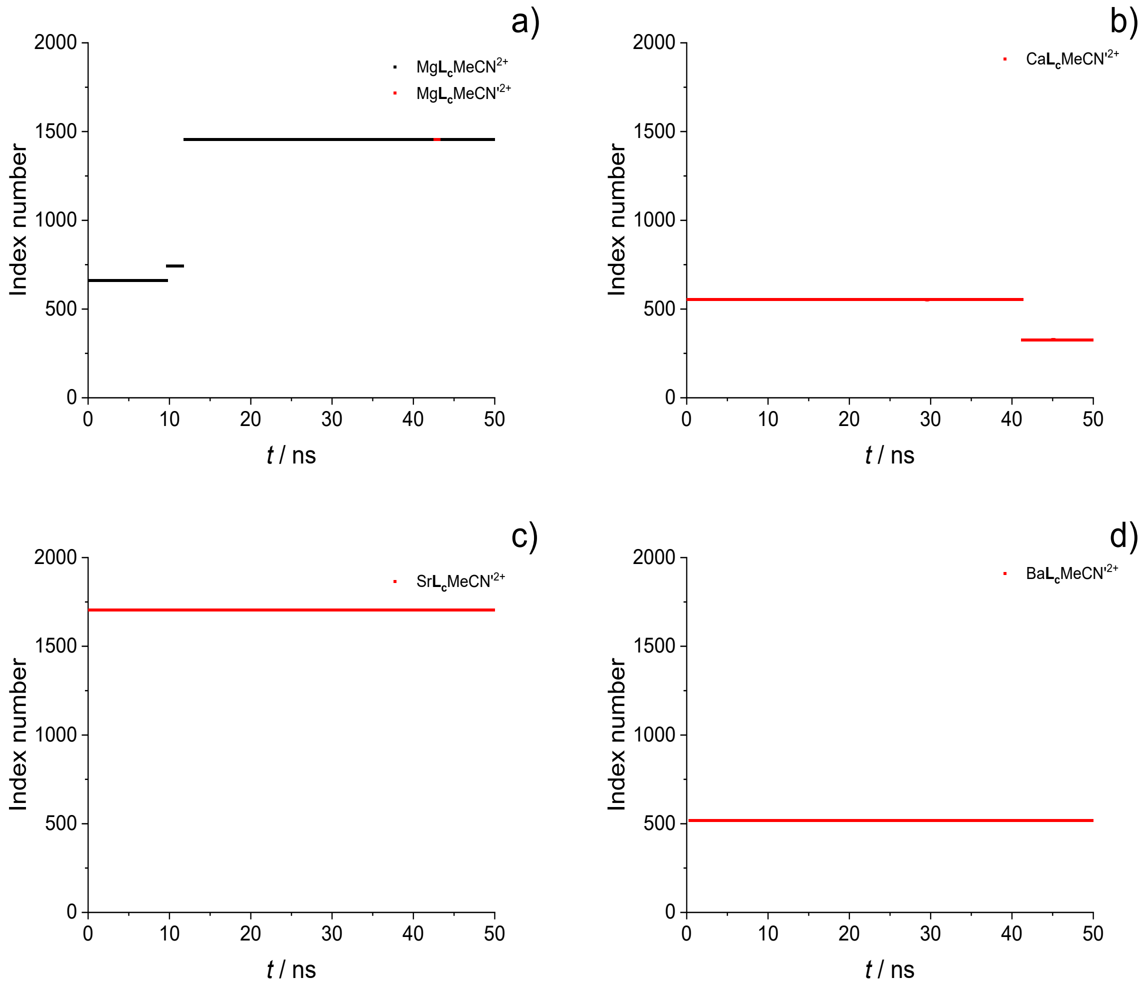

Figure S19. Index number of acetonitrile molecules that occupy hydrophobic cavities of a) Mg**L**cMeCN^{2+} and Mg**L**cMeCN'$^{2+}$, b) Ca**L**cMeCN'$^{2+}$, c) Sr**L**cMeCN'$^{2+}$, and d) Ba**L**cMeCN'$^{2+}$ during MD simulations in acetonitrile at 25 °C.

Table S5. Energies of interactions of $\mathbf{L_c}$ with alkali metal cations and acetonitrile, occurrence time ratio of different chemical species, number of ether and carbonyl groups which coordinate metal cations, and structural characteristics of calixarene *basket* in the complexes obtained by MD simulations in acetonitrile at 25 °C.

	Li^+			Na^+		
	Li**L**cMeCN+	Li**L**cMeCN'+	Li**L**c*+	Na**L**cMeCN+	Na**L**cMeCN'+	Na**L**c*+
$E(M^+–\mathbf{L_c})$ / kJ mol^{-1}	–562	–548	–551	–490	–460	–480
$E(\mathbf{L_c}$–MeCN) / kJ mol^{-1}	–498	–473	–451	–505	–481	–459
$E(\mathbf{L_c}$–MeCN$_{incl}$) / kJ mol^{-1}	–47	–26	/	–48	–28	/
$E(M^+$–MeCN) / kJ mol^{-1}	–15	–27	–18	–4	–28	–9
$E(M^+$–MeCN$_{incl}$) / kJ mol^{-1}	4	–13	/	5	–18	/
t_{total} / ns		50			50	
t / t_{total}	0.990	0.007	0.003	0.867	0.119	0.014
N(coordination, C–O–C)	1.32	1.90	1.65	2.53	3.41	2.83
N(coordination, C=O)	3.14	2.71	2.95	3.33	2.75	3.13
N(MeCN$_{incl}$)	2	2	/	7	4	/
$\bar{N}$(MeCN$_{solv}$)	0.025	0	0	0	0	0
$\bar{d}$ / Å	7.95<br>7.91	7.98<br>7.97	7.76<br>7.86	7.91<br>7.92	7.96<br>7.94	7.81<br>7.79
$\sigma(d)$ / Å	0.29<br>0.28	0.34<br>0.35	0.48<br>0.49	0.27<br>0.27	0.29<br>0.29	0.49<br>0.49
$\lvert d - d_{ref}\rvert$ / Å	0.24<br>0.23	0.29<br>0.29	0.40<br>0.38	0.22<br>0.22	0.25<br>0.24	0.39<br>0.39
ω_1 ($\sigma(\omega_1)$) / °	84 (7)<br>83 (7)<br>84 (7)<br>83 (7)	85 (7)<br>86 (7)<br>84 (8)<br>84 (8)	81 (8)<br>82 (8)<br>83 (8)<br>84 (8)	83 (7)<br>83 (7)<br>83 (7)<br>83 (7)	84 (7)<br>84 (7)<br>84 (7)<br>84 (7)	82 (7)<br>82 (7)<br>82 (8)<br>82 (7)
ω_2 ($\sigma(\omega_2)$) / °	–83 (7)<br>–84 (7)<br>–83 (7)<br>–84 (8)	–85 (7)<br>–85 (8)<br>–84 (8)<br>–85 (7)	–83 (8)<br>–84 (7)<br>–84 (8)<br>–80 (8)	–83 (7)<br>–83 (7)<br>–83 (7)<br>–83 (7)	–83 (7)<br>–84 (7)<br>–84 (7)<br>–84 (7)	–82 (7)<br>–82 (7)<br>–82 (7)<br>–83 (7)

	K^+			Rb^+			Cs^+		
	K**L**$_c$MeCN$^+$	K**L**$_c$MeCN’$^+$	K**L**$_c$*$^+$	Rb**L**$_c$MeCN$^+$	Rb**L**$_c$MeCN’$^+$	Rb**L**$_c$*$^+$	Cs**L**$_c$MeCN$^+$	Cs**L**$_c$MeCN’$^+$	Cs**L**$_c$*$^+$
E(M$^+$–**L**$_c$) / kJ mol^{-1}	–395	–389	–396	–340	–345	–342	–283	–287	–289
E(**L**$_c$–MeCN) / kJ mol^{-1}	–506	–490	–461	–495	–491	–454	–486	–481	–449
E(**L**$_c$–MeCN$_{incl}$) / kJ mol^{-1}	–48	–27	/	–47	–29	/	–46	–29	/
E(M$^+$–MeCN) / kJ mol^{-1}	–2	–17	–3	–16	–18	–13	–31	–29	–27
E(M$^+$–MeCN$_{incl}$) / kJ mol^{-1}	5	–15	/	4	–14	/	3	–12	/
t_{total} / ns		50			50			50	
t / t_{total}	0.967	0.010	0.023	0.941	0.012	0.047	0.984	0.011	0.005
N(coordination, C–O–C)	3.90	3.97	3.92	3.74	3.92	3.78	3.54	3.86	3.58
N(coordination, C=O)	3.87	3.71	3.85	3.76	3.73	3.73	3.67	3.63	3.73
N(MeCN$_{incl}$)	7	3	/	7	2	/	3	1	/
$\bar{N}$(MeCN$_{solv}$)	0.01	0	0.003	0.25	0.04	0.18	0.53	0.27	0.39
$\bar{d}$ / Å	7.86<br>7.87	7.90<br>7.88	7.67<br>7.80	7.84<br>7.84	7.85<br>7.85	7.72<br>7.67	7.82<br>7.81	7.85<br>7.81	7.78<br>7.52
σ(d) / Å	0.26<br>0.26	0.27<br>0.29	0.56<br>0.56	0.26<br>0.26	0.28<br>0.27	0.58<br>0.60	0.26<br>0.26	0.28<br>0.28	0.68<br>0.67
|d – d_{ref}| / Å	0.21<br>0.21	0.22<br>0.23	0.47<br>0.45	0.21<br>0.21	0.22<br>0.21	0.47<br>0.49	0.21<br>0.21	0.23<br>0.23	0.53<br>0.62
ω_1 (σ(ω_1)) / °	82 (7)<br>82 (7)<br>82 (7)<br>82 (7)	81 (7)<br>83 (7)<br>82 (7)<br>81 (7)	80 (8)<br>81 (8)<br>79 (8)<br>80 (7)	81 (7)<br>81 (7)<br>81 (7)<br>81 (7)	81 (7)<br>81 (7)<br>81 (7)<br>81 (7)	79 (8)<br>79 (8)<br>79 (8)<br>79 (8)	80 (7)<br>80 (7)<br>80 (7)<br>80 (7)	80 (7)<br>80 (7)<br>81 (7)<br>80 (7)	79 (7)<br>78 (8)<br>79 (9)<br>78 (8)
ω_2 (σ(ω_2)) / °	–82 (7)<br>–82 (7)<br>–82 (7)<br>–82 (7)	–82 (7)<br>–82 (7)<br>–81 (7)<br>–82 (7)	–80 (8)<br>–79 (7)<br>–81 (7)<br>–80 (8)	–81 (7)<br>–81 (7)<br>–81 (7)<br>–81 (7)	–81 (7)<br>–81 (7)<br>–81 (7)<br>–81 (7)	–79 (8)<br>–79 (8)<br>–79 (8)<br>–80 (8)	–80 (7)<br>–80 (7)<br>–80 (7)<br>–80 (7)	–80 (8)<br>–80 (7)<br>–81 (7)<br>–80 (7)	–77 (8)<br>–80 (9)<br>–77 (8)<br>–80 (8)

$\bar{d}$ denotes average distance between opposing aryl carbon atoms connected to the *tert*-butyl groups and d_{ref} = 7.85 Å corresponds to C_{4v} *cone* conformation.

ω_1, ω_2 denotes two dihedral angles along the methylene bridge – benzyl covalent bond

N(MeCN$_{incl}$) denotes the number of different acetonitrile molecules found in calixarene *basket*, whereas $\bar{N}$(MeCN$_{solv}$) denotes the average number of solvent molecules found to coordinate cation besides the included solvent molecule

Table S6. Energies of interactions of **L**$_c$ with alkaline earth metal cations and acetonitrile, occurrence time ratio of different chemical species, number of ether and carbonyl groups which coordinate metal cations, and structural characteristics of calixarene *basket* in the complexes obtained by MD simulations in acetonitrile at 25 °C.

	Mg^{2+}			Ca^{2+}		Sr^{2+}	Ba^{2+}
	Mg**L**$_c$MeCN^{2+}	Mg**L**$_c$MeCN'$^{2+}$	Mg**L**$_c$*$^{2+}$	Ca**L**$_c$MeCN'$^{2+}$	Ca**L**$_c$*$^{2+}$	Sr**L**$_c$MeCN'$^{2+}$	Ba**L**$_c$MeCN'$^{2+}$
E(M^{2+}–**L**$_c$) / kJ mol^{-1}	–1284	–1294	–1287	–989	–971	–826	–833
E(**L**$_c$–MeCN) / kJ mol^{-1}	–365	–345	–316	–390	–362	–408	–408
E(**L**$_c$–MeCN$_{incl}$) / kJ mol^{-1}	–47	–27	/	–21	/	–21	–20
E(M^{2+}–MeCN) / kJ mol^{-1}	–415	–405	–410	–253	–248	–251	–241
E(M^{2+}–MeCN$_{incl}$) / kJ mol^{-1}	3	–8	/	–34	/	–35	–36
t_{total} / ns	50			50		50	
t / t_{total}	0.980	0.010	0.010	0.998	0.002	1.000	1.000
N(coordination, C–O–C)	0	0	0	1.83	1.10	2.12	3.65
N(coordination, C=O)	4.00	4.00	4.00	3.97	3.98	3.94	4.00
N(MeCN$_{incl}$)	3	1	/	2	/	1	1
$\bar{N}$(MeCN$_{solv}$)	1.00	1.00	1.00	0.78	0.98	0.59	1.11
$\bar{d}$ / Å	7.89<br>7.88	7.92<br>7.93	7.74<br>7.77	7.92<br>7.93	8.01<br>7.51	7.86<br>7.85	7.86<br>7.86
σ(d) / Å	0.25<br>0.25	0.29<br>0.29	0.44<br>0.46	0.26<br>0.26	0.34<br>0.47	0.25<br>0.26	0.26<br>0.25
|d – d_{ref}| / Å	0.20<br>0.20	0.24<br>0.24	0.36<br>0.36	0.21<br>0.22	0.30<br>0.43	0.20<br>0.20	0.20<br>0.20
ω_1 (σ(ω_1)) / °	82 (7)<br>82 (7)<br>82 (7)<br>82 (7)	83 (7)<br>83 (7)<br>83 (8)<br>84 (7)	80 (7)<br>81 (7)<br>80 (7)<br>81 (7)	84 (7)<br>84 (7)<br>84 (7)<br>84 (7)	83 (6)<br>80 (7)<br>85 (6)<br>79 (7)	81 (7)<br>82 (7)<br>81 (7)<br>81 (7)	81 (7)<br>81 (7)<br>82 (7)<br>82 (7)
ω_2 (σ(ω_2)) / °	–82 (7)<br>–82 (7)<br>–82 (7)<br>–82 (7)	–83 (8)<br>–83 (7)<br>–84 (7)<br>–83 (7)	–81 (7)<br>–81 (7)<br>–81 (7)<br>–81 (7)	–84 (7)<br>–84 (7)<br>–84 (7)<br>–84 (7)	–82 (7)<br>–82 (6)<br>–82 (6)<br>–82 (6)	–81 (7)<br>–82 (7)<br>–81 (7)<br>–82 (7)	–81 (7)<br>–82 (7)<br>–81 (7)<br>–81 (7)

$\bar{d}$ denotes average distance between opposing aryl carbon atoms connected to the *tert*-butyl groups and d_{ref} = 7.85 Å corresponds to C_{4v} *cone* conformation.
ω_1, ω_2 denotes two dihedral angles along the methylene bridge – benzyl covalent bond
N(MeCN$_{incl}$) denotes the number of different acetonitrile molecules found in calixarene *basket*, whereas $\bar{N}$(MeCN$_{solv}$) denotes the average number of solvent molecules found to coordinate cation besides the included solvent molecule

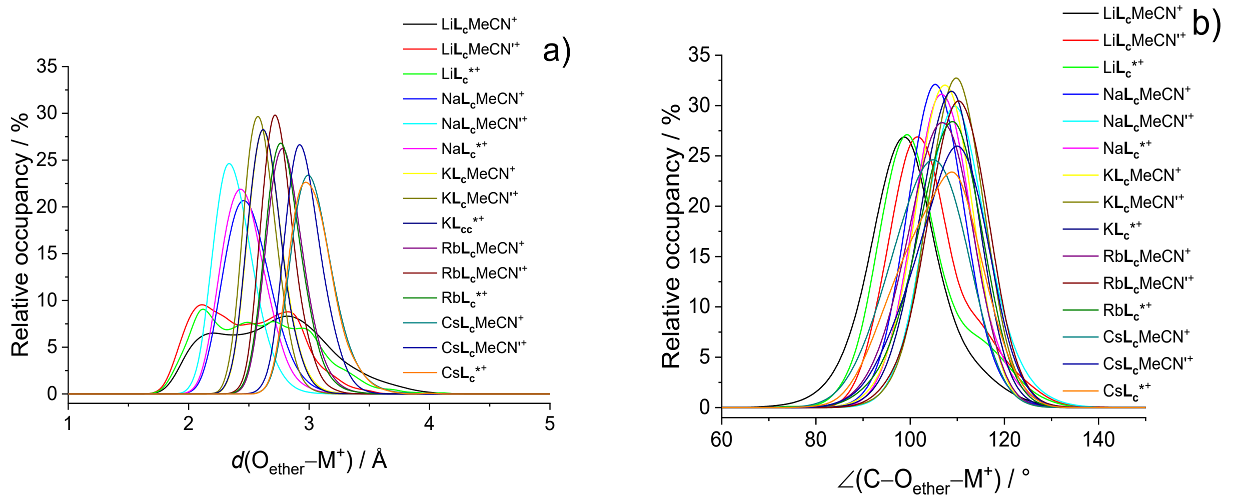

Figure S20. Distribution of a) metal cation–ether oxygen distances and b) metal cation–ether oxygen angles for M^{+}–$\mathbf{L_c}$ complexes in acetonitrile obtained by MD simulations. Data were binned at 0.1 Å and 5 ° interval.

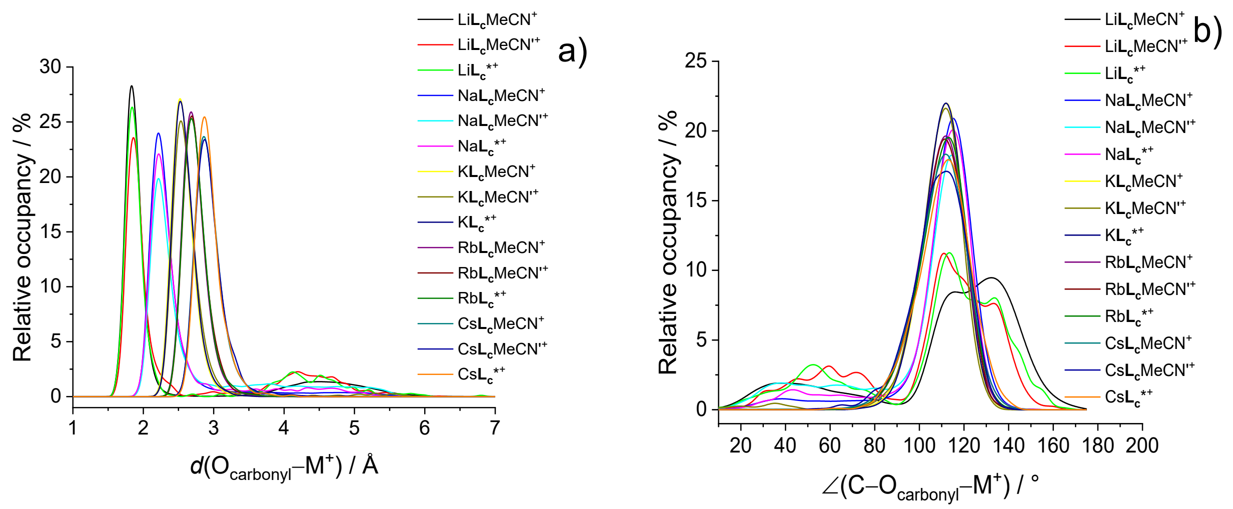

Figure S21. Distribution of a) metal cation–carbonyl oxygen distances and b) metal cation–carbonyl oxygen angles for M–$\mathbf{L_c}^{+}$ complexes in acetonitrile obtained by MD simulations. Data were binned at 0.1 Å and 5 ° interval.

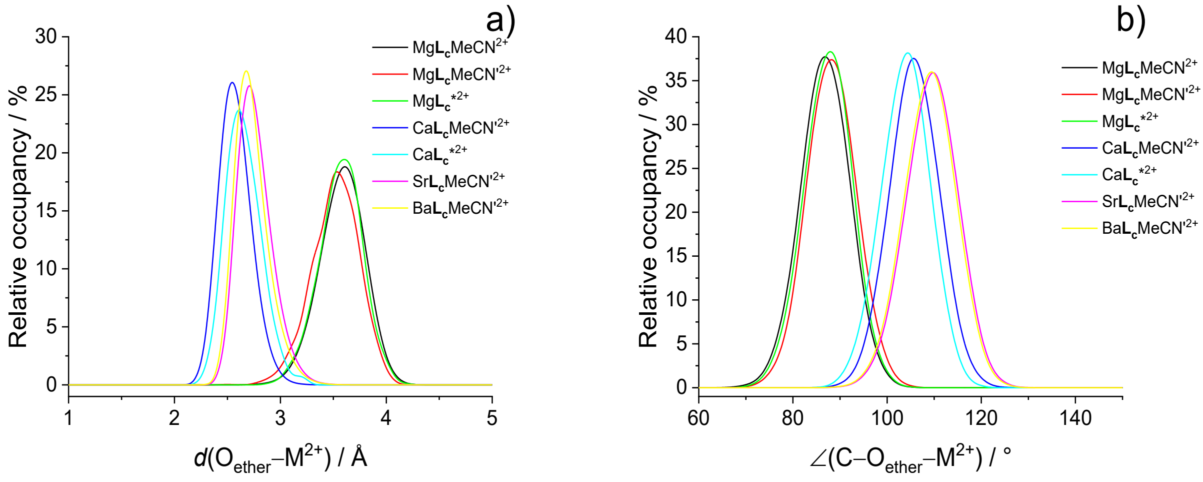

Figure S22. Distribution of a) metal cation–ether oxygen distances and b) metal cation–ether oxygen angles for M^{2+}–**L$_c$** complexes in acetonitrile obtained by MD simulations. Data were binned at 0.1 Å and 5 ° interval.

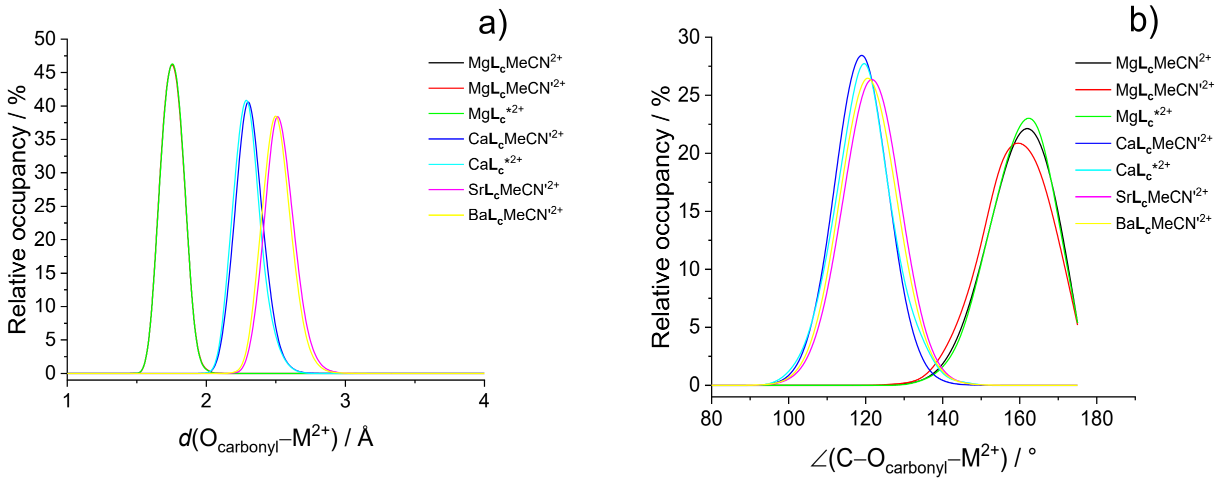

Figure S23. Distribution of a) metal cation–carbonyl oxygen distances and b) metal cation–carbonyl oxygen angles for M^{2+}–**L$_c$** complexes in acetonitrile obtained by MD simulations. Data were binned at 0.1 Å and 5 ° interval.

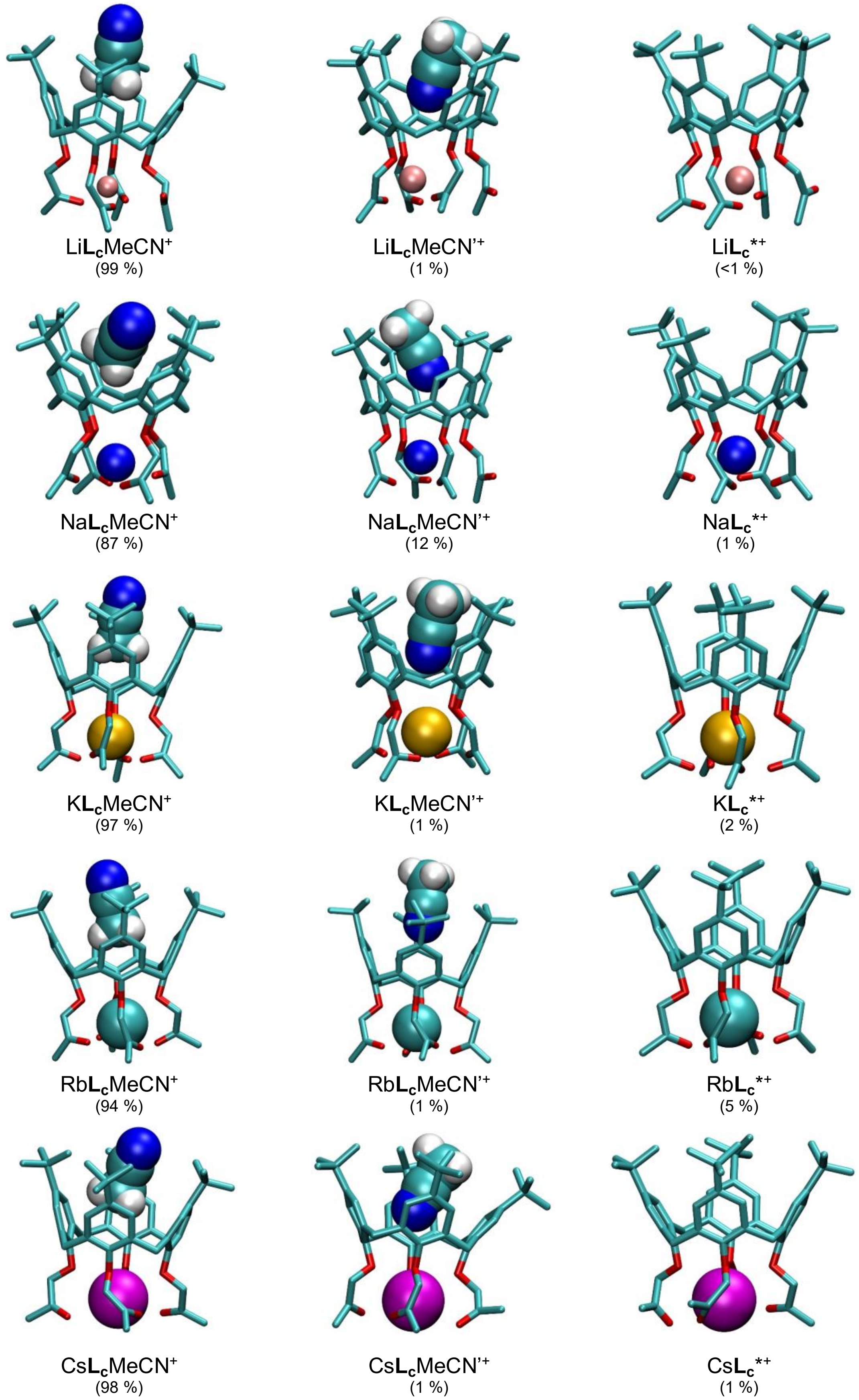

Figure S24. Representative structures of M$\mathbf{L_c}^+$ complexes and their MeCN adducts obtained by MD simulations at 25 °C. Hydrogen atoms of receptors are omitted for clarity. Numbers in parentheses represent percentages of total simulation time in which the species existed.

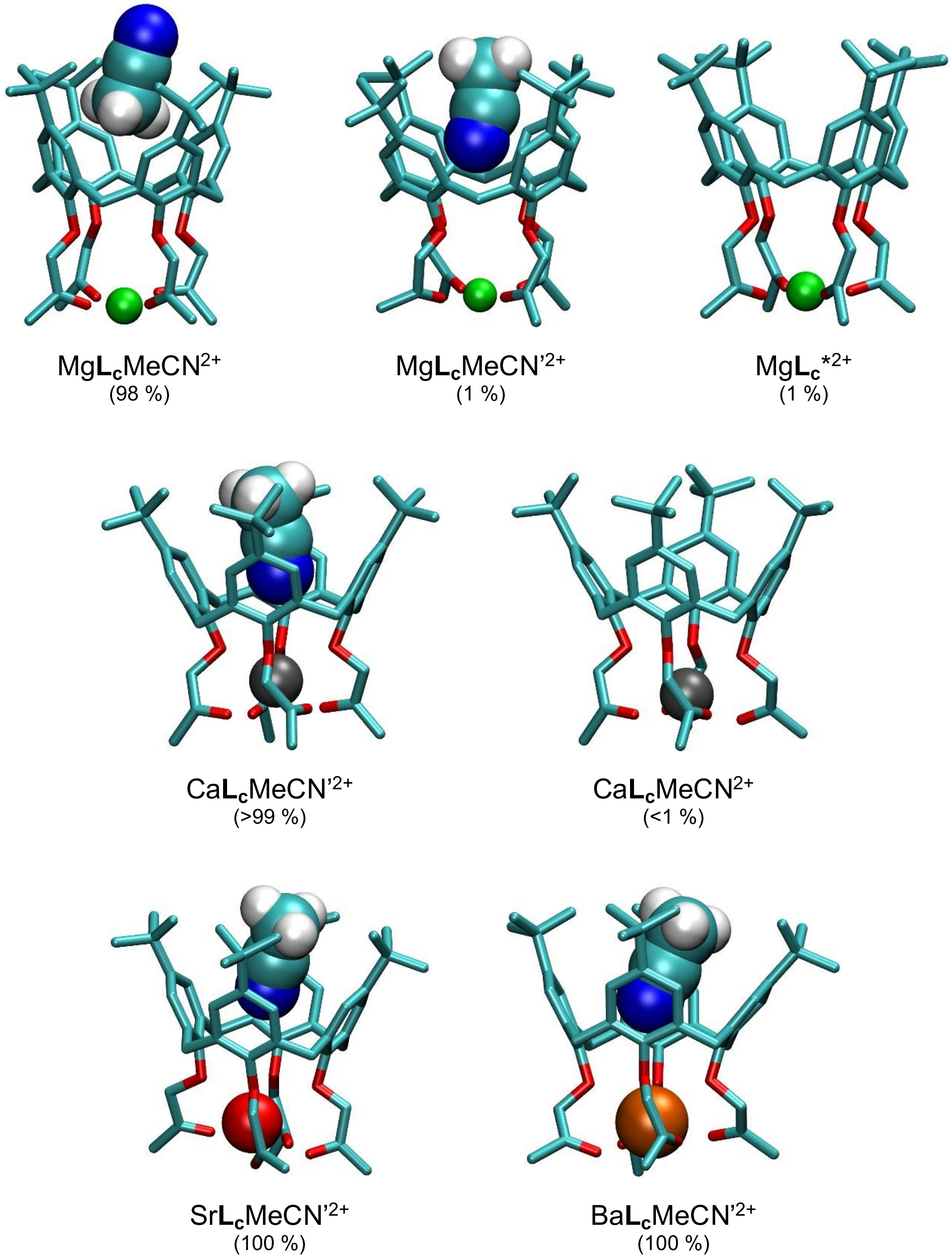

Figure S25. Representative structures of M**L**c^{2+} complexes and their MeCN adducts obtained by MD simulations at 25 °C. Hydrogen atoms of receptors are omitted for clarity. Numbers in parentheses represent percentages of total simulation time in which the species existed.

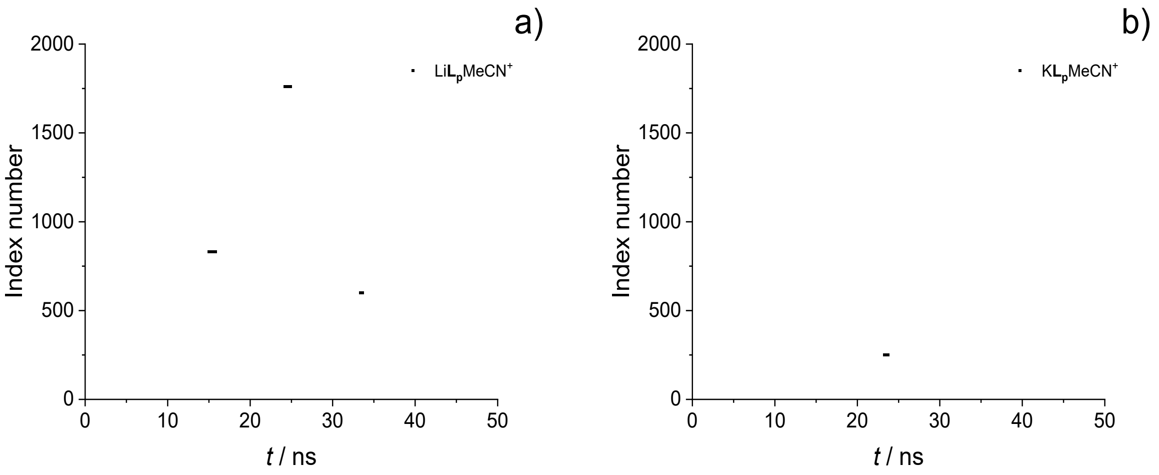

Figure S26. Index number of acetonitrile molecules that occupy hydrophobic cavities of a) Li**L**$_\mathbf{p}$MeCN$^+$ and b) K**L**$_\mathbf{p}$MeCN$^+$ during MD simulations in acetonitrile at 25 °C.

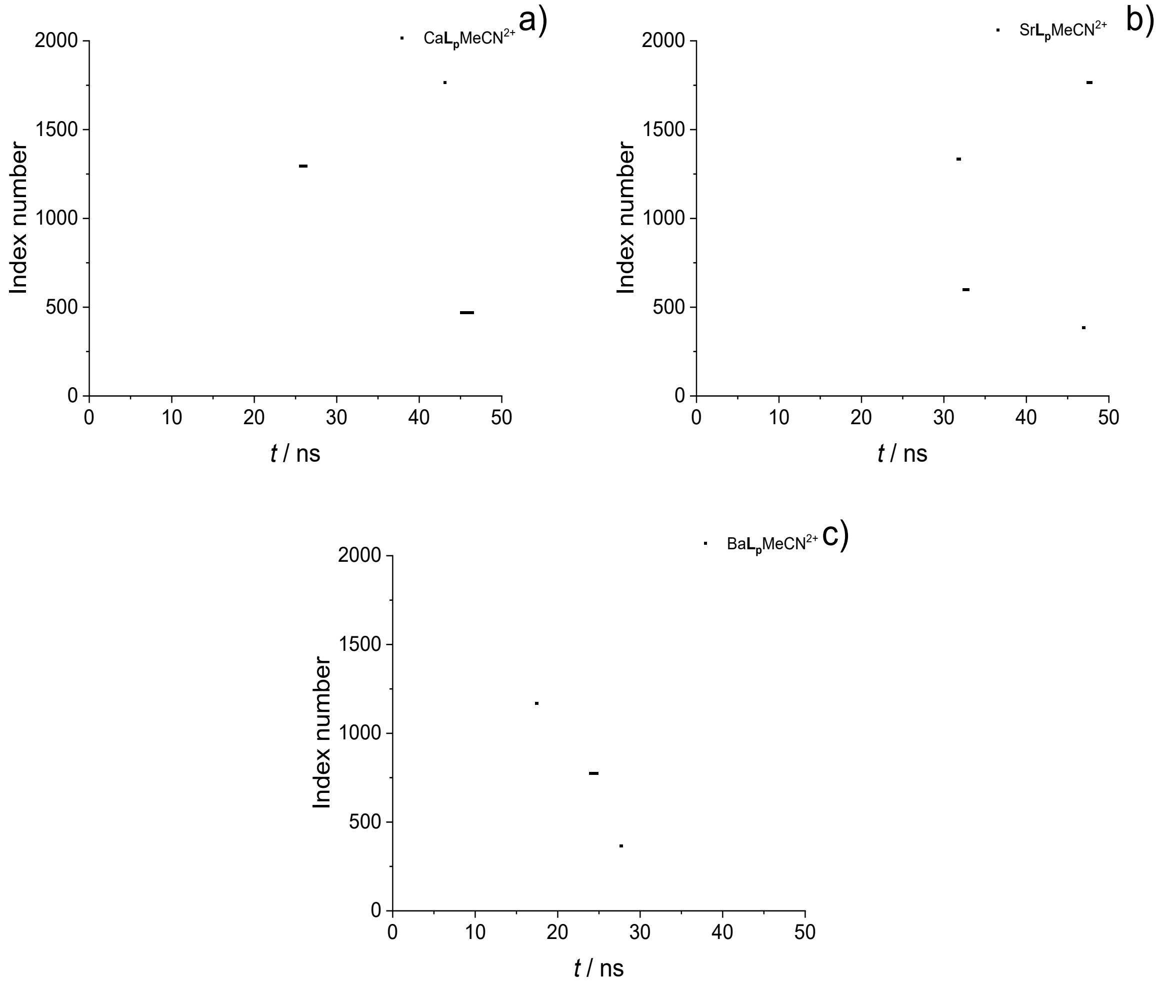

Figure S27. Index number of acetonitrile molecules that occupy hydrophobic cavities of a) Ca**L**$_\mathbf{p}$MeCN^{2+}, b) Sr**L**$_\mathbf{p}$MeCN^{2+}, and c) Ba**L**$_\mathbf{p}$MeCN^{2+} during MD simulations in acetonitrile at 25 °C.

Table S7. Energies of interactions of $\mathbf{L_p}$ with alkali metal cations and acetonitrile, occurrence time ratio of different chemical species, number of ether and carbonyl groups which coordinate metal cations, and structural characteristics of calixarene *basket* in the complexes obtained by MD simulations in acetonitrile at 25 °C.

	Li^+		Na^+	K^+		Rb^+	Cs^+
	Li**L**$_\mathbf{p}$MeCN$^+$	Li**L**$_\mathbf{p}$*$^+$	Na**L**$_\mathbf{p}$*$^+$	K**L**$_\mathbf{p}$MeCN$^+$	K**L**$_\mathbf{p}$*$^+$	Rb**L**$_\mathbf{p}$*$^+$	Cs**L**$_\mathbf{p}$*$^+$
E(M^+–$\mathbf{L_p}$) / kJ mol^{-1}	–519	–522	–422	–321	–322	–276	–230
E($\mathbf{L_p}$–MeCN) / kJ mol^{-1}	–502	–456	–466	–508	–464	–447	–439
E($\mathbf{L_p}$–$MeCN_{incl}$) / kJ mol^{-1}	–45	/	/	–44	/	/	/
E(M^+–MeCN) / kJ mol^{-1}	–20	–21	–18	–16	–18	–38	–55
E(M^+–$MeCN_{incl}$) / kJ mol^{-1}	3	/	/	3	/	/	/
t_{total} / ns	50		50	50		50	50
t / t_{total}	0.034	0.966	1.000	0.008	0.992	1.000	1.000
N(coordination, C–O–C)	1.22	1.23	2.52	2.85	2.90	2.65	2.60
N(coordination, C=O)	2.99	2.98	2.97	2.98	2.97	2.90	2.86
N($MeCN_{incl}$)	3	/	/	1	/	/	/
$\bar{N}$($MeCN_{solv}$)	0.005	0	0	0.03	0.05	0.42	0.77
$\bar{d}$ / Å	7.67	7.82	7.72	7.48	7.54	7.47	7.44
σ(d) / Å	0.26	0.29	0.27	0.25	0.31	0.33	0.31
$\|d - d_{ref}\|$ / Å	0.26	0.23	0.24	0.38	0.35	0.41	0.42
ω_1 (σ(ω_1)) / °	85 (7)	85 (7)	83 (6)	80 (7)	80 (7)	79 (7)	79 (7)
	85 (7)	81 (7)	82 (7)	84 (7)	82 (7)	81 (7)	81 (7)
	137 (11)	137 (10)	139 (9)	133 (9)	132 (9)	131 (9)	129 (9)
	–126 (9)	–137 (10)	–124 (9)	–124 (9)	–135 (10)	–137 (10)	–140 (10)
ω_2 (σ(ω_2)) / °	–85 (7)	–81 (7)	–82 (7)	–85 (7)	–82 (7)	–81 (7)	–81 (7)
	–84 (7)	–85 (7)	–83 (6)	–80 (7)	–80 (7)	–79 (7)	–79 (7)
	126 (9)	137 (10)	139 (10)	125 (9)	135 (9)	137 (10)	140 (10)
	–138 (11)	–137 (10)	–135 (9)	–134 (8)	–132 (9)	–131 (9)	–129 (9)

$\bar{d}$ denotes average distance between opposing aryl carbon atoms connected to the *tert*-butyl groups and d_{ref} = 7.85 Å corresponds to C_{4v} *cone* conformation.

ω_1, ω_2 denotes two dihedral angles along the methylene bridge – benzyl covalent bond

N($MeCN_{incl}$) denotes the number of different acetonitrile molecules found in calixarene *basket*, whereas $\bar{N}$($MeCN_{solv}$) denotes the average number of solvent molecules found to coordinate cation besides the included solvent molecule

Table S8. Energies of interactions of $\mathbf{L_p}$ with alkaline earth metal cations and acetonitrile, occurrence time ratio of different chemical species, number of ether and carbonyl groups which coordinate metal cations, and structural characteristics of calixarene *basket* in the complexes obtained by MD simulations in acetonitrile at 25 °C.

	Mg^{2+}	Ca^{2+}		Sr^{2+}		Ba^{2+}	
	$Mg\mathbf{L_p}*^{2+}$	$Ca\mathbf{L_p}MeCN^{2+}$	$Ca\mathbf{L_p}*^{2+}$	$Sr\mathbf{L_p}MeCN^{2+}$	$Sr\mathbf{L_p}*^{2+}$	$Ba\mathbf{L_p}MeCN^{2+}$	$Ba\mathbf{L_p}*^{2+}$
$E(M^{2+}–\mathbf{L_p})$ / kJ mol^{-1}	–890	–327	–343	–273	–348	–276	–301
$E(\mathbf{L_p}$–MeCN) / kJ mol^{-1}	–276	–343	–295	–367	–320	–370	–323
$E(\mathbf{L_p}–MeCN_{incl})$ / kJ mol^{-1}	/	–42	/	–42	/	–42	/
$E(M^{2+}$–MeCN) / kJ mol^{-1}	–793	–1062	–1046	–855	–784	–852	–831
$E(M^{2+}–MeCN_{incl})$ / kJ mol^{-1}	/	0	/	0	/	0	/
t_{total} / ns	50	50		50		50	
t / t_{total}	1.000	0.041	0.959	0.022	0.978	0.017	0.983
N(coordination, C–O–C)	0	0	0	0	0	0	0
N(coordination, C=O)	3.00	1.91	1.99	1.93	2.40	2.00	2.14
$N(MeCN_{incl})$	/	3	/	4	/	3	/
$\bar{N}(MeCN_{solv})$	3.00	5.88	5.79	3.49	2.19	6.73	6.55
$\bar{d}$ / Å	7.92	7.29	6.82	7.31	7.25	7.29	7.00
$\sigma(d)$ / Å	0.29	0.27	0.87	0.29	0.90	0.26	0.90
$\|d - d_{ref}\|$ / Å	0.23	0.57	1.10	0.56	0.78	0.56	0.95
ω_1 ($\sigma(\omega_1)$) / °	85 (7)	78 (7)	74 (11)	79 (7)	79 (10)	78 (7)	76 (11)
	78 (7)	94 (9)	89 (12)	93 (10)	85 (12)	95 (9)	87 (12)
	137 (10)	131 (9)	123 (16)	131 (11)	127 (14)	131 (9)	126 (12)
	–142 (11)	–125 (8)	–134 (13)	–128 (10)	–139 (15)	–125 (9)	–136 (13)
ω_2 ($\sigma(\omega_2)$) / °	–78 (7)	–93 (9)	–89 (12)	–93 (10)	–85 (12)	–95 (8)	–88 (12)
	–85 (7)	–80 (7)	–74 (10)	–79 (8)	–78 (11)	–80 (7)	–76 (10)
	142 (10)	126 (9)	133 (14)	128 (9)	139 (16)	124 (9)	135 (14)
	–137 (9)	–130 (9)	–124 (13)	–130 (9)	–128 (16)	–130 (9)	–126 (14)

$\bar{d}$ denotes average distance between opposing aryl carbon atoms connected to the *tert*-butyl groups and d_{ref} = 7.85 Å corresponds to C_{4v} *cone* conformation.
ω_1, ω_2 denotes two dihedral angles along the methylene bridge – benzyl covalent bond
$N(MeCN_{incl})$ denotes the number of different acetonitrile molecules found in calixarene *basket*, whereas $\bar{N}(MeCN_{solv})$ denotes the average number of solvent molecules found to coordinate cation besides the included solvent molecule

Table S9. Occurrence time ratio of certain orientation of inverted ketone methyl group relative to the calixarene cavity in the $\mathbf{L_p}$ ligand and its complexes without included solvent molecule obtained my MD simulations in acetonitrile at 25 °C.

	t / t_{total}	
	–CH3-in	–CH3-out
$\mathbf{L_p}$	0.772	0.228
Li$\mathbf{L_p}^+$	0.996	0.004
Na$\mathbf{L_p}^+$	0.997	0.003
K$\mathbf{L_p}^+$	0.990	0.010
Rb$\mathbf{L_p}^+$	0.986	0.014
Cs$\mathbf{L_p}^+$	0.991	0.009
Mg$\mathbf{L_p}^{2+}$	0.998	0.002
Ca$\mathbf{L_p}^{2+}$	0.806	0.194
Sr$\mathbf{L_p}^{2+}$	0.877	0.123
Ba$\mathbf{L_p}^{2+}$	0.833	0.167

–CH3-in and –CH3-out denote orientations in which methyl group is facing towards and from calixarene cavity. Total time is in this case only that corresponding to period during which no inclusion of solvent molecule into the calixarene *basket* was observed.

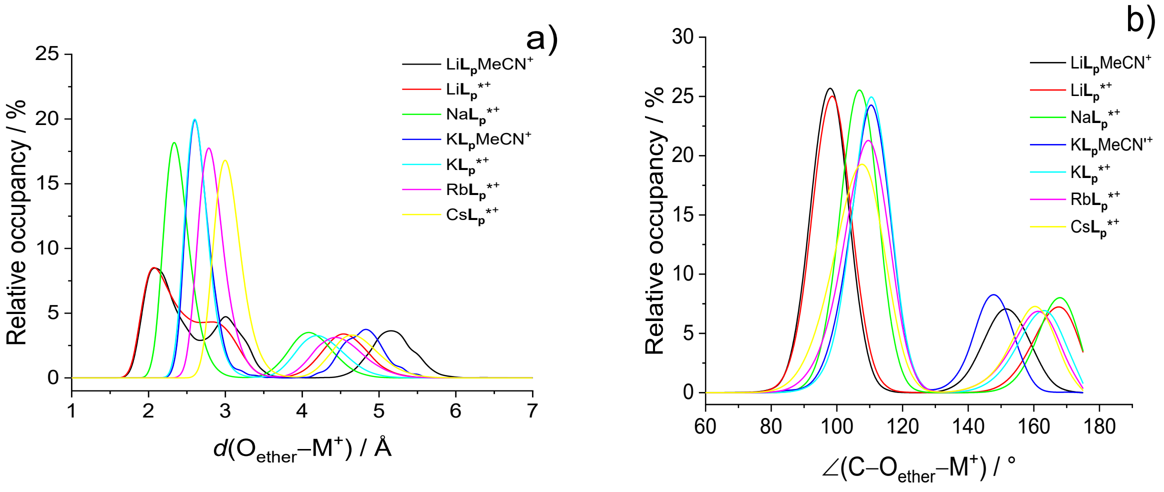

Figure S28. Distribution of a) metal cation–ether oxygen distances and b) metal cation–ether oxygen angles for M^+–$\mathbf{L_p}$ complexes in acetonitrile obtained by MD simulations. Data were binned at 0.1 Å and 5 ° interval.

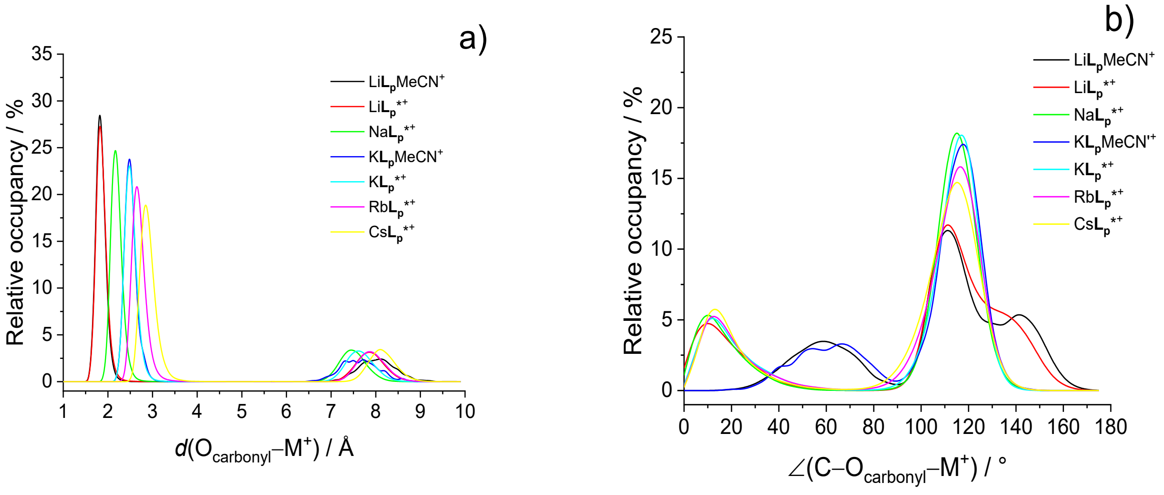

Figure SS29. Distribution of a) metal cation–carbonyl oxygen distances and b) metal cation–carbonyl oxygen angles for M^+–$\mathbf{L_p}$ complexes in acetonitrile obtained by MD simulations. Data were binned at 0.1 Å and 5 ° interval.

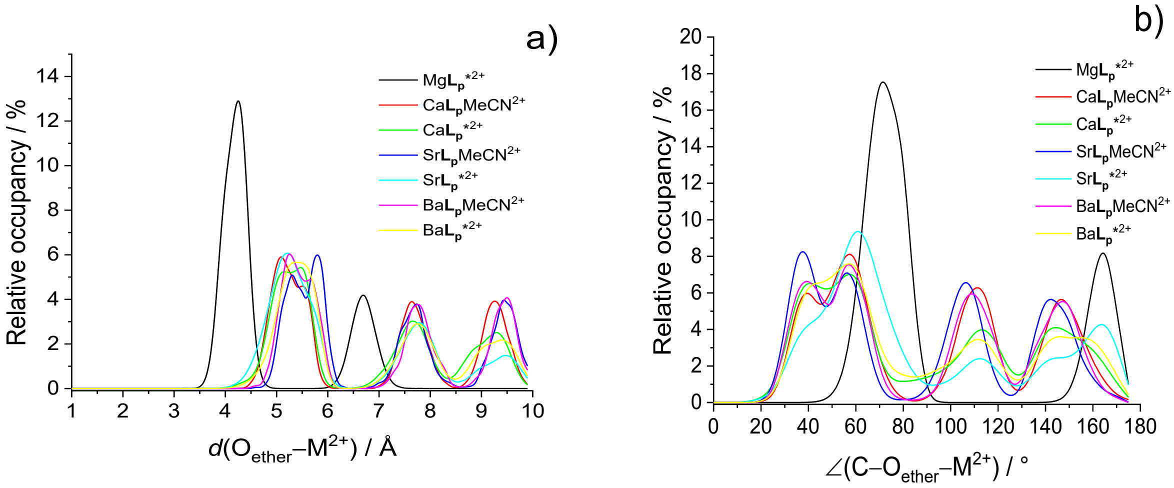

Figure S30. Distribution of a) metal cation–ether oxygen distances and b) metal cation–ether oxygen angles for M^{2+}–$\mathbf{L_p}$ complexes in acetonitrile obtained by MD simulations. Data were binned at 0.1 Å and 5 ° interval.

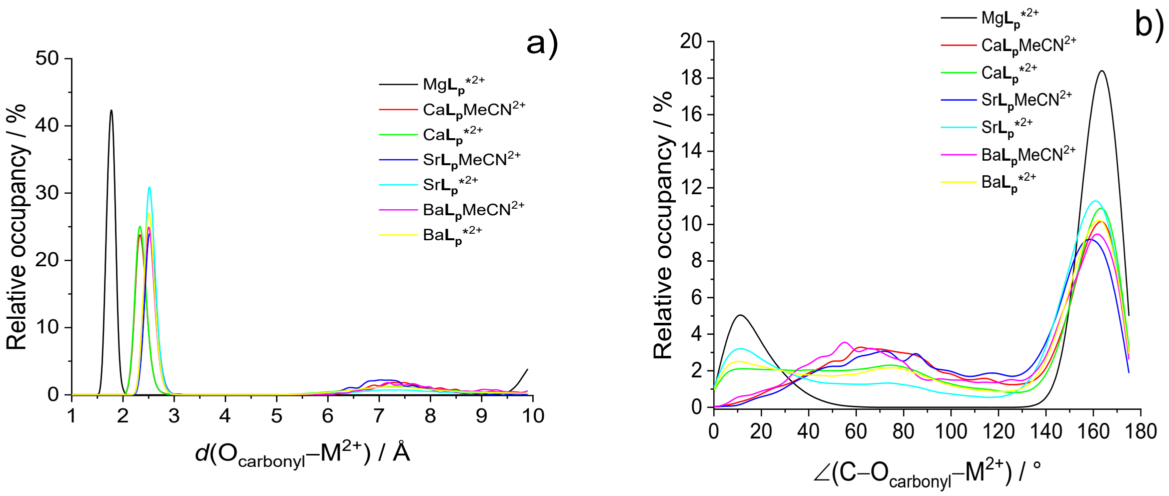

Figure S31. Distribution of a) metal cation–carbonyl oxygen distances and b) metal cation–carbonyl oxygen angles for M^{2+}–$\mathbf{L_p}$ complexes in acetonitrile obtained by MD simulations. Data were binned at 0.1 Å and 5 ° interval.

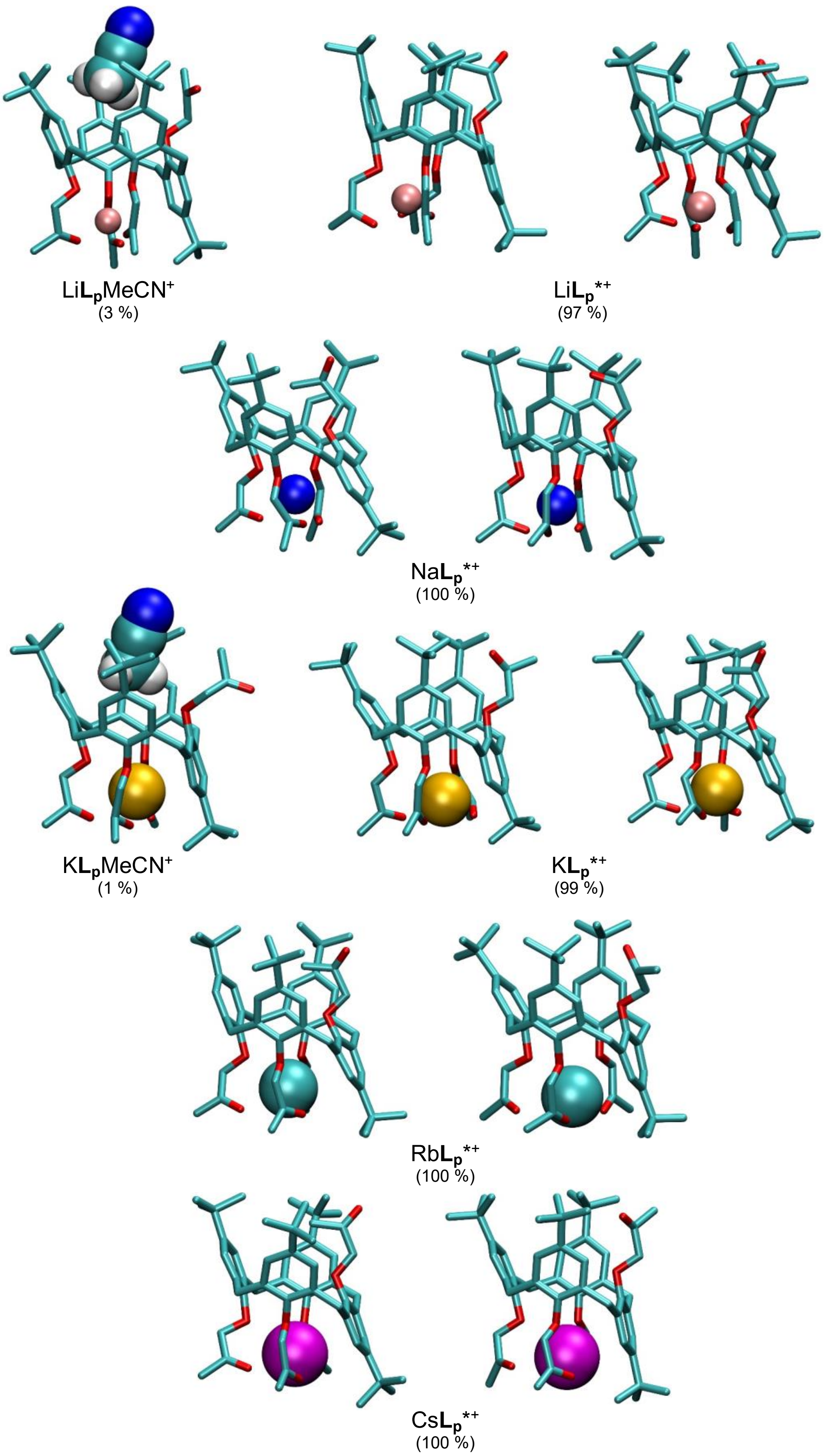

Figure S32. Representative structures of M$\mathbf{L_p}^+$ complexes and their MeCN adducts obtained by MD simulations at 25 °C. Hydrogen atoms of receptors are omitted for clarity. Numbers in parentheses represent percentages of total simulation time in which the species existed.

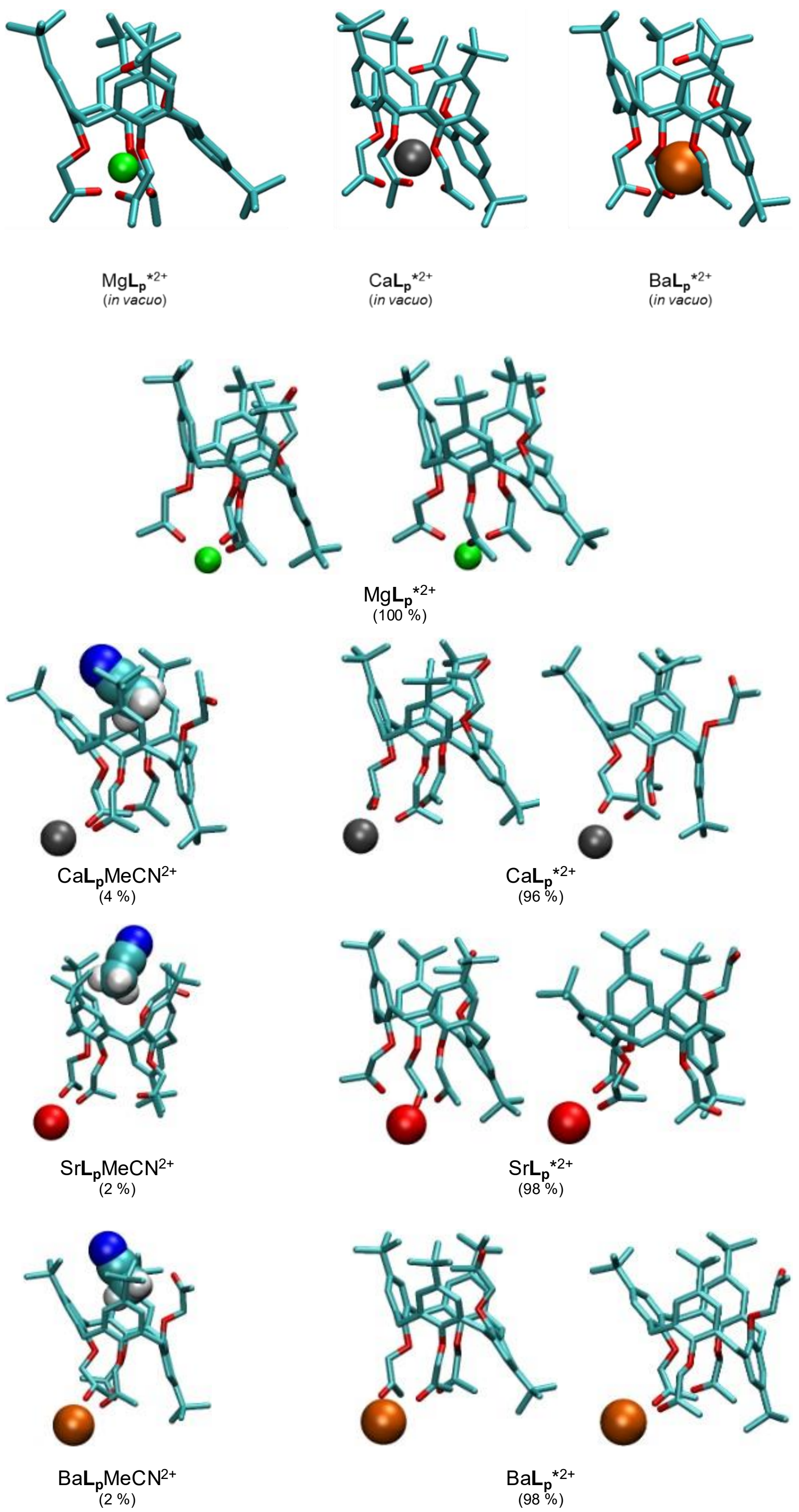

Figure S33. Representative structures of M**L$_\mathbf{p}$**$^{2+}$ complexes *in vacuo* and in acetonitrile and their MeCN adducts obtained by MD simulations at 25 °C. Hydrogen atoms of receptors are omitted for clarity. Numbers in parentheses represent percentages of total simulation time in which the species existed.

S2.3.2. Solvent: methanol

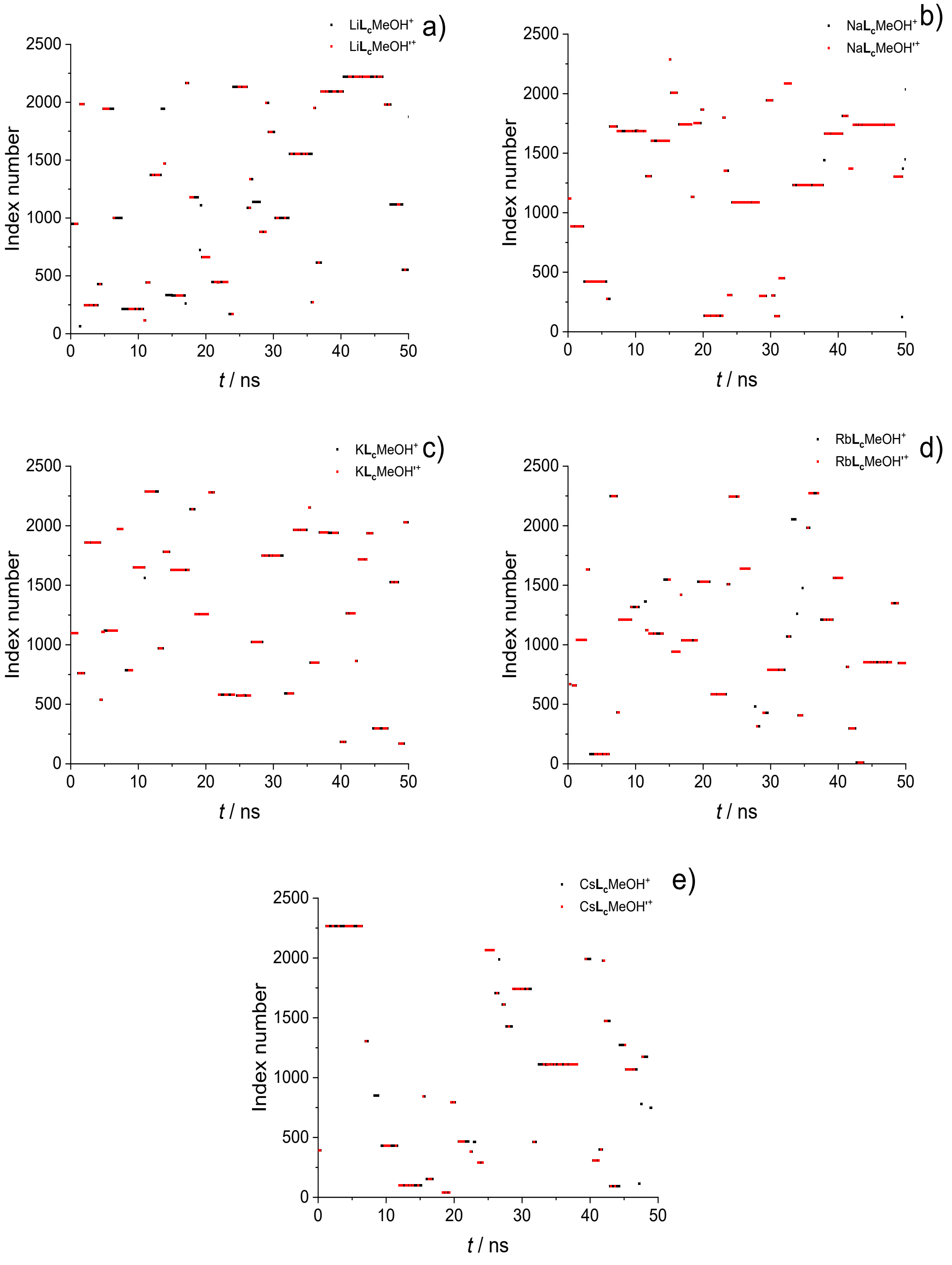

Figure S34. Index number of methanol molecules that occupy hydrophobic cavities of a) Li**L**$_c$MeOH$^+$ and Li**L**$_c$MeOH'$^+$, b) Na**L**$_c$MeOH$^+$ and Na**L**$_c$MeOH'$^+$, c) K**L**$_c$MeOH$^+$ and K**L**$_c$MeOH'$^+$, d) Rb**L**$_c$MeOH$^+$ and Rb**L**$_c$MeOH'$^+$, and e) Cs**L**$_c$MeOH$^+$ and Cs**L**$_c$MeOH'$^+$ during MD simulations in methanol at 25 °C.

Table S10. Energies of interactions of **L**$_c$ with alkali metal cations and methanol, occurrence time ratio of different chemical species, number of ether and carbonyl groups which coordinate metal cations, and structural characteristics of calixarene *basket* in the complexes obtained by MD simulations in methanol at 25 °C.

	Li^+			Na^+		
	Li**L**$_c$MeOH$^+$	Li**L**$_c$MeOH'$^+$	Li**L**$_c$*$^+$	Na**L**$_c$MeOH$^+$	Na**L**$_c$MeOH'$^+$	Na**L**$_c$*$^+$
E(M$^+$–**L**$_c$) / kJ mol^{-1}	–552	–548	–543	–478	–464	–474
E(**L**$_c$–MeOH) / kJ mol^{-1}	–448	–443	–415	–464	–459	–433
E(**L**$_c$–MeOH$_{incl}$) / kJ mol^{-1}	–46	–36	/	–44	–34	/
E(M$^+$–MeOH) / kJ mol^{-1}	–10	–7	–5	11	–1	7
E(M$^+$–MeOH$_{incl}$) / kJ mol^{-1}	4	–2	/	4	–8	/
t_{total} / ns			50		50	
t / t_{total}	0.849	0.016	0.135	0.828	0.075	0.097
N(coordination, C–O–C)	0.92	1.15	1.13	2.85	3.30	2.93
N(coordination, C=O)	3.09	2.91	2.98	3.13	2.87	3.06
N(MeOH$_{incl}$)	39	33	/	36	32	/
$\bar{N}$(MeCN$_{solv}$)	0.09	0.02	0.01	0	0	0
$\bar{d}$ / Å	7.91<br>7.96	7.94<br>7.95	7.48<br>8.11	7.92<br>7.92	7.92<br>7.91	7.84<br>7.82
σ(d) / Å	0.30<br>0.30	0.33<br>0.32	0.82<br>0.73	0.28<br>0.28	0.30<br>0.29	0.49<br>0.48
$\lvert d - d_{ref} \rvert$ / Å	0.24<br>0.25	0.27<br>0.27	0.71<br>0.63	0.23<br>0.23	0.25<br>0.24	0.38<br>0.38
ω_1 (σ(ω_1)) / °	84 (7)<br>84 (7)<br>83 (7)<br>84 (7)	84 (7)<br>84 (8)<br>84 (7)<br>84 (7)	80 (9)<br>85 (9)<br>79 (10)<br>85 (9)	83 (7)<br>83 (7)<br>83 (7)<br>84 (7)	83 (7)<br>83 (7)<br>83 (7)<br>83 (7)	82 (7)<br>82 (7)<br>82 (7)<br>83 (7)
ω_2 (σ(ω_2)) / °	–84 (7)<br>–83 (7)<br>–84 (7)<br>–84 (7)	–84 (7)<br>–84 (7)<br>–84 (7)<br>–84 (7)	–85 (9)<br>–79 (10)<br>–85 (9)<br>–81 (9)	–83 (7)<br>–83 (7)<br>–83 (7)<br>–83 (7)	–83 (7)<br>–83 (7)<br>–83 (7)<br>–83 (7)	–82 (7)<br>–82 (7)<br>–83 (8)<br>–83 (7)

	K^+			Rb^+			Cs^+		
	K$\mathbf{L}_c$MeOH$^+$	K$\mathbf{L}_c$MeOH'$^+$	K$\mathbf{L}_c$*$^+$	Rb$\mathbf{L}_c$MeOH$^+$	Rb$\mathbf{L}_c$MeOH'$^+$	Rb$\mathbf{L}_c$*$^+$	Cs$\mathbf{L}_c$MeOH$^+$	Cs$\mathbf{L}_c$MeOH'$^+$	Cs$\mathbf{L}_c$*$^+$
E(M$^+$–$\mathbf{L}_c$) / kJ mol^{-1}	–396	–395	–395	–340	–344	–342	–278	–280	–279
E($\mathbf{L}_c$–MeOH) / kJ mol^{-1}	–459	–448	–420	–453	–451	–421	–444	–437	–408
E($\mathbf{L}_c$–MeOH$_{incl}$) / kJ mol^{-1}	–44	–35	/	–46	–37	/	–44	–35	/
E(M$^+$–MeOH) / kJ mol^{-1}	15	8	13	3	5	5	–20	–18	–18
E(M$^+$–MeOH$_{incl}$) / kJ mol^{-1}	4	–6	/	4	–6	/	3	–3	/
t_{total} / ns		50			50			50	
t / t_{total}	0.791	0.049	0.160	0.759	0.043	0.198	0.688	0.019	0.293
N(coordination, C–O–C)	3.91	3.94	3.91	3.75	3.88	3.78	3.56	3.74	3.60
N(coordination, C=O)	3.84	3.77	3.82	3.71	3.70	3.68	3.52	3.46	3.48
N(MeOH$_{incl}$)	36	35	/	39	34	/	35	29	/
$\bar{N}$(MeCN$_{solv}$)	0.01	0.01	0.01	0.23	0.09	0.17	0.65	0.54	0.58
$\bar{d}$ / Å	7.86<br>7.87	7.87<br>7.85	7.80<br>7.71	7.83<br>7.84	7.84<br>7.81	7.62<br>7.78	7.81<br>7.81	7.80<br>7.80	7.72<br>7.62
σ(d) / Å	0.27<br>0.27	0.28<br>0.27	0.51<br>0.51	0.27<br>0.27	0.28<br>0.27	0.59<br>0.57	0.27<br>0.27	0.29<br>0.28	0.65<br>0.66
$\|d - d_{ref}\|$ / Å	0.22<br>0.22	0.22<br>0.22	0.40<br>0.42	0.22<br>0.22	0.22<br>0.22	0.49<br>0.45	0.22<br>0.22	0.23<br>0.22	0.52<br>0.55
ω_1 (σ(ω_1)) / °	82 (7)<br>82 (7)<br>82 (7)<br>82 (7)	82 (7)<br>82 (7)<br>82 (7)<br>81 (7)	80 (7)<br>80 (8)<br>81 (7)<br>80 (7)	81 (7)<br>81 (7)<br>81 (7)<br>81 (7)	81 (7)<br>81 (7)<br>81 (7)<br>80 (7)	79 (8)<br>80 (8)<br>79 (8)<br>80 (8)	80 (7)<br>80 (7)<br>80 (7)<br>80 (7)	80 (7)<br>80 (7)<br>80 (7)<br>80 (7)	79 (8)<br>78 (8)<br>79 (8)<br>78 (8)
ω_2 (σ(ω_2)) / °	–82 (7)<br>–82 (7)<br>–82 (7)<br>–82 (7)	–82 (7)<br>–82 (7)<br>–82 (7)<br>–82 (7)	–80 (7)<br>–81 (7)<br>–80 (7)<br>–81 (7)	–81 (7)<br>–81 (7)<br>–81 (7)<br>–81 (7)	–81 (7)<br>–81 (7)<br>–80 (7)<br>–80 (7)	–80 (8)<br>–79 (8)<br>–80 (8)<br>–79 (8)	–80 (7)<br>–80 (7)<br>–80 (7)<br>–80 (7)	–80 (7)<br>–80 (7)<br>–80 (7)<br>–80 (7)	–78 (8)<br>–79 (8)<br>–78 (8)<br>–79 (8)

$\bar{d}$ denotes average distance between opposing aryl carbon atoms connected to the *tert*-butyl groups and d_{ref} = 7.85 Å corresponds to C_{4v} *cone* conformation.

ω_1, ω_2 denotes two dihedral angles along the methylene bridge – benzyl covalent bond

N(MeCN$_{incl}$) denotes the number of different acetonitrile molecules found in calixarene *basket*, whereas $\bar{N}$(MeCN$_{solv}$) denotes the average number of solvent molecules found to coordinate cation besides the included solvent molecule

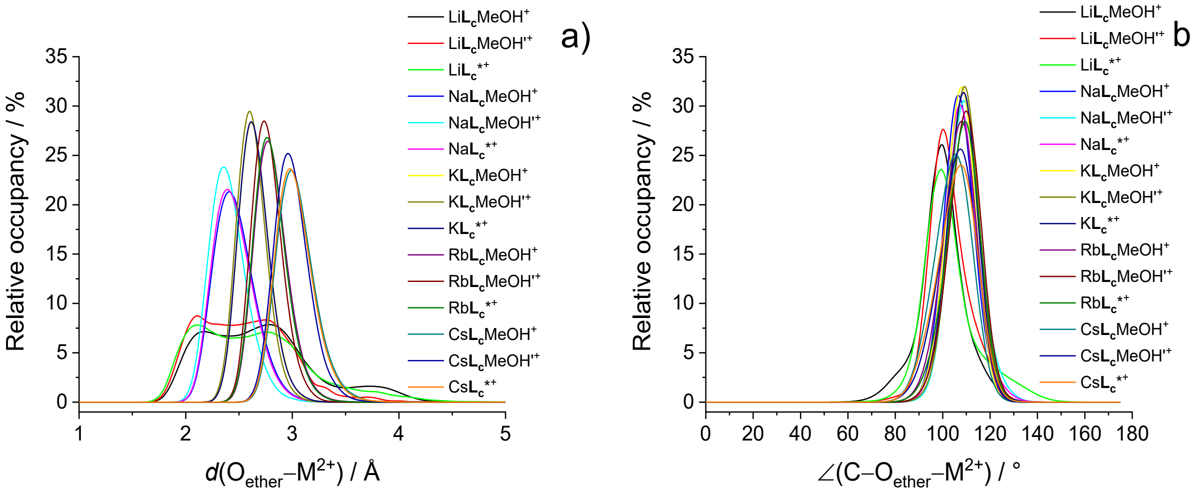

Figure S35. Distribution of a) metal cation–ether oxygen distances and b) metal cation–ether oxygen angles for M^{2+}–**L**$_c$ complexes in methanol obtained by MD simulations. Data were binned at 0.1 Å and 5 ° interval.

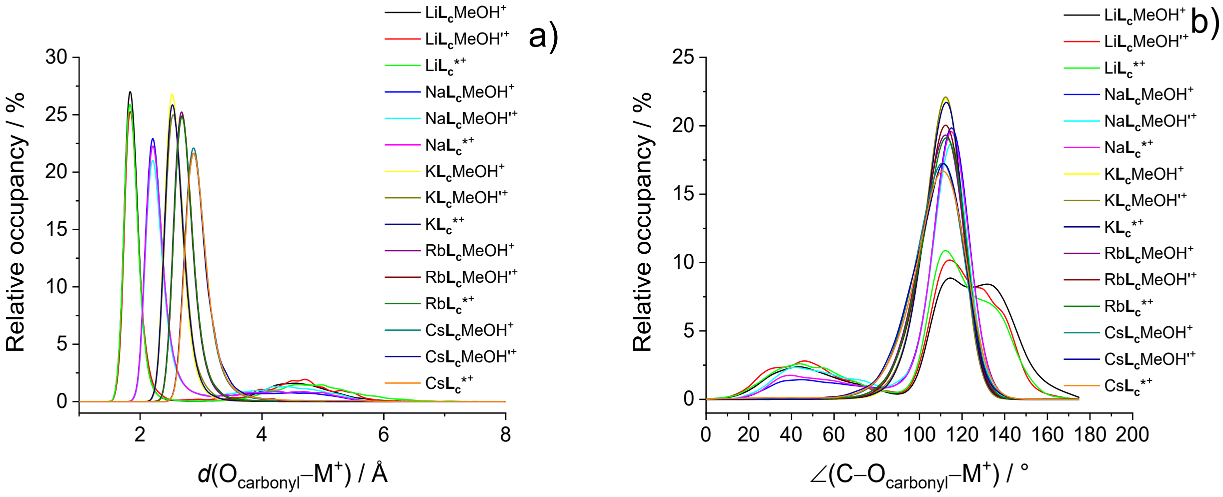

Figure S36. Distribution of a) metal cation–carbonyl oxygen distances and b) metal cation–carbonyl oxygen angles for M^{+}–**L**$_c$ complexes in methanol obtained by MD simulations. Data were binned at 0.1 Å and 5 ° interval.

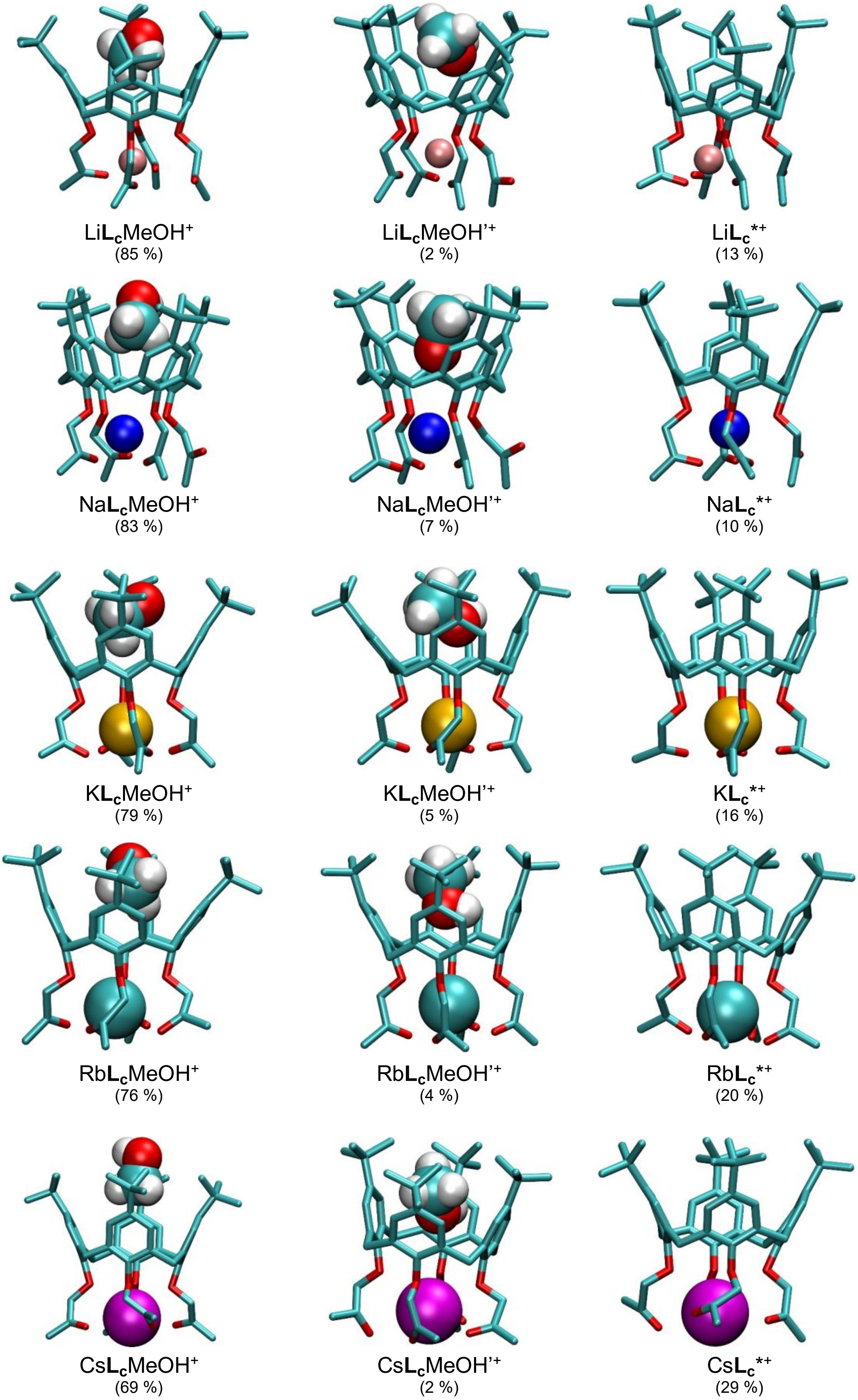

Figure S37. Representative structures of M$\mathbf{L_c}^+$ complexes and their MeOH adducts obtained by MD simulations at 25 °C. Hydrogen atoms of receptors are omitted for clarity. Numbers in parentheses represent percentages of total simulation time in which the species existed.

Table S11. Energies of interactions of $\mathbf{L_p}$ with alkali metal cations and methanol, occurrence time ratio of different chemical species, number of ether and carbonyl groups which coordinate metal cations, and structural characteristics of calixarene *basket* in the complexes obtained by MD simulations in methanol at 25 °C.

	Li^+	Na^+		K^+		Rb^+		Cs^+
	Li$\mathbf{L_p}$*$^+$	Na$\mathbf{L_p}$MeOH$^+$	Na$\mathbf{L_p}$*$^+$	K$\mathbf{L_p}$MeOH$^+$	K$\mathbf{L_p}$*$^+$	Rb$\mathbf{L_p}$MeOH$^+$	Rb$\mathbf{L_p}$*$^+$	Cs$\mathbf{L_p}$*$^+$
E(M$^+$–$\mathbf{L_p}$) / kJ mol^{-1}	–511	–415	–422	–324	–321	–265	–272	–226
E($\mathbf{L_p}$–MeOH) / kJ mol^{-1}	–405	–483	–432	–478	–427	–457	–410	–403
E($\mathbf{L_p}$–MeOH$_{incl}$) / kJ mol^{-1}	/	–47	/	–49	/	–53	/	/
E(M$^+$–MeOH) / kJ mol^{-1}	–26	1	–3	1	–6	–36	–31	–47
E(M$^+$–MeOH$_{incl}$) / kJ mol^{-1}	/	4	/	3	/	3	/	/
t_{total} / ns	50	50		50		50		50
t / t_{total}	1.000	0.002	0.998	0.005	0.995	0.003	0.997	1.000
N(coordination, C–O–C)	1.10	2.37	2.55	2.92	2.88	2.44	2.61	2.51
N(coordination, C=O)	2.98	2.95	2.96	3.00	2.96	2.81	2.85	2.78
N(MeOH$_{incl}$)	/	1	/	1	/	1	/	/
$\bar{N}$(MeCN$_{solv}$)	0.13	0	0	0	0.08	0.70	0.52	0.85
$\bar{d}$ / Å	7.81	7.58	7.72	7.46	7.55	7.44	7.48	7.44
σ(d) / Å	0.29	0.25	0.27	0.25	0.30	0.26	0.32	0.33
|d – d_{ref}| / Å	0.23	0.29	0.24	0.40	0.34	0.42	0.40	0.43
ω_1 (σ(ω_1)) / °	85 (7)<br>81 (7)<br>137 (10)<br>–137 (10)	82 (8)<br>85 (6)<br>138 (7)<br>–128 (8)	83 (6)<br>82 (7)<br>135 (9)<br>–139 (9)	81 (7)<br>84 (7)<br>135 (8)<br>–128 (8)	80 (7)<br>82 (7)<br>132 (9)<br>–136 (10)	79 (6)<br>84 (7)<br>132 (9)<br>–125 (9)	79 (7)<br>82 (7)<br>130 (9)<br>–138 (11)	79 (7)<br>81 (7)<br>129 (9)<br>–140 (10)
ω_2 (σ(ω_2)) / °	–81 (7)<br>–85 (7)<br>137 (10)<br>–137 (10)	–86 (7)<br>–83 (7)<br>127 (9)<br>–136 (8)	–82 (7)<br>–83 (6)<br>139 (9)<br>–135 (9)	–86 (8)<br>–79 (7)<br>126 (8)<br>–132 (9)	–82 (7)<br>–80 (7)<br>136 (10)<br>–132 (9)	–82 (7)<br>–80 (7)<br>127 (10)<br>–133 (11)	–82 (7)<br>–79 (7)<br>138 (11)<br>–130 (9)	–81 (7)<br>–79 (7)<br>140 (10)<br>–129 (9)

$\bar{d}$ denotes average distance between opposing aryl carbon atoms connected to the *tert*-butyl groups and d_{ref} = 7.85 Å corresponds to C_{4v} *cone* conformation.
ω_1, ω_2 denotes two dihedral angles along the methylene bridge – benzyl covalent bond
N(MeCN$_{incl}$) denotes the number of different acetonitrile molecules found in calixarene *basket*, whereas $\bar{N}$(MeCN$_{solv}$) denotes the average number of solvent molecules found to coordinate cation besides the included solvent molecule

Table S12. Occurrence time ratio of certain orientation of inverted ketone methyl group relative to the calixarene cavity in the **L**$_p$ ligand and its complexes without included solvent molecule obtained my MD simulations in methanol at 25 °C.

	t / t_{total}	
	–CH_3-in	–CH_3-out
**L**$_p$	0.721	0.279
Li**L**$_p^+$	0.997	0.003
Na**L**$_p^+$	0.997	0.003
K**L**$_p^+$	0.990	0.010
Rb**L**$_p^+$	0.984	0.016
Cs**L**$_p^+$	0.984	0.016

–CH_3-in and –CH_3-out denote orientations in which methyl group is facing towards and from calixarene cavity, respectively. Total time is in this case only that corresponding to period during which no inclusion of solvent molecule into the calixarene *basket* was observed.

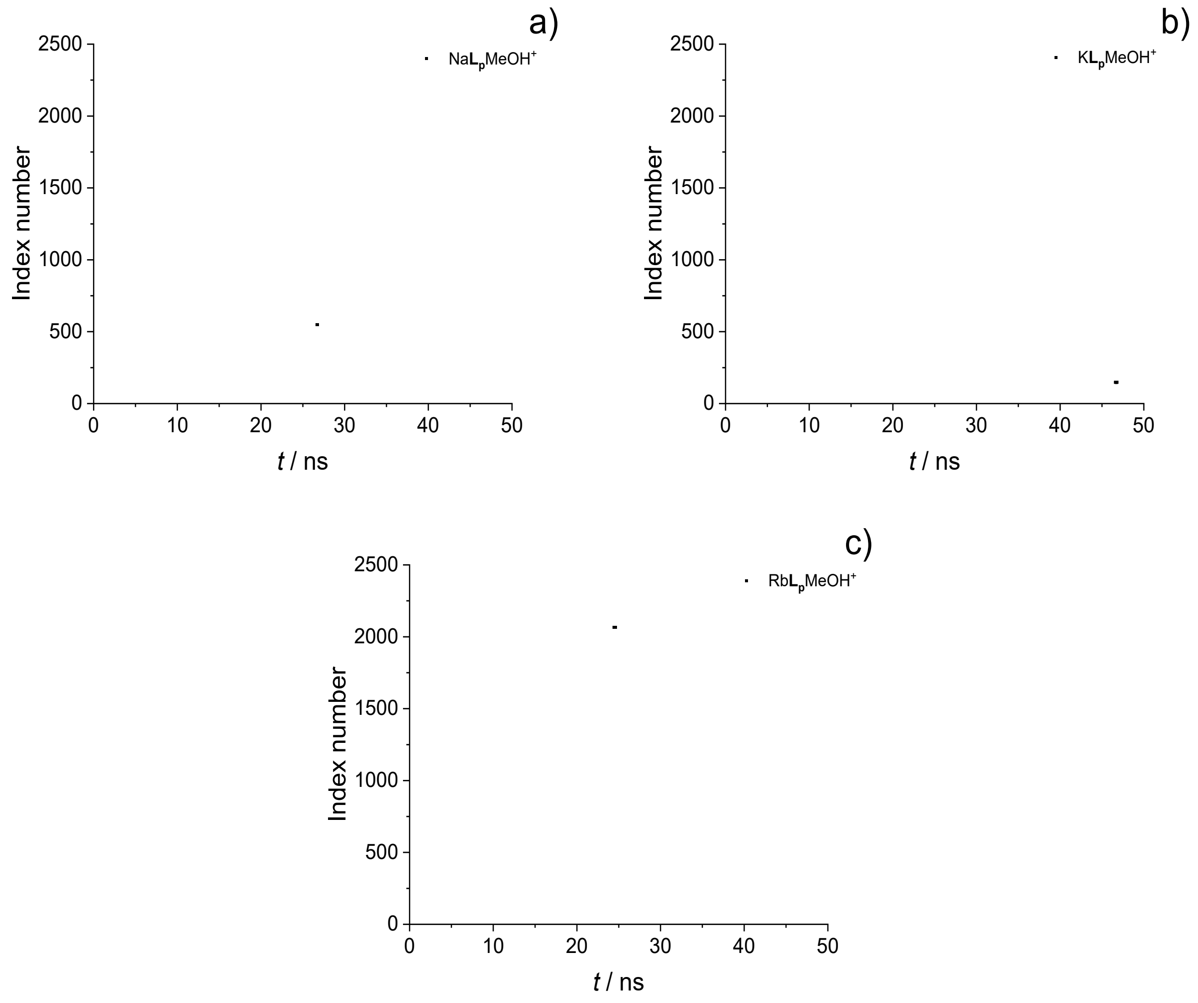

Figure S38. Index number of methanol molecules that occupy hydrophobic cavities of a) Na**L**$_p$MeOH$^+$, b) K**L**$_p$MeOH$^+$, and c) Rb**L**$_p$MeOH$^+$ during MD simulations in methanol at 25 °C.

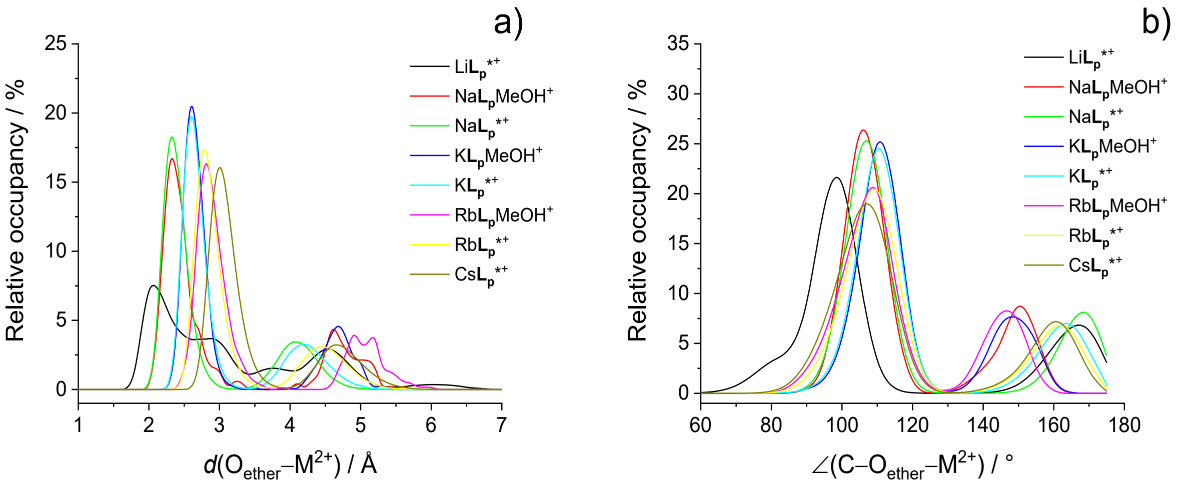

Figure S39. Distribution of a) metal cation–ether oxygen distances and b) metal cation–ether oxygen angles for M^{2+}–$\mathbf{L_p}$ complexes in methanol obtained by MD simulations. Data were binned at 0.1 Å and 5 ° interval.

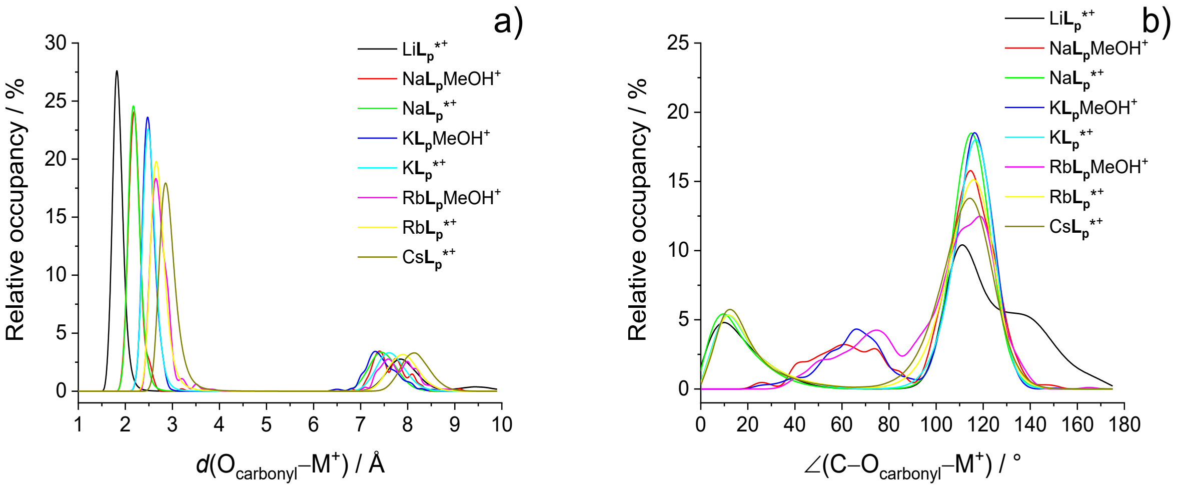

Figure S40. Distribution of a) metal cation–carbonyl oxygen distances and b) metal cation–carbonyl oxygen angles for M^{+}–$\mathbf{L_p}$ complexes in methanol obtained by MD simulations. Data were binned at 0.1 Å and 5 ° interval.

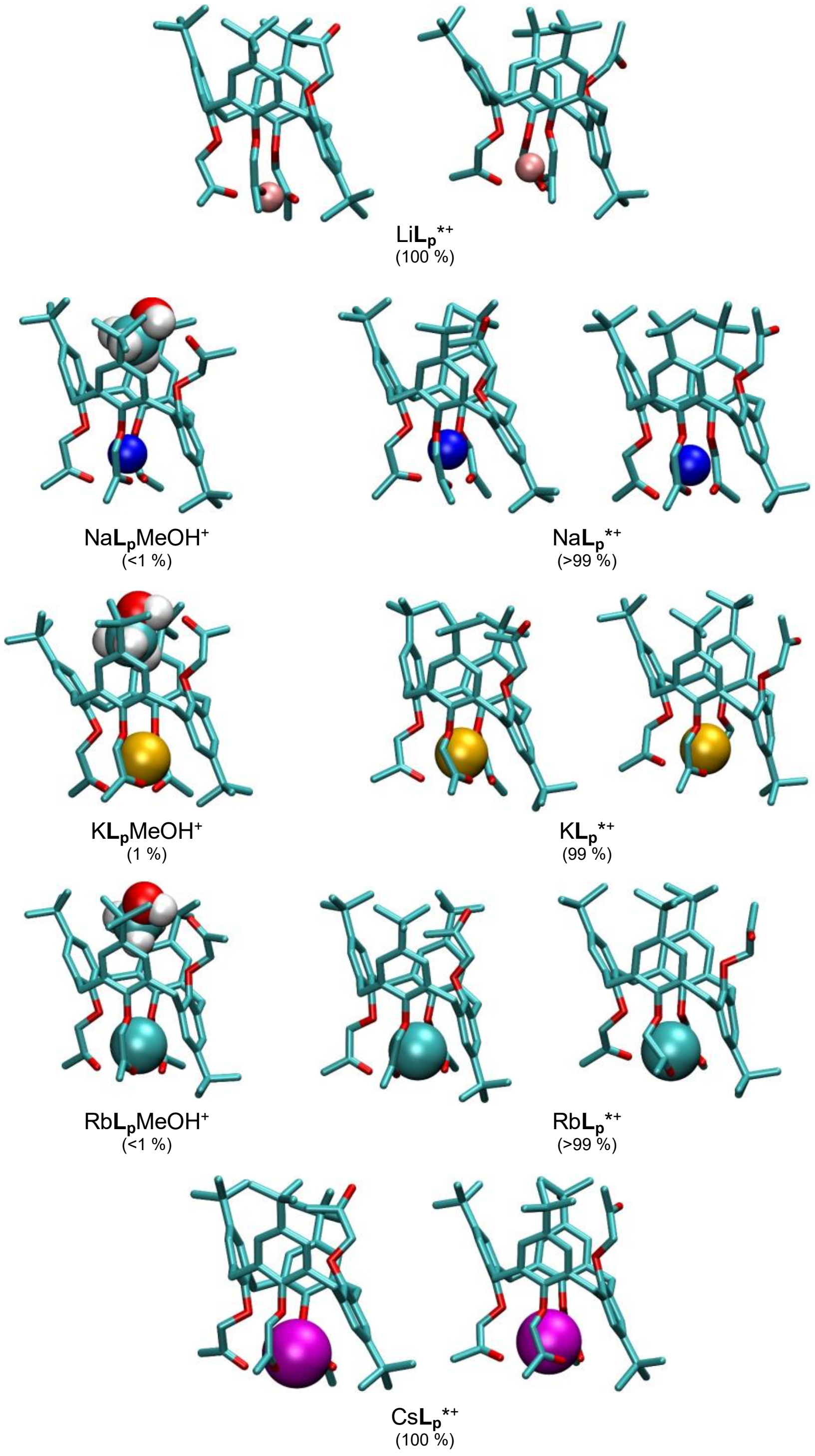

Figure S41. Representative structures of M$\mathbf{L_p}^+$ complexes and their MeOH adducts obtained by MD simulations at 25 °C. Hydrogen atoms of receptors are omitted for clarity. Numbers in parentheses represent percentages of total simulation time in which the species existed.

S2.4. Alkali metal cation complexation

S2.4.1. Solvent: acetonitrile

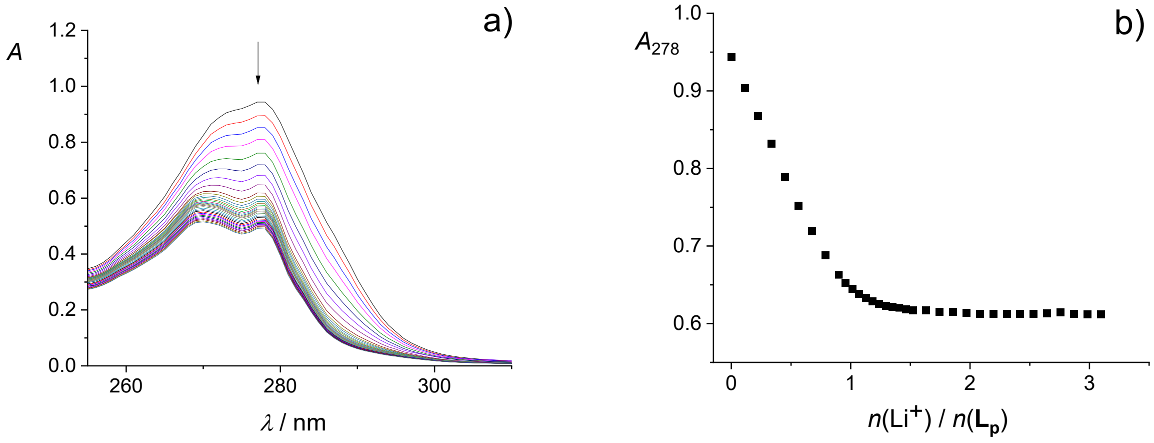

Figure S42. a) Spectrophotometric titration of $\mathbf{L_p}$ ($c = 2.46 \times 10^{-4}$ mol dm^{-3}) with $LiClO_4$ ($c = 3.05 \times 10^{-3}$ mol dm^{-3}) in MeCN at 25 °C; $V_0(\mathbf{L_p}) = 2.2$ cm^3; $l = 1$ cm. Spectra are corrected for dilution. b) Absorbance of $\mathbf{L_p}$ at 278 nm as a function of cation to ligand molar ratio.

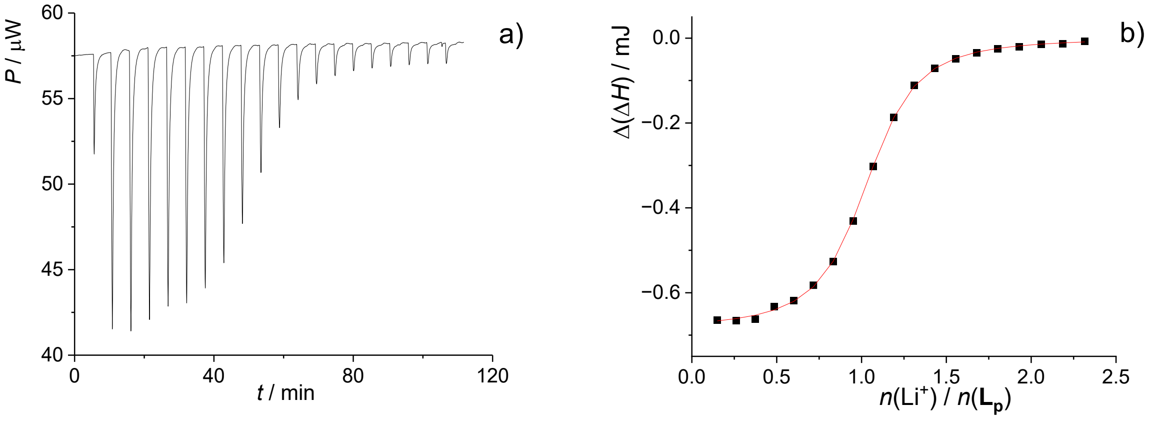

Figure S43. a) Microcalorimetric titration of $\mathbf{L_p}$ ($c = 3.00 \times 10^{-4}$ mol dm^{-3}, $V = 1.42$ cm^3) with $LiClO_4$ ($c = 2.58 \times 10^{-3}$ mol dm^{-3}) in MeCN at 25 °C. b) Dependence of successive enthalpy changes on cation to ligand molar ratio. ▪ experimental; — calculated.

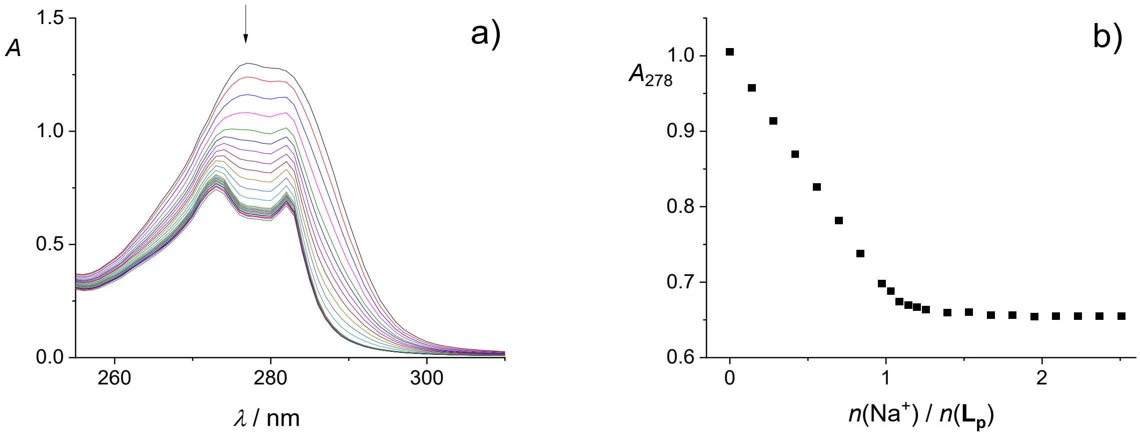

Figure S44. a) Spectrophotometric titration of $\mathbf{L_p}$ ($c = 3.11 \times 10^{-4}$ mol dm^{-3}) with $NaClO_4$ ($c = 2.48 \times 10^{-3}$ mol dm^{-3}) in MeCN at 25 °C; $V_0(\mathbf{L_p}) = 2.2$ cm^3; $l = 1$ cm. Spectra are corrected for dilution. b) Absorbance of $\mathbf{L_p}$ at 278 nm as a function of cation to ligand molar ratio.

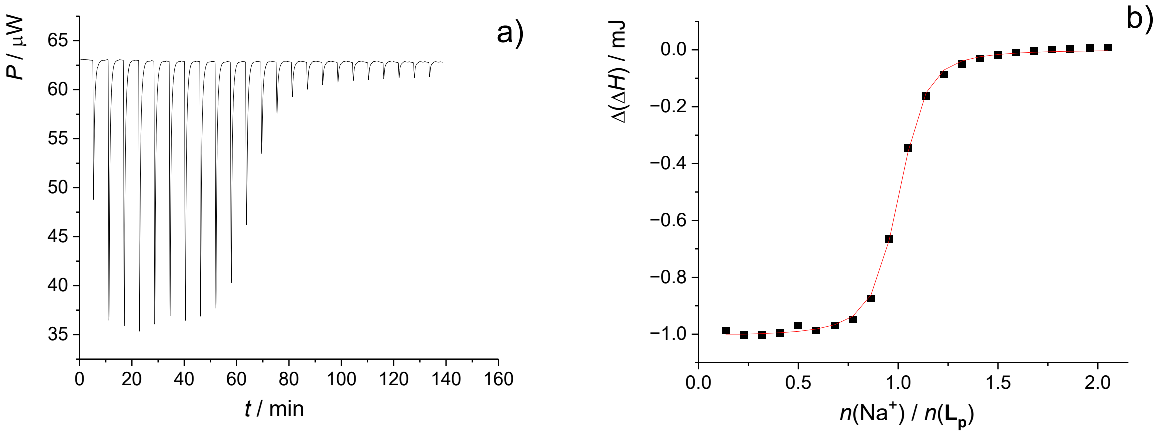

Figure S45. a) Microcalorimetric titration of $\mathbf{L_p}$ ($c = 2.37 \times 10^{-4}$ mol dm^{-3}, $V = 1.42$ cm^3) with $NaClO_4$ ($c = 3.05 \times 10^{-3}$ mol dm^{-3}) in MeCN at 25 °C. b) Dependence of successive enthalpy changes on cation to ligand molar ratio. ▪ experimental; — calculated.

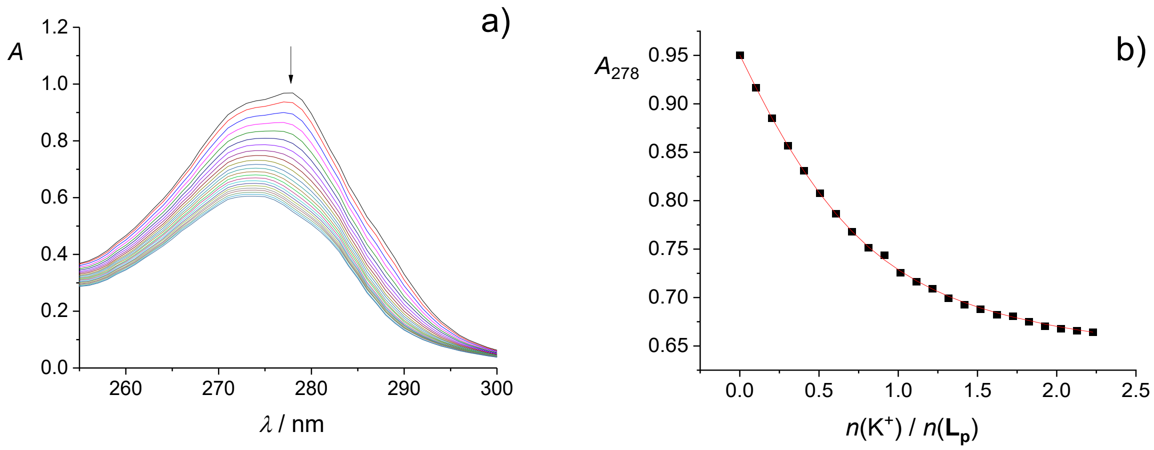

Figure S46. a) Spectrophotometric titration of $\mathbf{L_p}$ (c = 2.69 × 10^{-4} mol dm^{-3}) with $KClO_4$ (c = 3.01 × 10^{-3} mol dm^{-3}) in MeCN at 25 °C; $V_0(\mathbf{L_p})$ = 2.2 cm^3; l = 1 cm. Spectra are corrected for dilution. b) Absorbance of $\mathbf{L_p}$ at 278 nm as a function of cation to ligand molar ratio. ■ experimental; — calculated.

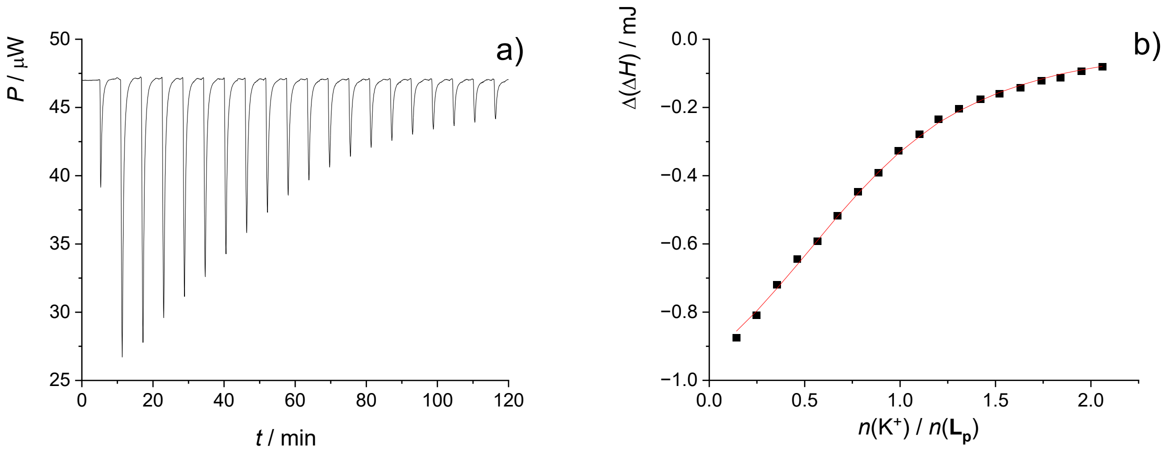

Figure S47. a) Microcalorimetric titration of $\mathbf{L_p}$ (c = 2.90 × 10^{-4} mol dm^{-3}, V = 1.42 cm^3) with $KClO_4$ (c = 2.91 × 10^{-3} mol dm^{-3}) in MeCN at 25 °C. b) Dependence of successive enthalpy changes on cation to ligand molar ratio. ■ experimental; — calculated.

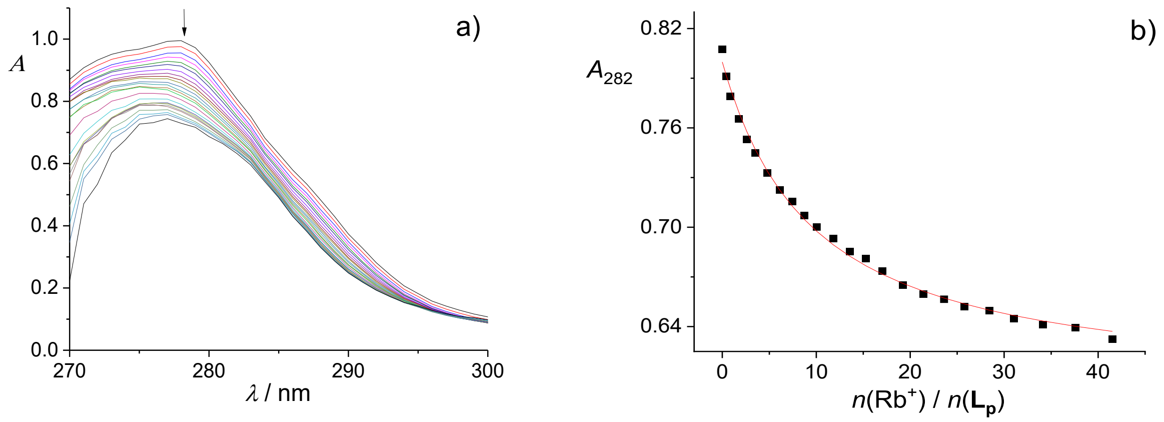

Figure S48. a) Spectrophotometric titration of $\mathbf{L_p}$ (c = 2.32 × 10^{-4} mol dm^{-3}) with RbI (c = 2.03 × 10^{-2} mol dm^{-3}) in MeCN at 25 °C; $V_0(\mathbf{L_p})$ = 2.2 cm^3; l = 1 cm. Spectra are corrected for dilution and absorption of I^-. b) Absorbance of $\mathbf{L_p}$ at 282 nm as a function of cation to ligand molar ratio. ■ experimental; — calculated.

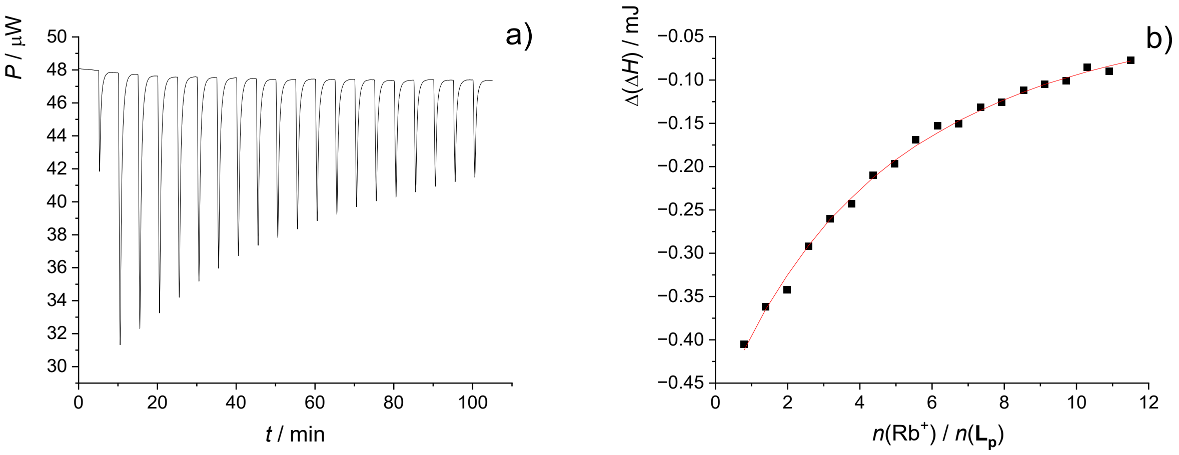

Figure S49. a) Microcalorimetric titration of $\mathbf{L_p}$ (c = 3.28 × 10^{-4} mol dm^{-3}, V = 1.42 cm^3) with RbI (c = 1.86 × 10^{-2} mol dm^{-3}) in MeCN at 25 °C. b) Dependence of successive enthalpy changes on cation to ligand molar ratio. ■ experimental; — calculated.

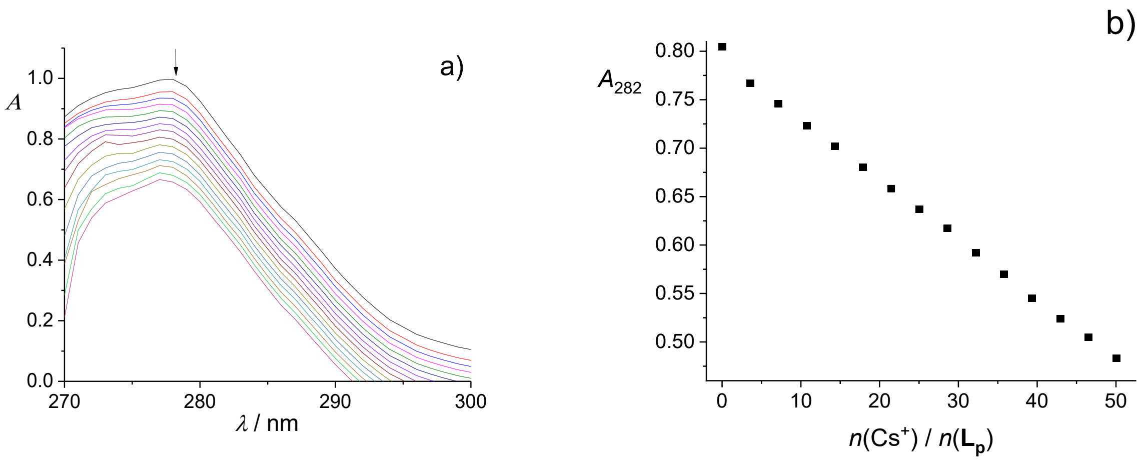

Figure S50. a) Spectrophotometric titration of **L$_p$** (c = 2.32 × 10^{-4} mol dm^{-3}) with CsI (c = 1.66 × 10^{-2} mol dm^{-3}) in MeCN at 25 °C; V_0(**L$_p$**) = 2.0 cm^3; l = 1 cm. Spectra are corrected for dilution and absorption of I$^-$. b) Absorbance of **L$_p$** at 282 nm as a function of cation to ligand molar ratio.

S2.4.2. Solvent: methanol

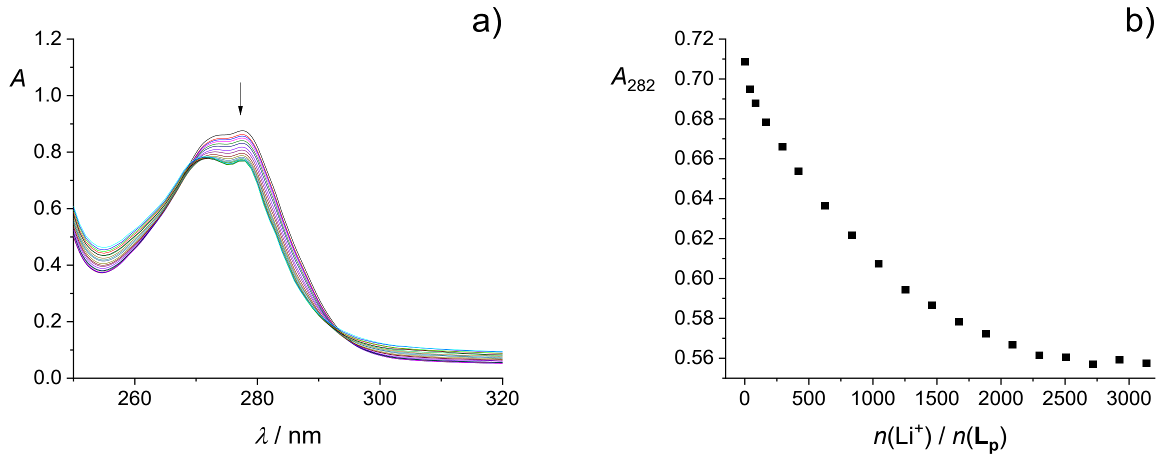

Figure S51. a) Spectrophotometric titration of $\mathbf{L_p}$ ($c = 2.32 \times 10^{-4}$ mol dm^{-3}) with $LiClO_4$ ($c = 9.64 \times 10^{-1}$ mol dm^{-3}) in MeOH; $V_0(\mathbf{L_p}) = 1.9$ cm^3; $l = 1$ cm. Spectra are corrected for dilution. b) Absorbance of $\mathbf{L_p}$ at 282 nm as a function of cation to ligand molar ratio.

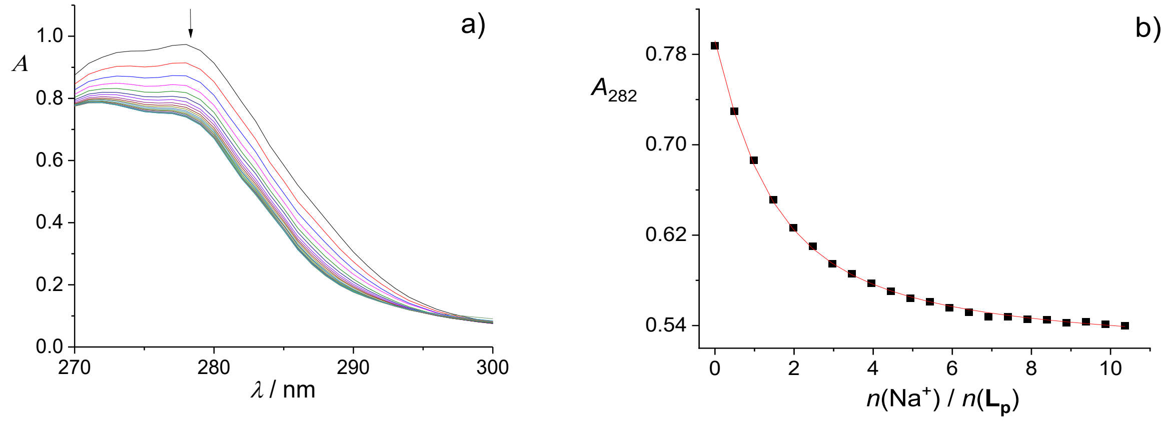

Figure S52. a) Spectrophotometric titration of $\mathbf{L_p}$ ($c = 2.32 \times 10^{-4}$ mol dm^{-3}) with $NaClO_4$ ($c = 5.03 \times 10^{-3}$ mol dm^{-3}) in MeOH; $V_0(\mathbf{L_p}) = 2.2$ cm^3; $l = 1$ cm. Spectra are corrected for dilution. b) Absorbance of $\mathbf{L_p}$ at 282 nm as a function of cation to ligand molar ratio. ■ experimental; — calculated.

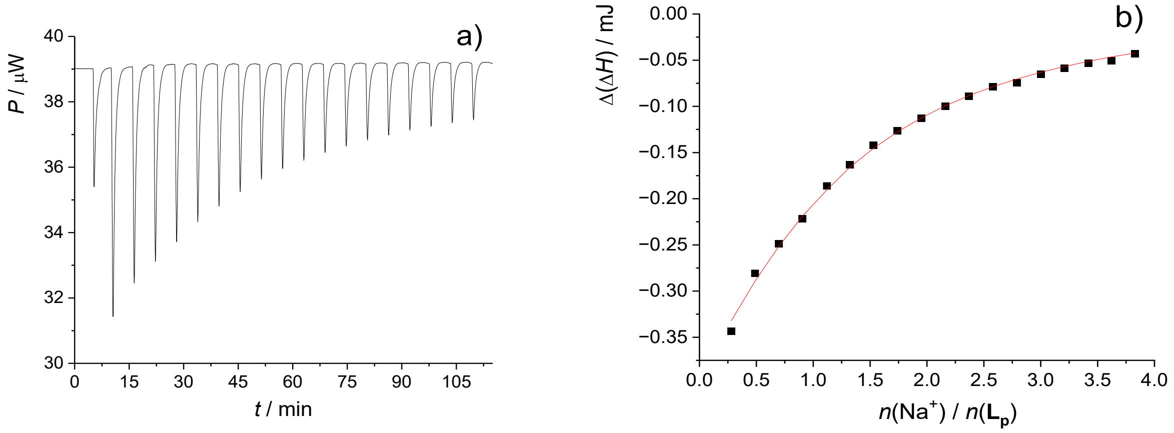

Figure S53. a) Microcalorimetric titration of **Lp** ($c = 2.10 \times 10^{-4}$ mol dm^{-3}, $V = 1.42$ cm^3) with $NaClO_4$ ($c = 4.17 \times 10^{-3}$ mol dm^{-3}) in MeOH at 25 °C. b) Dependence of successive enthalpy changes on cation to ligand molar ratio. ■ experimental; — calculated.

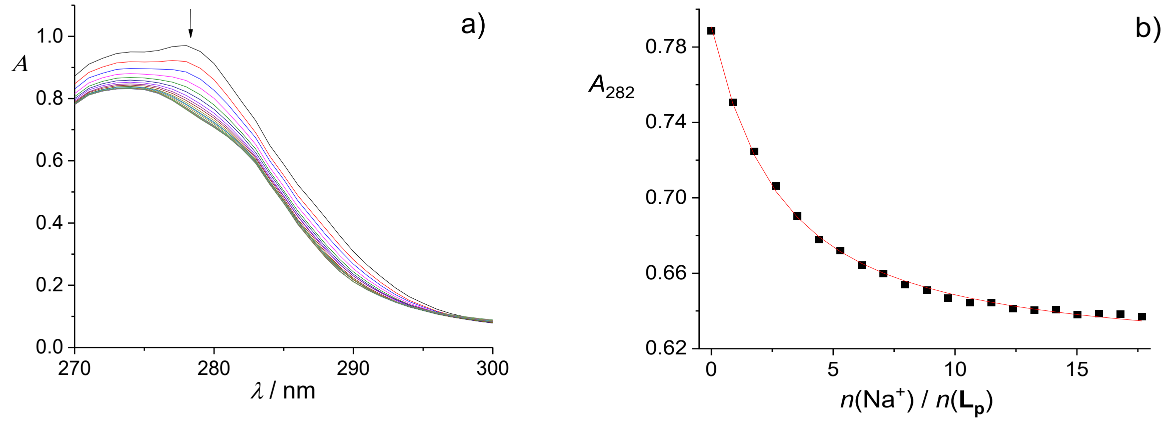

Figure S54. a) Spectrophotometric titration of **Lp** ($c = 2.32 \times 10^{-4}$ mol dm^{-3}) with KCl ($c = 9.00 \times 10^{-3}$ mol dm^{-3}) in MeOH; V_0(**Lp**) = 2.2 cm^3; $l = 1$ cm. Spectra are corrected for dilution. b) Absorbance of **Lp** at 282 nm as a function of cation to ligand molar ratio. ■ experimental; — calculated.

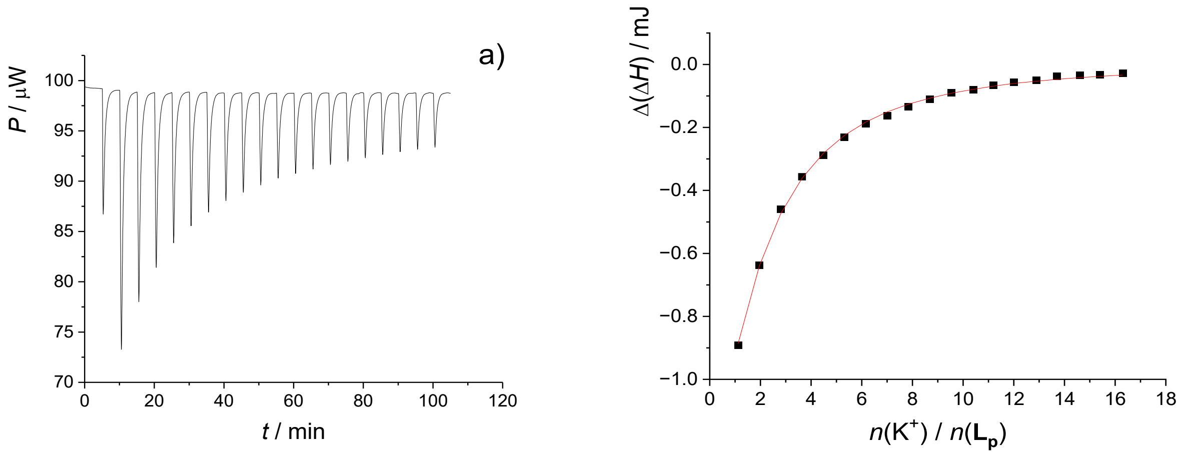

Figure S55. a) Microcalorimetric titration of $\mathbf{L_p}$ (c = 2.10 × 10^{-4} mol dm^{-3}, V = 1.42 cm^3) with KCl (c = 1.68 × 10^{-2} mol dm^{-3}) in MeOH at 25 °C. b) Dependence of successive enthalpy changes on cation to ligand molar ratio. ■ experimental; — calculated.

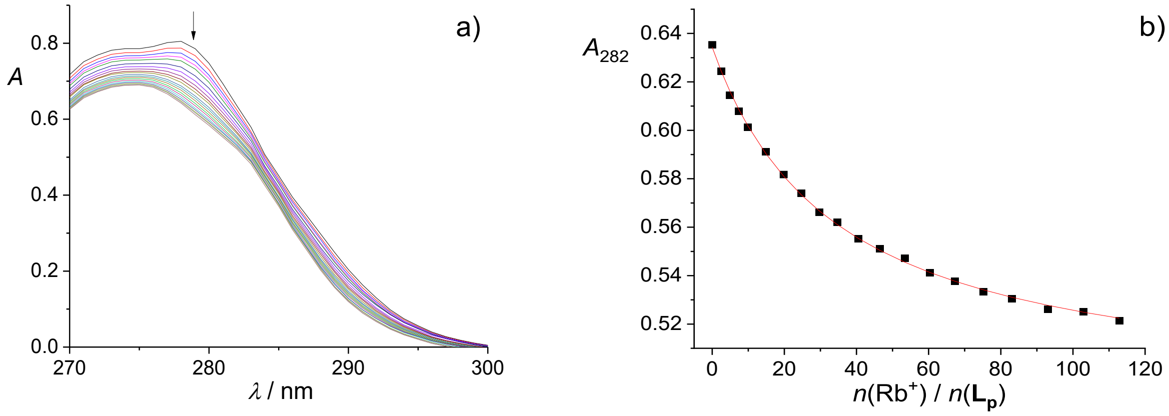

Figure S56. a) Spectrophotometric titration of $\mathbf{L_p}$ (c = 2.32 × 10^{-4} mol dm^{-3}) with RbCl (c = 5.05 × 10^{-2} mol dm^{-3}) in MeOH; $V_0(\mathbf{L_p})$ = 2.2 cm^3; l = 1 cm. Spectra are corrected for dilution. b) Absorbance of $\mathbf{L_p}$ at 282 nm as a function of cation to ligand molar ratio. ■ experimental; — calculated.

S2.5. Alkaline earth metal cation complexation

S2.5.1. Solvent: acetonitrile

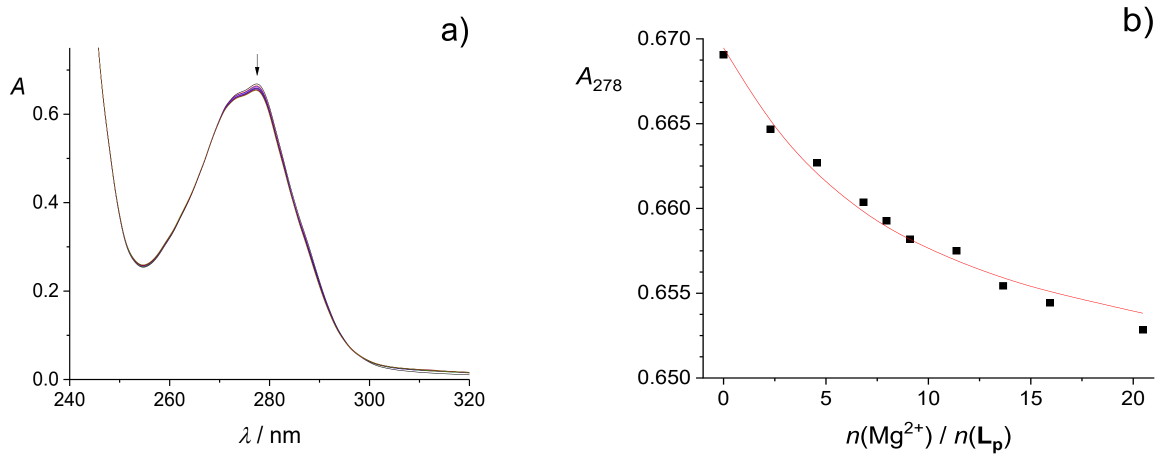

Figure S57. a) Spectrophotometric titration of $\mathbf{L_p}$ ($c = 1.99 \times 10^{-4}$ mol dm^{-3}) with $Mg(ClO_4)_2$ ($c = 9.97 \times 10^{-3}$ mol dm^{-3}) in MeCN at 25 °C; $V_0(\mathbf{L_p}) = 2.2$ cm^3; $l = 1$ cm. Spectra are corrected for dilution. b) Absorbance of $\mathbf{L_p}$ at 278 nm as a function of cation to ligand molar ratio. ■ experimental; — calculated.

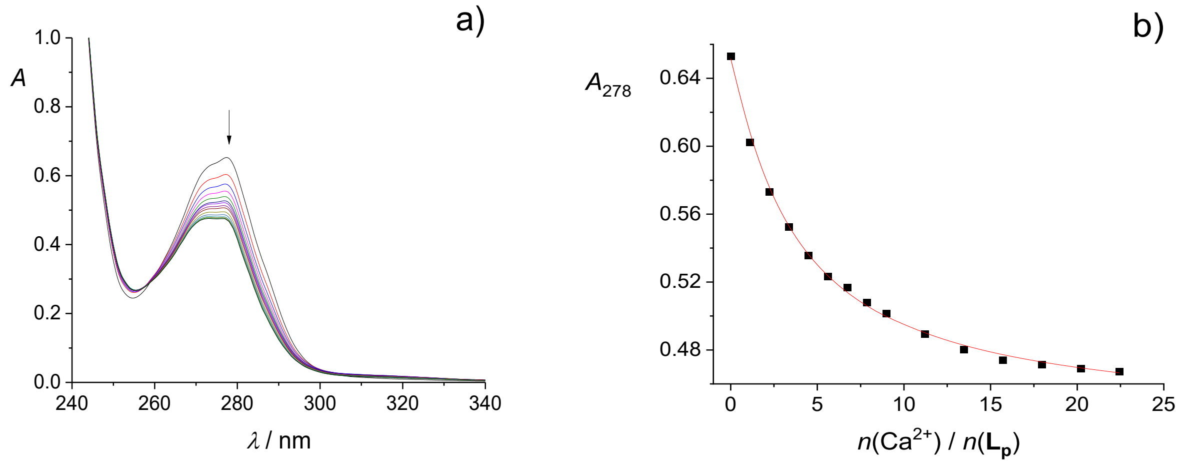

Figure S58. a) Spectrophotometric titration of $\mathbf{L_p}$ ($c = 1.99 \times 10^{-4}$ mol dm^{-3}) with $Ca(ClO_4)_2$ ($c = 9.84 \times 10^{-3}$ mol dm^{-3}) in MeCN at 25 °C; $V_0(\mathbf{L_p}) = 2.2$ cm^3; $l = 1$ cm. Spectra are corrected for dilution. b) Absorbance of $\mathbf{L_p}$ at 278 nm as a function of cation to ligand molar ratio. ■ experimental; — calculated.

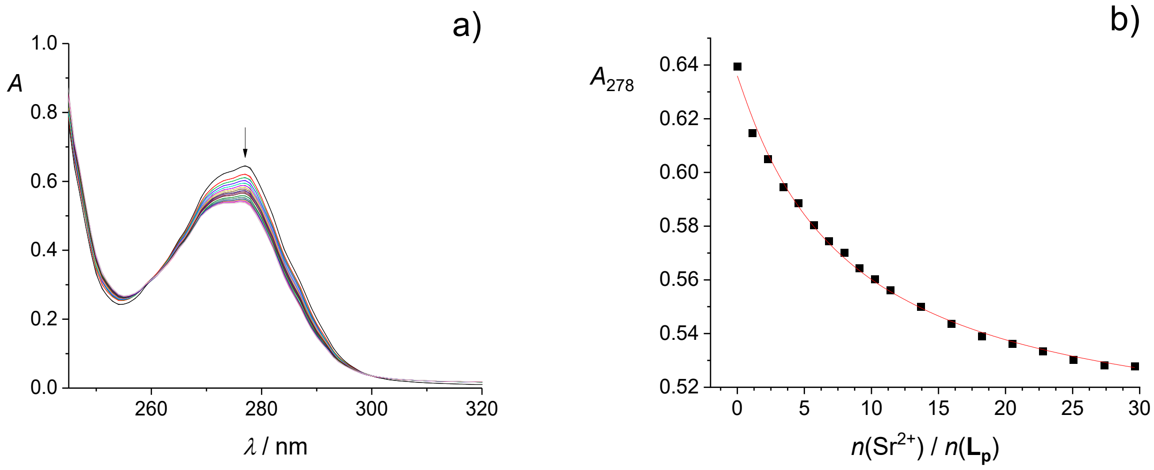

Figure S59. a) Spectrophotometric titration of $\mathbf{L_p}$ ($c = 1.99 \times 10^{-4}$ mol dm^{-3}) with $Sr(ClO_4)_2 \times 3H_2O$ ($c = 9.97 \times 10^{-3}$ mol dm^{-3}) in MeCN at 25 °C; $V_0(\mathbf{L_p}) = 2.2$ cm^3; $l = 1$ cm. Spectra are corrected for dilution. b) Absorbance of $\mathbf{L_p}$ at 278 nm as a function of cation to ligand molar ratio. ■ experimental; — calculated.

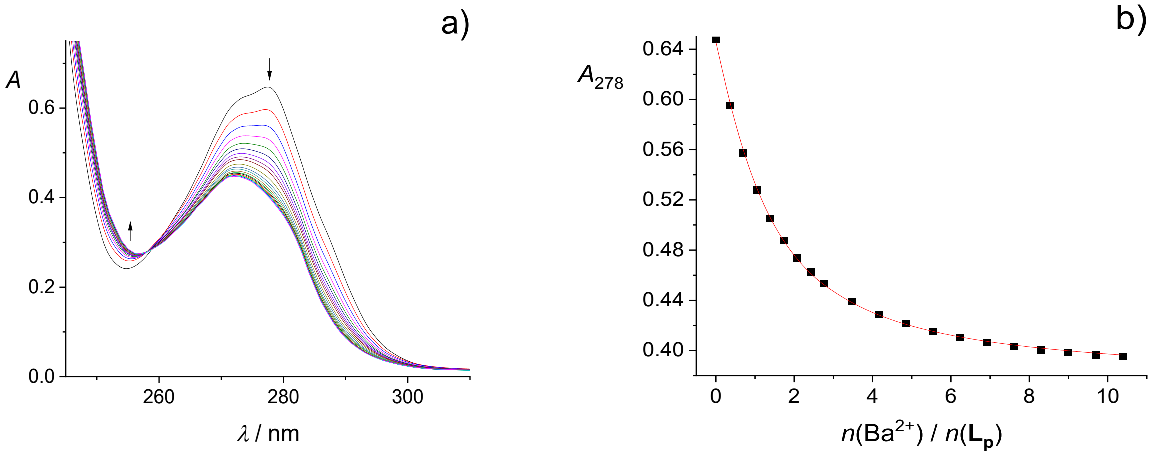

Figure S60. a) Spectrophotometric titration of $\mathbf{L_p}$ ($c = 1.99 \times 10^{-4}$ mol dm^{-3}) with $Ba(ClO_4)_2$ ($c = 6.07 \times 10^{-3}$ mol dm^{-3}) in MeCN at 25 °C; $V_0(\mathbf{L_p}) = 2.2$ cm^3; $l = 1$ cm. Spectra are corrected for dilution. b) Absorbance of $\mathbf{L_p}$ at 278 nm as a function of cation to ligand molar ratio. ■ experimental; — calculated.

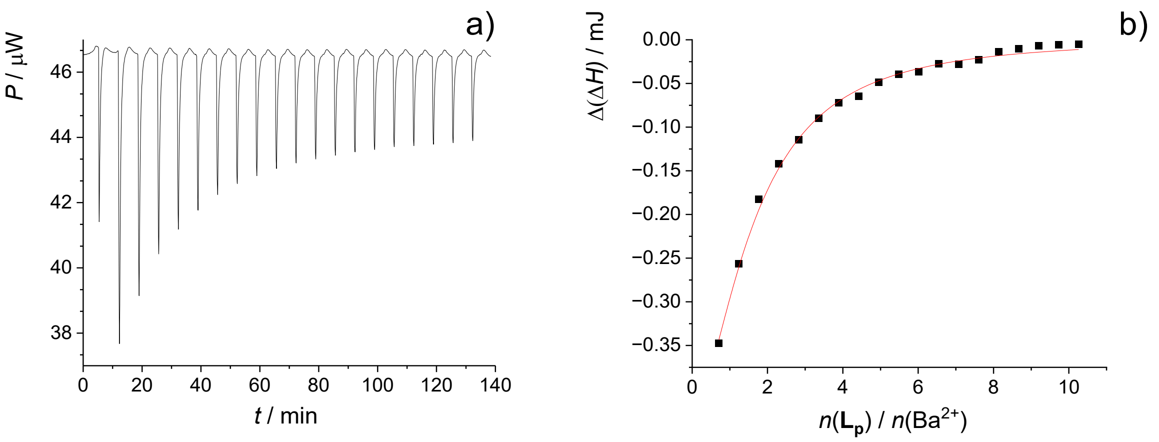

Figure S61. a) Microcalorimetric titration of $Ba(ClO_4)_2$ ($c = 1.5 \times 10^{-4}$ mol dm^{-3}) with $\mathbf{L_p}$ ($c = 7.6 \times 10^{-3}$ mol dm^{-3}) in MeCN at 25 °C; $V(Ba(ClO_4)_2) = 1.42$ cm^3. b) Dependence of successive enthalpy changes on ligand to cation molar ratio. ■ experimental; — calculated.

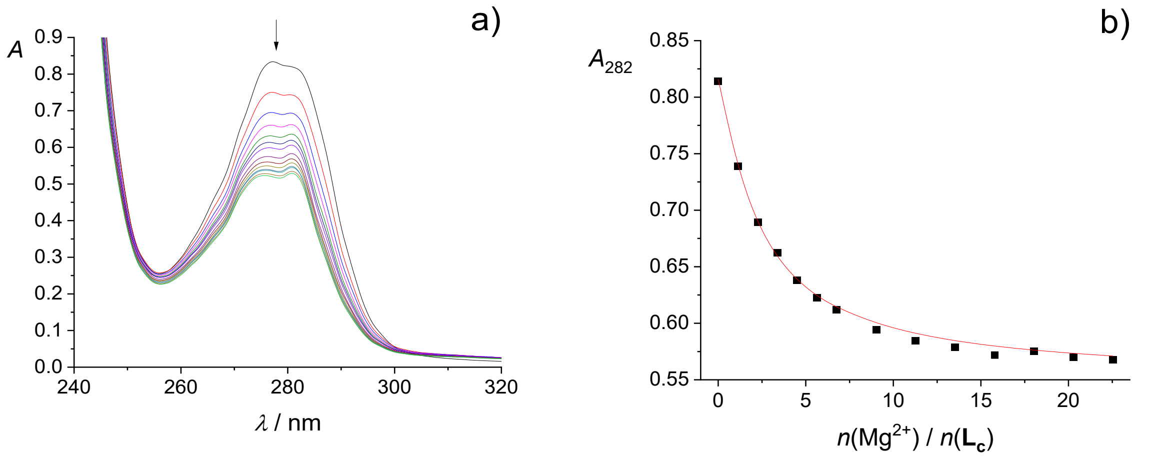

Figure S62. a) Spectrophotometric titration of $\mathbf{L_c}$ ($c = 2.02\times10^{-4}$ mol dm^{-3}) with $Mg(ClO_4)_2$ ($c = 5.00\times10^{-2}$ mol dm^{-3}) in MeCN; ϑ = (25.0 ± 0.1) °C; $V_0(\mathbf{L_c}) = 2.2$ cm^3; $l = 1$ cm. Spectra are corrected for dilution. b) Absorbance of $\mathbf{L_c}$ at 282 nm as a function of cation to ligand molar ratio. ■ experimental; — calculated.

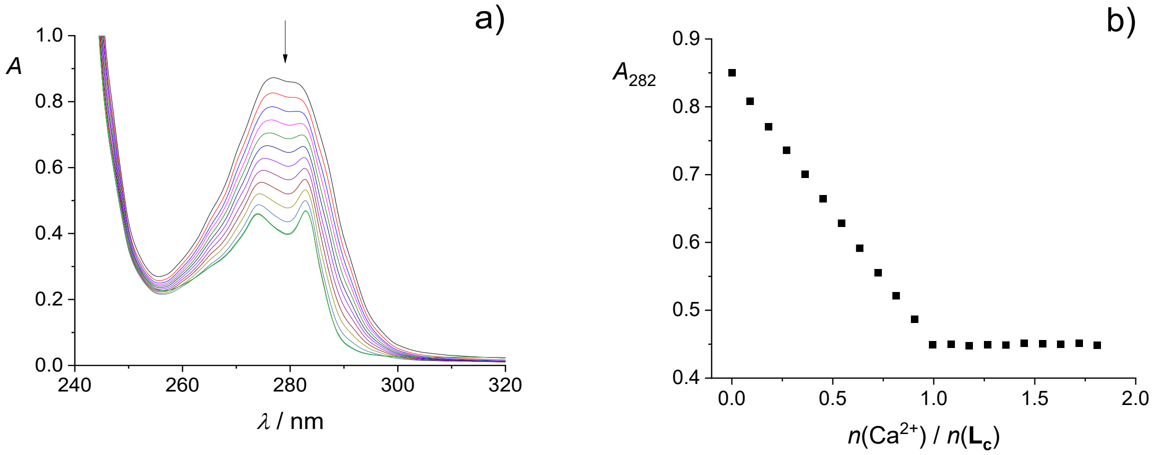

Figure S63. a) Spectrophotometric titration of $\mathbf{L_c}$ ($c = 2.03\times10^{-4}$ mol dm^{-3}) with Ca(trf)$_2$ ($c = 2.02\times10^{-3}$ mol dm^{-3}) in MeCN ; ϑ = (25.0 ± 0.1) °C; $V_0(\mathbf{L_c})$ = 2.2 cm^3; l = 1 cm. Spectra are corrected for dilution. b) Absorbance of $\mathbf{L_c}$ at 282 nm as a function of cation to ligand molar ratio.

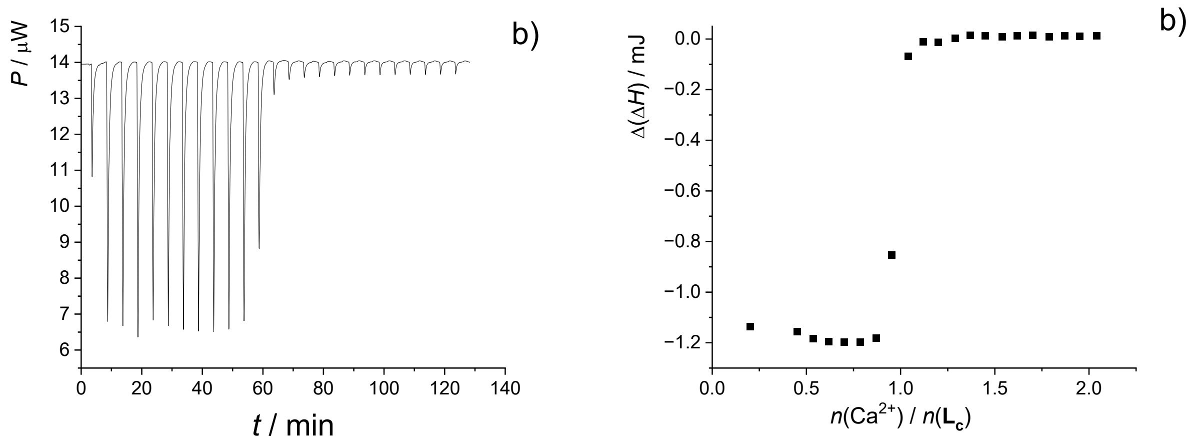

Figure S64. a) Microcalorimetric titration of $\mathbf{L_c}$ (c = 2.03×10^{-4} mol dm^{-3}, V = 1.43 cm^3) with Ca(trf)$_2$ (c = 2.02×10^{-3} mol dm^{-3}) in MeCN at 25 °C. b) Dependence of successive enthalpy changes on cation to ligand molar ratio.

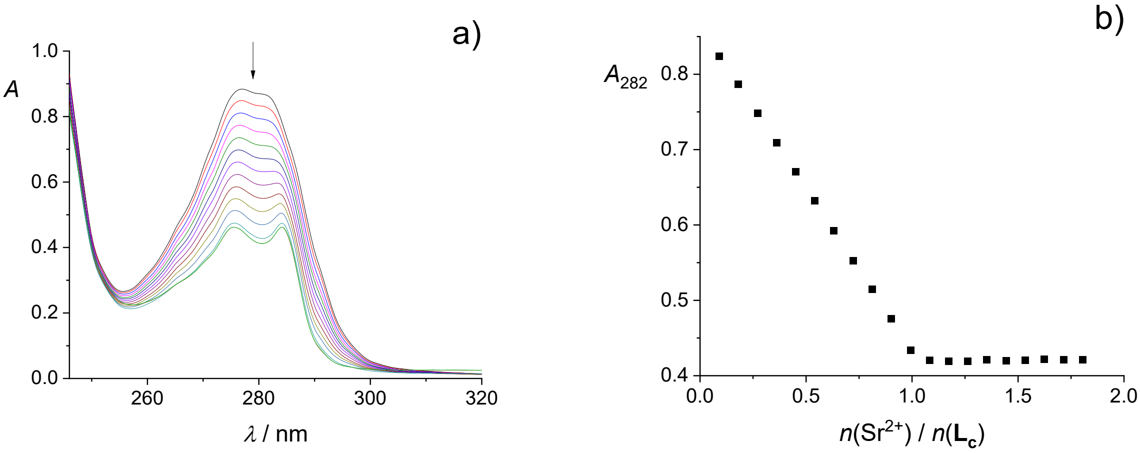

Figure S65. a) Spectrophotometric titration of $\mathbf{L_c}$ (c = 2.03×10^{-4} mol dm^{-3}) with $Sr(ClO_4)_2$×3H_2O (c = 2.01×10^{-3} mol dm^{-3}) in MeCN; ϑ = (25.0 ± 0.1) °C; $V_0(\mathbf{L_c})$ = 2.2 cm^3; l = 1 cm. Spectra are corrected for dilution. b) Absorbance of $\mathbf{L_c}$ at 282 nm as a function of cation to ligand molar ratio.

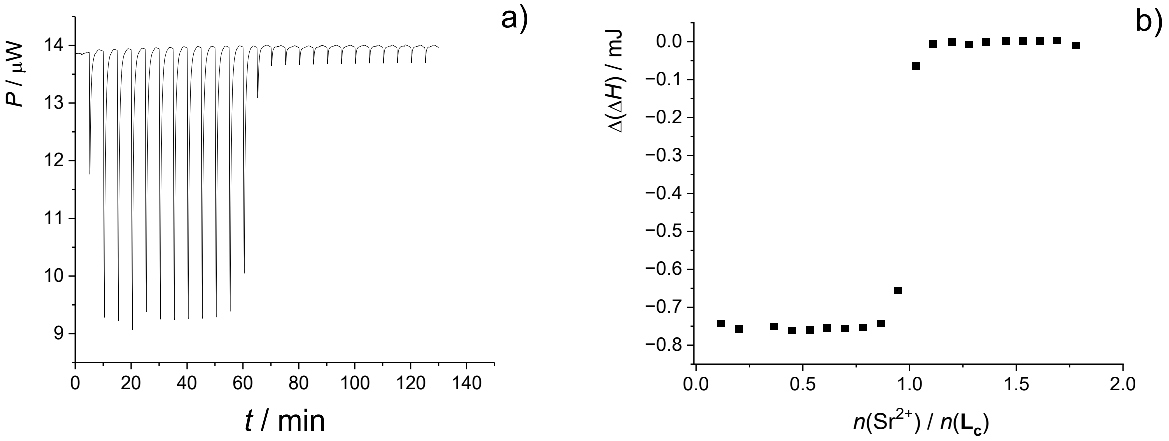

Figure S66. a) Microcalorimetric titration of $\mathbf{L_c}$ (c = 2.03×10^{-4} mol dm^{-3}, V = 1.43 cm^3) with $Sr(ClO_4)_2$×3H_2O (c = 2.01×10^{-3} mol dm^{-3}) in MeCN at 25 °C. b) Dependence of successive enthalpy changes on cation to ligand molar ratio.

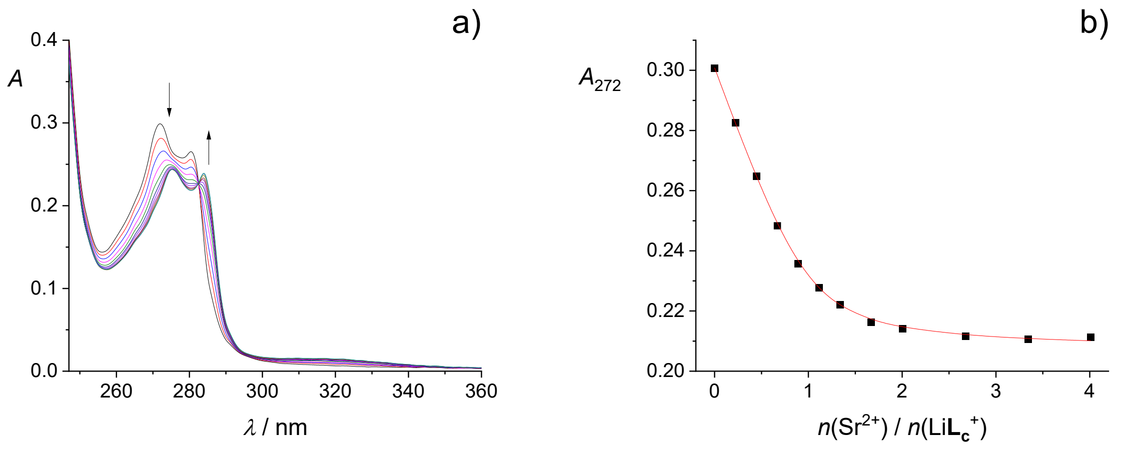

Figure S67. a) Spectrophotometric competitive titration of LiL_c^+ (c = 1.02×10^{-4} mol dm^{-3}) with $Sr(ClO_4)_2$×3H_2O (c = 1.00×10^{-3} mol dm^{-3}) in MeCN; ϑ = (25.0 ± 0.1) °C; $V_0(\mathbf{L_c})$ = 2.2 cm^3; l = 1 cm. Spectra are corrected for dilution. b) Absorbance of $\mathbf{L_c}$ at 272 nm as a function of cation to ligand molar ratio. ■ experimental; — calculated.

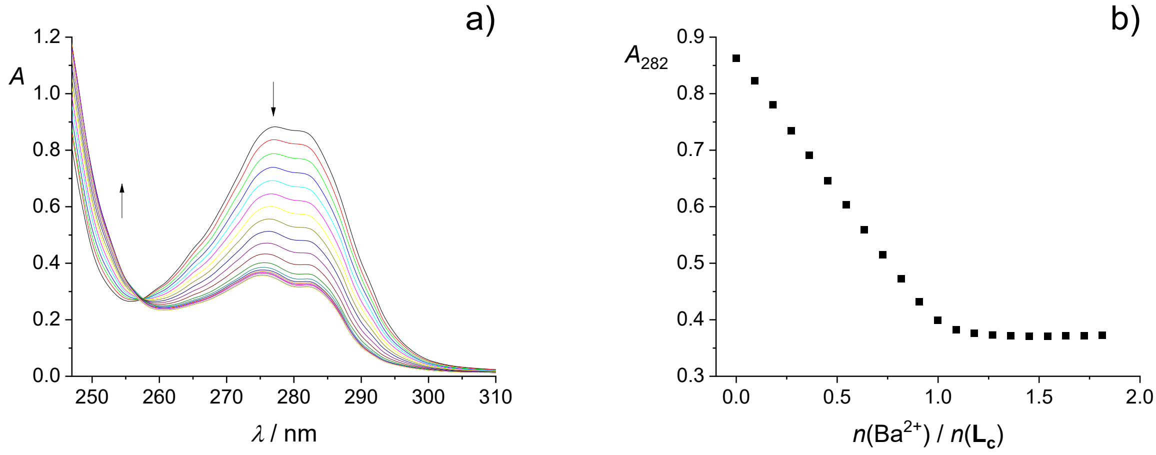

Figure S68. a) Spectrophotometric titration of $\mathbf{L_c}$ (c = 2.03×10^{-4} mol dm^{-3}) with Ba(trf)$_2$ (c = 2.02×10^{-3} mol dm^{-3}) in MeCN; ϑ = (25.0 ± 0.1) °C; $V_0(\mathbf{L_c})$ = 2.2 cm^3; l = 1 cm. Spectra are corrected for dilution. b) Absorbance of $\mathbf{L_c}$ at 282 nm as a function of cation to ligand molar ratio.

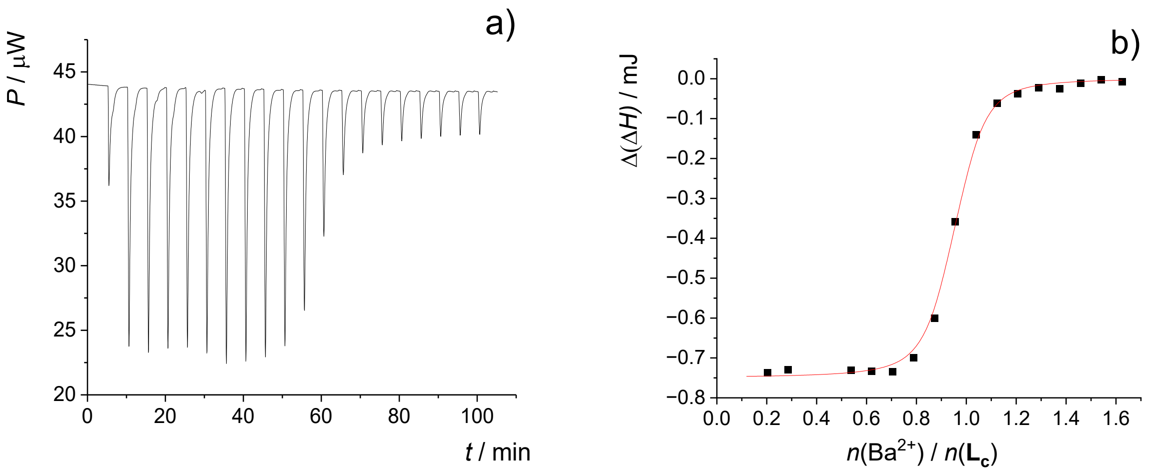

Figure S69. a) Microcalorimetric titration of **L**c (c = 2.03×10^{-4} mol dm^{-3}, V = 1.43 cm^3) with Ba(trf)$_2$ (c = 2.02 × 10^{-3} mol dm^{-3}) in MeCN at 25 °C. b) Dependence of successive enthalpy changes on cation to ligand molar ratio. ■ experimental; — calculated.

S2.5.2. Solvent: methanol

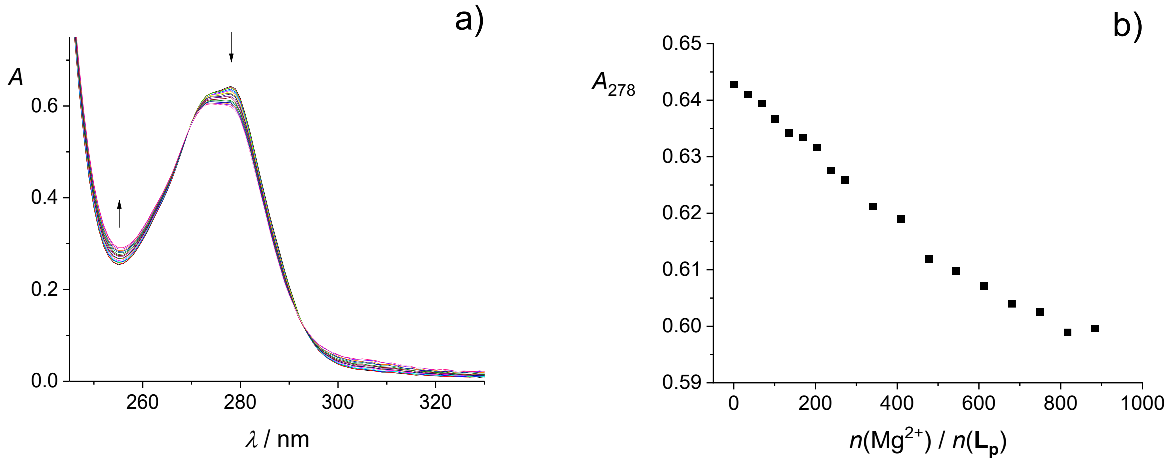

Figure S70. a) Spectrophotometric titration of **Lp** ($c = 2.01 \times 10^{-4}$ mol dm^{-3}) with $Mg(ClO_4)_2$ ($c = 3.01 \times 10^{-1}$ mol dm^{-3}) in MeOH; V_0(**Lp**) = 2.2 cm^3; $l = 1$ cm. Spectra are corrected for dilution. b) Absorbance of **Lp** at 278 nm as a function of cation to ligand molar ratio.

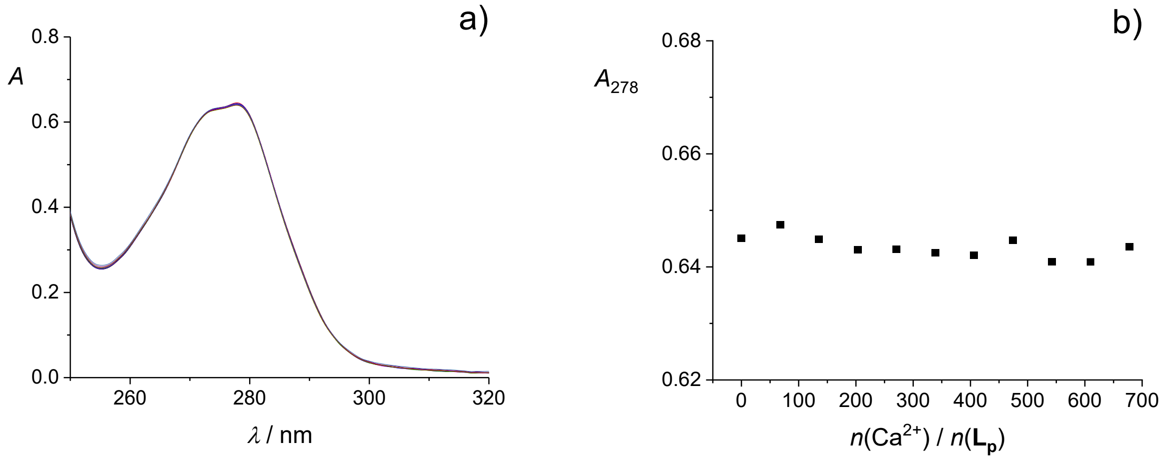

Figure S71. a) Spectrophotometric titration of **Lp** ($c = 2.01 \times 10^{-4}$ mol dm^{-3}) with $Ca(ClO_4)_2$ ($c = 3.00 \times 10^{-1}$ mol dm^{-3}) in MeOH; V_0(**Lp**) = 2.2 cm^3; $l = 1$ cm. Spectra are corrected for dilution. b) Absorbance of **Lp** at 278 nm as a function of cation to ligand molar ratio.

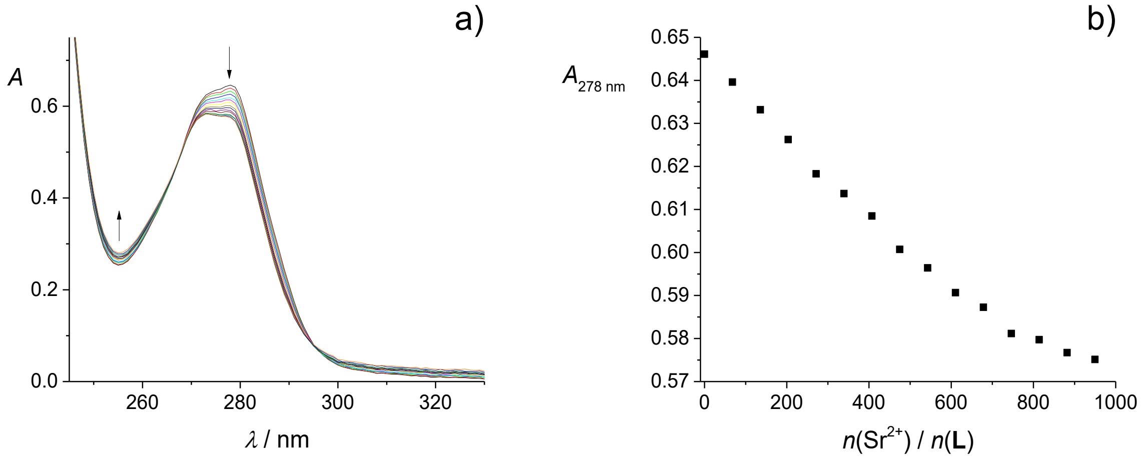

Figure S72. a) Spectrophotometric titration of $\mathbf{L_p}$ ($c = 2.01 \times 10^{-4}$ mol dm^{-3}) with $Sr(ClO_4)_2 \times 3H_2O$ ($c = 3.00 \times 10^{-1}$ mol dm^{-3}) in MeOH; $V_0(\mathbf{L_p}) = 2.2$ cm^3; $l = 1$ cm. Spectra are corrected for dilution. b) Absorbance of $\mathbf{L_p}$ at 278 nm as a function of cation to ligand molar ratio.

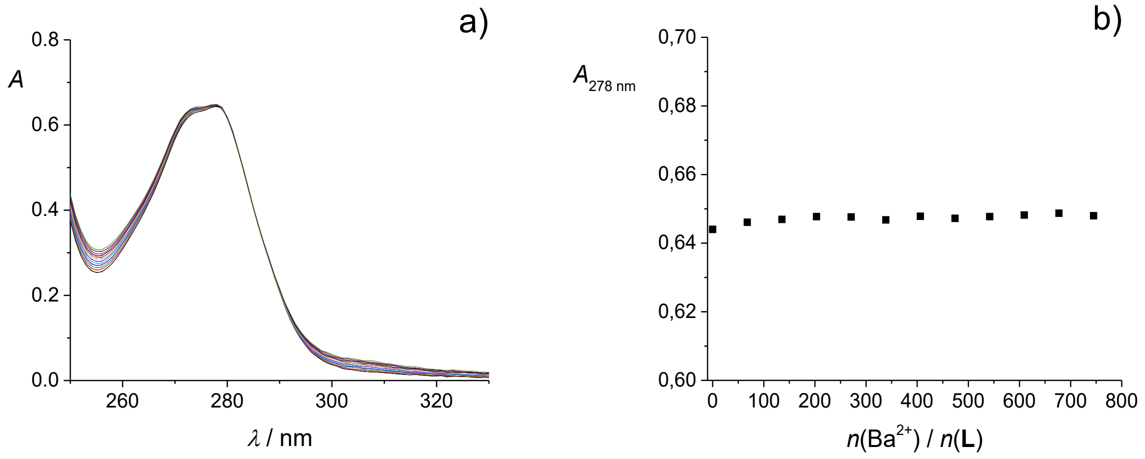

Figure S73. a) Spectrophotometric titration of $\mathbf{L_p}$ ($c = 2.01 \times 10^{-4}$ mol dm^{-3}) with $Ba(ClO_4)_2$ ($c = 3.00 \times 10^{-1}$ mol dm^{-3}) in MeOH; $V_0(\mathbf{L_p}) = 2.2$ cm^3; $l = 1$ cm. Spectra are corrected for dilution. b) Absorbance of $\mathbf{L_p}$ at 278 nm as a function of cation to ligand molar ratio.

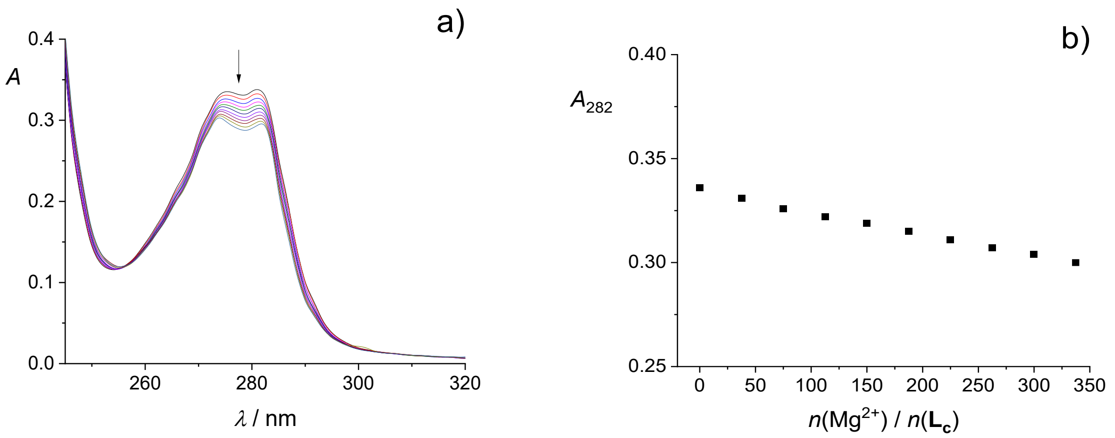

Figure S74. a) Spectrophotometric titration of $\mathbf{L_c}$ ($c = 9.8\times10^{-5}$ mol dm^{-3}) with $Mg(ClO_4)_2\times6H_2O$ ($c = 8.08\times10^{-2}$ mol dm^{-3}) in MeOH at 25 °C; $V_0(\mathbf{L_c}) = 2.2$ cm^3; $l = 1$ cm. Spectra are corrected for dilution. b) Absorbance of $\mathbf{L_c}$ at 282 nm as a function of cation to ligand molar ratio.

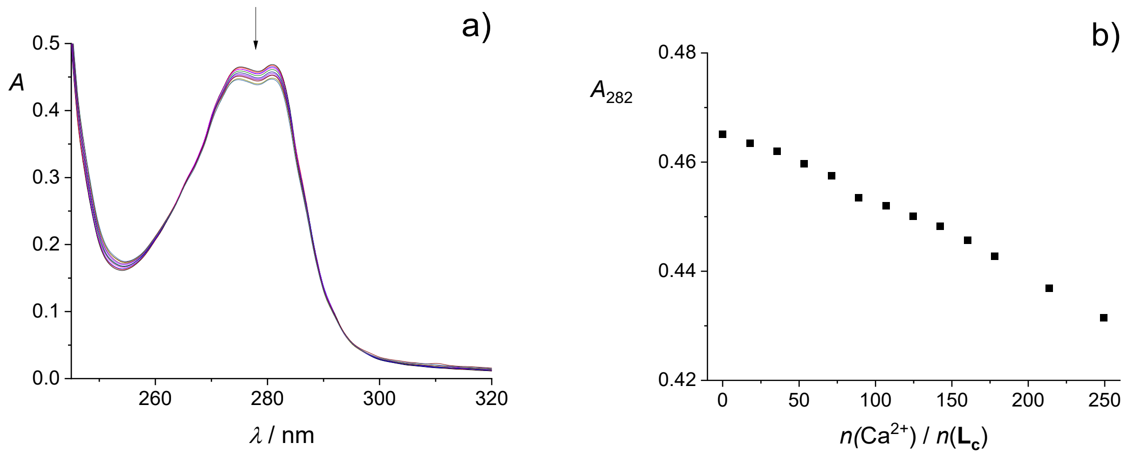

Figure S75. a) Spectrophotometric titration of $\mathbf{L_c}$ ($c = 1.52\times10^{-4}$ mol dm^{-3}) with $Ca(trf)_2$ ($c = 5.01\times10^{-3}$ mol dm^{-3}) in MeOH at 25 °C; $V_0(\mathbf{L_c}) = 2.2$ cm^3; $l = 1$ cm. Spectra are corrected for dilution. b) Absorbance of $\mathbf{L_c}$ at 282 nm as a function of cation to ligand molar ratio.

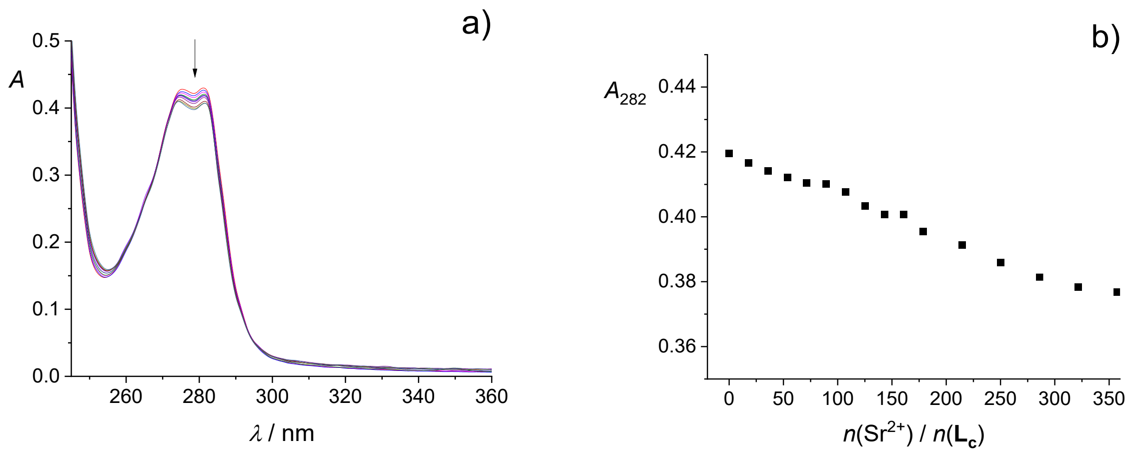

Figure S76. a) Spectrophotometric titration of $\mathbf{L_c}$ (c = 1.02×10^{-4} mol dm^{-3}) with $Sr(ClO_4)_2$×3H_2O (c = 8.02×10^{-2} mol dm^{-3}) in MeOH at 25 °C; $V_0(\mathbf{L_c})$ = 2.2 cm^3; l = 1 cm. Spectra are corrected for dilution. b) Absorbance of $\mathbf{L_c}$ at 282 nm as a function of cation to ligand molar ratio.

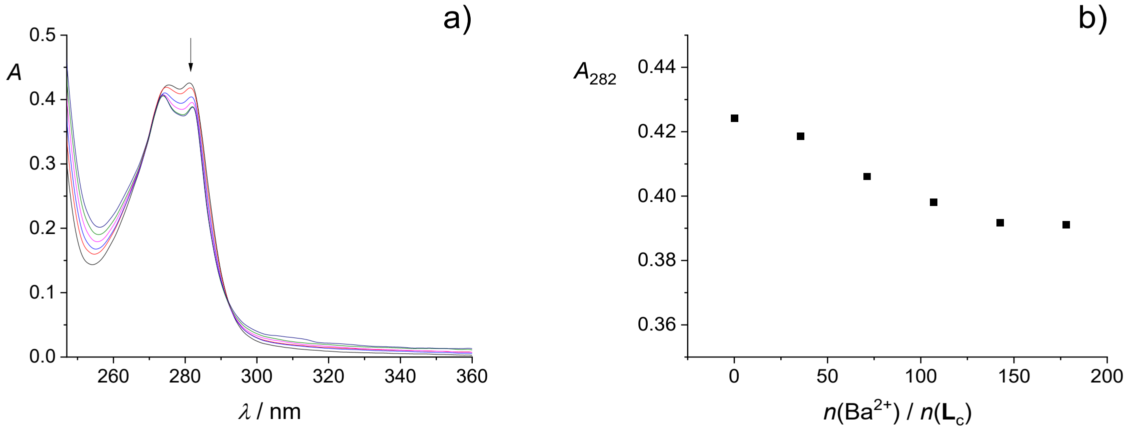

Figure S77. a) Spectrophotometric titration of $\mathbf{L_c}$ (c = 1.02×10^{-4} mol dm^{-3}) with $Ba(trf)_2$ (c = 7.99×10^{-2} mol dm^{-3}) in MeOH at 25 °C; $V_0(\mathbf{L_c})$ = 2.2 cm^3; l = 1 cm. Spectra are corrected for dilution. b) Absorbance of $\mathbf{L_c}$ at 282 nm as a function of cation to ligand molar ratio.

S2.6. Dissolution calorimetry

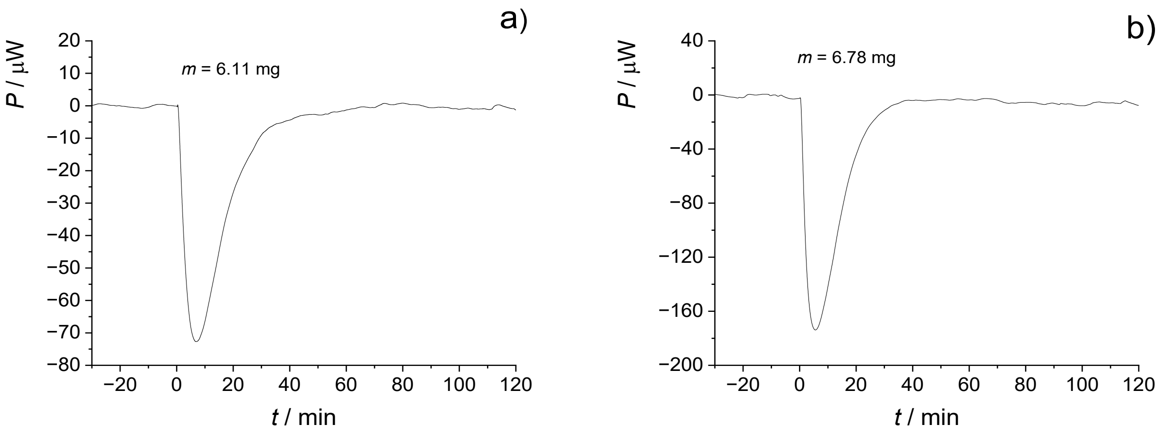

Figure S78. Dissolution of $\mathbf{L_p}$ in a) MeCN and b) MeOH at 25.0 °C; V = 16 mL.

Table S13. Thermodynamic dissolution parameters for $\mathbf{L_p}$ in MeCN and MeOH at 25 °C. Uncertainties of the last digit are given in parentheses as standard errors of the mean (N = 3).

solvent	$10^3\ s$ / mol dm^{-3}	$\Delta_{sol}G°$ / kJ mol^{-1}	$\Delta_{sol}H°$ / kJ mol^{-1}	$\Delta_{sol}S°$ / J K^{-1} mol^{-1}
MeCN	13.99(4)	10.58(1)	10.6(5)	≈0
MeOH	6.44(5)	12.51(2)	19.8(2)	24.8(7)

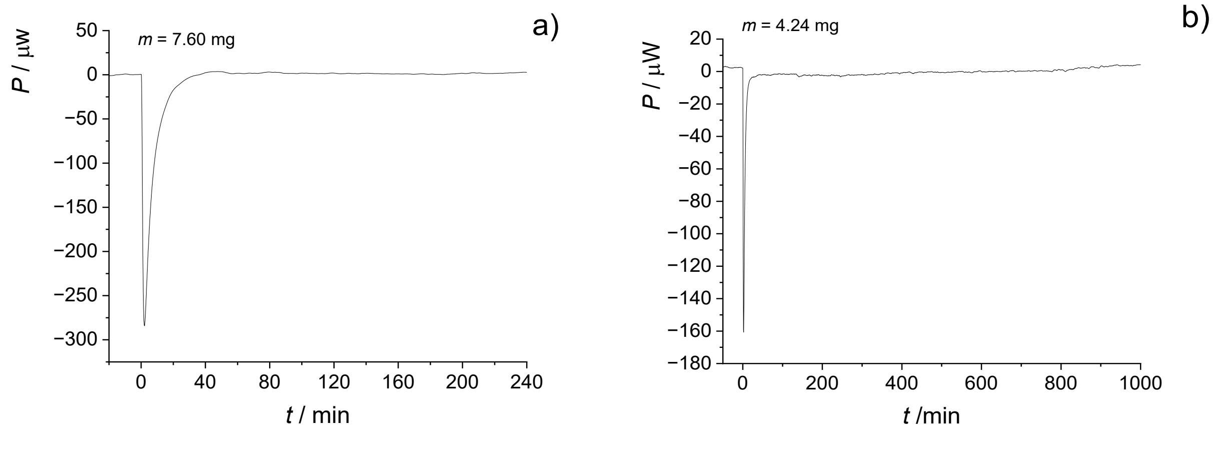

Figure S79. Dissolution of $\mathbf{L_c}$ in a) MeCN and b) MeOH at 25.0 °C; V = 16 mL.

Table S14. Thermodynamic dissolution parameters for **L_c** in MeCN and MeOH at 25 °C. Uncertainties of the last digit are given in parentheses as standard errors of the mean ($N = 3$).

solvent	$10^4\ s$ / mol dm^{-3}	$\Delta_{sol}G°$ / kJ mol^{-1}	$\Delta_{sol}H°$ / kJ mol^{-1}	$\Delta_{sol}S°$ / J K^{-1} mol^{-1}
MeCN	27.3[a]	14.6[a]	12.4[a]	−7.5[a]
			15.8(6)	
MeOH	4.46[a]	19.1[a]	16.5[a]	−8.7[a]

[a]data from ref. [2]

S2.7. Solubility measurements

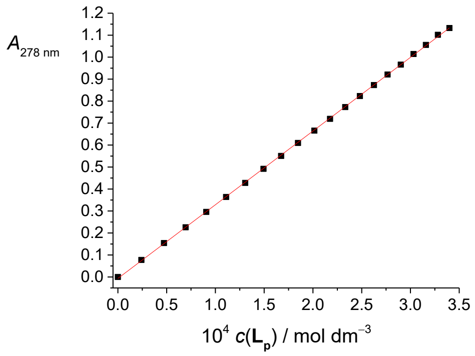

Figure S80. Determination of molar absorption coefficient of $\mathbf{L_p}$ in MeCN at 278 nm ($\vartheta = 25$ °C).

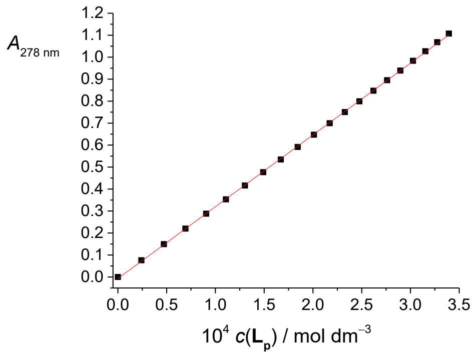

Figure S81. Determination of molar absorption coefficient of $\mathbf{L_p}$ in MeOH at 278 nm ($\vartheta = 25$ °C).

S2.8. NMR and ITC investigations of solvent inclusion into $\mathbf{L_c}$, $\mathbf{L_p}$, and their complexes

a)

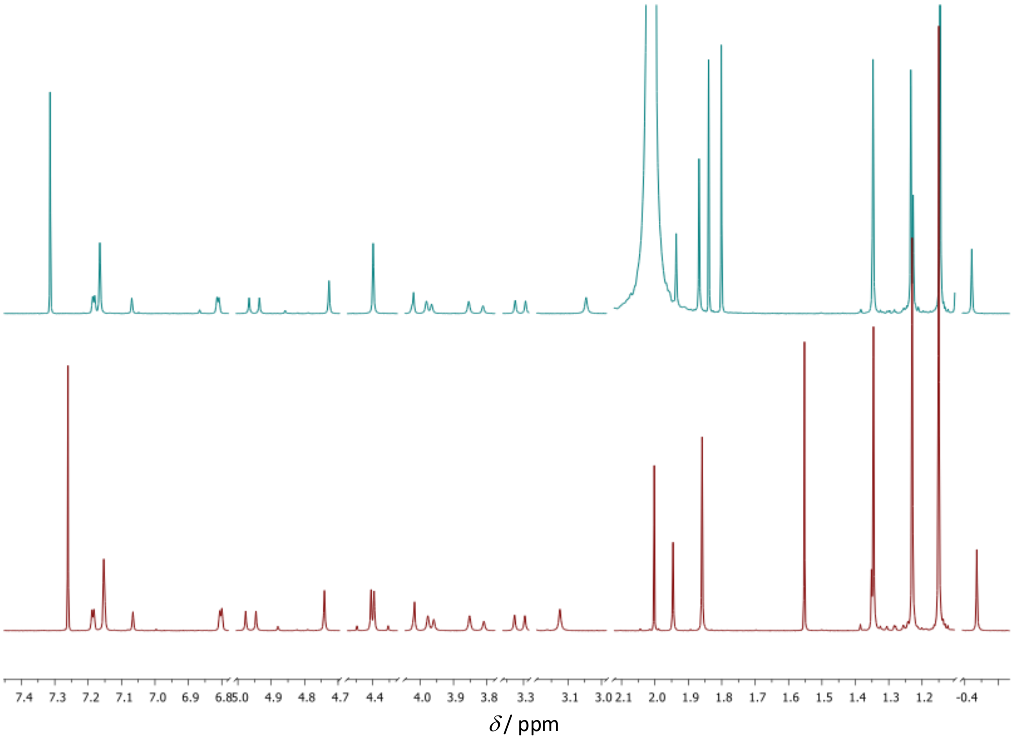

b)

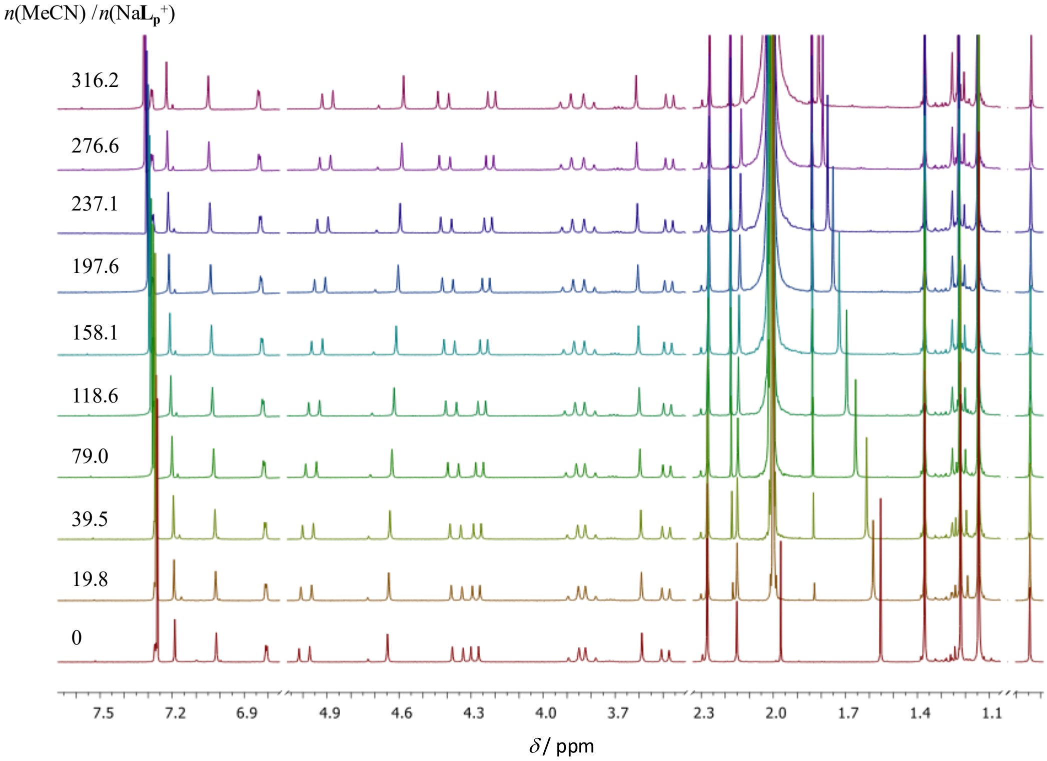

Figure S82. a) ^{1}H NMR spectra of $\mathbf{L_p}$ in $CDCl_3$ ($c_0 = 6.04 \times 10^{-3}$ mol dm^{-3}, $V_0 = 500$ µL) before (below) and after (above) addition of 160 µL acetonitrile solution ($c = 5.94$ mol dm^{-3}) at 25 °C. b) ^{1}H NMR titration of $Na\mathbf{L_p}^{+}$ ($c_0 = 6.01 \times 10^{-3}$ mol dm^{-3}) with acetonitrile solution ($c = 5.94$ mol dm^{-3}) at 25 °C.

a)

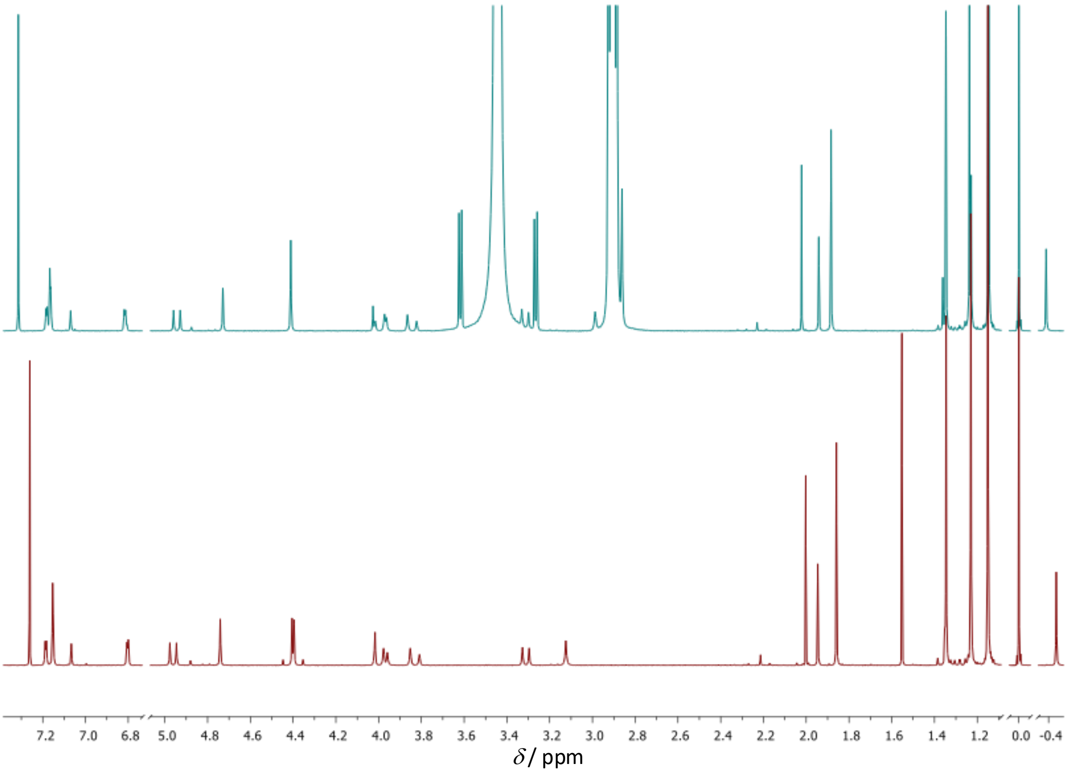

b)

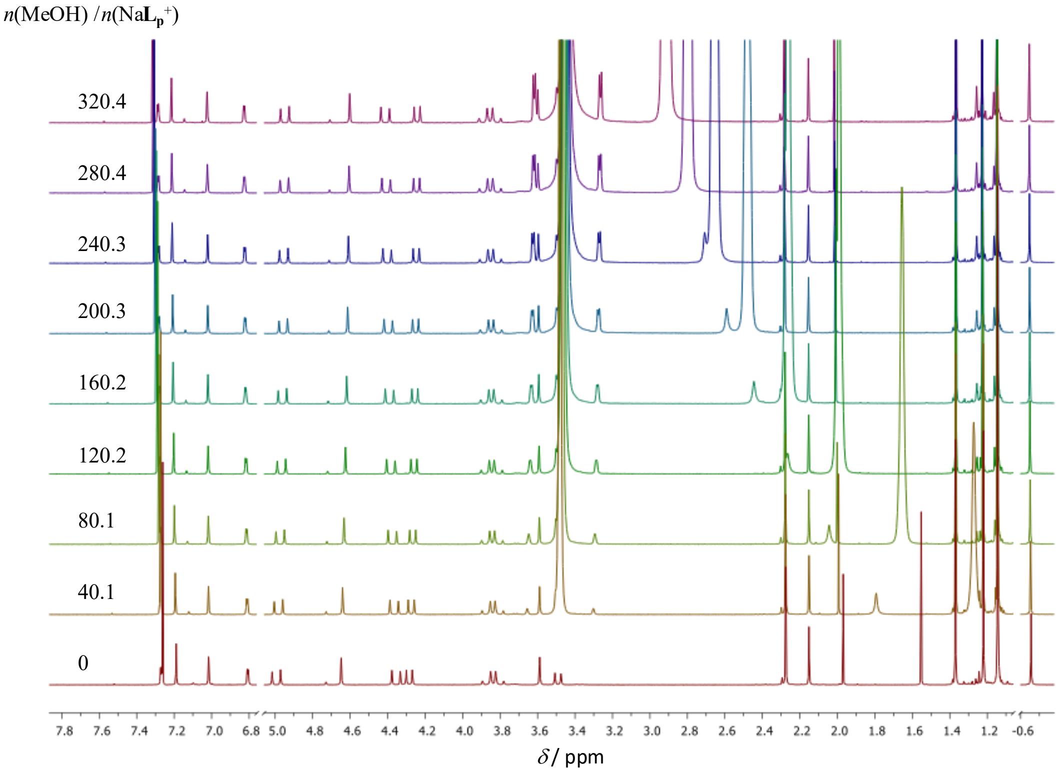

Figure S83. ^{1}H NMR spectra of $\mathbf{L_p}$ in $CDCl_3$ ($c_0 = 6.04 \times 10^{-3}$ mol dm^{-3}, V_0 = 500 μL) before (below) and after (above) addition 160 μL methanol solution (c = 6.01 mol dm^{-3}) at 25 °C. b) ^{1}H NMR titration of Na$\mathbf{L_p}^{+}$ ($c_0 = 6.01 \times 10^{-3}$ mol dm^{-3}) with methanol solution (c = 6.01 mol dm^{-3}) at 25 °C.

a)

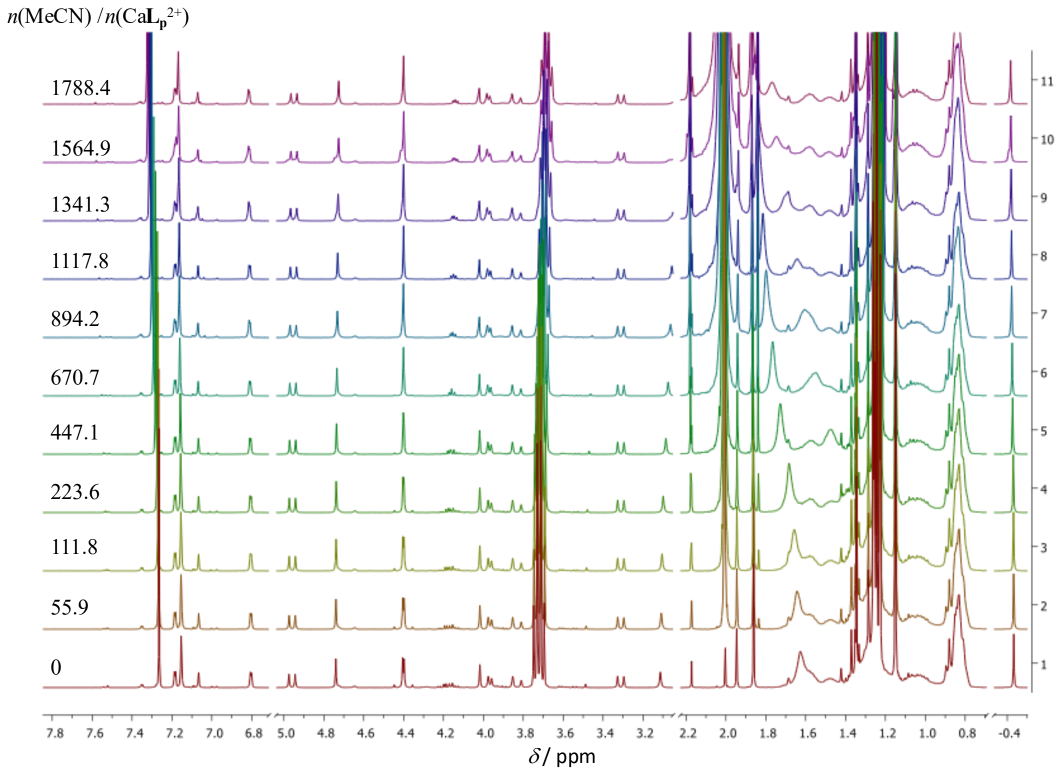

b)

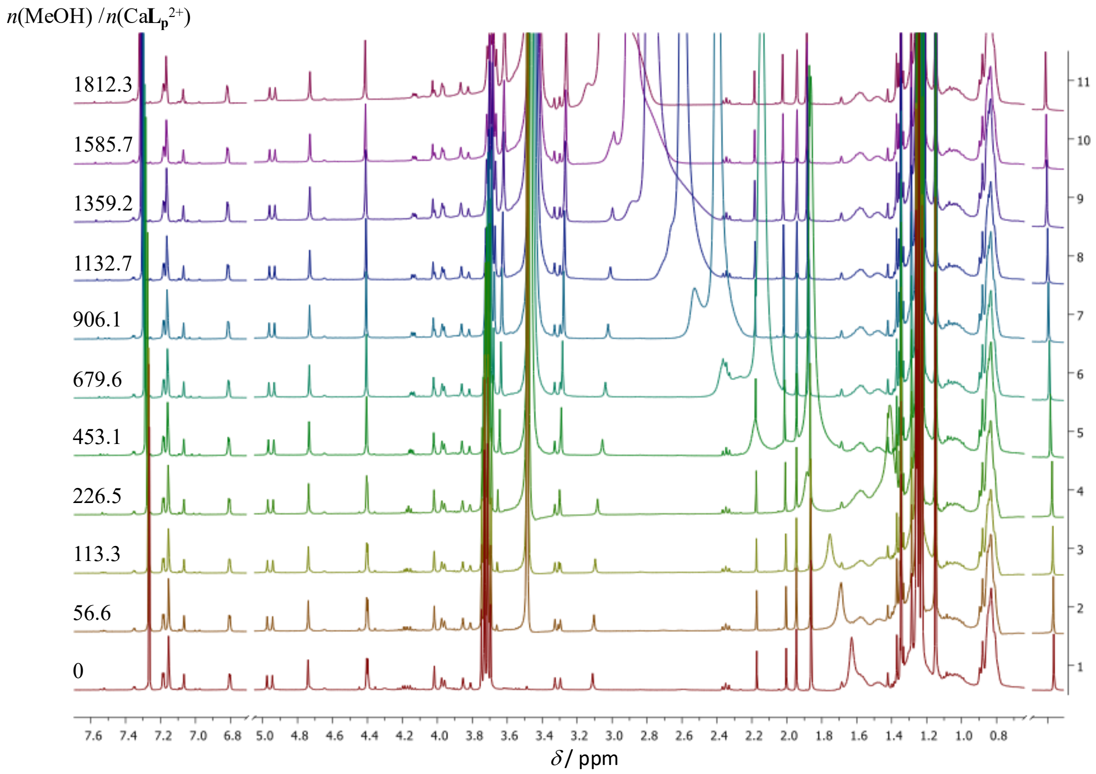

Figure S84. ^{1}H NMR titration of Ca$\mathbf{L_p}^{2+}$ ($c_0 = 1.06 \times 10^{-3}$ mol dm^{-3}) with acetonitrile solution ($c = 5.94$ mol dm^{-3}) at 25 °C. b) ^{1}H NMR titration of Ca$\mathbf{L_p}^{2+}$ ($c_0 = 1.06 \times 10^{-3}$ mol dm^{-3}) with methanol solution ($c = 6.01$ mol dm^{-3}) at 25 °C.

Table S15. Changes in chemical shifts of aromatic (Ar-H) and *tert*-butyl (tb-H) proton signals of $\mathbf{L_p}$, Na$\mathbf{L_p}^+$, Ca$\mathbf{L_p}^{2+}$ and in $CDCl_3$ after addition of acetonitrile or methanol at 25 °C.

Host (H)	Guest (G)	n(G) / n(H)	$\Delta\delta$(Ar-H) / ppm			$\Delta\delta$(tb-H) / ppm		
			7.27 ppm	7.19 ppm	6.81 ppm	1.37 ppm	1.22 ppm	1.14 ppm
$\mathbf{L_p}$	MeCN	314	−0.0017	0.0118	0.0089	0.0013	0.0042	−0.0038
Na$\mathbf{L_p}^+$		316	0.0153	0.0344	0.0322	−0.0011	0.0094	0.0042
Ca$\mathbf{L_p}^{2+}$		1788	−0.0007	0.0138	0.0099	0.0021	−0.0072	−0.0034
$\mathbf{L_p}$	MeOH	319	−0.0039	0.0134	0.0129	0.0011	0.0068	−0.0030
Na$\mathbf{L_p}^+$		320	0.0163	0.0254	0.0194	−0.0036	0.0081	0.0041
Ca$\mathbf{L_p}^{2+}$		1812	−0.0027	0.0322	0.0131	0.0015	−0.0003	−0.0024

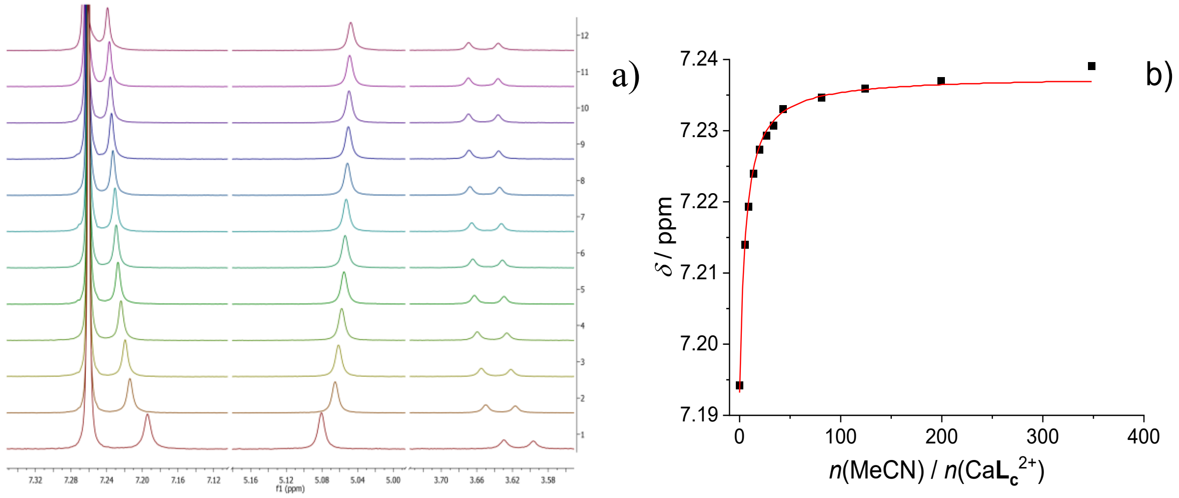

Figure S85. a) ^{1}H NMR titration of Ca$\mathbf{L_c}^{2+}$ (c = 5.13 × 10^{-4} mol dm^{-3}, V_0 = 0.5 mL) with MeCN solution (c = 0.893 mol dm^{-3}) in $CDCl_3$ at 25.0 °C. b) Dependence of chemical shift of aromatic protons on n(MeCN) / n(Ca$\mathbf{L_c}^{2+}$) ratio. ■ experimental; — calculated.

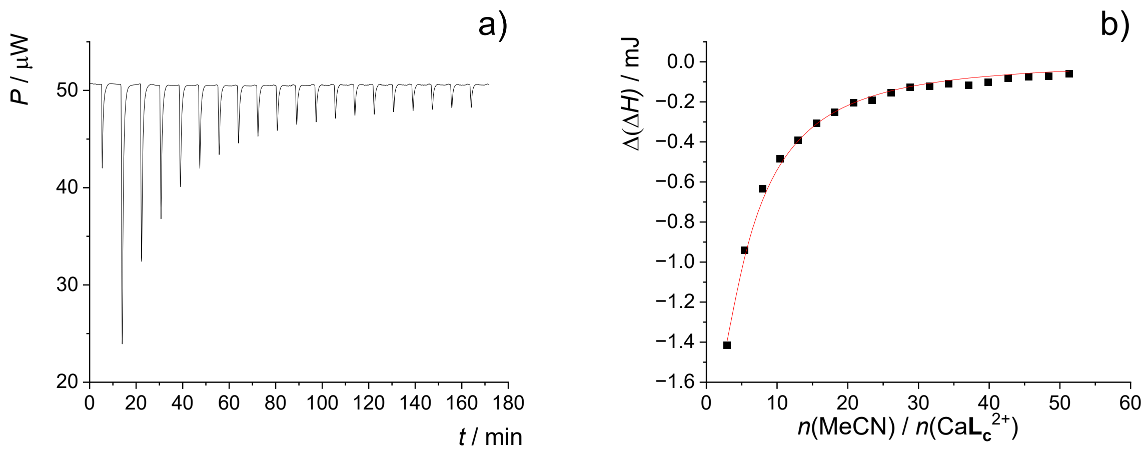

Figure S86. a) Microcalorimetric titration of Ca**L**$_c$ (c = 1.42×10^{-4} mol dm^{-3}, V = 1.43 cm^3) with MeCN solution (c = 3.35×10^{-2} mol dm^{-3}) in $CDCl_3$ at 25 °C. b) Dependence of successive enthalpy changes on n(MeCN) / n(Ca**L**$_c$$^{2+}$) ratio. ■ experimental; — calculated.

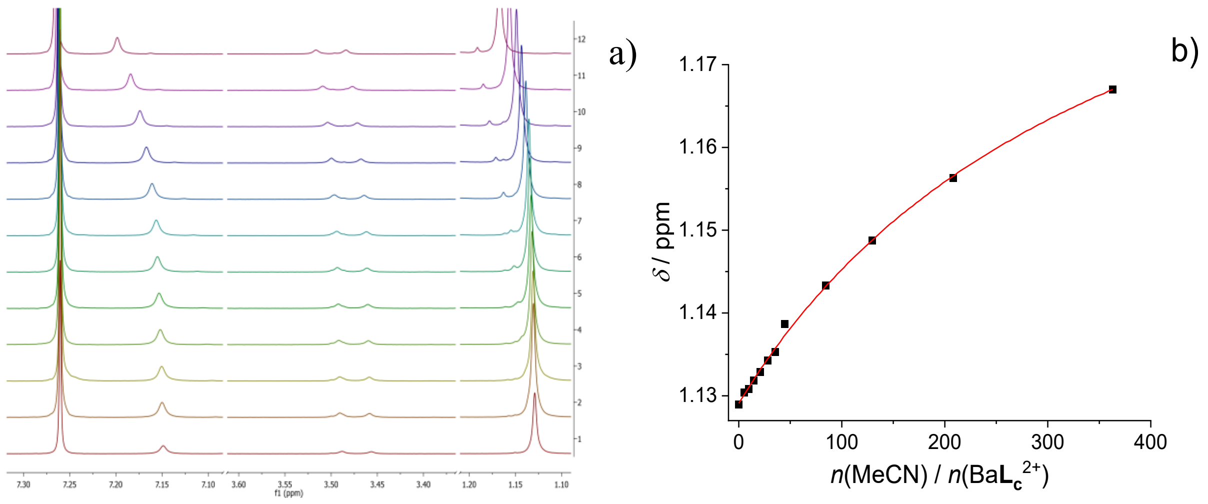

Figure S87. a) ^{1}H NMR titration of Ba**L**$_c$$^{2+}$ (c = 4.93 × 10^{-4} mol dm^{-3}, V_0 = 0.5 mL) with MeCN solution (c = 0.893 mol dm^{-3}) in $CDCl_3$ at 25.0 °C. b) Dependence of chemical shift of *tert*-butyl protons on n(MeCN) / n(Ba**L**$_c$$^{2+}$) ratio. ■ experimental; — calculated.

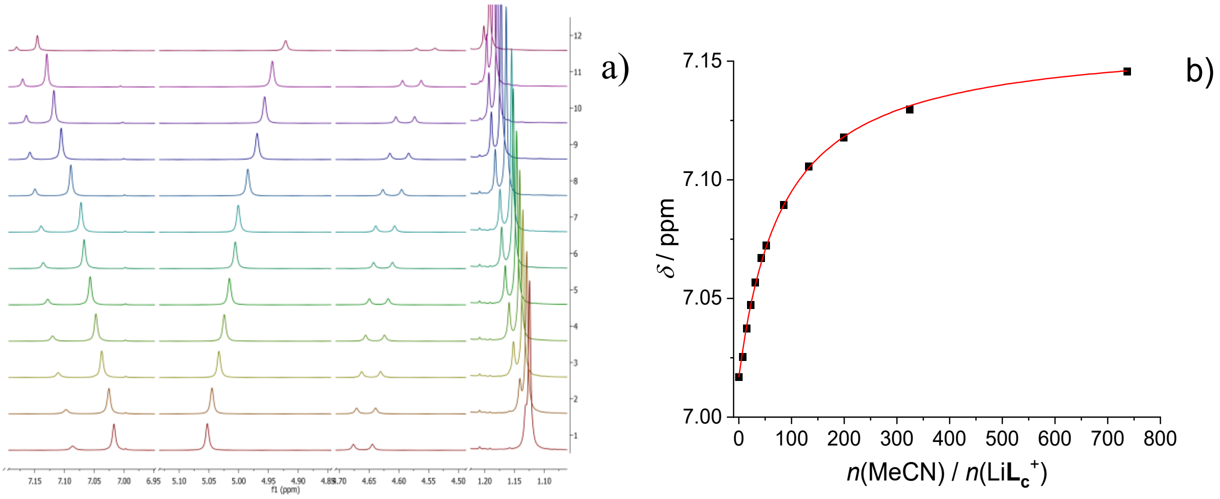

Figure S88. a) ^{1}H NMR titration of Li$\mathbf{L_c}^{+}$ (c = 8.01 × 10^{-4} mol dm^{-3}, V_0 = 0.5 mL) with MeCN solution (c = 2.949 mol dm^{-3}) in $CDCl_3$ at 25.0 °C. b) Dependence of chemical shift of aromatic protons on n(MeCN) / n(Li$\mathbf{L_c}^{+}$) ratio. ■ experimental; — calculated.

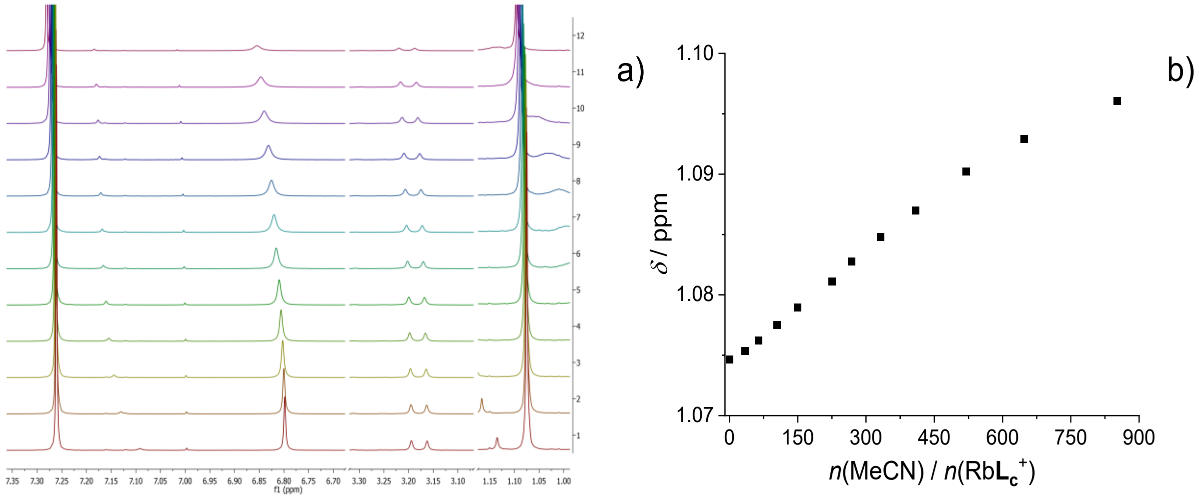

Figure S89. a) ^{1}H NMR titration of Rb$\mathbf{L_c}^{+}$ (c = 6.92 × 10^{-4} mol dm^{-3}, V_0 = 0.5 mL) with MeCN solution (c = 2.95 mol dm^{-3}) in $CDCl_3$ at 25.0 °C. b) Dependence of chemical shift of *tert*-butyl protons on n(MeCN) / n(Rb$\mathbf{L_c}^{+}$) ratio. ■ experimental.

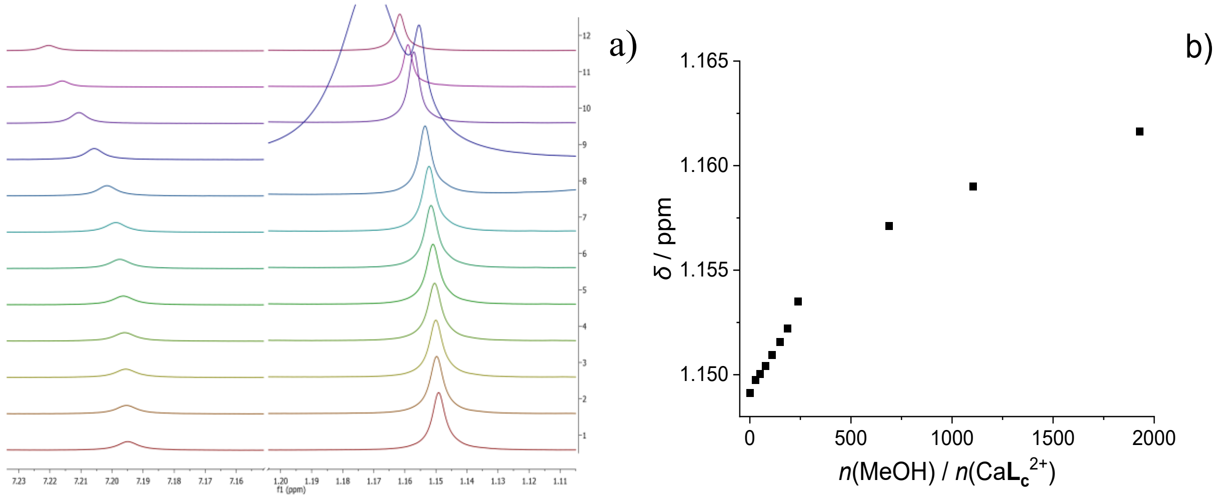

Figure S90. a) ^{1}H NMR titration of Ca$\mathbf{L}_{c}^{2+}$ (c = 5.13 × 10^{-4} mol dm^{-3}, V_0 = 0.5 mL) with MeOH solution (c = 4.94 mol dm^{-3}) in $CDCl_3$ at 25.0 °C. b) Dependence of chemical shift of *tert*-butyl protons on n(MeOH) / n(Ca$\mathbf{L}_{c}^{2+}$) ratio. ■ experimental; — calculated.

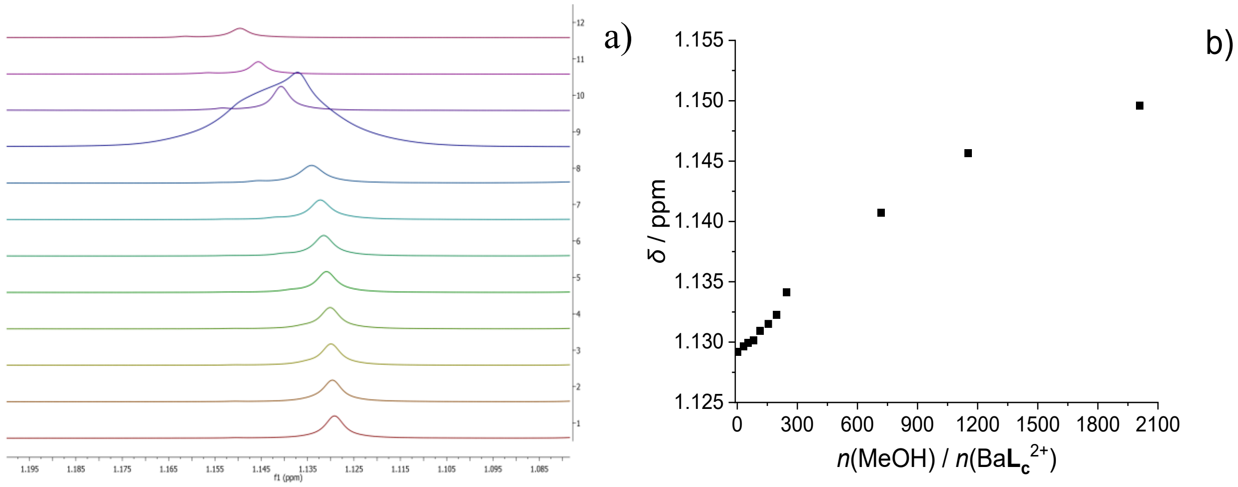

Figure S91. a) ^{1}H NMR titration of Ba$\mathbf{L}_{c}^{2+}$ (c = 4.92 × 10^{-4} mol dm^{-3}, V_0 = 0.5 mL) with MeOH solution (c = 4.94 mol dm^{-3}) in $CDCl_3$ at 25.0 °C. b) Dependence of chemical shift of *tert*-butyl protons on n(MeOH) / n(Ba$\mathbf{L}_{c}^{2+}$) ratio. ■ experimental; — calculated.

S2.9. Solvent effect on the thermodynamics of cation complexation

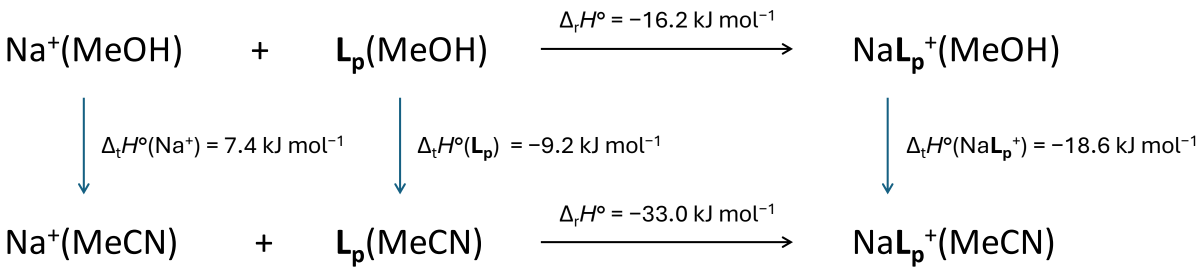

Scheme S1. Thermodynamic cycle explaining the difference in $\Delta_r H°$ for complexation of Na^+ with $\mathbf{L_p}$ in MeOH and MeCN.

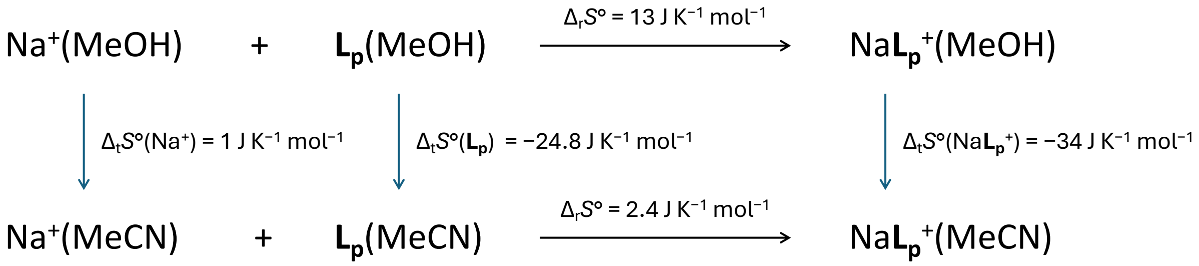

Scheme S2. Thermodynamic cycle explaining the difference in $\Delta_r S°$ for complexation of Na^+ with $\mathbf{L_p}$ in MeOH and MeCN.

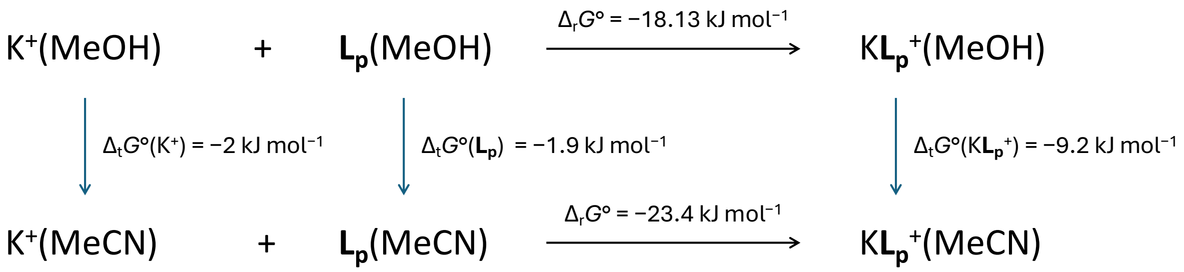

Scheme S3. Thermodynamic cycle explaining the difference in $\Delta_r G°$ for complexation of Na^+ with $\mathbf{L_p}$ in MeOH and MeCN.

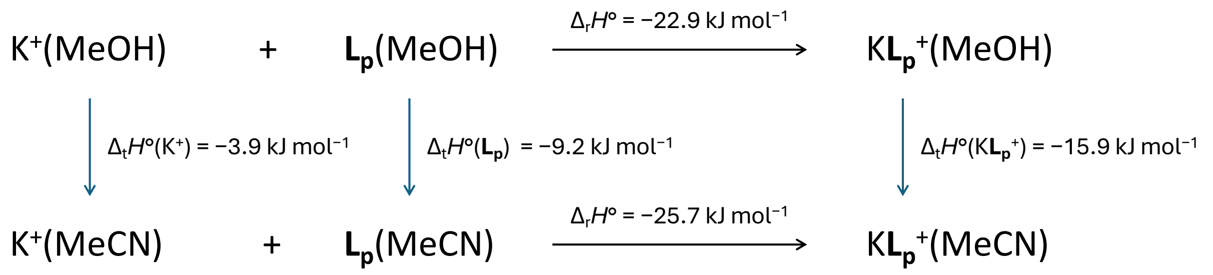

Scheme S4. Thermodynamic cycle explaining the difference in $\Delta_r H°$ for complexation of K^+ with $\mathbf{L_p}$ in MeOH and MeCN.

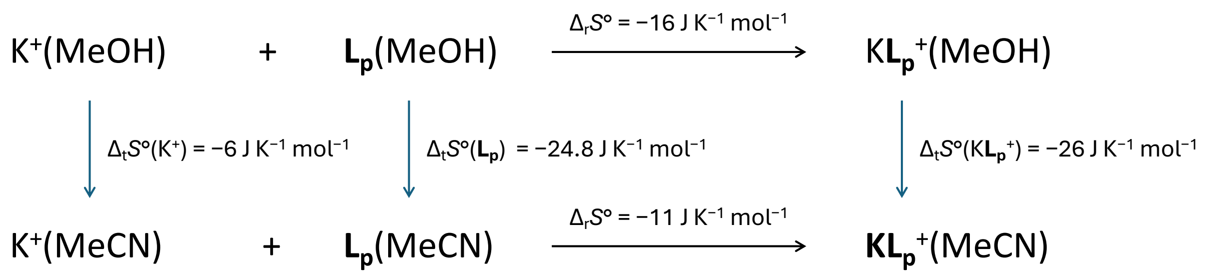

Scheme S5. Thermodynamic cycle explaining the difference in $\Delta_r S°$ for complexation of K^+ with $\mathbf{L_p}$ in MeOH and MeCN.

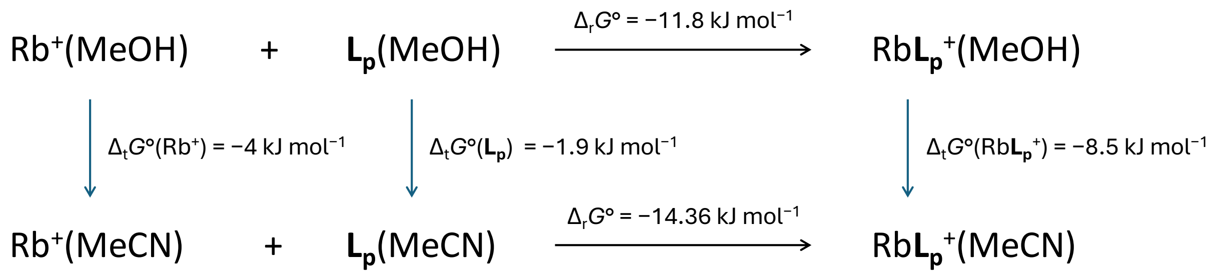

Scheme S6. Thermodynamic cycle explaining the difference in $\Delta_r G°$ for complexation of Rb^+ with $\mathbf{L_p}$ in MeOH and MeCN.

S2.10. Quantum chemical studies

Table S16. Standard enthalpies, entropies, and Gibbs energies of the reactions $M^{+} + \mathbf{L_c}MeCN \rightleftharpoons M\mathbf{L_c}MeCN^{+}$ and $M^{+} + \mathbf{L_c}MeCN' \rightleftharpoons M\mathbf{L_c}MeCN'^{+}$ calculated at the B3LYP-D3BJ/def2SVP level of the theory.

Cation	$M\mathbf{L_c}MeCN^{+}$			$M\mathbf{L_c}MeCN'^{+}$		
	Δ_rH° / kJ mol^{-1}	Δ_rS° / J K^{-1} mol^{-1}	Δ_rG° / kJ mol^{-1}	Δ_rH° / kJ mol^{-1}	Δ_rS° / J K^{-1} mol^{-1}	Δ_rG° / kJ mol^{-1}
Li^{+}	–536.57	–92.72	–508.92	–543.89	–93.33	–516.59
Na^{+}	–474.55	–92.08	–447.10	–481.03	–93.86	–453.05
K^{+}	–346.90	–73.44	–325.00	–345.48	–71.69	–324.10
Rb^{+}	–285.30	–24.63	–277.95	–280.05	–25.07	–278.48
Cs^{+}	–226.42	–12.37	–222.73	–215.95	–8.94	–213.28

Table S17. Standard enthalpies, entropies, and Gibbs energies of the reactions $M\mathbf{L_c}^{+} + MeCN \rightleftharpoons M\mathbf{L_c}MeCN^{+}$ and $M\mathbf{L_c}^{+} + MeCN \rightleftharpoons M\mathbf{L_c}MeCN'^{+}$ calculated at the B3LYP-D3BJ/def2SVP level of the theory.

Cation	$M\mathbf{L_c}MeCN^{+}$			$M\mathbf{L_c}MeCN'^{+}$		
	Δ_rH° / kJ mol^{-1}	Δ_rS° / J K^{-1} mol^{-1}	Δ_rG° / kJ mol^{-1}	Δ_rH° / kJ mol^{-1}	Δ_rS° / J K^{-1} mol^{-1}	Δ_rG° / kJ mol^{-1}
No cation	–69.33	–196.98	–10.61	–[a]	–[a]	–[a]
Li^{+}	–75.06	–182.45	–20.67	–82.38	–183.05	–27.81
Na^{+}	–77.00	–175.65	–24.63	–82.76	–176.80	–30.05
K^{+}	–77.14	–171.14	–26.11	–75.00	–168.75	–24.69
Rb^{+}	–76.64	–172.86	–25.11	–70.68	–172.66	–19.20
Cs^{+}	–76.79	–171.67	–25.79	–65.78	–167.60	–15.81

[a] not observed

Table S18. Standard enthalpies, entropies, and Gibbs energies of the reactions $M^{+} + \mathbf{L_c}MeOH \rightleftharpoons M\mathbf{L_c}MeOH^{+}$ and $M^{+} + \mathbf{L_c}MeOH' \rightleftharpoons M\mathbf{L_c}MeOH'^{+}$ calculated at the B3LYP-D3BJ/def2SVP level of the theory

Cation	$M\mathbf{L_c}MeOH^{+}$			$M\mathbf{L_c}MeOH'^{+}$		
	Δ_rH° / kJ mol^{-1}	Δ_rS° / J K^{-1} mol^{-1}	Δ_rG° / kJ mol^{-1}	Δ_rH° / kJ mol^{-1}	Δ_rS° / J K^{-1} mol^{-1}	Δ_rG° / kJ mol^{-1}
Li^{+}	−548.25	−110.72	−515.24	−566.27	−131.11	−527.18
Na^{+}	−484.95	−114.25	−450.89	−505.01	−130.65	−466.05
K^{+}	−355.71	−94.95	−327.40	−373.37	−99.68	−343.65
Rb^{+}	−293.98	−49.81	−279.13	−309.10	−42.75	−296.35
Cs^{+}	−235.63	−43.87	−222.55	−249.01	−57.85	−231.76

Table S19. Standard enthalpies, entropies, and Gibbs energies of the reactions $M\mathbf{L_c}^{+} + MeOH \rightleftharpoons M\mathbf{L_c}MeOH^{+}$ and $M\mathbf{L_c}^{+} + MeOH \rightleftharpoons M\mathbf{L_c}MeOH'^{+}$ calculated at the B3LYP-D3BJ/def2SVP level of the theory.

Cation	$M\mathbf{L_c}MeOH^{+}$			$M\mathbf{L_c}MeOH'^{+}$		
	Δ_rH° / kJ mol^{-1}	Δ_rS° / J K^{-1} mol^{-1}	Δ_rG° / kJ mol^{-1}	Δ_rH° / kJ mol^{-1}	Δ_rS° / J K^{-1} mol^{-1}	Δ_rG° / kJ mol^{-1}
No cation	−60.93	−176.28	−8.37	−45.12	−156.85	1.65
Li^{+}	−78.34	−179.75	−24.75	−80.55	−180.71	−26.67
Na^{+}	−79.00	−177.13	−26.19	−83.24	−174.09	−31.33
K^{+}	−77.55	−171.95	−26.28	−79.39	−157.24	−32.51
Rb^{+}	−76.92	−177.34	−24.05	−76.23	−150.85	−31.25
Cs^{+}	−77.77	−182.47	−23.37	−75.34	−177.01	−22.57

Table S20. Standard enthalpies, entropies, and Gibbs energies of the reactions $M^{2+} + \mathbf{L_c}MeCN \rightleftharpoons M\mathbf{L_c}MeCN^{2+}$ and $M^{2+} + \mathbf{L_c}MeCN' \rightleftharpoons M\mathbf{L_c}MeCN'^{2+}$ calculated at the B3LYP-D3BJ/def2SVP level of the theory

Cation	$M\mathbf{L_c}MeCN^{2+}$			$M\mathbf{L_c}MeCN'^{2+}$		
	$\Delta_r H^\circ$ / kJ mol^{-1}	$\Delta_r S^\circ$ / J K^{-1} mol^{-1}	$\Delta_r G^\circ$ / kJ mol^{-1}	$\Delta_r H^\circ$ / kJ mol^{-1}	$\Delta_r S^\circ$ / J K^{-1} mol^{-1}	$\Delta_r G^\circ$ / kJ mol^{-1}
Mg^{2+}	−1536.93	−110.36	−1504.02	−1618.47	−136.56	−1577.76
Ca^{2+}	−1303.04	−99.41	−1273.40	−1366.61	−114.47	−1332.48
Sr^{2+}	−1151.36	−82.55	−1126.75	−1211.83	−91.93	−1184.42
Ba^{2+}	−978.30	−59.24	−960.64	−1031.59	−73.80	−1009.59

Table S21. Standard enthalpies, entropies, and Gibbs energies of the reactions $M\mathbf{L_c}^{2+} + MeCN \rightleftharpoons M\mathbf{L_c}MeCN^{2+}$ and $M\mathbf{L_c}^{2+} + MeCN \rightleftharpoons M\mathbf{L_c}MeCN'^{2+}$ calculated at the B3LYP-D3BJ/def2SVP level of the theory

Cation	$M\mathbf{L_c}MeCN^{2+}$			$M\mathbf{L_c}MeCN'^{2+}$		
	$\Delta_r H^\circ$ / kJ mol^{-1}	$\Delta_r S^\circ$ / J K^{-1} mol^{-1}	$\Delta_r G^\circ$ / kJ mol^{-1}	$\Delta_r H^\circ$ / kJ mol^{-1}	$\Delta_r S^\circ$ / J K^{-1} mol^{-1}	$\Delta_r G^\circ$ / kJ mol^{-1}
Mg^{2+}	−61.64	−167.27	−11.77	−142.47	−192.84	−84.97
Ca^{2+}	−64.71	−171.97	−13.44	−127.56	−186.39	−71.99
Sr^{2+}	−64.04	−169.14	−13.61	−123.79	−177.88	−70.75
Ba^{2+}	−64.39	−167.65	−14.40	−116.96	−181.58	−62.82

Table S22. Standard enthalpies, entropies, and Gibbs energies of the reactions $M^{2+} + \mathbf{L_c}MeOH \rightleftharpoons M\mathbf{L_c}MeOH^{2+}$ and $M^{2+} + \mathbf{L_c}MeOH' \rightleftharpoons M\mathbf{L_c}MeOH'^{2+}$ calculated at the B3LYP-D3BJ/def2SVP level of the theory

Cation	$M\mathbf{L_c}MeOH^{2+}$			$M\mathbf{L_c}MeOH'^{2+}$		
	$\Delta_r H^\circ$ / kJ mol^{-1}	$\Delta_r S^\circ$ / J K^{-1} mol^{-1}	$\Delta_r G^\circ$ / kJ mol^{-1}	$\Delta_r H^\circ$ / kJ mol^{-1}	$\Delta_r S^\circ$ / J K^{-1} mol^{-1}	$\Delta_r G^\circ$ / kJ mol^{-1}
Mg^{2+}	−1556.38	−133.79	−1516.49	−1618.77	−172.57	−1567.32
Ca^{2+}	−1319.40	−111.62	−1286.13	−1371.53	−151.24	−1326.44
Sr^{2+}	−1167.52	−93.73	−1139.57	−1220.18	−133.99	−1180.24
Ba^{2+}	−993.98	−74.50	−971.76	−1042.84	−109.16	−1010.29

Table S23. Standard enthalpies, entropies, and Gibbs energies of the reactions $M\mathbf{L_c}^{2+} + MeOH \rightleftharpoons M\mathbf{L_c}MeOH^{2+}$ and $M\mathbf{L_c}^{2+} + MeOH \rightleftharpoons M\mathbf{L_c}MeOH'^{2+}$ calculated at the B3LYP-D3BJ/def2SVP level of the theory

Cation	$M\mathbf{L_c}MeOH^{2+}$			$M\mathbf{L_c}MeOH'^{2+}$		
	$\Delta_r H^\circ$ / kJ mol^{-1}	$\Delta_r S^\circ$ / J K^{-1} mol^{-1}	$\Delta_r G^\circ$ / kJ mol^{-1}	$\Delta_r H^\circ$ / kJ mol^{-1}	$\Delta_r S^\circ$ / J K^{-1} mol^{-1}	$\Delta_r G^\circ$ / kJ mol^{-1}
Mg^{2+}	−72.69	−170.01	−22.00	−119.27	−189.35	−62.81
Ca^{2+}	−72.67	−163.48	−23.93	−108.99	−183.66	−54.23
Sr^{2+}	−71.79	−159.63	−24.20	−108.65	−180.45	−54.85
Ba^{2+}	−71.65	−162.22	−23.29	−104.70	−177.44	−51.80

Table S24. Interatomic distances in complexes of calixarene $\mathbf{L_c}$ and cations calculated at the B3LYP-D3BJ/def2SVP level of the theory.

Cation	*r* / Å (plane[a] to cation)				
	$\mathbf{ML_c}$*$^{+}$	$\mathbf{ML_c}$MeCN^{+}	$\mathbf{ML_c}$MeCN'$^{+}$	$\mathbf{ML_c}$MeOH^{+}	$\mathbf{ML_c}$MeOH'$^{+}$
Li^{+}	0.612	0.627	0.148	0.611	0.162
Na^{+}	0.707	0.711	0.446	0.691	0.457
K^{+}	1.101	1.109	0.933	1.100	0.957
Rb^{+}	1.380	1.400	1.225	1.387	1.266
Cs^{+}	1.735	1.747	1.575	1.760	1.649
	$\mathbf{ML_c}$*$^{2+}$	$\mathbf{ML_c}$MeCN^{2+}	$\mathbf{ML_c}$MeCN'$^{2+}$	$\mathbf{ML_c}$MeOH^{+}	$\mathbf{ML_c}$MeOH'$^{+}$
Mg^{2+}	0.637	0.650	0.361	0.627	0.333
Ca^{2+}	0.895	0.903	0.720	0.887	0.673
Sr^{2+}	0.952	0.961	0.814	0.946	0.759
Ba^{2+}	1.103	1.119	1.026	1.099	0.947
	r / Å (plane[a] to solvent[b])				
	$\mathbf{ML_c}$*$^{+}$	$\mathbf{ML_c}$MeCN^{+}	$\mathbf{ML_c}$MeCN'$^{+}$	$\mathbf{ML_c}$MeOH^{+}	$\mathbf{ML_c}$MeOH'$^{+}$
Li^{+}	–	4.807	3.343	4.350	2.757
Na^{+}	–	4.828	3.452	4.348	2.937
K^{+}	–	4.851	3.366	4.392	2.931
Rb^{+}	–	4.877	3.275	4.421	2.898
Cs^{+}	–	4.889	3.154	4.430	2.982
	$\mathbf{ML_c}$*$^{2+}$	$\mathbf{ML_c}$MeCN^{2+}	$\mathbf{ML_c}$MeCN'$^{2+}$	$\mathbf{ML_c}$MeOH^{+}	$\mathbf{ML_c}$MeOH'$^{+}$
Mg^{2+}	–	4.803	3.157	4.148	2.539
Ca^{2+}	–	4.866	3.117	4.254	2.543
Sr^{2+}	–	4.885	3.193	4.393	2.616
Ba^{2+}	–	4.901	3.182	4.425	2.631
	r / Å (cation to solvent[b])				
	$\mathbf{ML_c}$*$^{+}$	$\mathbf{ML_c}$MeCN^{+}	$\mathbf{ML_c}$MeCN'$^{+}$	$\mathbf{ML_c}$MeOH^{+}	$\mathbf{ML_c}$MeOH'$^{+}$
Li^{+}	–	5.472	3.494	4.980	2.921
Na^{+}	–	5.572	3.902	5.056	3.394
K^{+}	–	5.988	4.302	5.507	3.889
Rb^{+}	–	6.306	4.502	5.823	4.165
Cs^{+}	–	6.663	4.730	6.205	4.632
	$\mathbf{ML_c}$*$^{2+}$	$\mathbf{ML_c}$MeCN^{2+}	$\mathbf{ML_c}$MeCN'$^{2+}$	$\mathbf{ML_c}$MeOH^{+}	$\mathbf{ML_c}$MeOH'$^{+}$
Mg^{2+}	–	5.496	3.520	4.793	2.872
Ca^{2+}	–	5.807	3.837	5.157	3.216
Sr^{2+}	–	5.880	4.008	5.355	3.375
Ba^{2+}	–	6.051	4.209	5.540	3.577

[a] least-squares-fit plane through the 4 ether oxygen atoms of the calixarene unit; [b] solvent center of mass.

Table S25. Interatomic distances in complexes of calixarene $\mathbf{L_c}$ and alkali metal cations calculated at the B3LYP-D3BJ/def2SVP level of the theory (atoms used in the definition of distances are highlighted in blue).

Cation		*r* / Å				
		$ML_c{*}^{+}$	ML_cMeCN^{+}	$ML_cMeCN'^{+}$	ML_cMeOH^{+}	$ML_cMeOH'^{+}$
Li^+	–O–	2.264	2.195	2.144	2.169	2.109
		2.185	2.285	2.218	2.278	2.495
		2.263	2.177	2.128	2.193	2.093
		2.186	2.281	2.219	2.275	2.068
	C=O	3.288	2.152	2.583	2.160	2.130
		2.163	3.288	3.592	3.278	3.828
		3.285	2.154	2.640	2.151	3.016
		2.160	3.259	3.570	3.293	3.432
Na^+	–O–	2.373	2.345	2.311	2.328	2.313
		2.341	2.381	2.354	2.376	2.384
		2.373	2.337	2.302	2.335	2.288
		2.341	2.379	2.353	2.376	2.320
	C=O	3.042	2.422	2.498	2.417	2.477
		2.419	3.043	3.300	3.059	3.336
		3.044	2.422	2.515	2.417	2.509
		2.419	3.010	3.281	3.058	3.244
K^+	–O–	2.644	2.645	2.622	2.631	2.626
		2.644	2.646	2.624	2.647	2.641
		2.644	2.642	2.617	2.638	2.608
		2.644	2.649	2.621	2.650	2.608
	C=O	2.781	2.778	2.838	2.775	2.814
		2.781	2.783	2.845	2.785	2.841
		2.781	2.773	2.844	2.772	2.826
		2.781	2.769	2.842	2.785	2.812
Rb^+	–O–	2.832	2.840	2.810	2.812	2.836
		2.832	2.827	2.810	2.840	2.812
		2.832	2.847	2.801	2.821	2.798
		2.832	2.840	2.807	2.845	2.797
	C=O	2.943	2.932	2.982	2.946	2.957
		2.943	2.951	2.985	2.937	2.982
		2.943	2.908	2.984	2.949	2.952
		2.943	2.946	2.976	2.941	2.958
Cs^+	–O–	3.039	3.045	3.011	3.021	3.037
		3.039	3.040	3.013	3.061	3.015
		3.039	3.037	3.007	3.009	3.012
		3.039	3.044	3.009	3.078	3.009
	C=O	3.133	3.130	3.182	3.136	3.145
		3.133	3.130	3.183	3.121	3.158
		3.133	3.128	3.176	3.172	3.139
		3.133	3.132	3.176	3.129	3.132

[a] least squares fit plane through the 4 ether oxygen atoms of the calixarene unit.

Table S26. Interatomic distances in complexes of calixarene $\mathbf{L_c}$ and alkaline earth metal cations calculated at the B3LYP-D3BJ/def2SVP level of the theory (atoms used in the definition of distances are highlighted in blue).

Cation		*r* / Å				
		$ML_c{}^{*2+}$	ML_cMeCN^{2+}	$ML_cMeCN'^{2+}$	ML_cMeOH^{2+}	$ML_cMeOH'^{2+}$
Mg^{2+}	–O–	2.220	2.195	2.191	2.182	2.179
		2.188	2.242	2.239	2.235	2.323
		2.220	2.177	2.184	2.177	2.149
		2.188	2.231	2.237	2.219	2.169
	C=O	3.008	2.119	2.207	2.124	2.213
		2.122	3.017	3.279	3.038	3.365
		3.010	2.121	2.210	2.125	2.226
		2.122	2.952	3.270	2.998	3.249
Ca^{2+}	–O–	2.447	2.451	2.452	2.440	2.439
		2.447	2.451	2.451	2.449	2.486
		2.447	2.446	2.449	2.444	2.418
		2.447	2.452	2.450	2.445	2.406
	C=O	2.464	2.463	2.524	2.459	2.531
		2.464	2.465	2.526	2.474	2.541
		2.464	2.459	2.525	2.457	2.547
		2.464	2.458	2.524	2.471	2.522
Sr^{2+}	–O–	2.571	2.572	2.573	2.561	2.560
		2.571	2.574	2.574	2.573	2.597
		2.571	2.568	2.570	2.567	2.544
		2.571	2.575	2.572	2.571	2.533
	C=O	2.601	2.599	2.638	2.596	2.642
		2.601	2.602	2.640	2.606	2.651
		2.601	2.596	2.639	2.595	2.652
		2.601	2.594	2.639	2.605	2.638
Ba^{2+}	–O–	2.749	2.749	2.757	2.735	2.736
		2.749	2.753	2.756	2.750	2.772
		2.749	2.747	2.753	2.744	2.721
		2.749	2.757	2.755	2.749	2.709
	C=O	2.761	2.757	2.789	2.758	2.791
		2.761	2.764	2.789	2.766	2.800
		2.761	2.754	2.789	2.757	2.798
		2.761	2.756	2.789	2.766	2.784

[(a)] least squares fit plane through the 4 ether oxygen atoms of the calixarene unit.

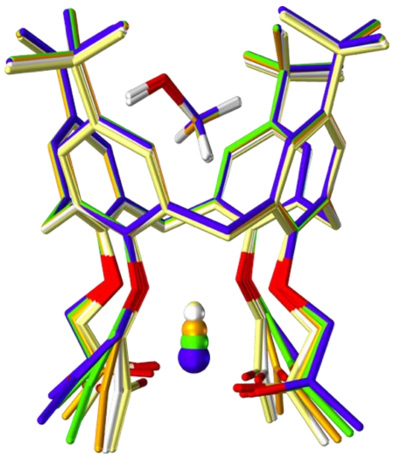

$M\mathbf{L}_{c}MeOH^{+}$

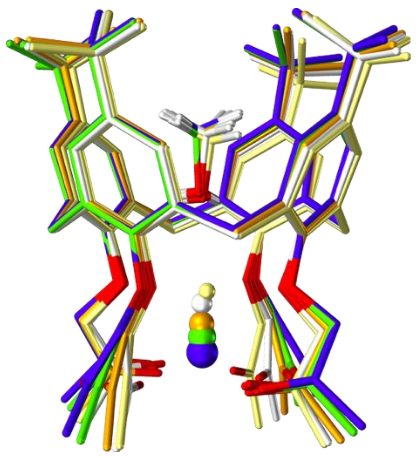

$M\mathbf{L}_{c}MeOH'^{+}$

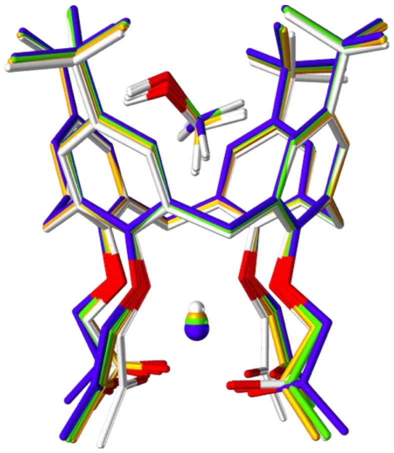

$M\mathbf{L}_{c}MeOH^{2+}$

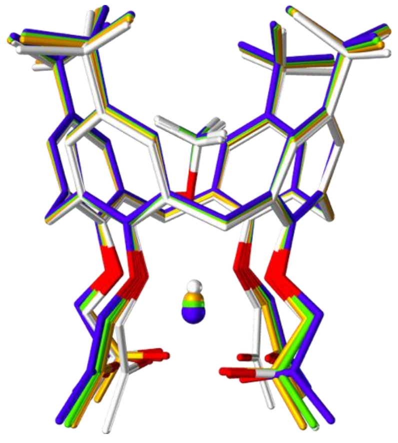

$M\mathbf{L}_{c}MeOH'^{2+}$

Figure S92. a) Optimized geometries of $\mathbf{L}_c$ complexes with alkali and alkaline earth metal cations and the corresponding adducts with MeOH calculated by B3LYPD3BJ/def2SVP method. Li^{+} (orange), Na^{+} and Mg^{2+} (white), K^{+} and Ca^{2+} (orange), Rb^{+} and Sr^{2+} (green), Cs^{+} and Ba^{2+} (blue).

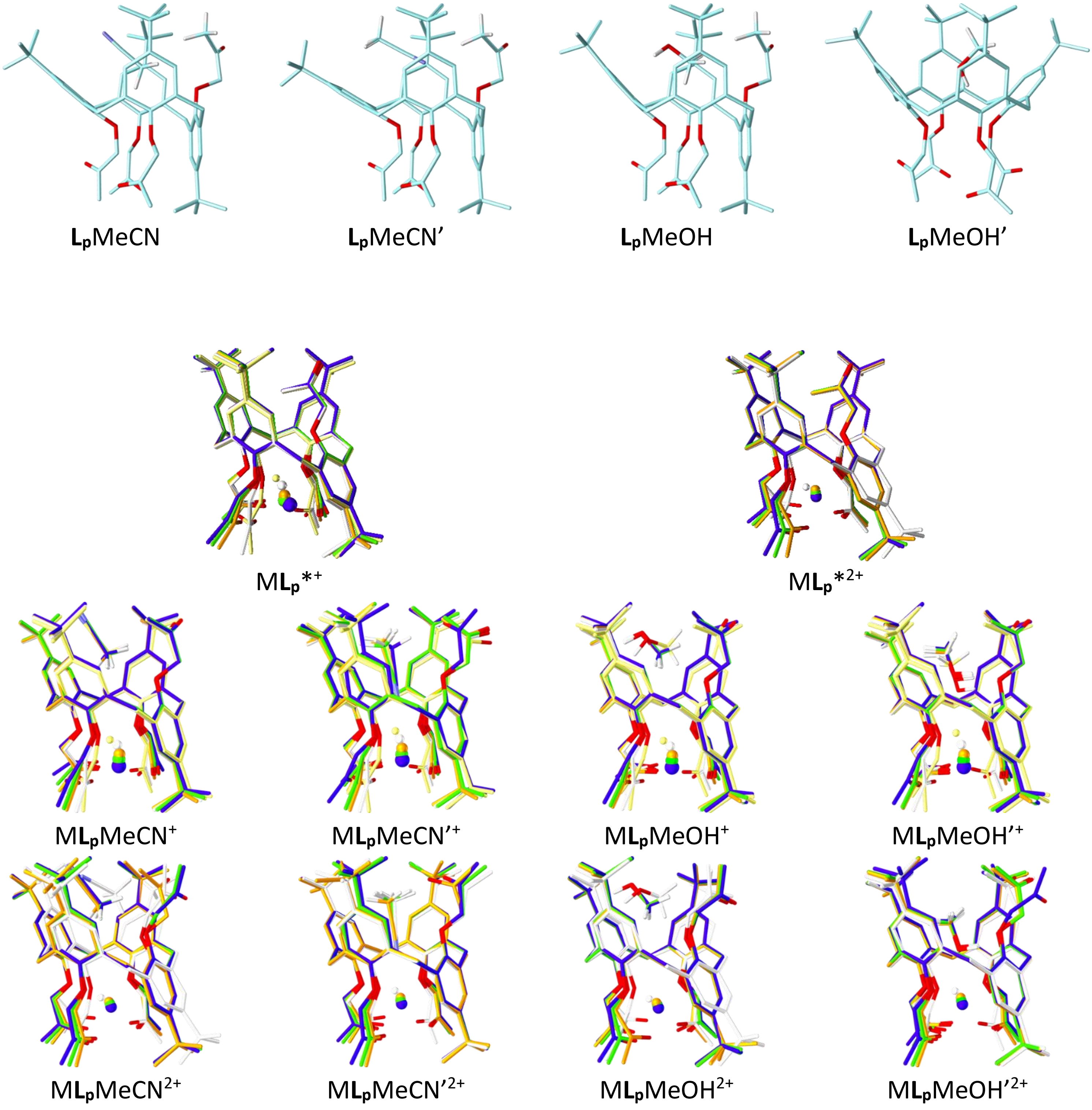

Figure S93. a) Optimized geometries of MeCN and MeOH adducts of $\mathbf{L_p}$ and its complexes with alkali and alkaline earth metal cations calculated by B3LYPD3BJ/def2SVP method. Li^{+} (orange), Na^{+} and Mg^{2+} (white), K^{+} and Ca^{2+} (orange), Rb^{+} and Sr^{2+} (green), Cs^{+} and Ba^{2+} (blue).

Optimized geometries and energies (hartree) calculated at the B3LYP-D3BJ/def2SVP level of the theory

$\mathbf{L_c}$*, zero imaginary frequencies
136
–2777.44868519

C	–0.550258000000	5.836932000000	–2.421769000000
C	–1.404680000000	6.999589000000	–1.876711000000
C	–1.263344000000	5.193191000000	–3.630894000000
C	0.791198000000	6.409524000000	–2.904430000000
C	–0.366238000000	4.772721000000	–1.329455000000
C	–1.491801000000	4.157734000000	–0.760333000000
C	–1.396540000000	3.139578000000	0.193959000000
C	–0.114259000000	2.757446000000	0.631299000000
C	1.035391000000	3.326541000000	0.064897000000
C	0.890982000000	4.328552000000	–0.902535000000
C	2.389939000000	2.765142000000	0.437058000000
O	0.058766000000	1.730080000000	1.519448000000
C	–0.395185000000	1.857358000000	2.840265000000
H	–1.553344000000	7.767793000000	–2.653193000000
H	–0.912836000000	7.474321000000	–1.013296000000
H	–2.397877000000	6.657461000000	–1.549870000000
H	–1.401092000000	5.930396000000	–4.438918000000
H	–2.255721000000	4.803976000000	–3.358380000000
H	–0.673931000000	4.351966000000	–4.028332000000
H	1.350494000000	6.883562000000	–2.083219000000
H	0.614574000000	7.175536000000	–3.675458000000
H	1.430168000000	5.631935000000	–3.350344000000
H	–2.491150000000	4.445403000000	–1.095787000000
H	1.799406000000	4.754912000000	–1.328267000000
H	2.440311000000	2.683182000000	1.526601000000
H	3.173632000000	3.460781000000	0.097986000000
H	–0.896734000000	0.924800000000	3.131157000000
H	–1.166858000000	2.644090000000	2.945221000000
C	0.671502000000	2.209942000000	3.861080000000
O	1.761382000000	2.664724000000	3.574731000000
C	0.277744000000	1.945421000000	5.296204000000
H	0.756236000000	0.992587000000	5.579905000000
H	–0.807235000000	1.837062000000	5.429311000000
H	0.685485000000	2.731895000000	5.946573000000
C	2.764242000000	0.151206000000	–3.860992000000
C	4.161080000000	0.640430000000	–4.298194000000
C	1.692167000000	1.104902000000	–4.428187000000
C	2.524919000000	–1.248913000000	–4.449720000000
C	2.702541000000	0.143051000000	–2.326450000000
C	2.649463000000	1.350879000000	–1.618292000000
C	2.595957000000	1.410949000000	–0.223664000000
C	2.631746000000	0.202633000000	0.496890000000
C	2.674666000000	–1.027862000000	–0.175166000000
C	2.699470000000	–1.035879000000	–1.575583000000
C	2.631746000000	–2.348257000000	0.578772000000
O	2.570614000000	0.191905000000	1.876330000000
C	3.726391000000	0.576072000000	2.561928000000
H	4.238143000000	0.670415000000	–5.397826000000
H	4.947352000000	–0.029333000000	–3.915989000000
H	4.366074000000	1.651662000000	–3.914341000000
H	1.692194000000	1.070343000000	–5.529539000000
H	1.867328000000	2.148918000000	–4.130390000000
H	0.689857000000	0.823582000000	–4.075266000000
H	3.311815000000	–1.958820000000	–4.153248000000
H	2.524892000000	–1.198539000000	–5.549639000000
H	1.557524000000	–1.664245000000	–4.129103000000
H	2.609980000000	2.295632000000	–2.163420000000
H	2.700989000000	–2.004327000000	–2.073928000000
H	2.680150000000	–2.144038000000	1.653330000000
H	3.521312000000	–2.944207000000	0.320211000000
H	4.015143000000	1.619317000000	2.344138000000
H	4.596537000000	–0.059702000000	2.287874000000
C	3.521666000000	0.426347000000	4.060376000000
O	2.653396000000	–0.280877000000	4.526595000000
C	4.458911000000	1.232530000000	4.922426000000
H	4.499404000000	0.813730000000	5.936326000000
H	4.035277000000	2.250413000000	4.966061000000
H	5.468638000000	1.305544000000	4.489549000000
C	0.550258000000	–5.836932000000	–2.421769000000
C	1.404680000000	–6.999589000000	–1.876711000000
C	1.263344000000	–5.193191000000	–3.630894000000
C	–0.791198000000	–6.409524000000	–2.904430000000
C	0.366238000000	–4.772721000000	–1.329455000000
C	1.491801000000	–4.157734000000	–0.760333000000
C	1.396540000000	–3.139578000000	0.193959000000
C	0.114259000000	–2.757446000000	0.631299000000
C	–1.035391000000	–3.326541000000	0.064897000000
C	–0.890982000000	–4.328552000000	–0.902535000000
C	–2.389939000000	–2.765142000000	0.437058000000
O	–0.058766000000	–1.730080000000	1.519448000000
C	0.395185000000	–1.857358000000	2.840265000000
H	1.553344000000	–7.767793000000	–2.653193000000
H	0.912836000000	–7.474321000000	–1.013296000000
H	2.397877000000	–6.657461000000	–1.549870000000
H	1.401092000000	–5.930396000000	–4.438918000000
H	2.255721000000	–4.803976000000	–3.358380000000
H	0.673931000000	–4.351966000000	–4.028332000000
H	–1.350494000000	–6.883562000000	–2.083219000000
H	–0.614574000000	–7.175536000000	–3.675458000000
H	–1.430168000000	–5.631935000000	–3.350344000000
H	2.491150000000	–4.445403000000	–1.095787000000
H	–1.799406000000	–4.754912000000	–1.328267000000
H	–2.440311000000	–2.683182000000	1.526601000000
H	–3.173632000000	–3.460781000000	0.097986000000
H	0.896734000000	–0.924800000000	3.131157000000
H	1.166858000000	–2.644090000000	2.945221000000
C	–0.671502000000	–2.209942000000	3.861080000000
O	–1.761382000000	–2.664724000000	3.574731000000

C	–0.277744000000	–1.945421000000	5.296204000000
H	–0.756236000000	–0.992587000000	5.579905000000
H	0.807235000000	–1.837062000000	5.429311000000
H	–0.685485000000	–2.731895000000	5.946573000000
C	–2.764242000000	–0.151206000000	–3.860992000000
C	–4.161080000000	–0.640430000000	–4.298194000000
C	–1.692167000000	–1.104902000000	–4.428187000000
C	–2.524919000000	1.248913000000	–4.449720000000
C	–2.702541000000	–0.143051000000	–2.326450000000
C	–2.649463000000	–1.350879000000	–1.618292000000
C	–2.595957000000	–1.410949000000	–0.223664000000
C	–2.631746000000	–0.202633000000	0.496890000000
C	–2.674666000000	1.027862000000	–0.175166000000
C	–2.699470000000	1.035879000000	–1.575583000000
C	–2.631746000000	2.348257000000	0.578772000000
O	–2.570614000000	–0.191905000000	1.876330000000
C	–3.726391000000	–0.576072000000	2.561928000000
H	–4.238143000000	–0.670415000000	–5.397826000000
H	–4.947352000000	0.029333000000	–3.915989000000
H	–4.366074000000	–1.651662000000	–3.914341000000
H	–1.692194000000	–1.070343000000	–5.529539000000
H	–1.867328000000	–2.148918000000	–4.130390000000
H	–0.689857000000	–0.823582000000	–4.075266000000
H	–3.311815000000	1.958820000000	–4.153248000000
H	–2.524892000000	1.198539000000	–5.549639000000
H	–1.557524000000	1.664245000000	–4.129103000000
H	–2.609980000000	–2.295632000000	–2.163420000000
H	–2.700989000000	2.004327000000	–2.073928000000
H	–2.680150000000	2.144038000000	1.653330000000
H	–3.521312000000	2.944207000000	0.320211000000
H	–4.015143000000	–1.619317000000	2.344138000000
H	–4.596537000000	0.059702000000	2.287874000000
C	–3.521666000000	–0.426347000000	4.060376000000
O	–2.653396000000	0.280877000000	4.526595000000
C	–4.458911000000	–1.232530000000	4.922426000000
H	–4.499404000000	–0.813730000000	5.936326000000
H	–4.035277000000	–2.250413000000	4.966061000000
H	–5.468638000000	–1.305544000000	4.489549000000

Li**L**$_{c}^{*+}$, zero imaginary frequencies
137
–2784.93402578

Li	2.481814000000	0.000051000000	0.000818000000
C	–3.481851000000	1.324134000000	–3.986091000000
C	–3.522339000000	2.442070000000	–5.048111000000
C	–4.346797000000	1.729419000000	–2.772248000000
C	–4.076282000000	0.046965000000	–4.598929000000
C	–2.036505000000	1.118427000000	–3.512097000000
C	–1.334333000000	2.183894000000	–2.932589000000
C	–0.034230000000	2.050270000000	–2.440127000000
C	0.594139000000	0.799113000000	–2.550839000000
C	–0.071194000000	–0.296010000000	–3.117753000000
C	–1.377154000000	–0.110988000000	–3.592832000000
C	0.530171000000	–1.690631000000	–3.146092000000
O	1.902918000000	0.663995000000	–2.085081000000
C	2.899462000000	0.727010000000	–3.083613000000
H	–4.555236000000	2.603945000000	–5.394385000000
H	–2.906459000000	2.177174000000	–5.921532000000
H	–3.150906000000	3.398689000000	–4.651156000000
H	–5.393294000000	1.881369000000	–3.080308000000
H	–3.993490000000	2.664722000000	–2.312564000000
H	–4.326103000000	0.946898000000	–1.997629000000
H	–3.506393000000	–0.287707000000	–5.479416000000
H	–5.109490000000	0.237075000000	–4.925811000000
H	–4.107334000000	–0.779064000000	–3.871730000000
H	–1.818196000000	3.157031000000	–2.842409000000
H	–1.884985000000	–0.969843000000	–4.027890000000
H	1.620064000000	–1.637260000000	–3.061228000000
H	0.294078000000	–2.163546000000	–4.110505000000
H	2.509468000000	1.233417000000	–3.986727000000
H	3.218335000000	–0.282006000000	–3.395569000000
C	4.118462000000	1.515971000000	–2.633524000000
O	4.063832000000	2.311600000000	–1.720674000000
C	5.382162000000	1.248420000000	–3.406700000000
H	6.075852000000	2.092998000000	–3.307214000000
H	5.184406000000	1.035345000000	–4.468544000000
H	5.847716000000	0.350539000000	–2.966298000000
C	–3.241110000000	–4.564001000000	–1.353927000000
C	–3.143107000000	–5.574863000000	–2.514840000000
C	–4.270491000000	–3.467148000000	–1.704111000000
C	–3.733946000000	–5.306344000000	–0.102443000000
C	–1.874692000000	–3.900023000000	–1.131886000000
C	–1.264092000000	–3.192951000000	–2.179245000000
C	–0.040694000000	–2.537556000000	–2.024747000000
C	0.591861000000	–2.602396000000	–0.774057000000
C	0.016745000000	–3.281172000000	0.303840000000
C	–1.209649000000	–3.929306000000	0.097683000000
C	0.615262000000	–3.203273000000	1.695869000000
O	1.838835000000	–1.993874000000	–0.621418000000
C	2.906180000000	–2.875597000000	–0.904512000000
H	–4.119043000000	–6.055239000000	–2.687145000000
H	–2.409075000000	–6.363661000000	–2.288254000000
H	–2.840066000000	–5.091915000000	–3.455869000000
H	–5.266091000000	–3.911084000000	–1.861732000000
H	–3.995862000000	–2.927485000000	–2.622977000000
H	–4.347738000000	–2.728704000000	–0.890690000000
H	–3.041101000000	–6.107195000000	0.198180000000
H	–4.710075000000	–5.771138000000	–0.306305000000
H	–3.866659000000	–4.624313000000	0.751570000000
H	–1.756534000000	–3.142275000000	–3.151548000000
H	–1.650216000000	–4.461577000000	0.938876000000
H	1.699351000000	–3.056152000000	1.649891000000
H	0.413270000000	–4.142773000000	2.230553000000
H	2.737519000000	–3.397376000000	–1.866993000000
H	3.013266000000	–3.649359000000	–0.126603000000
C	4.191821000000	–2.096768000000	–1.022527000000
O	4.171538000000	–0.878626000000	–1.023698000000
C	5.457927000000	–2.892613000000	–1.129469000000
H	6.259357000000	–2.286325000000	–1.570957000000
H	5.318082000000	–3.826352000000	–1.694475000000
H	5.741781000000	–3.162779000000	–0.098486000000
C	–3.482783000000	–1.326610000000	3.984579000000
C	–3.522827000000	–2.443947000000	5.047246000000
C	–4.346716000000	–1.733415000000	2.770520000000
C	–4.078699000000	–0.049596000000	4.596298000000
C	–2.037353000000	–1.119895000000	3.511278000000
C	–1.334057000000	–2.184971000000	2.932404000000
C	–0.033859000000	–2.050424000000	2.440463000000
C	0.593507000000	–0.798762000000	2.551262000000
C	–0.072906000000	0.295949000000	3.117712000000
C	–1.378997000000	0.110050000000	3.592076000000
C	0.527551000000	1.690950000000	3.146443000000
O	1.902352000000	–0.662726000000	2.085993000000
C	2.898691000000	–0.725208000000	3.084731000000
H	–4.555769000000	–2.606609000000	5.393016000000
H	–2.907709000000	–2.177939000000	5.920867000000
H	–3.150234000000	–3.400440000000	4.651071000000
H	–5.393259000000	–1.886007000000	3.078104000000
H	–3.992420000000	–2.668747000000	2.311667000000
H	–4.326234000000	–0.951405000000	1.995379000000
H	–3.509692000000	0.286047000000	5.476985000000
H	–5.111964000000	–0.240396000000	4.922600000000
H	–4.109946000000	0.775989000000	3.868602000000
H	–1.817128000000	–3.158501000000	2.842242000000
H	–1.887733000000	0.968626000000	4.026628000000
H	1.617519000000	1.638234000000	3.062062000000
H	0.290762000000	2.163590000000	4.110821000000
H	2.508823000000	–1.231902000000	3.987737000000
H	3.216830000000	0.284000000000	3.396927000000
C	4.118276000000	–1.513302000000	2.634747000000
O	4.064390000000	–2.308476000000	1.721471000000
C	5.381559000000	–1.245420000000	3.408524000000
H	6.075828000000	–2.089489000000	3.308746000000
H	5.183298000000	–1.033171000000	4.470441000000
H	5.846806000000	–0.346981000000	2.968932000000

C	–3.244029000000	4.563363000000	1.353312000000
C	–3.145661000000	5.575595000000	2.513013000000
C	–4.273120000000	3.466805000000	1.705223000000
C	–3.737527000000	5.304111000000	0.101147000000
C	–1.877492000000	3.899529000000	1.131535000000
C	–1.266963000000	3.192697000000	2.179100000000
C	–0.043291000000	2.537718000000	2.024986000000
C	0.589614000000	2.602788000000	0.774500000000
C	0.014613000000	3.281358000000	–0.303590000000
C	–1.212093000000	3.929032000000	–0.097846000000
C	0.613722000000	3.203670000000	–1.695368000000
O	1.836770000000	1.994614000000	0.622185000000
C	2.903897000000	2.876556000000	0.905440000000
H	–4.121622000000	6.055924000000	2.685332000000
H	–2.411951000000	6.364312000000	2.285112000000
H	–2.841917000000	5.093749000000	3.454385000000
H	–5.268738000000	3.910814000000	1.862505000000
H	–3.998258000000	2.928417000000	2.624764000000
H	–4.350491000000	2.727268000000	0.892808000000
H	–3.045243000000	6.105079000000	–0.200447000000
H	–4.713887000000	5.768554000000	0.304705000000
H	–3.869956000000	4.621085000000	–0.752121000000
H	–1.759672000000	3.141909000000	3.151268000000
H	–1.652592000000	4.461121000000	–0.939193000000
H	1.697904000000	3.057350000000	–1.649000000000
H	0.411215000000	4.142932000000	–2.230271000000
H	2.735141000000	3.398141000000	1.868000000000
H	3.010868000000	3.650431000000	0.127623000000
C	4.189638000000	2.097880000000	1.023097000000
O	4.169501000000	0.879714000000	1.022809000000
C	5.455637000000	2.893670000000	1.131391000000
H	6.256966000000	2.286910000000	1.572415000000
H	5.315561000000	3.826770000000	1.697387000000
H	5.739820000000	3.164974000000	0.100793000000

Na$\mathbf{L_c}$*$^+$, zero imaginary frequencies
137
–2939.66142871

Na	2.575482000000	0.000767000000	–0.000812000000
C	–3.529966000000	1.103250000000	–4.048943000000
C	–3.598831000000	2.222946000000	–5.107454000000
C	–4.396749000000	1.487268000000	–2.829427000000
C	–4.101759000000	–0.182809000000	–4.664737000000
C	–2.079514000000	0.924027000000	–3.578742000000
C	–1.384170000000	2.013247000000	–3.035028000000
C	–0.078426000000	1.907727000000	–2.549754000000
C	0.559786000000	0.658748000000	–2.641074000000
C	–0.105506000000	–0.466949000000	–3.145637000000
C	–1.415644000000	–0.305423000000	–3.617846000000
C	0.501008000000	–1.861794000000	–3.092082000000
O	1.875318000000	0.551277000000	–2.199815000000
C	2.864768000000	0.625405000000	–3.201564000000
H	–4.636138000000	2.361071000000	–5.450595000000
H	–2.979275000000	1.975027000000	–5.983249000000
H	–3.250268000000	3.187707000000	–4.709687000000
H	–5.447188000000	1.623814000000	–3.131456000000
H	–4.055317000000	2.425381000000	–2.366615000000
H	–4.359247000000	0.701135000000	–2.059094000000
H	–3.520365000000	–0.509920000000	–5.540531000000
H	–5.135537000000	–0.008022000000	–4.998209000000
H	–4.126991000000	–1.008811000000	–3.937643000000
H	–1.878794000000	2.982556000000	–2.971904000000
H	–1.924602000000	–1.181101000000	–4.015805000000
H	1.590053000000	–1.799863000000	–2.998975000000
H	0.270975000000	–2.390640000000	–4.028764000000
H	2.497671000000	1.201827000000	–4.072278000000
H	3.140718000000	–0.378030000000	–3.570740000000
C	4.112413000000	1.330315000000	–2.694968000000
O	4.088198000000	2.021329000000	–1.698104000000
C	5.360089000000	1.106586000000	–3.504130000000
H	6.078691000000	1.917987000000	–3.331738000000
H	5.148375000000	0.998349000000	–4.578847000000
H	5.801818000000	0.159234000000	–3.151006000000
C	–3.370972000000	–4.448594000000	–1.091792000000
C	–3.362369000000	–5.491481000000	–2.227941000000
C	–4.352070000000	–3.307390000000	–1.439600000000
C	–3.862094000000	–5.134641000000	0.192207000000
C	–1.967272000000	–3.848685000000	–0.927045000000
C	–1.337467000000	–3.235495000000	–2.020717000000
C	–0.077685000000	–2.640622000000	–1.922191000000
C	0.578210000000	–2.683498000000	–0.680544000000
C	–0.029872000000	–3.238162000000	0.451777000000
C	–1.291594000000	–3.830927000000	0.296685000000
C	0.572779000000	–3.087414000000	1.839859000000
O	1.861415000000	–2.150685000000	–0.587080000000
C	2.889589000000	–3.094885000000	–0.789458000000
H	–4.366065000000	–5.926548000000	–2.354718000000
H	–2.660674000000	–6.310184000000	–2.004994000000
H	–3.069627000000	–5.048815000000	–3.191644000000
H	–5.373557000000	–3.701217000000	–1.561014000000
H	–4.073312000000	–2.802603000000	–2.376823000000
H	–4.367891000000	–2.548107000000	–0.641968000000
H	–3.193795000000	–5.953760000000	0.499296000000
H	–4.861008000000	–5.564435000000	0.025304000000
H	–3.947210000000	–4.424882000000	1.029254000000
H	–1.846035000000	–3.208955000000	–2.985249000000
H	–1.753962000000	–4.279319000000	1.173822000000
H	1.655245000000	–2.933617000000	1.777632000000
H	0.385485000000	–4.005201000000	2.416873000000
H	2.671537000000	–3.728940000000	–1.671647000000
H	2.998122000000	–3.769833000000	0.077186000000
C	4.211538000000	–2.401510000000	–1.044170000000
O	4.267218000000	–1.201509000000	–1.243014000000
C	5.430057000000	–3.277386000000	–1.018004000000
H	6.247180000000	–2.816406000000	–1.587728000000
H	5.225808000000	–4.295575000000	–1.381323000000
H	5.732843000000	–3.349427000000	0.040291000000
C	–3.528218000000	–1.104032000000	4.049572000000
C	–3.596496000000	–2.223652000000	5.108201000000
C	–4.395262000000	–1.488359000000	2.830343000000
C	–4.100146000000	0.181939000000	4.665429000000
C	–2.077934000000	–0.924480000000	3.578960000000
C	–1.382514000000	–2.013550000000	3.035065000000
C	–0.076911000000	–1.907777000000	2.549515000000
C	0.561139000000	–0.658672000000	2.640637000000
C	–0.104324000000	0.466899000000	3.145319000000
C	–1.414310000000	0.305108000000	3.617853000000
C	0.501842000000	1.861888000000	3.091628000000
O	1.876550000000	–0.551077000000	2.199178000000
C	2.866065000000	–0.625366000000	3.200921000000
H	–4.633667000000	–2.362003000000	5.451665000000
H	–2.976739000000	–1.975501000000	5.983788000000
H	–3.247807000000	–3.188361000000	4.710417000000
H	–5.445585000000	–1.625097000000	3.132687000000
H	–4.053773000000	–2.426449000000	2.367522000000
H	–4.358144000000	–0.702295000000	2.059920000000
H	–3.518608000000	0.509230000000	5.541061000000
H	–5.133801000000	0.006947000000	4.999178000000
H	–4.125737000000	1.007887000000	3.938290000000
H	–1.877003000000	–2.982933000000	2.972006000000
H	–1.923357000000	1.180682000000	4.015923000000
H	1.590861000000	1.800177000000	2.997987000000
H	0.272149000000	2.390621000000	4.028462000000
H	2.499050000000	–1.202183000000	4.071395000000
H	3.141822000000	0.378026000000	3.570323000000
C	4.113738000000	–1.330077000000	2.694069000000
O	4.089275000000	–2.021506000000	1.697518000000
C	5.361659000000	–1.105628000000	3.502631000000
H	6.080521000000	–1.916795000000	3.330239000000
H	5.150364000000	–0.997028000000	4.577391000000
H	5.802823000000	–0.158209000000	3.148947000000

C	–3.371566000000	4.448091000000	1.093170000000
C	–3.362151000000	5.491496000000	2.228849000000
C	–4.352407000000	3.307054000000	1.442195000000
C	–3.863634000000	5.133523000000	–0.190797000000
C	–1.967915000000	3.848261000000	0.927718000000
C	–1.337487000000	3.235262000000	2.021137000000
C	–0.077582000000	2.640702000000	1.922080000000
C	0.577731000000	2.683805000000	0.680169000000
C	–0.030984000000	3.238174000000	–0.451957000000
C	–1.292814000000	3.830574000000	–0.296348000000
C	0.571143000000	3.087553000000	–1.840297000000
O	1.861037000000	2.151358000000	0.586201000000
C	2.889143000000	3.095767000000	0.788360000000
H	–4.365775000000	5.926557000000	2.356226000000
H	–2.660681000000	6.310139000000	2.004981000000
H	–3.068591000000	5.049251000000	3.192498000000
H	–5.373811000000	3.700945000000	1.564085000000
H	–4.073076000000	2.802737000000	2.379497000000
H	–4.368766000000	2.547371000000	0.644956000000
H	–3.195741000000	5.952700000000	–0.498612000000
H	–4.862550000000	5.563144000000	–0.023458000000
H	–3.949056000000	4.423402000000	–1.027506000000
H	–1.845635000000	3.208646000000	2.985890000000
H	–1.755731000000	4.278730000000	–1.173315000000
H	1.653702000000	2.934173000000	–1.778519000000
H	0.383275000000	4.005269000000	–2.417233000000
H	2.670794000000	3.730224000000	1.670159000000
H	2.997757000000	3.770235000000	–0.078649000000
C	4.211077000000	2.402507000000	1.043569000000
O	4.266687000000	1.202562000000	1.242820000000
C	5.429592000000	3.278333000000	1.017228000000
H	6.246713000000	2.817530000000	1.587092000000
H	5.225345000000	4.296630000000	1.380258000000
H	5.732392000000	3.350109000000	–0.041087000000

$\mathrm{K}\mathbf{L}_c^{*+}$, zero imaginary frequencies
137
–3377.26053404

K	0.000000000000	0.000000000000	2.968742000000
C	–1.640606000000	3.949293000000	–3.550945000000
C	–2.944327000000	4.766653000000	–3.651867000000
C	–1.743812000000	2.693119000000	–4.444046000000
C	–0.484238000000	4.819459000000	–4.067248000000
C	–1.419209000000	3.493103000000	–2.101578000000
C	–2.422989000000	2.776914000000	–1.433933000000
C	–2.268142000000	2.303118000000	–0.128413000000
C	–1.063682000000	2.595096000000	0.537559000000
C	0.000000000000	3.236233000000	–0.114586000000
C	–0.215433000000	3.697855000000	–1.421219000000
C	1.395504000000	3.322934000000	0.494307000000
O	–0.936329000000	2.213971000000	1.868164000000
C	–1.180914000000	3.227848000000	2.815099000000
H	–3.107631000000	5.097625000000	–4.689396000000
H	–2.898981000000	5.660954000000	–3.011129000000
H	–3.824727000000	4.179717000000	–3.350810000000
H	–1.900088000000	2.978822000000	–5.496394000000
H	–2.583077000000	2.049325000000	–4.141142000000
H	–0.822939000000	2.092239000000	–4.382597000000
H	–0.345600000000	5.721214000000	–3.451112000000
H	–0.695388000000	5.146657000000	–5.096230000000
H	0.466945000000	4.266501000000	–4.090107000000
H	–3.360340000000	2.570250000000	–1.949902000000
H	0.597949000000	4.223078000000	–1.916860000000
H	1.351961000000	3.175873000000	1.578653000000
H	1.813833000000	4.322143000000	0.299643000000
H	–1.985402000000	3.907492000000	2.471121000000
H	–0.283045000000	3.850167000000	2.984232000000
C	–1.626896000000	2.640259000000	4.145293000000
O	–1.978688000000	1.479704000000	4.245416000000
C	–1.564144000000	3.574471000000	5.320757000000
H	–2.227077000000	3.225729000000	6.122847000000
H	–1.797700000000	4.613909000000	5.044840000000
H	–0.522043000000	3.551033000000	5.685003000000
C	3.948799000000	1.640892000000	–3.551170000000
C	4.765430000000	2.945067000000	–3.652087000000
C	2.692407000000	1.743515000000	–4.444031000000
C	4.819489000000	0.485050000000	–4.067769000000
C	3.492985000000	1.419060000000	–2.101748000000
C	2.776407000000	2.422383000000	–1.433826000000
C	2.302788000000	2.267110000000	–0.128275000000
C	2.595498000000	1.062675000000	0.537416000000
C	3.237158000000	–0.000536000000	–0.114965000000
C	3.698469000000	0.215271000000	–1.421626000000
C	3.324416000000	–1.396081000000	0.493714000000
O	2.214564000000	0.934655000000	1.867990000000
C	3.228295000000	1.179567000000	2.815000000000
H	5.096119000000	3.108676000000	–4.689658000000
H	5.659875000000	2.900134000000	–3.011522000000
H	4.178072000000	3.825114000000	–3.350820000000
H	2.977832000000	1.900069000000	–5.496413000000
H	2.048213000000	2.582390000000	–4.140906000000
H	2.092039000000	0.822308000000	–4.382577000000
H	5.721436000000	0.346825000000	–3.451822000000
H	5.146375000000	0.696495000000	–5.096789000000
H	4.267033000000	–0.466427000000	–4.090628000000
H	2.569314000000	3.359726000000	–1.949621000000
H	4.223973000000	–0.597815000000	–1.917472000000
H	3.178067000000	–1.352710000000	1.578159000000
H	4.323484000000	–1.814365000000	0.298280000000
H	3.908074000000	1.983842000000	2.470790000000
H	3.850476000000	0.281708000000	2.984625000000
C	2.640481000000	1.626283000000	4.144849000000
O	1.480141000000	1.978969000000	4.244329000000
C	3.574090000000	1.563127000000	5.320756000000
H	3.225366000000	2.226351000000	6.122612000000
H	4.613793000000	1.796051000000	5.045289000000
H	3.549875000000	0.521064000000	5.685056000000
C	1.640606000000	–3.949293000000	–3.550945000000
C	2.944327000000	–4.766653000000	–3.651867000000
C	1.743812000000	–2.693119000000	–4.444046000000
C	0.484238000000	–4.819459000000	–4.067248000000
C	1.419209000000	–3.493103000000	–2.101578000000
C	2.422989000000	–2.776914000000	–1.433933000000
C	2.268142000000	–2.303118000000	–0.128413000000
C	1.063682000000	–2.595096000000	0.537559000000
C	0.000000000000	–3.236233000000	–0.114586000000
C	0.215433000000	–3.697855000000	–1.421219000000
C	–1.395504000000	–3.322934000000	0.494307000000
O	0.936329000000	–2.213971000000	1.868164000000
C	1.180914000000	–3.227848000000	2.815099000000
H	3.107631000000	–5.097625000000	–4.689396000000
H	2.898981000000	–5.660954000000	–3.011129000000
H	3.824727000000	–4.179717000000	–3.350810000000
H	1.900088000000	–2.978822000000	–5.496394000000
H	2.583077000000	–2.049325000000	–4.141142000000
H	0.822939000000	–2.092239000000	–4.382597000000
H	0.345600000000	–5.721214000000	–3.451112000000
H	0.695388000000	–5.146657000000	–5.096230000000
H	–0.466945000000	–4.266501000000	–4.090107000000
H	3.360340000000	–2.570250000000	–1.949902000000
H	–0.597949000000	–4.223078000000	–1.916860000000
H	–1.351961000000	–3.175873000000	1.578653000000
H	–1.813833000000	–4.322143000000	0.299643000000
H	1.985402000000	–3.907492000000	2.471121000000
H	0.283045000000	–3.850167000000	2.984232000000
C	1.626896000000	–2.640259000000	4.145293000000
O	1.978688000000	–1.479704000000	4.245416000000
C	1.564144000000	–3.574471000000	5.320757000000
H	2.227077000000	–3.225729000000	6.122847000000
H	1.797700000000	–4.613909000000	5.044840000000
H	0.522043000000	–3.551033000000	5.685003000000

C	–3.948799000000	–1.640892000000	–3.551170000000
C	–4.765430000000	–2.945067000000	–3.652087000000
C	–2.692407000000	–1.743515000000	–4.444031000000
C	–4.819489000000	–0.485050000000	–4.067769000000
C	–3.492985000000	–1.419060000000	–2.101748000000
C	–2.776407000000	–2.422383000000	–1.433826000000
C	–2.302788000000	–2.267110000000	–0.128275000000
C	–2.595498000000	–1.062675000000	0.537416000000
C	–3.237158000000	0.000536000000	–0.114965000000
C	–3.698469000000	–0.215271000000	–1.421626000000
C	–3.324416000000	1.396081000000	0.493714000000
O	–2.214564000000	–0.934655000000	1.867990000000
C	–3.228295000000	–1.179567000000	2.815000000000
H	–5.096119000000	–3.108676000000	–4.689658000000
H	–5.659875000000	–2.900134000000	–3.011522000000
H	–4.178072000000	–3.825114000000	–3.350820000000
H	–2.977832000000	–1.900069000000	–5.496413000000
H	–2.048213000000	–2.582390000000	–4.140906000000
H	–2.092039000000	–0.822308000000	–4.382577000000
H	–5.721436000000	–0.346825000000	–3.451822000000
H	–5.146375000000	–0.696495000000	–5.096789000000
H	–4.267033000000	0.466427000000	–4.090628000000
H	–2.569314000000	–3.359726000000	–1.949621000000
H	–4.223973000000	0.597815000000	–1.917472000000
H	–3.178067000000	1.352710000000	1.578159000000
H	–4.323484000000	1.814365000000	0.298280000000
H	–3.908074000000	–1.983842000000	2.470790000000
H	–3.850476000000	–0.281708000000	2.984625000000
C	–2.640481000000	–1.626283000000	4.144849000000
O	–1.480141000000	–1.978969000000	4.244329000000
C	–3.574090000000	–1.563127000000	5.320756000000
H	–3.225366000000	–2.226351000000	6.122612000000
H	–4.613793000000	–1.796051000000	5.045289000000
H	–3.549875000000	–0.521064000000	5.685056000000

Rb**L**$_c$$^{*+}$, zero imaginary frequencies
137
–2801.49325634

Rb	0.000000000000	0.000000000000	3.168630000000
C	4.023798000000	–1.354153000000	–3.662637000000
C	5.493294000000	–0.900088000000	–3.773276000000
C	3.134152000000	–0.455283000000	–4.549613000000
C	3.925423000000	–2.798062000000	–4.178830000000
C	3.542771000000	–1.224746000000	–2.210152000000
C	3.668099000000	–0.000079000000	–1.539114000000
C	3.223335000000	0.189846000000	–0.227797000000
C	2.662474000000	–0.913970000000	0.440992000000
C	2.432217000000	–2.131265000000	–0.217254000000
C	2.907437000000	–2.267239000000	–1.529679000000
C	1.580467000000	–3.235954000000	0.398708000000
O	2.345517000000	–0.783373000000	1.788841000000
C	3.337228000000	–1.260570000000	2.669904000000
H	5.843784000000	–0.992893000000	–4.813092000000
H	6.145376000000	–1.517987000000	–3.136559000000
H	5.625549000000	0.150154000000	–3.473774000000
H	3.445711000000	–0.522391000000	–5.604129000000
H	3.196885000000	0.600245000000	–4.245742000000
H	2.078094000000	–0.759941000000	–4.481632000000
H	4.520316000000	–3.491940000000	–3.565100000000
H	4.306671000000	–2.851695000000	–5.209485000000
H	2.885579000000	–3.157511000000	–4.197562000000
H	4.124635000000	0.843276000000	–2.056117000000
H	2.762627000000	–3.222696000000	–2.028486000000
H	1.496091000000	–3.093262000000	1.481191000000
H	2.056776000000	–4.211020000000	0.213855000000
H	4.338155000000	–1.214148000000	2.200387000000
H	3.159898000000	–2.319455000000	2.940569000000
C	3.399382000000	–0.447506000000	3.952577000000
O	2.731746000000	0.556663000000	4.112303000000
C	4.312246000000	–0.994341000000	5.019477000000
H	4.638895000000	–0.186885000000	5.687811000000
H	5.181105000000	–1.522239000000	4.598869000000
H	3.736752000000	–1.724969000000	5.614379000000
C	–1.353982000000	–4.023323000000	–3.662825000000
C	–0.899728000000	–5.492743000000	–3.773686000000
C	–0.455223000000	–3.133430000000	–4.549669000000
C	–2.797904000000	–3.925064000000	–4.179003000000
C	–1.224636000000	–3.542470000000	–2.210276000000
C	0.000000000000	–3.667875000000	–1.539198000000
C	0.189883000000	–3.223225000000	–0.227836000000
C	–0.913952000000	–2.662407000000	0.440960000000
C	–2.131228000000	–2.432104000000	–0.217309000000
C	–2.267151000000	–2.907193000000	–1.529784000000
C	–3.235965000000	–1.580448000000	0.398697000000
O	–0.783391000000	–2.345527000000	1.788828000000
C	–1.260710000000	–3.337229000000	2.669830000000
H	–0.992524000000	–5.843093000000	–4.813550000000
H	–1.517519000000	–6.144999000000	–3.137042000000
H	0.150543000000	–5.624909000000	–3.474246000000
H	–0.522261000000	–3.444860000000	–5.604227000000
H	0.600307000000	–3.196052000000	–4.245775000000
H	–0.760035000000	–2.077426000000	–4.481555000000
H	–3.491708000000	–4.520093000000	–3.565321000000
H	–2.851501000000	–4.306219000000	–5.209693000000
H	–3.157474000000	–2.885261000000	–4.197636000000
H	0.843368000000	–4.124371000000	–2.056211000000
H	–3.222585000000	–2.762321000000	–2.028618000000
H	–3.093261000000	–1.496105000000	1.481181000000
H	–4.211000000000	–2.056818000000	0.213838000000
H	–1.214148000000	–4.338172000000	2.200358000000
H	–2.319650000000	–3.159942000000	2.940296000000
C	–0.447872000000	–3.399297000000	3.952648000000
O	0.556294000000	–2.731686000000	4.112489000000
C	–0.994927000000	–4.312051000000	5.019529000000
H	–0.187610000000	–4.638641000000	5.688061000000
H	–1.522747000000	–5.180947000000	4.598898000000
H	–1.725675000000	–3.736488000000	5.614217000000
C	–4.023798000000	1.354153000000	–3.662637000000
C	–5.493294000000	0.900088000000	–3.773276000000
C	–3.134152000000	0.455283000000	–4.549613000000
C	–3.925423000000	2.798062000000	–4.178830000000
C	–3.542771000000	1.224746000000	–2.210152000000
C	–3.668099000000	0.000079000000	–1.539114000000
C	–3.223335000000	–0.189846000000	–0.227797000000
C	–2.662474000000	0.913970000000	0.440992000000
C	–2.432217000000	2.131265000000	–0.217254000000
C	–2.907437000000	2.267239000000	–1.529679000000
C	–1.580467000000	3.235954000000	0.398708000000
O	–2.345517000000	0.783373000000	1.788841000000
C	–3.337228000000	1.260570000000	2.669904000000
H	–5.843784000000	0.992893000000	–4.813092000000
H	–6.145376000000	1.517987000000	–3.136559000000
H	–5.625549000000	–0.150154000000	–3.473774000000
H	–3.445711000000	0.522391000000	–5.604129000000
H	–3.196885000000	–0.600245000000	–4.245742000000
H	–2.078094000000	0.759941000000	–4.481632000000
H	–4.520316000000	3.491940000000	–3.565100000000
H	–4.306671000000	2.851695000000	–5.209485000000
H	–2.885579000000	3.157511000000	–4.197562000000
H	–4.124635000000	–0.843276000000	–2.056117000000
H	–2.762627000000	3.222696000000	–2.028486000000
H	–1.496091000000	3.093262000000	1.481191000000
H	–2.056776000000	4.211020000000	0.213855000000
H	–4.338155000000	1.214148000000	2.200387000000
H	–3.159898000000	2.319455000000	2.940569000000
C	–3.399382000000	0.447506000000	3.952577000000
O	–2.731746000000	–0.556663000000	4.112303000000
C	–4.312246000000	0.994341000000	5.019477000000
H	–4.638895000000	0.186885000000	5.687811000000
H	–5.181105000000	1.522239000000	4.598869000000
H	–3.736752000000	1.724969000000	5.614379000000

C	1.353982000000	4.023323000000	–3.662825000000
C	0.899728000000	5.492743000000	–3.773686000000
C	0.455223000000	3.133430000000	–4.549669000000
C	2.797904000000	3.925064000000	–4.179003000000
C	1.224636000000	3.542470000000	–2.210276000000
C	0.000000000000	3.667875000000	–1.539198000000
C	–0.189883000000	3.223225000000	–0.227836000000
C	0.913952000000	2.662407000000	0.440960000000
C	2.131228000000	2.432104000000	–0.217309000000
C	2.267151000000	2.907193000000	–1.529784000000
C	3.235965000000	1.580448000000	0.398697000000
O	0.783391000000	2.345527000000	1.788828000000
C	1.260710000000	3.337229000000	2.669830000000
H	0.992524000000	5.843093000000	–4.813550000000
H	1.517519000000	6.144999000000	–3.137042000000
H	–0.150543000000	5.624909000000	–3.474246000000
H	0.522261000000	3.444860000000	–5.604227000000
H	–0.600307000000	3.196052000000	–4.245775000000
H	0.760035000000	2.077426000000	–4.481555000000
H	3.491708000000	4.520093000000	–3.565321000000
H	2.851501000000	4.306219000000	–5.209693000000
H	3.157474000000	2.885261000000	–4.197636000000
H	–0.843368000000	4.124371000000	–2.056211000000
H	3.222585000000	2.762321000000	–2.028618000000
H	3.093261000000	1.496105000000	1.481181000000
H	4.211000000000	2.056818000000	0.213838000000
H	1.214148000000	4.338172000000	2.200358000000
H	2.319650000000	3.159942000000	2.940296000000
C	0.447872000000	3.399297000000	3.952648000000
O	–0.556294000000	2.731686000000	4.112489000000
C	0.994927000000	4.312051000000	5.019529000000
H	0.187610000000	4.638641000000	5.688061000000
H	1.522747000000	5.180947000000	4.598898000000
H	1.725675000000	3.736488000000	5.614217000000

$\mathrm{Cs}\mathbf{L}_c^{*+}$, zero imaginary frequencies
137
–2797.53374583

Cs	0.000000000000	0.000000000000	3.409673000000
C	–4.143525000000	0.888379000000	–3.787104000000
C	–5.549906000000	0.266329000000	–3.899785000000
C	–3.154426000000	0.099859000000	–4.673614000000
C	–4.213566000000	2.334309000000	–4.302332000000
C	–3.652217000000	0.814834000000	–2.334238000000
C	–3.637782000000	–0.415379000000	–1.662456000000
C	–3.177818000000	–0.553168000000	–0.349635000000
C	–2.748141000000	0.607231000000	0.320106000000
C	–2.655784000000	1.842082000000	–0.339803000000
C	–3.140822000000	1.922814000000	–1.653127000000
C	–1.936272000000	3.036669000000	0.276340000000
O	–2.439269000000	0.522112000000	1.674463000000
C	–3.485315000000	0.924669000000	2.531665000000
H	–5.907789000000	0.318401000000	–4.939931000000
H	–6.270227000000	0.803490000000	–3.263344000000
H	–5.559016000000	–0.792382000000	–3.601092000000
H	–3.470696000000	0.130569000000	–5.728447000000
H	–3.093937000000	–0.955831000000	–4.369908000000
H	–2.141183000000	0.525512000000	–4.604441000000
H	–4.885535000000	2.953710000000	–3.688452000000
H	–4.597742000000	2.344075000000	–5.333281000000
H	–3.222706000000	2.812502000000	–4.320016000000
H	–3.995499000000	–1.305324000000	–2.179084000000
H	–3.105362000000	2.888576000000	–2.151751000000
H	–1.836579000000	2.908485000000	1.359020000000
H	–2.518585000000	3.951672000000	0.087575000000
H	–4.449413000000	0.953690000000	1.992338000000
H	–3.305655000000	1.946183000000	2.921279000000
C	–3.646744000000	–0.000702000000	3.727187000000
O	–2.937941000000	–0.975428000000	3.891824000000
C	–4.703346000000	0.408820000000	4.722611000000
H	–5.030065000000	–0.463373000000	5.303827000000
H	–5.564826000000	0.896376000000	4.242689000000
H	–4.256268000000	1.140445000000	5.418166000000
C	0.888027000000	4.142222000000	–3.787644000000
C	0.266270000000	5.548683000000	–3.900921000000
C	0.099200000000	3.152866000000	–4.673606000000
C	2.333913000000	4.211758000000	–4.303078000000
C	0.814614000000	3.651496000000	–2.334544000000
C	–0.415517000000	3.637111000000	–1.662589000000
C	–0.553114000000	3.177459000000	–0.349631000000
C	0.607400000000	2.748040000000	0.320083000000
C	1.842184000000	2.655650000000	–0.339973000000
C	1.922717000000	3.140343000000	–1.653423000000
C	3.036924000000	1.936389000000	0.276161000000
O	0.522446000000	2.439383000000	1.674495000000
C	0.925100000000	3.485527000000	2.531525000000
H	0.318507000000	5.906132000000	–4.941207000000
H	0.803530000000	6.269152000000	–3.264731000000
H	–0.792465000000	5.558152000000	–3.602336000000
H	0.129758000000	3.468697000000	–5.728576000000
H	–0.956436000000	3.092643000000	–4.369655000000
H	0.524729000000	2.139597000000	–4.604090000000
H	2.953493000000	4.883957000000	–3.689632000000
H	2.343632000000	4.595371000000	–5.334235000000
H	2.811943000000	3.220811000000	–4.320269000000
H	–1.305563000000	3.994573000000	–2.179198000000
H	2.888420000000	3.104772000000	–2.152179000000
H	2.908821000000	1.836837000000	1.358861000000
H	3.951837000000	2.518790000000	0.087237000000
H	0.953852000000	4.449600000000	1.992138000000
H	1.946726000000	3.306011000000	2.920901000000
C	0.000000000000	3.646893000000	3.727263000000
O	–0.974639000000	2.938018000000	3.892131000000
C	0.409669000000	4.703546000000	4.722572000000
H	–0.462417000000	5.030218000000	5.303974000000
H	0.897057000000	5.565049000000	4.242520000000
H	1.141481000000	4.256534000000	5.417971000000
C	4.143525000000	–0.888379000000	–3.787104000000
C	5.549906000000	–0.266329000000	–3.899785000000
C	3.154426000000	–0.099859000000	–4.673614000000
C	4.213566000000	–2.334309000000	–4.302332000000
C	3.652217000000	–0.814834000000	–2.334238000000
C	3.637782000000	0.415379000000	–1.662456000000
C	3.177818000000	0.553168000000	–0.349635000000
C	2.748141000000	–0.607231000000	0.320106000000
C	2.655784000000	–1.842082000000	–0.339803000000
C	3.140822000000	–1.922814000000	–1.653127000000
C	1.936272000000	–3.036669000000	0.276340000000
O	2.439269000000	–0.522112000000	1.674463000000
C	3.485315000000	–0.924669000000	2.531665000000
H	5.907789000000	–0.318401000000	–4.939931000000
H	6.270227000000	–0.803490000000	–3.263344000000
H	5.559016000000	0.792382000000	–3.601092000000
H	3.470696000000	–0.130569000000	–5.728447000000
H	3.093937000000	0.955831000000	–4.369908000000
H	2.141183000000	–0.525512000000	–4.604441000000
H	4.885535000000	–2.953710000000	–3.688452000000
H	4.597742000000	–2.344075000000	–5.333281000000
H	3.222706000000	–2.812502000000	–4.320016000000
H	3.995499000000	1.305324000000	–2.179084000000
H	3.105362000000	–2.888576000000	–2.151751000000
H	1.836579000000	–2.908485000000	1.359020000000
H	2.518585000000	–3.951672000000	0.087575000000
H	4.449413000000	–0.953690000000	1.992338000000
H	3.305655000000	–1.946183000000	2.921279000000
C	3.646744000000	0.000702000000	3.727187000000
O	2.937941000000	0.975428000000	3.891824000000
C	4.703346000000	–0.408820000000	4.722611000000
H	5.030065000000	0.463373000000	5.303827000000
H	5.564826000000	–0.896376000000	4.242689000000
H	4.256268000000	–1.140445000000	5.418166000000

C	–0.888027000000	–4.142222000000	–3.787644000000
C	–0.266270000000	–5.548683000000	–3.900921000000
C	–0.099200000000	–3.152866000000	–4.673606000000
C	–2.333913000000	–4.211758000000	–4.303078000000
C	–0.814614000000	–3.651496000000	–2.334544000000
C	0.415517000000	–3.637111000000	–1.662589000000
C	0.553114000000	–3.177459000000	–0.349631000000
C	–0.607400000000	–2.748040000000	0.320083000000
C	–1.842184000000	–2.655650000000	–0.339973000000
C	–1.922717000000	–3.140343000000	–1.653423000000
C	–3.036924000000	–1.936389000000	0.276161000000
O	–0.522446000000	–2.439383000000	1.674495000000
C	–0.925100000000	–3.485527000000	2.531525000000
H	–0.318507000000	–5.906132000000	–4.941207000000
H	–0.803530000000	–6.269152000000	–3.264731000000
H	0.792465000000	–5.558152000000	–3.602336000000
H	–0.129758000000	–3.468697000000	–5.728576000000
H	0.956436000000	–3.092643000000	–4.369655000000
H	–0.524729000000	–2.139597000000	–4.604090000000
H	–2.953493000000	–4.883957000000	–3.689632000000
H	–2.343632000000	–4.595371000000	–5.334235000000
H	–2.811943000000	–3.220811000000	–4.320269000000
H	1.305563000000	–3.994573000000	–2.179198000000
H	–2.888420000000	–3.104772000000	–2.152179000000
H	–2.908821000000	–1.836837000000	1.358861000000
H	–3.951837000000	–2.518790000000	0.087237000000
H	–0.953852000000	–4.449600000000	1.992138000000
H	–1.946726000000	–3.306011000000	2.920901000000
C	0.000000000000	–3.646893000000	3.727263000000
O	0.974639000000	–2.938018000000	3.892131000000
C	–0.409669000000	–4.703546000000	4.722572000000
H	0.462417000000	–5.030218000000	5.303974000000
H	–0.897057000000	–5.565049000000	4.242520000000
H	–1.141481000000	–4.256534000000	5.417971000000

Mg$\mathbf{L_c}^{*2+}$, zero imaginary frequencies
137
–2977.21032455

Mg	2.463962000000	0.000221000000	–0.000501000000
C	–3.550577000000	1.007762000000	–4.014703000000
C	–3.572540000000	1.995922000000	–5.199726000000
C	–4.396137000000	1.570503000000	–2.850615000000
C	–4.172294000000	–0.321417000000	–4.469135000000
C	–2.106351000000	0.830901000000	–3.529382000000
C	–1.389200000000	1.937536000000	–3.052420000000
C	–0.088540000000	1.836490000000	–2.554201000000
C	0.513517000000	0.569802000000	–2.546878000000
C	–0.145446000000	–0.560701000000	–3.039931000000
C	–1.454389000000	–0.405671000000	–3.516768000000
C	0.464347000000	–1.949805000000	–3.007625000000
O	1.849647000000	0.454939000000	–2.084427000000
C	2.813578000000	0.575897000000	–3.131896000000
H	–4.603956000000	2.136109000000	–5.557517000000
H	–2.969213000000	1.619006000000	–6.039846000000
H	–3.182033000000	2.985569000000	–4.918424000000
H	–5.441395000000	1.700625000000	–3.169830000000
H	–4.028581000000	2.550428000000	–2.510088000000
H	–4.384282000000	0.886197000000	–1.987682000000
H	–3.625707000000	–0.762051000000	–5.317068000000
H	–5.207345000000	–0.152305000000	–4.799564000000
H	–4.204181000000	–1.059264000000	–3.652140000000
H	–1.861955000000	2.919878000000	–3.054157000000
H	–1.968226000000	–1.290556000000	–3.887610000000
H	1.557995000000	–1.887916000000	–2.967542000000
H	0.202981000000	–2.476490000000	–3.935782000000
H	2.348945000000	1.084117000000	–3.993930000000
H	3.139407000000	–0.418057000000	–3.482277000000
C	4.020139000000	1.389642000000	–2.708123000000
O	4.023332000000	1.977625000000	–1.645780000000
C	5.174746000000	1.407147000000	–3.665878000000
H	5.795899000000	2.295813000000	–3.494877000000
H	4.849607000000	1.360603000000	–4.716041000000
H	5.790203000000	0.511760000000	–3.469591000000
C	–3.207255000000	–4.806333000000	–1.002786000000
C	–3.036457000000	–5.931089000000	–2.045430000000
C	–4.267272000000	–3.795309000000	–1.493200000000
C	–3.700546000000	–5.426924000000	0.312981000000
C	–1.872615000000	–4.070177000000	–0.828099000000
C	–1.276974000000	–3.432793000000	–1.928904000000
C	–0.077523000000	–2.726034000000	–1.825154000000
C	0.538465000000	–2.667165000000	–0.568781000000
C	–0.011945000000	–3.272732000000	0.560038000000
C	–1.216706000000	–3.973267000000	0.403603000000
C	0.574362000000	–3.056844000000	1.940135000000
O	1.802845000000	–2.032549000000	–0.467379000000
C	2.862548000000	–2.939077000000	–0.761149000000
H	–3.987804000000	–6.466940000000	–2.183861000000
H	–2.279482000000	–6.660742000000	–1.719058000000
H	–2.730769000000	–5.540501000000	–3.027731000000
H	–5.239930000000	–4.295644000000	–1.615614000000
H	–3.997768000000	–3.354272000000	–2.464860000000
H	–4.393484000000	–2.974255000000	–0.769946000000
H	–2.991656000000	–6.170173000000	0.708899000000
H	–4.656351000000	–5.943236000000	0.143063000000
H	–3.874608000000	–4.662906000000	1.086867000000
H	–1.762455000000	–3.482769000000	–2.904446000000
H	–1.645883000000	–4.450027000000	1.282950000000
H	1.660432000000	–2.924036000000	1.888381000000
H	0.368848000000	–3.936194000000	2.565984000000
H	2.620523000000	–3.522980000000	–1.667044000000
H	3.016137000000	–3.652866000000	0.064233000000
C	4.127354000000	–2.169303000000	–1.009462000000
O	4.086129000000	–0.941845000000	–0.992991000000
C	5.385692000000	–2.926839000000	–1.247797000000
H	6.112562000000	–2.312881000000	–1.795031000000
H	5.212203000000	–3.886174000000	–1.756003000000
H	5.802532000000	–3.146932000000	–0.248674000000
C	–3.549324000000	–1.007370000000	4.015541000000
C	–3.570980000000	–1.995391000000	5.200690000000
C	–4.395300000000	–1.570183000000	2.851794000000
C	–4.170794000000	0.321913000000	4.470003000000
C	–2.105228000000	–0.830683000000	3.529774000000
C	–1.388363000000	–1.937415000000	3.052578000000
C	–0.087838000000	–1.836538000000	2.554032000000
C	0.514412000000	–0.569933000000	2.546539000000
C	–0.144264000000	0.560656000000	3.039768000000
C	–1.453097000000	0.405785000000	3.516996000000
C	0.465591000000	1.949743000000	3.007304000000
O	1.850444000000	–0.455253000000	2.083693000000
C	2.814614000000	–0.576019000000	3.131009000000
H	–4.602290000000	–2.135476000000	5.558825000000
H	–2.967361000000	–1.618414000000	6.040573000000
H	–3.180612000000	–2.985089000000	4.919372000000
H	–5.440467000000	–1.700162000000	3.171361000000
H	–4.027964000000	–2.550194000000	2.511276000000
H	–4.383655000000	–0.885992000000	1.988767000000
H	–3.623934000000	0.762595000000	5.317735000000
H	–5.205765000000	0.152930000000	4.800747000000
H	–4.202839000000	1.059667000000	3.652931000000
H	–1.861267000000	–2.919685000000	3.054400000000
H	–1.966695000000	1.290740000000	3.888004000000
H	1.559226000000	1.887851000000	2.966881000000
H	0.204542000000	2.476389000000	3.935573000000
H	2.350136000000	–1.084111000000	3.993185000000
H	3.140530000000	0.417998000000	3.481069000000
C	4.021148000000	–1.389887000000	2.707159000000
O	4.023875000000	–1.978799000000	1.645352000000
C	5.176125000000	–1.406431000000	3.664478000000
H	5.799117000000	–2.293501000000	3.491985000000
H	4.851039000000	–1.362254000000	4.714779000000
H	5.789517000000	–0.509305000000	3.469666000000

C	–3.206778000000	4.806204000000	1.003708000000
C	–3.035632000000	5.930909000000	2.046354000000
C	–4.266654000000	3.795175000000	1.494406000000
C	–3.700475000000	5.426868000000	–0.311872000000
C	–1.872191000000	4.070061000000	0.828562000000
C	–1.276138000000	3.432737000000	1.929187000000
C	–0.076685000000	2.726045000000	1.825050000000
C	0.538889000000	2.667239000000	0.568472000000
C	–0.011961000000	3.272676000000	–0.560199000000
C	–1.216726000000	3.973121000000	–0.403375000000
C	0.573913000000	3.056778000000	–1.940500000000
O	1.803301000000	2.032760000000	0.466672000000
C	2.863104000000	2.939108000000	0.760536000000
H	–3.986935000000	6.466749000000	2.185124000000
H	–2.278767000000	6.660578000000	1.719765000000
H	–2.729618000000	5.540268000000	3.028532000000
H	–5.239274000000	4.295512000000	1.617119000000
H	–3.996876000000	3.354111000000	2.465976000000
H	–4.393088000000	2.974141000000	0.771168000000
H	–2.991733000000	6.170176000000	–0.707941000000
H	–4.656253000000	5.943126000000	–0.141642000000
H	–3.874724000000	4.662895000000	–1.085760000000
H	–1.761305000000	3.482705000000	2.904886000000
H	–1.646274000000	4.449781000000	–1.282593000000
H	1.660004000000	2.923980000000	–1.889062000000
H	0.368203000000	3.936127000000	–2.566281000000
H	2.621202000000	3.522899000000	1.666543000000
H	3.016700000000	3.653050000000	–0.064716000000
C	4.127784000000	2.169095000000	1.008856000000
O	4.086301000000	0.941656000000	0.992333000000
C	5.386287000000	2.926381000000	1.247127000000
H	6.113056000000	2.312283000000	1.794337000000
H	5.213008000000	3.885751000000	1.755347000000
H	5.803156000000	3.146423000000	0.248009000000

Ca$\mathbf{L}_c$*$^{2+}$, zero imaginary frequencies
137
–3454.76739893

Ca	0.000000000000	0.000000000000	2.748511000000
C	–1.701300000000	4.045377000000	–3.491861000000
C	–2.976450000000	4.912979000000	–3.520060000000
C	–1.882632000000	2.819138000000	–4.414287000000
C	–0.528343000000	4.884652000000	–4.020854000000
C	–1.451361000000	3.539308000000	–2.065079000000
C	–2.449326000000	2.814365000000	–1.395809000000
C	–2.268927000000	2.293008000000	–0.112661000000
C	–1.040257000000	2.535998000000	0.522293000000
C	0.000000000000	3.222828000000	–0.110917000000
C	–0.235436000000	3.722540000000	–1.399711000000
C	1.389502000000	3.331367000000	0.496198000000
O	–0.873188000000	2.103517000000	1.853988000000
C	–1.353743000000	3.034490000000	2.814011000000
H	–3.160809000000	5.281016000000	–4.540717000000
H	–2.875481000000	5.785936000000	–2.856989000000
H	–3.868824000000	4.350178000000	–3.207782000000
H	–2.060005000000	3.144407000000	–5.450866000000
H	–2.738959000000	2.199825000000	–4.107059000000
H	–0.984174000000	2.182075000000	–4.403292000000
H	–0.342603000000	5.770481000000	–3.394196000000
H	–0.755396000000	5.239993000000	–5.036346000000
H	0.401814000000	4.298904000000	–4.081951000000
H	–3.402794000000	2.638883000000	–1.893924000000
H	0.567405000000	4.269062000000	–1.889889000000
H	1.337821000000	3.221882000000	1.585507000000
H	1.802908000000	4.326605000000	0.279219000000
H	–2.209242000000	3.599766000000	2.404083000000
H	–0.575577000000	3.774596000000	3.077218000000
C	–1.803260000000	2.318512000000	4.066650000000
O	–1.713760000000	1.100395000000	4.134978000000
C	–2.314273000000	3.155433000000	5.195073000000
H	–2.966956000000	2.560196000000	5.846834000000
H	–2.828114000000	4.063936000000	4.849272000000
H	–1.439565000000	3.473719000000	5.790211000000
C	4.045271000000	1.701285000000	–3.491905000000
C	4.912955000000	2.976380000000	–3.520107000000
C	2.819012000000	1.882715000000	–4.414283000000
C	4.884451000000	0.528285000000	–4.020951000000
C	3.539239000000	1.451347000000	–2.065109000000
C	2.814314000000	2.449313000000	–1.395820000000
C	2.292983000000	2.268913000000	–0.112662000000
C	2.535988000000	1.040243000000	0.522286000000
C	3.222809000000	–0.000012000000	–0.110939000000
C	3.722486000000	0.235422000000	–1.399746000000
C	3.331376000000	–1.389511000000	0.496179000000
O	2.103529000000	0.873164000000	1.853987000000
C	3.034504000000	1.353726000000	2.814004000000
H	5.280978000000	3.160730000000	–4.540772000000
H	5.785923000000	2.875345000000	–2.857060000000
H	4.350220000000	3.868786000000	–3.207801000000
H	3.144256000000	2.060094000000	–5.450869000000
H	2.199762000000	2.739073000000	–4.107013000000
H	2.181894000000	0.984296000000	–4.403288000000
H	5.770282000000	0.342464000000	–3.394319000000
H	5.239785000000	0.755344000000	–5.036445000000
H	4.298636000000	–0.401829000000	–4.082062000000
H	2.638821000000	3.402780000000	–1.893931000000
H	4.268994000000	–0.567420000000	–1.889938000000
H	3.221909000000	–1.337828000000	1.585489000000
H	4.326617000000	–1.802902000000	0.279185000000
H	3.599796000000	2.209208000000	2.404060000000
H	3.774596000000	0.575555000000	3.077232000000
C	2.318523000000	1.803279000000	4.066629000000
O	1.100406000000	1.713786000000	4.134953000000
C	3.155442000000	2.314314000000	5.195044000000
H	2.560196000000	2.966984000000	5.846809000000
H	4.063928000000	2.828177000000	4.849232000000
H	3.473759000000	1.439615000000	5.790181000000
C	1.701300000000	–4.045377000000	–3.491861000000
C	2.976450000000	–4.912979000000	–3.520060000000
C	1.882632000000	–2.819138000000	–4.414287000000
C	0.528343000000	–4.884652000000	–4.020854000000
C	1.451361000000	–3.539308000000	–2.065079000000
C	2.449326000000	–2.814365000000	–1.395809000000
C	2.268927000000	–2.293008000000	–0.112661000000
C	1.040257000000	–2.535998000000	0.522293000000
C	0.000000000000	–3.222828000000	–0.110917000000
C	0.235436000000	–3.722540000000	–1.399711000000
C	–1.389502000000	–3.331367000000	0.496198000000
O	0.873188000000	–2.103517000000	1.853988000000
C	1.353743000000	–3.034490000000	2.814011000000
H	3.160809000000	–5.281016000000	–4.540717000000
H	2.875481000000	–5.785936000000	–2.856989000000
H	3.868824000000	–4.350178000000	–3.207782000000
H	2.060005000000	–3.144407000000	–5.450866000000
H	2.738959000000	–2.199825000000	–4.107059000000
H	0.984174000000	–2.182075000000	–4.403292000000
H	0.342603000000	–5.770481000000	–3.394196000000
H	0.755396000000	–5.239993000000	–5.036346000000
H	–0.401814000000	–4.298904000000	–4.081951000000
H	3.402794000000	–2.638883000000	–1.893924000000
H	–0.567405000000	–4.269062000000	–1.889889000000
H	–1.337821000000	–3.221882000000	1.585507000000
H	–1.802908000000	–4.326605000000	0.279219000000
H	2.209242000000	–3.599766000000	2.404083000000
H	0.575577000000	–3.774596000000	3.077218000000
C	1.803260000000	–2.318512000000	4.066650000000
O	1.713760000000	–1.100395000000	4.134978000000
C	2.314273000000	–3.155433000000	5.195073000000
H	2.966956000000	–2.560196000000	5.846834000000
H	2.828114000000	–4.063936000000	4.849272000000
H	1.439565000000	–3.473719000000	5.790211000000

C	–4.045271000000	–1.701285000000	–3.491905000000
C	–4.912955000000	–2.976380000000	–3.520107000000
C	–2.819012000000	–1.882715000000	–4.414283000000
C	–4.884451000000	–0.528285000000	–4.020951000000
C	–3.539239000000	–1.451347000000	–2.065109000000
C	–2.814314000000	–2.449313000000	–1.395820000000
C	–2.292983000000	–2.268913000000	–0.112662000000
C	–2.535988000000	–1.040243000000	0.522286000000
C	–3.222809000000	0.000012000000	–0.110939000000
C	–3.722486000000	–0.235422000000	–1.399746000000
C	–3.331376000000	1.389511000000	0.496179000000
O	–2.103529000000	–0.873164000000	1.853987000000
C	–3.034504000000	–1.353726000000	2.814004000000
H	–5.280978000000	–3.160730000000	–4.540772000000
H	–5.785923000000	–2.875345000000	–2.857060000000
H	–4.350220000000	–3.868786000000	–3.207801000000
H	–3.144256000000	–2.060094000000	–5.450869000000
H	–2.199762000000	–2.739073000000	–4.107013000000
H	–2.181894000000	–0.984296000000	–4.403288000000
H	–5.770282000000	–0.342464000000	–3.394319000000
H	–5.239785000000	–0.755344000000	–5.036445000000
H	–4.298636000000	0.401829000000	–4.082062000000
H	–2.638821000000	–3.402780000000	–1.893931000000
H	–4.268994000000	0.567420000000	–1.889938000000
H	–3.221909000000	1.337828000000	1.585489000000
H	–4.326617000000	1.802902000000	0.279185000000
H	–3.599796000000	–2.209208000000	2.404060000000
H	–3.774596000000	–0.575555000000	3.077232000000
C	–2.318523000000	–1.803279000000	4.066629000000
O	–1.100406000000	–1.713786000000	4.134953000000
C	–3.155442000000	–2.314314000000	5.195044000000
H	–2.560196000000	–2.966984000000	5.846809000000
H	–4.063928000000	–2.828177000000	4.849232000000
H	–3.473759000000	–1.439615000000	5.790181000000

Sr$\mathbf{L_c}^{*2+}$, zero imaginary frequencies
137
–2807.95790666

Sr	0.000000000000	0.000000000000	2.740285000000
C	3.023514000000	3.069572000000	–3.624901000000
C	3.290327000000	4.586927000000	–3.698932000000
C	1.821408000000	2.705693000000	–4.525006000000
C	4.265976000000	2.331373000000	–4.146185000000
C	2.678108000000	2.671846000000	–2.183621000000
C	1.632521000000	3.321811000000	–1.510314000000
C	1.246451000000	2.982977000000	–0.211079000000
C	1.972045000000	1.968623000000	0.437508000000
C	2.981432000000	1.245052000000	–0.208340000000
C	3.325226000000	1.630155000000	–1.512261000000
C	3.613088000000	0.000001000000	0.401396000000
O	1.683089000000	1.694644000000	1.788625000000
C	2.385301000000	2.520803000000	2.705519000000
H	3.543139000000	4.876135000000	–4.730208000000
H	4.132301000000	4.872673000000	–3.049823000000
H	2.413059000000	5.179113000000	–3.398597000000
H	2.032496000000	2.973970000000	–5.571593000000
H	0.908304000000	3.238902000000	–4.220027000000
H	1.610835000000	1.625356000000	–4.483138000000
H	5.153276000000	2.535415000000	–3.527343000000
H	4.492249000000	2.663739000000	–5.169600000000
H	4.111459000000	1.242262000000	–4.186329000000
H	1.090175000000	4.118628000000	–2.018205000000
H	4.125834000000	1.088608000000	–2.010745000000
H	3.485133000000	0.004067000000	1.490053000000
H	4.692436000000	0.001428000000	0.191334000000
H	2.546944000000	3.524471000000	2.272955000000
H	3.385760000000	2.106413000000	2.932295000000
C	1.608169000000	2.678356000000	3.994972000000
O	0.517758000000	2.138757000000	4.126718000000
C	2.247201000000	3.484574000000	5.081555000000
H	1.479384000000	3.932178000000	5.726475000000
H	2.928536000000	4.255138000000	4.693356000000
H	2.843654000000	2.791722000000	5.701896000000
C	3.069620000000	–3.023481000000	–3.624896000000
C	4.586996000000	–3.290181000000	–3.698896000000
C	2.705669000000	–1.821415000000	–4.525028000000
C	2.331520000000	–4.266006000000	–4.146171000000
C	2.671859000000	–2.678079000000	–2.183625000000
C	3.321820000000	–1.632500000000	–1.510304000000
C	2.982980000000	–1.246445000000	–0.211067000000
C	1.968620000000	–1.972043000000	0.437506000000
C	1.245053000000	–2.981424000000	–0.208356000000
C	1.630168000000	–3.325210000000	–1.512276000000
C	0.000000000000	–3.613091000000	0.401371000000
O	1.694636000000	–1.683094000000	1.788623000000
C	2.520794000000	–2.385308000000	2.705517000000
H	4.876250000000	–3.542947000000	–4.730170000000
H	4.872788000000	–4.132148000000	–3.049799000000
H	5.179106000000	–2.412875000000	–3.398521000000
H	2.973956000000	–2.032514000000	–5.571610000000
H	3.238826000000	–0.908273000000	–4.220075000000
H	1.625320000000	–1.610905000000	–4.483163000000
H	2.535670000000	–5.153296000000	–3.527351000000
H	2.663871000000	–4.492233000000	–5.169601000000
H	1.242393000000	–4.111591000000	–4.186271000000
H	4.118645000000	–1.090153000000	–2.018182000000
H	1.088631000000	–4.125818000000	–2.010768000000
H	0.004068000000	–3.485153000000	1.490029000000
H	0.001430000000	–4.692437000000	0.191293000000
H	3.524462000000	–2.546951000000	2.272952000000
H	2.106403000000	–3.385767000000	2.932293000000
C	2.678351000000	–1.608174000000	3.994969000000
O	2.138759000000	–0.517759000000	4.126712000000
C	3.484568000000	–2.247207000000	5.081552000000
H	3.932164000000	–1.479392000000	5.726478000000
H	4.255139000000	–2.928535000000	4.693350000000
H	2.791718000000	–2.843671000000	5.701884000000
C	–3.023514000000	–3.069572000000	–3.624901000000
C	–3.290327000000	–4.586927000000	–3.698932000000
C	–1.821408000000	–2.705693000000	–4.525006000000
C	–4.265976000000	–2.331373000000	–4.146185000000
C	–2.678108000000	–2.671846000000	–2.183621000000
C	–1.632521000000	–3.321811000000	–1.510314000000
C	–1.246451000000	–2.982977000000	–0.211079000000
C	–1.972045000000	–1.968623000000	0.437508000000
C	–2.981432000000	–1.245052000000	–0.208340000000
C	–3.325226000000	–1.630155000000	–1.512261000000
C	–3.613088000000	–0.000001000000	0.401396000000
O	–1.683089000000	–1.694644000000	1.788625000000
C	–2.385301000000	–2.520803000000	2.705519000000
H	–3.543139000000	–4.876135000000	–4.730208000000
H	–4.132301000000	–4.872673000000	–3.049823000000
H	–2.413059000000	–5.179113000000	–3.398597000000
H	–2.032496000000	–2.973970000000	–5.571593000000
H	–0.908304000000	–3.238902000000	–4.220027000000
H	–1.610835000000	–1.625356000000	–4.483138000000
H	–5.153276000000	–2.535415000000	–3.527343000000
H	–4.492249000000	–2.663739000000	–5.169600000000
H	–4.111459000000	–1.242262000000	–4.186329000000
H	–1.090175000000	–4.118628000000	–2.018205000000
H	–4.125834000000	–1.088608000000	–2.010745000000
H	–3.485133000000	–0.004067000000	1.490053000000
H	–4.692436000000	–0.001428000000	0.191334000000
H	–2.546944000000	–3.524471000000	2.272955000000
H	–3.385760000000	–2.106413000000	2.932295000000
C	–1.608169000000	–2.678356000000	3.994972000000
O	–0.517758000000	–2.138757000000	4.126718000000
C	–2.247201000000	–3.484574000000	5.081555000000
H	–1.479384000000	–3.932178000000	5.726475000000
H	–2.928536000000	–4.255138000000	4.693356000000
H	–2.843654000000	–2.791722000000	5.701896000000

C	–3.069620000000	3.023481000000	–3.624896000000
C	–4.586996000000	3.290181000000	–3.698896000000
C	–2.705669000000	1.821415000000	–4.525028000000
C	–2.331520000000	4.266006000000	–4.146171000000
C	–2.671859000000	2.678079000000	–2.183625000000
C	–3.321820000000	1.632500000000	–1.510304000000
C	–2.982980000000	1.246445000000	–0.211067000000
C	–1.968620000000	1.972043000000	0.437506000000
C	–1.245053000000	2.981424000000	–0.208356000000
C	–1.630168000000	3.325210000000	–1.512276000000
C	0.000000000000	3.613091000000	0.401371000000
O	–1.694636000000	1.683094000000	1.788623000000
C	–2.520794000000	2.385308000000	2.705517000000
H	–4.876250000000	3.542947000000	–4.730170000000
H	–4.872788000000	4.132148000000	–3.049799000000
H	–5.179106000000	2.412875000000	–3.398521000000
H	–2.973956000000	2.032514000000	–5.571610000000
H	–3.238826000000	0.908273000000	–4.220075000000
H	–1.625320000000	1.610905000000	–4.483163000000
H	–2.535670000000	5.153296000000	–3.527351000000
H	–2.663871000000	4.492233000000	–5.169601000000
H	–1.242393000000	4.111591000000	–4.186271000000
H	–4.118645000000	1.090153000000	–2.018182000000
H	–1.088631000000	4.125818000000	–2.010768000000
H	–0.004068000000	3.485153000000	1.490029000000
H	–0.001430000000	4.692437000000	0.191293000000
H	–3.524462000000	2.546951000000	2.272952000000
H	–2.106403000000	3.385767000000	2.932293000000
C	–2.678351000000	1.608174000000	3.994969000000
O	–2.138759000000	0.517759000000	4.126712000000
C	–3.484568000000	2.247207000000	5.081552000000
H	–3.932164000000	1.479392000000	5.726478000000
H	–4.255139000000	2.928535000000	4.693350000000
H	–2.791718000000	2.843671000000	5.701884000000

$\mathrm{Ba}\mathbf{L}_c{}^{*2+}$, zero imaginary frequencies
137
–2802.7011506

Ba	0.000000000000	0.000000000000	2.820161000000
C	3.019555000000	–2.978012000000	–3.757081000000
C	4.540183000000	–3.213366000000	–3.862686000000
C	2.614411000000	–1.777991000000	–4.642017000000
C	2.297860000000	–4.232946000000	–4.271934000000
C	2.642085000000	–2.649546000000	–2.306346000000
C	3.297226000000	–1.609334000000	–1.630511000000
C	2.984818000000	–1.246977000000	–0.317654000000
C	1.995037000000	–1.997593000000	0.343367000000
C	1.247254000000	–2.984752000000	–0.312583000000
C	1.609382000000	–3.304337000000	–1.629297000000
C	0.000000000000	–3.615698000000	0.298895000000
O	1.787907000000	–1.772479000000	1.717202000000
C	2.660575000000	–2.527960000000	2.544088000000
H	4.813651000000	–3.455100000000	–4.900874000000
H	4.856065000000	–4.052542000000	–3.223993000000
H	5.120711000000	–2.325783000000	–3.570050000000
H	2.864607000000	–1.977283000000	–5.695414000000
H	3.137301000000	–0.857671000000	–4.341501000000
H	1.531735000000	–1.587004000000	–4.575969000000
H	2.526963000000	–5.117555000000	–3.658028000000
H	2.620238000000	–4.450137000000	–5.300504000000
H	1.205667000000	–4.099570000000	–4.297285000000
H	4.080438000000	–1.056012000000	–2.146626000000
H	1.059386000000	–4.094971000000	–2.133535000000
H	0.000904000000	–3.485378000000	1.387608000000
H	0.001002000000	–4.696038000000	0.092179000000
H	3.661258000000	–2.605332000000	2.080771000000
H	2.292253000000	–3.563661000000	2.672384000000
C	2.822032000000	–1.881831000000	3.904044000000
O	2.274219000000	–0.817977000000	4.155031000000
C	3.652298000000	–2.620500000000	4.908504000000
H	4.109872000000	–1.914675000000	5.614473000000
H	4.418099000000	–3.255673000000	4.440563000000
H	2.977398000000	–3.279273000000	5.483799000000
C	–2.978075000000	–3.019332000000	–3.757129000000
C	–3.213767000000	–4.539903000000	–3.862824000000
C	–1.777926000000	–2.614404000000	–4.641988000000
C	–4.232822000000	–2.297331000000	–4.272007000000
C	–2.649605000000	–2.641978000000	–2.306364000000
C	–1.609383000000	–3.297150000000	–1.630568000000
C	–1.246989000000	–2.984782000000	–0.317709000000
C	–1.997602000000	–1.995028000000	0.343362000000
C	–2.984784000000	–1.247236000000	–0.312539000000
C	–3.304377000000	–1.609297000000	–1.629267000000
C	–3.615707000000	–0.000001000000	0.298986000000
O	–1.772467000000	–1.787915000000	1.717197000000
C	–2.527952000000	–2.660576000000	2.544088000000
H	–3.455604000000	–4.813247000000	–4.901021000000
H	–4.052988000000	–4.855641000000	–3.224119000000
H	–2.326303000000	–5.120650000000	–3.570268000000
H	–1.977206000000	–2.864538000000	–5.695402000000
H	–0.857729000000	–3.137482000000	–4.341428000000
H	–1.586728000000	–1.531768000000	–4.575903000000
H	–5.117488000000	–2.526180000000	–3.658089000000
H	–4.450096000000	–2.619690000000	–5.300565000000
H	–4.099162000000	–1.205172000000	–4.297401000000
H	–1.056068000000	–4.080339000000	–2.146723000000
H	–4.095006000000	–1.059265000000	–2.133478000000
H	–3.485332000000	–0.000909000000	1.387694000000
H	–4.696056000000	–0.001003000000	0.092319000000
H	–2.605344000000	–3.661256000000	2.080767000000
H	–3.563646000000	–2.292238000000	2.672395000000
C	–1.881817000000	–2.822044000000	3.904039000000
O	–0.817961000000	–2.274235000000	4.155025000000
C	–2.620491000000	–3.652302000000	4.908502000000
H	–1.914668000000	–4.109882000000	5.614469000000
H	–3.255673000000	–4.418097000000	4.440564000000
H	–3.279256000000	–2.977396000000	5.483798000000
C	–3.019555000000	2.978012000000	–3.757081000000
C	–4.540183000000	3.213366000000	–3.862686000000
C	–2.614411000000	1.777991000000	–4.642017000000
C	–2.297860000000	4.232946000000	–4.271934000000
C	–2.642085000000	2.649546000000	–2.306346000000
C	–3.297226000000	1.609334000000	–1.630511000000
C	–2.984818000000	1.246977000000	–0.317654000000
C	–1.995037000000	1.997593000000	0.343367000000
C	–1.247254000000	2.984752000000	–0.312583000000
C	–1.609382000000	3.304337000000	–1.629297000000
C	0.000000000000	3.615698000000	0.298895000000
O	–1.787907000000	1.772479000000	1.717202000000
C	–2.660575000000	2.527960000000	2.544088000000
H	–4.813651000000	3.455100000000	–4.900874000000
H	–4.856065000000	4.052542000000	–3.223993000000
H	–5.120711000000	2.325783000000	–3.570050000000
H	–2.864607000000	1.977283000000	–5.695414000000
H	–3.137301000000	0.857671000000	–4.341501000000
H	–1.531735000000	1.587004000000	–4.575969000000
H	–2.526963000000	5.117555000000	–3.658028000000
H	–2.620238000000	4.450137000000	–5.300504000000
H	–1.205667000000	4.099570000000	–4.297285000000
H	–4.080438000000	1.056012000000	–2.146626000000
H	–1.059386000000	4.094971000000	–2.133535000000
H	–0.000904000000	3.485378000000	1.387608000000
H	–0.001002000000	4.696038000000	0.092179000000
H	–3.661258000000	2.605332000000	2.080771000000
H	–2.292253000000	3.563661000000	2.672384000000
C	–2.822032000000	1.881831000000	3.904044000000
O	–2.274219000000	0.817977000000	4.155031000000
C	–3.652298000000	2.620500000000	4.908504000000
H	–4.109872000000	1.914675000000	5.614473000000
H	–4.418099000000	3.255673000000	4.440563000000
H	–2.977398000000	3.279273000000	5.483799000000

C	2.978075000000	3.019332000000	–3.757129000000
C	3.213767000000	4.539903000000	–3.862824000000
C	1.777926000000	2.614404000000	–4.641988000000
C	4.232822000000	2.297331000000	–4.272007000000
C	2.649605000000	2.641978000000	–2.306364000000
C	1.609383000000	3.297150000000	–1.630568000000
C	1.246989000000	2.984782000000	–0.317709000000
C	1.997602000000	1.995028000000	0.343362000000
C	2.984784000000	1.247236000000	–0.312539000000
C	3.304377000000	1.609297000000	–1.629267000000
C	3.615707000000	0.000001000000	0.298986000000
O	1.772467000000	1.787915000000	1.717197000000
C	2.527952000000	2.660576000000	2.544088000000
H	3.455604000000	4.813247000000	–4.901021000000
H	4.052988000000	4.855641000000	–3.224119000000
H	2.326303000000	5.120650000000	–3.570268000000
H	1.977206000000	2.864538000000	–5.695402000000
H	0.857729000000	3.137482000000	–4.341428000000
H	1.586728000000	1.531768000000	–4.575903000000
H	5.117488000000	2.526180000000	–3.658089000000
H	4.450096000000	2.619690000000	–5.300565000000
H	4.099162000000	1.205172000000	–4.297401000000
H	1.056068000000	4.080339000000	–2.146723000000
H	4.095006000000	1.059265000000	–2.133478000000
H	3.485332000000	0.000909000000	1.387694000000
H	4.696056000000	0.001003000000	0.092319000000
H	2.605344000000	3.661256000000	2.080767000000
H	3.563646000000	2.292238000000	2.672395000000
C	1.881817000000	2.822044000000	3.904039000000
O	0.817961000000	2.274235000000	4.155025000000
C	2.620491000000	3.652302000000	4.908502000000
H	1.914668000000	4.109882000000	5.614469000000
H	3.255673000000	4.418097000000	4.440564000000
H	3.279256000000	2.977396000000	5.483798000000

L_cMeCN, zero imaginary frequencies
142
–2910.13790218

C	–3.580969000000	–0.448935000000	–3.634168000000
C	–3.773118000000	–1.192932000000	–4.971893000000
C	–4.213636000000	0.947011000000	–3.746464000000
C	–4.311981000000	–1.214949000000	–2.510038000000
C	–2.079182000000	–0.373858000000	–3.320162000000
C	–1.391657000000	0.835832000000	–3.189101000000
C	–0.017020000000	0.887939000000	–2.919691000000
C	0.688421000000	–0.314423000000	–2.761596000000
C	0.013292000000	–1.550109000000	–2.778891000000
C	–1.347919000000	–1.551465000000	–3.099359000000
C	0.670676000000	–2.841718000000	–2.318779000000
O	2.054473000000	–0.258422000000	–2.567468000000
C	2.820386000000	–0.665291000000	–3.667656000000
H	–4.843648000000	–1.261660000000	–5.225188000000
H	–3.370935000000	–2.216754000000	–4.927879000000
H	–3.259560000000	–0.665261000000	–5.791021000000
H	–5.288662000000	0.850436000000	–3.963994000000
H	–3.762475000000	1.534486000000	–4.561237000000
H	–4.110242000000	1.512773000000	–2.808834000000
H	–3.911160000000	–2.230064000000	–2.371963000000
H	–5.383317000000	–1.309668000000	–2.750000000000
H	–4.227139000000	–0.678813000000	–1.553806000000
H	–1.925695000000	1.779995000000	–3.280435000000
H	–1.862258000000	–2.514370000000	–3.123340000000
H	1.760268000000	–2.747275000000	–2.300846000000
H	0.400364000000	–3.652059000000	–3.013151000000
H	2.563679000000	–0.081464000000	–4.577965000000
H	2.656306000000	–1.728363000000	–3.916924000000
C	4.300429000000	–0.451275000000	–3.409439000000
O	4.708581000000	0.284753000000	–2.536047000000
C	5.225644000000	–1.232440000000	–4.307546000000
H	6.221984000000	–0.771864000000	–4.319108000000
H	4.831101000000	–1.333138000000	–5.330452000000
H	5.296864000000	–2.243343000000	–3.871691000000
C	–2.611856000000	–5.331078000000	0.643017000000
C	–2.330109000000	–6.695518000000	–0.018410000000
C	–3.798024000000	–4.644562000000	–0.068939000000
C	–3.008058000000	–5.573831000000	2.107375000000
C	–1.374985000000	–4.429531000000	0.519165000000
C	–0.881010000000	–4.089752000000	–0.749869000000
C	0.202868000000	–3.229113000000	–0.925365000000
C	0.815574000000	–2.669924000000	0.216216000000
C	0.355612000000	–2.986536000000	1.508291000000
C	–0.721138000000	–3.880940000000	1.625269000000
C	0.850584000000	–2.289538000000	2.771900000000
O	1.784304000000	–1.729110000000	–0.000304000000
C	3.087465000000	–1.899532000000	0.493129000000
H	–3.214818000000	–7.349189000000	0.051227000000
H	–1.487649000000	–7.203509000000	0.476541000000
H	–2.077066000000	–6.587304000000	–1.083688000000
H	–4.709081000000	–5.259056000000	0.015703000000
H	–3.596260000000	–4.490846000000	–1.139456000000
H	–4.004472000000	–3.658639000000	0.376144000000
H	–2.204514000000	–6.071956000000	2.671392000000
H	–3.897044000000	–6.221736000000	2.150797000000
H	–3.258436000000	–4.633519000000	2.622407000000
H	–1.353264000000	–4.505953000000	–1.642404000000
H	–1.065990000000	–4.119751000000	2.630780000000
H	1.918815000000	–2.060891000000	2.727927000000
H	0.693602000000	–2.971051000000	3.621951000000
H	3.154485000000	–2.734431000000	1.217036000000
H	3.384516000000	–0.997166000000	1.045309000000
C	4.121245000000	–2.218528000000	–0.573421000000
O	3.840422000000	–2.676347000000	–1.663488000000
C	5.547973000000	–1.915941000000	–0.182967000000
H	6.226264000000	–2.656330000000	–0.628968000000
H	5.688614000000	–1.842223000000	0.903844000000
H	5.785395000000	–0.930768000000	–0.620576000000
C	–3.584034000000	–0.030301000000	3.680373000000
C	–3.742942000000	–0.558517000000	5.121013000000
C	–4.242622000000	–1.021150000000	2.694845000000
C	–4.315041000000	1.315967000000	3.560071000000
C	–2.087122000000	0.098099000000	3.359808000000
C	–1.280828000000	–1.048354000000	3.325890000000
C	0.078542000000	–1.000458000000	3.004629000000
C	0.670821000000	0.253760000000	2.773410000000
C	–0.111299000000	1.420912000000	2.741037000000
C	–1.478013000000	1.315007000000	3.037308000000
C	0.449441000000	2.771279000000	2.325632000000
O	2.036956000000	0.303723000000	2.568311000000
C	2.768462000000	0.754408000000	3.675264000000
H	–4.809446000000	–0.664225000000	5.378634000000
H	–3.281797000000	0.132332000000	5.844286000000
H	–3.266717000000	–1.543132000000	5.246718000000
H	–5.311532000000	–1.141156000000	2.934058000000
H	–3.777965000000	–2.017859000000	2.738457000000
H	–4.167564000000	–0.652533000000	1.661375000000
H	–3.915739000000	2.059941000000	4.266807000000
H	–5.382940000000	1.179699000000	3.791482000000
H	–4.240027000000	1.723922000000	2.541171000000
H	–1.728913000000	–2.025432000000	3.511849000000
H	–2.074249000000	2.224633000000	2.982987000000
H	1.543132000000	2.756829000000	2.335161000000
H	0.105090000000	3.531880000000	3.043058000000
H	2.554869000000	0.143080000000	4.578940000000
H	2.522382000000	1.798941000000	3.934277000000
C	4.260619000000	0.652292000000	3.420392000000
O	4.725068000000	–0.071995000000	2.564415000000
C	5.122347000000	1.524929000000	4.295693000000
H	6.154624000000	1.151968000000	4.307554000000
H	4.726561000000	1.610626000000	5.319540000000
H	5.101671000000	2.530263000000	3.841600000000
C	–2.673725000000	5.436783000000	–0.731466000000

C	–2.170780000000	6.687080000000	–1.481668000000
C	–3.304612000000	5.883396000000	0.596140000000
C	–3.764758000000	4.729227000000	–1.564463000000
C	–1.501486000000	4.469413000000	–0.511889000000
C	–1.048241000000	4.097118000000	0.757840000000
C	–0.000248000000	3.187216000000	0.937414000000
C	0.612398000000	2.627223000000	–0.195001000000
C	0.170395000000	2.953482000000	–1.493183000000
C	–0.862559000000	3.888898000000	–1.615953000000
C	0.664725000000	2.234540000000	–2.740298000000
O	1.558823000000	1.656125000000	0.020912000000
C	2.877141000000	1.826029000000	–0.427988000000
H	–3.001202000000	7.388971000000	–1.664081000000
H	–1.732517000000	6.424645000000	–2.456638000000
H	–1.398658000000	7.212386000000	–0.897364000000
H	–4.145764000000	6.565642000000	0.397529000000
H	–2.584644000000	6.421133000000	1.232510000000
H	–3.695927000000	5.025174000000	1.163153000000
H	–3.399846000000	4.458566000000	–2.567040000000
H	–4.634979000000	5.392914000000	–1.697057000000
H	–4.088877000000	3.805225000000	–1.064751000000
H	–1.517399000000	4.510446000000	1.650118000000
H	–1.200304000000	4.140859000000	–2.623665000000
H	1.747692000000	2.079378000000	–2.728414000000
H	0.437082000000	2.869778000000	–3.609951000000
H	2.945953000000	2.548953000000	–1.261764000000
H	3.224692000000	0.874295000000	–0.849021000000
C	3.875828000000	2.321680000000	0.600226000000
O	3.571488000000	2.824116000000	1.664259000000
C	5.322103000000	2.138391000000	0.199763000000
H	5.922466000000	2.977179000000	0.578499000000
H	5.453790000000	2.005239000000	–0.882092000000
H	5.670229000000	1.218112000000	0.699332000000
C	–2.479019000000	0.810786000000	–0.022712000000
N	–3.338630000000	1.588300000000	–0.056977000000
C	–1.454208000000	–0.214763000000	0.027835000000
H	–0.446682000000	0.216281000000	–0.010842000000
H	–1.576320000000	–0.897359000000	–0.818888000000
H	–1.554054000000	–0.788328000000	0.955053000000

Li$\mathbf{L}_c$MeCN$^+$, zero imaginary frequencies
143
–2917.62521654

Li	2.626478000000	–0.062403000000	0.042502000000
C	–3.104933000000	4.468751000000	1.212610000000
C	–3.144663000000	5.390910000000	2.447768000000
C	–4.115714000000	3.314064000000	1.391366000000
C	–3.515694000000	5.290124000000	–0.019092000000
C	–1.705501000000	3.857154000000	1.058642000000
C	–1.122719000000	3.162083000000	2.129422000000
C	0.115670000000	2.525107000000	2.023378000000
C	0.787383000000	2.584028000000	0.791872000000
C	0.248176000000	3.264536000000	–0.304663000000
C	–0.989634000000	3.903544000000	–0.141951000000
C	0.893733000000	3.199002000000	–1.677029000000
O	2.024820000000	1.953416000000	0.669916000000
C	3.109890000000	2.801425000000	0.987928000000
H	–4.146240000000	5.834530000000	2.558143000000
H	–2.416285000000	6.211074000000	2.351615000000
H	–2.922232000000	4.847238000000	3.377862000000
H	–5.135828000000	3.711064000000	1.512909000000
H	–3.884971000000	2.702170000000	2.275237000000
H	–4.113984000000	2.646560000000	0.515338000000
H	–2.815596000000	6.117714000000	–0.211652000000
H	–4.512550000000	5.726531000000	0.141821000000
H	–3.575751000000	4.668576000000	–0.925559000000
H	–1.653904000000	3.100804000000	3.079799000000
H	–1.402684000000	4.437143000000	–0.995714000000
H	1.971488000000	3.024294000000	–1.596377000000
H	0.733096000000	4.154454000000	–2.197283000000
H	2.931210000000	3.322638000000	1.948548000000
H	3.262815000000	3.574821000000	0.217310000000
C	4.365341000000	1.978169000000	1.132547000000
O	4.303035000000	0.761455000000	1.111005000000
C	5.653411000000	2.727893000000	1.291385000000
H	6.419006000000	2.088422000000	1.749340000000
H	5.527439000000	3.658308000000	1.865025000000
H	5.979948000000	3.001787000000	0.274060000000
C	–3.188867000000	1.436066000000	–4.083193000000
C	–3.216947000000	2.588639000000	–5.107400000000
C	–4.056494000000	1.805003000000	–2.858982000000
C	–3.788614000000	0.181549000000	–4.736268000000
C	–1.747643000000	1.205475000000	–3.608762000000
C	–1.038496000000	2.246687000000	–2.994097000000
C	0.246110000000	2.078684000000	–2.471498000000
C	0.853685000000	0.817729000000	–2.590539000000
C	0.184369000000	–0.251520000000	–3.201626000000
C	–1.104890000000	–0.031320000000	–3.707569000000
C	0.739197000000	–1.665755000000	–3.202888000000
O	2.136941000000	0.643277000000	–2.074837000000
C	3.175289000000	0.677394000000	–3.032569000000
H	–4.246884000000	2.765637000000	–5.454428000000
H	–2.596529000000	2.350478000000	–5.985312000000
H	–2.845505000000	3.530803000000	–4.676909000000
H	–5.095560000000	1.994429000000	–3.170469000000
H	–3.688820000000	2.711795000000	–2.355310000000
H	–4.069531000000	0.987790000000	–2.121405000000
H	–3.216278000000	–0.131450000000	–5.623258000000
H	–4.818155000000	0.389845000000	–5.063177000000
H	–3.832217000000	–0.663658000000	–4.032801000000
H	–1.508874000000	3.225107000000	–2.894299000000
H	–1.620689000000	–0.871820000000	–4.167220000000
H	1.824417000000	–1.651325000000	–3.063851000000
H	0.531796000000	–2.135683000000	–4.175150000000
H	2.838903000000	1.199827000000	–3.947873000000
H	3.473900000000	–0.340269000000	–3.335559000000
C	4.397849000000	1.425416000000	–2.525738000000
O	4.326841000000	2.216306000000	–1.609770000000
C	5.684328000000	1.123346000000	–3.245952000000
H	6.401486000000	1.942148000000	–3.105930000000
H	5.525757000000	0.929605000000	–4.318065000000
H	6.099375000000	0.204394000000	–2.798397000000
C	–3.255031000000	–4.338834000000	–1.579549000000
C	–3.005178000000	–5.634542000000	–2.381095000000
C	–4.139376000000	–3.383459000000	–2.408076000000
C	–4.012154000000	–4.684079000000	–0.286856000000
C	–1.894661000000	–3.689285000000	–1.286546000000
C	–1.177275000000	–3.058551000000	–2.313680000000
C	0.073998000000	–2.473228000000	–2.103238000000
C	0.633785000000	–2.548117000000	–0.819328000000
C	–0.040773000000	–3.167036000000	0.237997000000
C	–1.302589000000	–3.721645000000	–0.018814000000
C	0.518549000000	–3.155049000000	1.649300000000
O	1.905262000000	–2.009958000000	–0.610286000000
C	2.937041000000	–2.941436000000	–0.867889000000
H	–3.960032000000	–6.132093000000	–2.613976000000
H	–2.382610000000	–6.339180000000	–1.807851000000
H	–2.490755000000	–5.426289000000	–3.332252000000
H	–5.117718000000	–3.851820000000	–2.596190000000
H	–3.699093000000	–3.153798000000	–3.389954000000
H	–4.310434000000	–2.440920000000	–1.868502000000
H	–3.482102000000	–5.438464000000	0.314543000000
H	–4.997055000000	–5.105464000000	–0.537714000000
H	–4.176185000000	–3.787305000000	0.328734000000
H	–1.605523000000	–3.011984000000	–3.315203000000
H	–1.829511000000	–4.193903000000	0.808068000000
H	1.610256000000	–3.070649000000	1.631804000000
H	0.251295000000	–4.098368000000	2.147065000000
H	2.769104000000	–3.453402000000	–1.835344000000
H	2.986149000000	–3.719978000000	–0.089055000000
C	4.260272000000	–2.223328000000	–0.948357000000
O	4.296447000000	–1.005124000000	–0.937999000000
C	5.489987000000	–3.076334000000	–1.032085000000
H	6.329334000000	–2.505214000000	–1.449350000000
H	5.320303000000	–3.998381000000	–1.607979000000
H	5.736454000000	–3.367513000000	0.002836000000

C	–3.413682000000	–1.135567000000	4.188330000000
C	–3.268387000000	–1.179049000000	5.724878000000
C	–4.137067000000	–2.406890000000	3.715241000000
C	–4.274921000000	0.079777000000	3.787401000000
C	–2.008152000000	–1.019435000000	3.581063000000
C	–1.377931000000	–2.085732000000	2.931288000000
C	–0.072554000000	–1.996292000000	2.429048000000
C	0.620142000000	–0.787404000000	2.579524000000
C	0.009988000000	0.324649000000	3.183560000000
C	–1.286078000000	0.177646000000	3.687242000000
C	0.653389000000	1.699769000000	3.179681000000
O	1.935145000000	–0.701454000000	2.120231000000
C	2.915776000000	–0.803355000000	3.133063000000
H	–4.257992000000	–1.262880000000	6.201408000000
H	–2.783037000000	–0.268496000000	6.109594000000
H	–2.662584000000	–2.043174000000	6.039413000000
H	–5.150651000000	–2.434420000000	4.142300000000
H	–3.622338000000	–3.322232000000	4.045242000000
H	–4.233171000000	–2.426539000000	2.619580000000
H	–3.856928000000	1.027392000000	4.158983000000
H	–5.282555000000	–0.019086000000	4.219525000000
H	–4.378575000000	0.145643000000	2.695113000000
H	–1.908948000000	–3.026670000000	2.803024000000
H	–1.747768000000	1.038844000000	4.170792000000
H	1.742016000000	1.614762000000	3.108232000000
H	0.419876000000	2.211203000000	4.124567000000
H	2.490846000000	–1.286081000000	4.033060000000
H	3.275621000000	0.191981000000	3.443947000000
C	4.104346000000	–1.647908000000	2.704562000000
O	4.032237000000	–2.431645000000	1.782844000000
C	5.360570000000	–1.448547000000	3.509802000000
H	6.013345000000	–2.326267000000	3.420811000000
H	5.147887000000	–1.232928000000	4.568214000000
H	5.881947000000	–0.572570000000	3.088133000000
C	–2.975706000000	–0.496331000000	0.134443000000
N	–3.990472000000	–1.031214000000	0.298857000000
C	–1.706285000000	0.176810000000	–0.078361000000
H	–1.048619000000	0.006917000000	0.782411000000
H	–1.860247000000	1.257204000000	–0.198627000000
H	–1.225475000000	–0.216548000000	–0.982657000000

Na$\mathbf{L}_e$MeCN$^+$, zero imaginary frequencies
143
–3072.35341114

Na	2.700104000000	–0.051323000000	0.052356000000
C	–3.219637000000	4.448812000000	0.664273000000
C	–3.314125000000	5.490359000000	1.797439000000
C	–4.197892000000	3.286200000000	0.942527000000
C	–3.628365000000	5.128369000000	–0.651695000000
C	–1.798651000000	3.871632000000	0.599384000000
C	–1.210043000000	3.326758000000	1.750551000000
C	0.058771000000	2.742656000000	1.739525000000
C	0.760286000000	2.707225000000	0.521934000000
C	0.196988000000	3.196294000000	–0.663861000000
C	–1.069459000000	3.795443000000	–0.591588000000
C	0.848372000000	2.966796000000	–2.019891000000
O	2.035590000000	2.151658000000	0.502696000000
C	3.079979000000	3.083066000000	0.683477000000
H	–4.330371000000	5.911559000000	1.841287000000
H	–2.607601000000	6.318756000000	1.632825000000
H	–3.098560000000	5.051496000000	2.782835000000
H	–5.231599000000	3.660530000000	1.008941000000
H	–3.964071000000	2.773594000000	1.886449000000
H	–4.160862000000	2.534286000000	0.139180000000
H	–2.945756000000	5.950428000000	–0.917195000000
H	–4.638915000000	5.551405000000	–0.552273000000
H	–3.656635000000	4.416374000000	–1.490371000000
H	–1.760582000000	3.345562000000	2.691329000000
H	–1.496531000000	4.196143000000	–1.508444000000
H	1.923361000000	2.791383000000	–1.908158000000
H	0.705598000000	3.861282000000	–2.644229000000
H	2.851466000000	3.770601000000	1.521567000000
H	3.231001000000	3.704200000000	–0.216353000000
C	4.374817000000	2.370889000000	1.015814000000
O	4.391111000000	1.180906000000	1.273193000000
C	5.616080000000	3.213611000000	0.988752000000
H	6.401483000000	2.761488000000	1.608016000000
H	5.427067000000	4.253350000000	1.294734000000
H	5.956177000000	3.225643000000	–0.060597000000
C	–3.236275000000	0.957403000000	–4.236794000000
C	–3.263594000000	2.004703000000	–5.368499000000
C	–4.108476000000	1.441794000000	–3.057186000000
C	–3.831607000000	–0.355476000000	–4.767361000000
C	–1.795839000000	0.777306000000	–3.737736000000
C	–1.089685000000	1.878623000000	–3.232955000000
C	0.198429000000	1.770601000000	–2.702420000000
C	0.811642000000	0.505329000000	–2.713017000000
C	0.138436000000	–0.629246000000	–3.187997000000
C	–1.154623000000	–0.464263000000	–3.705182000000
C	0.698261000000	–2.035604000000	–3.022542000000
O	2.102631000000	0.389928000000	–2.210053000000
C	3.136305000000	0.379061000000	–3.170593000000
H	–4.292888000000	2.144373000000	–5.733990000000
H	–2.639984000000	1.683814000000	–6.217338000000
H	–2.895917000000	2.985441000000	–5.030961000000
H	–5.147708000000	1.595008000000	–3.387528000000
H	–3.745625000000	2.395716000000	–2.646070000000
H	–4.119210000000	0.703153000000	–2.241025000000
H	–3.252766000000	–0.754376000000	–5.614777000000
H	–4.858711000000	–0.180770000000	–5.120511000000
H	–3.880898000000	–1.126612000000	–3.984090000000
H	–1.566172000000	2.858748000000	–3.232336000000
H	–1.674366000000	–1.347734000000	–4.069475000000
H	1.783024000000	–1.999878000000	–2.881107000000
H	0.492779000000	–2.616937000000	–3.933273000000
H	2.821524000000	0.910298000000	–4.088993000000
H	3.400912000000	–0.651327000000	–3.465211000000
C	4.377943000000	1.084395000000	–2.649952000000
O	4.324990000000	1.834761000000	–1.697986000000
C	5.654192000000	0.783367000000	–3.385309000000
H	6.387554000000	1.583669000000	–3.223747000000
H	5.488410000000	0.621455000000	–4.461379000000
H	6.051431000000	–0.154812000000	–2.961750000000
C	–3.357499000000	–4.363568000000	–1.050686000000
C	–3.156552000000	–5.763545000000	–1.670425000000
C	–4.216276000000	–3.501288000000	–1.999036000000
C	–4.116543000000	–4.505937000000	0.278731000000
C	–1.972524000000	–3.730001000000	–0.852428000000
C	–1.247099000000	–3.255546000000	–1.955116000000
C	0.033820000000	–2.709750000000	–1.832107000000
C	0.620967000000	–2.679959000000	–0.556603000000
C	–0.076332000000	–3.105980000000	0.581240000000
C	–1.367179000000	–3.624943000000	0.405099000000
C	0.489637000000	–2.928082000000	1.982687000000
O	1.922584000000	–2.200377000000	–0.434533000000
C	2.917140000000	–3.194102000000	–0.554116000000
H	–4.129118000000	–6.255046000000	–1.831986000000
H	–2.554195000000	–6.405160000000	–1.008479000000
H	–2.641409000000	–5.702789000000	–2.641842000000
H	–5.212351000000	–3.955414000000	–2.114565000000
H	–3.779101000000	–3.427122000000	–3.005918000000
H	–4.349719000000	–2.487156000000	–1.595922000000
H	–3.606479000000	–5.188190000000	0.975877000000
H	–5.115434000000	–4.926442000000	0.089370000000
H	–4.249571000000	–3.529419000000	0.767043000000
H	–1.693963000000	–3.302156000000	–2.948114000000
H	–1.908144000000	–3.958371000000	1.288123000000
H	1.579555000000	–2.828938000000	1.942880000000
H	0.245215000000	–3.818264000000	2.580925000000
H	2.696103000000	–3.869324000000	–1.404124000000
H	2.975154000000	–3.820823000000	0.352643000000
C	4.272210000000	–2.568303000000	–0.810167000000
O	4.381771000000	–1.383964000000	–1.071836000000
C	5.452549000000	–3.489045000000	–0.704272000000
H	6.302523000000	–3.092166000000	–1.274167000000
H	5.215929000000	–4.516152000000	–1.019711000000
H	5.725438000000	–3.517605000000	0.364214000000

C	–3.455967000000	–0.680279000000	4.315157000000
C	–3.296964000000	–0.567390000000	5.846982000000
C	–4.172124000000	–1.998736000000	3.980332000000
C	–4.333021000000	0.480097000000	3.802353000000
C	–2.055103000000	–0.616161000000	3.688746000000
C	–1.419561000000	–1.742244000000	3.153598000000
C	–0.112098000000	–1.701387000000	2.649915000000
C	0.576861000000	–0.480660000000	2.695930000000
C	–0.044316000000	0.692425000000	3.156704000000
C	–1.344055000000	0.592598000000	3.663255000000
C	0.597757000000	2.061151000000	2.988942000000
O	1.897069000000	–0.440056000000	2.257763000000
C	2.869280000000	–0.482681000000	3.280449000000
H	–4.281549000000	–0.611836000000	6.338991000000
H	–2.817649000000	0.381899000000	6.132882000000
H	–2.679557000000	–1.389559000000	6.241385000000
H	–5.183006000000	–1.989544000000	4.414410000000
H	–3.648637000000	–2.872005000000	4.398734000000
H	–4.274846000000	–2.131449000000	2.893263000000
H	–3.918358000000	1.464601000000	4.066082000000
H	–5.333074000000	0.419821000000	4.258544000000
H	–4.453198000000	0.428948000000	2.711164000000
H	–1.948828000000	–2.692078000000	3.123241000000
H	–1.816640000000	1.497446000000	4.045838000000
H	1.686434000000	1.967818000000	2.922687000000
H	0.363831000000	2.680738000000	3.866887000000
H	2.465852000000	–0.984901000000	4.180168000000
H	3.177051000000	0.532518000000	3.586386000000
C	4.097913000000	–1.264346000000	2.846904000000
O	4.070283000000	–2.005222000000	1.886656000000
C	5.331741000000	–1.049592000000	3.679277000000
H	6.018447000000	–1.898866000000	3.571370000000
H	5.096094000000	–0.874745000000	4.740114000000
H	5.824569000000	–0.142485000000	3.289977000000
C	–3.009103000000	–0.416265000000	0.204880000000
N	–4.058584000000	–0.844537000000	0.446123000000
C	–1.698376000000	0.125551000000	–0.105955000000
H	–1.056801000000	0.073846000000	0.781760000000
H	–1.784204000000	1.173416000000	–0.421054000000
H	–1.240215000000	–0.453931000000	–0.917218000000

K**L**$_{\mathbf{c}}$MeCN$^+$, zero imaginary frequencies
143
–3509.9524966

K	3.084561000000	0.003964000000	0.083492000000
C	–3.333877000000	3.646523000000	–2.454418000000
C	–3.460620000000	5.174036000000	–2.285295000000
C	–4.292040000000	2.933748000000	–1.475762000000
C	–3.742333000000	3.285729000000	–3.891116000000
C	–1.899855000000	3.201827000000	–2.133124000000
C	–1.302570000000	3.577259000000	–0.920496000000
C	–0.007274000000	3.188276000000	–0.567057000000
C	0.713470000000	2.395555000000	–1.479928000000
C	0.124818000000	1.913157000000	–2.659057000000
C	–1.165695000000	2.362064000000	–2.976942000000
C	0.777012000000	0.829209000000	–3.511720000000
O	2.031396000000	2.074875000000	–1.180678000000
C	3.007360000000	2.868374000000	–1.817769000000
H	–4.484614000000	5.498217000000	–2.527052000000
H	–2.766670000000	5.705292000000	–2.955224000000
H	–3.250361000000	5.495827000000	–1.254639000000
H	–5.332841000000	3.242166000000	–1.662366000000
H	–4.053594000000	3.168897000000	–0.429166000000
H	–4.237231000000	1.840543000000	–1.589575000000
H	–3.069972000000	3.741288000000	–4.634582000000
H	–4.760085000000	3.652599000000	–4.090320000000
H	–3.753876000000	2.197588000000	–4.054750000000
H	–1.865157000000	4.194088000000	–0.220192000000
H	–1.607629000000	2.024691000000	–3.911731000000
H	1.852345000000	0.776631000000	–3.312428000000
H	0.636058000000	1.072212000000	–4.575806000000
H	2.668165000000	3.919110000000	–1.906789000000
H	3.217279000000	2.508319000000	–2.841507000000
C	4.306148000000	2.878668000000	–1.026139000000
O	4.357975000000	2.472291000000	0.119595000000
C	5.513028000000	3.382136000000	–1.766043000000
H	6.286851000000	3.707981000000	–1.059335000000
H	5.266599000000	4.187393000000	–2.474499000000
H	5.901393000000	2.529537000000	–2.350451000000
C	–3.298276000000	–2.172638000000	–3.760690000000
C	–3.334117000000	–2.147753000000	–5.302361000000
C	–4.180677000000	–1.035054000000	–3.200999000000
C	–3.876075000000	–3.511818000000	–3.279195000000
C	–1.856868000000	–1.958738000000	–3.277274000000
C	–1.161458000000	–0.800288000000	–3.653090000000
C	0.136484000000	–0.524249000000	–3.215322000000
C	0.771591000000	–1.480163000000	–2.399696000000
C	0.095610000000	–2.622479000000	–1.943143000000
C	–1.206649000000	–2.846455000000	–2.415166000000
C	0.666488000000	–3.531318000000	–0.856984000000
O	2.087866000000	–1.252055000000	–2.021479000000
C	3.070307000000	–1.919758000000	–2.779719000000
H	–4.363581000000	–2.300483000000	–5.662121000000
H	–2.702766000000	–2.945263000000	–5.724033000000
H	–2.980990000000	–1.187522000000	–5.707526000000
H	–5.220726000000	–1.153396000000	–3.543054000000
H	–3.831200000000	–0.046548000000	–3.534357000000
H	–4.184500000000	–1.038696000000	–2.100335000000
H	–3.286036000000	–4.366754000000	–3.644276000000
H	–4.902101000000	–3.630591000000	–3.657845000000
H	–3.924178000000	–3.563791000000	–2.181628000000
H	–1.653270000000	–0.074859000000	–4.300297000000
H	–1.726321000000	–3.739039000000	–2.075126000000
H	1.746864000000	–3.380907000000	–0.761503000000
H	0.487139000000	–4.581367000000	–1.132770000000
H	2.767434000000	–1.998140000000	–3.842329000000
H	3.234604000000	–2.949215000000	–2.412521000000
C	4.392437000000	–1.168146000000	–2.740386000000
O	4.462610000000	–0.023004000000	–2.334765000000
C	5.595209000000	–1.945658000000	–3.194403000000
H	6.403526000000	–1.263985000000	–3.488250000000
H	5.359395000000	–2.646574000000	–4.009418000000
H	5.930117000000	–2.541886000000	–2.327441000000
C	–3.451789000000	–3.861857000000	2.105107000000
C	–3.272886000000	–5.310873000000	2.608456000000
C	–4.290192000000	–3.870506000000	0.810573000000
C	–4.220436000000	–3.053608000000	3.163422000000
C	–2.054669000000	–3.279362000000	1.843545000000
C	–1.317873000000	–3.679366000000	0.719266000000
C	–0.014722000000	–3.233133000000	0.474416000000
C	0.584888000000	–2.397606000000	1.433795000000
C	–0.137714000000	–1.902889000000	2.530417000000
C	–1.448844000000	–2.365261000000	2.713447000000
C	0.423656000000	–0.825210000000	3.452650000000
O	1.920703000000	–2.051941000000	1.265563000000
C	2.840462000000	–2.846309000000	1.980390000000
H	–4.253238000000	–5.771842000000	2.808614000000
H	–2.686387000000	–5.335057000000	3.540195000000
H	–2.750534000000	–5.932445000000	1.864443000000
H	–5.295673000000	–4.265788000000	1.021292000000
H	–3.850155000000	–4.513341000000	0.033861000000
H	–4.402580000000	–2.854045000000	0.407146000000
H	–3.726761000000	–3.083734000000	4.146792000000
H	–5.225606000000	–3.481240000000	3.294817000000
H	–4.338683000000	–2.004931000000	2.854170000000
H	–1.771712000000	–4.361994000000	0.001736000000
H	–2.005771000000	–1.997224000000	3.572089000000
H	1.513622000000	–0.776168000000	3.358701000000
H	0.180947000000	–1.084682000000	4.494139000000
H	2.492939000000	–3.896273000000	2.042316000000
H	2.964930000000	–2.485424000000	3.017713000000
C	4.200955000000	–2.862732000000	1.301228000000
O	4.351741000000	–2.461844000000	0.162067000000
C	5.339147000000	–3.367510000000	2.142457000000
H	6.169587000000	–3.696807000000	1.505006000000
H	5.030768000000	–4.170834000000	2.828507000000
H	5.678360000000	–2.515173000000	2.756972000000

C	–3.518798000000	2.418848000000	3.644012000000
C	–3.347868000000	3.534990000000	4.697620000000
C	–4.239458000000	1.224987000000	4.290861000000
C	–4.399210000000	2.934264000000	2.487992000000
C	–2.120747000000	2.035905000000	3.135459000000
C	–1.490715000000	0.841300000000	3.503004000000
C	–0.175254000000	0.537239000000	3.125991000000
C	0.528325000000	1.484705000000	2.365159000000
C	–0.098278000000	2.645014000000	1.875663000000
C	–1.406347000000	2.906296000000	2.298836000000
C	0.549580000000	3.525781000000	0.813004000000
O	1.868068000000	1.247717000000	2.081644000000
C	2.784040000000	1.918984000000	2.917834000000
H	–4.328565000000	3.839248000000	5.096613000000
H	–2.866466000000	4.425867000000	4.264940000000
H	–2.727028000000	3.191233000000	5.539731000000
H	–5.247692000000	1.530680000000	4.607738000000
H	–3.715058000000	0.861220000000	5.187750000000
H	–4.349103000000	0.392809000000	3.580084000000
H	–3.977884000000	3.831325000000	2.010008000000
H	–5.392667000000	3.211788000000	2.872625000000
H	–4.534995000000	2.157261000000	1.723076000000
H	–2.029874000000	0.117331000000	4.109622000000
H	–1.882141000000	3.826041000000	1.958980000000
H	1.635978000000	3.388420000000	0.810619000000
H	0.337262000000	4.581337000000	1.039617000000
H	2.389908000000	2.002746000000	3.949572000000
H	2.978764000000	2.947089000000	2.561771000000
C	4.106486000000	1.172683000000	3.001660000000
O	4.220414000000	0.026785000000	2.608336000000
C	5.258582000000	1.957629000000	3.562500000000
H	6.037713000000	1.280404000000	3.934986000000
H	4.945097000000	2.661988000000	4.347774000000
H	5.671665000000	2.549968000000	2.727226000000
C	–3.044238000000	–0.139616000000	0.421301000000
N	–4.108100000000	–0.255920000000	0.866023000000
C	–1.717448000000	0.003508000000	–0.149633000000
H	–1.083291000000	0.589288000000	0.526096000000
H	–1.774465000000	0.517486000000	–1.117528000000
H	–1.268735000000	–0.986403000000	–0.298171000000

Rb**L**$_{\mathbf{c}}$MeCN$^+$, zero imaginary frequencies

143

–2934.18502764

Rb	3.294976000000	–0.043788000000	0.052357000000
C	–3.447668000000	4.368121000000	0.027556000000
C	–3.565886000000	5.541311000000	1.021734000000
C	–4.406081000000	3.233206000000	0.447479000000
C	–3.863408000000	4.872502000000	–1.363068000000
C	–2.013466000000	3.820053000000	0.037962000000
C	–1.412276000000	3.448233000000	1.249706000000
C	–0.109532000000	2.946308000000	1.321222000000
C	0.613899000000	2.822096000000	0.120108000000
C	0.017116000000	3.071997000000	–1.125363000000
C	–1.280315000000	3.605770000000	–1.133636000000
C	0.677361000000	2.658739000000	–2.436940000000
O	1.953307000000	2.456236000000	0.184871000000
C	2.851088000000	3.543877000000	0.149056000000
H	–4.589508000000	5.946552000000	1.009911000000
H	–2.872846000000	6.355275000000	0.757475000000
H	–3.347754000000	5.232080000000	2.054612000000
H	–5.445905000000	3.595834000000	0.469448000000
H	–4.164277000000	2.844787000000	1.446507000000
H	–4.356868000000	2.388435000000	–0.255841000000
H	–3.193487000000	5.666545000000	–1.727821000000
H	–4.881225000000	5.287357000000	–1.317795000000
H	–3.878005000000	4.062239000000	–2.107408000000
H	–1.976209000000	3.554128000000	2.175925000000
H	–1.726621000000	3.849310000000	–2.094904000000
H	1.749968000000	2.495941000000	–2.288729000000
H	0.547721000000	3.460356000000	–3.180123000000
H	2.388395000000	4.450942000000	0.582537000000
H	3.137499000000	3.796806000000	–0.890004000000
C	4.117357000000	3.261719000000	0.940153000000
O	4.248061000000	2.252511000000	1.606548000000
C	5.203073000000	4.298063000000	0.811462000000
H	5.858030000000	4.270844000000	1.692133000000
H	4.801041000000	5.311085000000	0.660803000000
H	5.807342000000	4.046089000000	–0.077542000000
C	–3.411640000000	0.327979000000	–4.318958000000
C	–3.456221000000	1.235017000000	–5.565433000000
C	–4.290760000000	0.934168000000	–3.202989000000
C	–3.986681000000	–1.045998000000	–4.693814000000
C	–1.968116000000	0.229226000000	–3.805183000000
C	–1.269682000000	1.396124000000	–3.463378000000
C	0.032878000000	1.376000000000	–2.957097000000
C	0.669490000000	0.125761000000	–2.837145000000
C	–0.012782000000	–1.074105000000	–3.092429000000
C	–1.319758000000	–0.991313000000	–3.595868000000
C	0.564520000000	–2.434027000000	–2.707415000000
O	2.006973000000	0.094762000000	–2.460488000000
C	2.918488000000	0.024181000000	–3.534124000000
H	–4.487392000000	1.311420000000	–5.944045000000
H	–2.825418000000	0.828690000000	–6.371333000000
H	–3.108452000000	2.255672000000	–5.346034000000
H	–5.332810000000	1.031847000000	–3.545601000000
H	–3.941414000000	1.935047000000	–2.908845000000
H	–4.288605000000	0.298684000000	–2.304421000000
H	–3.394038000000	–1.535752000000	–5.482028000000
H	–5.012301000000	–0.927746000000	–5.073763000000
H	–4.035689000000	–1.718259000000	–3.824909000000
H	–1.761915000000	2.360185000000	–3.584518000000
H	–1.842913000000	–1.918783000000	–3.814987000000
H	1.643277000000	–2.353645000000	–2.536523000000
H	0.393464000000	–3.149165000000	–3.526255000000
H	2.471196000000	0.433112000000	–4.459633000000
H	3.200442000000	–1.024109000000	–3.753160000000
C	4.189143000000	0.811922000000	–3.254859000000
O	4.318472000000	1.488439000000	–2.252305000000
C	5.280802000000	0.663540000000	–4.282685000000
H	5.936970000000	1.543641000000	–4.266144000000
H	4.884499000000	0.497178000000	–5.295452000000
H	5.883506000000	–0.221402000000	–4.013239000000
C	–3.571019000000	–4.338105000000	–0.442661000000
C	–3.401178000000	–5.820255000000	–0.842075000000
C	–4.405769000000	–3.612211000000	–1.517221000000
C	–4.338224000000	–4.261150000000	0.887534000000
C	–2.169699000000	–3.718479000000	–0.332170000000
C	–1.430701000000	–3.423299000000	–1.486807000000
C	–0.120440000000	–2.934268000000	–1.439811000000
C	0.484791000000	–2.798382000000	–0.177197000000
C	–0.242927000000	–2.990270000000	1.007616000000
C	–1.561412000000	–3.455222000000	0.900601000000
C	0.329654000000	–2.623990000000	2.372966000000
O	1.838247000000	–2.484325000000	–0.120746000000
C	2.678844000000	–3.606792000000	0.034483000000
H	–4.384483000000	–6.308073000000	–0.936027000000
H	–2.817886000000	–6.367963000000	–0.085430000000
H	–2.879330000000	–5.918435000000	–1.806848000000
H	–5.414311000000	–4.050742000000	–1.564330000000
H	–3.967117000000	–3.710258000000	–2.521258000000
H	–4.510777000000	–2.544113000000	–1.279219000000
H	–3.846381000000	–4.841267000000	1.683269000000
H	–5.345501000000	–4.684018000000	0.756657000000
H	–4.451356000000	–3.219485000000	1.221240000000
H	–1.887842000000	–3.578660000000	–2.463235000000
H	–2.120970000000	–3.624949000000	1.817448000000
H	1.417916000000	–2.523699000000	2.308380000000
H	0.098365000000	–3.427711000000	3.088500000000
H	2.231288000000	–4.501777000000	–0.438941000000
H	2.831746000000	–3.851699000000	1.102470000000
C	4.038513000000	–3.394227000000	–0.608262000000
O	4.279810000000	–2.422563000000	–1.299237000000
C	5.066734000000	–4.451392000000	–0.302716000000
H	5.836226000000	–4.469973000000	–1.085377000000
H	4.618011000000	–5.448419000000	–0.178366000000
H	5.547676000000	–4.183908000000	0.654371000000

C	–3.606100000000	–0.059401000000	4.384636000000
C	–3.424616000000	0.276306000000	5.881102000000
C	–4.322358000000	–1.413719000000	4.258931000000
C	–4.495676000000	1.011452000000	3.721997000000
C	–2.213085000000	–0.086645000000	3.737647000000
C	–1.583987000000	–1.279202000000	3.362322000000
C	–0.269659000000	–1.316858000000	2.875790000000
C	0.434241000000	–0.105287000000	2.784949000000
C	–0.194495000000	1.128188000000	3.033846000000
C	–1.501547000000	1.104883000000	3.533219000000
C	0.456209000000	2.451662000000	2.649508000000
O	1.785339000000	–0.137869000000	2.456165000000
C	2.650812000000	–0.094889000000	3.570074000000
H	–4.401388000000	0.301337000000	6.389886000000
H	–2.945968000000	1.258568000000	6.017652000000
H	–2.796930000000	–0.477655000000	6.381508000000
H	–5.327115000000	–1.341932000000	4.701318000000
H	–3.789694000000	–2.214747000000	4.794146000000
H	–4.440075000000	–1.707285000000	3.205527000000
H	–4.078017000000	2.024026000000	3.827136000000
H	–5.486428000000	1.020956000000	4.201860000000
H	–4.635660000000	0.796268000000	2.653738000000
H	–2.121618000000	–2.219613000000	3.457982000000
H	–1.976985000000	2.055476000000	3.774316000000
H	1.540908000000	2.330608000000	2.560335000000
H	0.254017000000	3.198583000000	3.431951000000
H	2.135904000000	–0.450646000000	4.482033000000
H	2.988648000000	0.939203000000	3.775484000000
C	3.884059000000	–0.963556000000	3.377387000000
O	4.031349000000	–1.668554000000	2.396765000000
C	4.923893000000	–0.855527000000	4.462679000000
H	5.530673000000	–1.769874000000	4.496398000000
H	4.482347000000	–0.649433000000	5.449045000000
H	5.587390000000	–0.008976000000	4.213482000000
C	–3.151339000000	–0.343231000000	0.279521000000
N	–4.216994000000	–0.676088000000	0.590563000000
C	–1.823989000000	0.080683000000	–0.126410000000
H	–1.183459000000	0.196983000000	0.755523000000
H	–1.878444000000	1.040890000000	–0.654625000000
H	–1.383515000000	–0.668865000000	–0.795237000000

Cs**L**$_{e}$MeCN^{+}, zero imaginary frequencies
143
–2930.22556179

Cs	3.528285000000	0.017975000000	0.113838000000
C	–3.566309000000	3.363483000000	–2.793055000000
C	–3.697279000000	4.899873000000	–2.765472000000
C	–4.526726000000	2.742472000000	–1.756177000000
C	–3.968625000000	2.871674000000	–4.192177000000
C	–2.131812000000	2.955388000000	–2.428432000000
C	–1.537480000000	3.445718000000	–1.256510000000
C	–0.235303000000	3.110022000000	–0.873506000000
C	0.495306000000	2.248204000000	–1.712943000000
C	–0.095104000000	1.642797000000	–2.833606000000
C	–1.391543000000	2.044381000000	–3.188523000000
C	0.570042000000	0.487804000000	–3.575036000000
O	1.839575000000	2.022994000000	–1.434875000000
C	2.725269000000	2.812718000000	–2.200367000000
H	–4.721325000000	5.197874000000	–3.038734000000
H	–3.002514000000	5.369252000000	–3.479403000000
H	–3.490652000000	5.315199000000	–1.768060000000
H	–5.567254000000	3.031517000000	–1.973115000000
H	–4.291291000000	3.073138000000	–0.735134000000
H	–4.470998000000	1.643686000000	–1.768195000000
H	–3.295080000000	3.260079000000	–4.971843000000
H	–4.987011000000	3.214935000000	–4.427193000000
H	–3.975533000000	1.773215000000	–4.256231000000
H	–2.105601000000	4.119542000000	–0.616093000000
H	–1.831183000000	1.616230000000	–4.086240000000
H	1.642641000000	0.457466000000	–3.358534000000
H	0.439097000000	0.625893000000	–4.659290000000
H	2.200432000000	3.679826000000	–2.640656000000
H	3.145916000000	2.230935000000	–3.044166000000
C	3.889734000000	3.340921000000	–1.378083000000
O	4.007339000000	3.095537000000	–0.192543000000
C	4.914789000000	4.136535000000	–2.146164000000
H	5.464532000000	4.798396000000	–1.464227000000
H	4.467192000000	4.715657000000	–2.967374000000
H	5.633643000000	3.429109000000	–2.595396000000
C	–3.513270000000	–2.513767000000	–3.536564000000
C	–3.547080000000	–2.621272000000	–5.074740000000
C	–4.399239000000	–1.334593000000	–3.078292000000
C	–4.088401000000	–3.808502000000	–2.943370000000
C	–2.072999000000	–2.255814000000	–3.071463000000
C	–1.374799000000	–1.139738000000	–3.553719000000
C	–0.072672000000	–0.830012000000	–3.150812000000
C	0.564566000000	–1.712231000000	–2.257419000000
C	–0.119388000000	–2.797021000000	–1.685764000000
C	–1.425330000000	–3.057648000000	–2.127593000000
C	0.456177000000	–3.595831000000	–0.519361000000
O	1.906566000000	–1.505220000000	–1.958039000000
C	2.800807000000	–2.294676000000	–2.712865000000
H	–4.575494000000	–2.807970000000	–5.421306000000
H	–2.912298000000	–3.449695000000	–5.425879000000
H	–3.196909000000	–1.698040000000	–5.560428000000
H	–5.438042000000	–1.482617000000	–3.412650000000
H	–4.048997000000	–0.377670000000	–3.492897000000
H	–4.406924000000	–1.244454000000	–1.981488000000
H	–3.493640000000	–4.689365000000	–3.231038000000
H	–5.112624000000	–3.963425000000	–3.313878000000
H	–4.140565000000	–3.765796000000	–1.845746000000
H	–1.865930000000	–0.477830000000	–4.265825000000
H	–1.947648000000	–3.911257000000	–1.702788000000
H	1.534253000000	–3.425625000000	–0.436072000000
H	0.284464000000	–4.669274000000	–0.691397000000
H	2.299941000000	–2.718699000000	–3.601916000000
H	3.172260000000	–3.149932000000	–2.114851000000
C	4.009964000000	–1.504788000000	–3.188677000000
O	4.150116000000	–0.323430000000	–2.934353000000
C	5.046237000000	–2.299801000000	–3.942104000000
H	5.645914000000	–1.631918000000	–4.574248000000
H	4.600181000000	–3.103886000000	–4.545976000000
H	5.718180000000	–2.774932000000	–3.206070000000
C	–3.685087000000	–3.626554000000	2.429434000000
C	–3.507253000000	–5.015615000000	3.080623000000
C	–4.514670000000	–3.771323000000	1.137524000000
C	–4.462523000000	–2.715477000000	3.393597000000
C	–2.286559000000	–3.071673000000	2.119520000000
C	–1.542645000000	–3.579238000000	1.044905000000
C	–0.232776000000	–3.168853000000	0.772022000000
C	0.366664000000	–2.254692000000	1.657162000000
C	–0.366944000000	–1.646806000000	2.688214000000
C	–1.684115000000	–2.078570000000	2.900318000000
C	0.198606000000	–0.489553000000	3.504446000000
O	1.724181000000	–1.981133000000	1.518008000000
C	2.553179000000	–2.752615000000	2.362241000000
H	–4.488100000000	–5.456308000000	3.320372000000
H	–2.927389000000	–4.941992000000	4.013965000000
H	–2.978158000000	–5.708841000000	2.408073000000
H	–5.521634000000	–4.143061000000	1.381516000000
H	–4.069290000000	–4.491639000000	0.435484000000
H	–4.624213000000	–2.802763000000	0.629242000000
H	–3.974923000000	–2.641798000000	4.377686000000
H	–5.467418000000	–3.130432000000	3.562697000000
H	–4.581235000000	–1.705178000000	2.976055000000
H	–1.994206000000	–4.325687000000	0.392801000000
H	–2.246749000000	–1.626008000000	3.713387000000
H	1.287201000000	–0.448357000000	3.397407000000
H	–0.038937000000	–0.647990000000	4.567472000000
H	2.000036000000	–3.614384000000	2.777025000000
H	2.903630000000	–2.151039000000	3.223825000000
C	3.782549000000	–3.286914000000	1.645238000000
O	3.999537000000	–3.053527000000	0.471118000000
C	4.742081000000	–4.070688000000	2.505128000000
H	5.355259000000	–4.730847000000	1.877848000000
H	4.227721000000	–4.650195000000	3.285985000000
H	5.412370000000	–3.355776000000	3.013648000000

C	–3.758154000000	2.739512000000	3.376420000000
C	–3.588311000000	3.951558000000	4.318397000000
C	–4.474598000000	1.611532000000	4.136370000000
C	–4.642426000000	3.140287000000	2.178831000000
C	–2.359622000000	2.313528000000	2.904678000000
C	–1.725410000000	1.161858000000	3.383828000000
C	–0.404547000000	0.832302000000	3.047387000000
C	0.300816000000	1.711598000000	2.210448000000
C	–0.333590000000	2.811456000000	1.603838000000
C	–1.646527000000	3.103018000000	1.990482000000
C	0.320030000000	3.585940000000	0.465461000000
O	1.660674000000	1.502382000000	2.004011000000
C	2.494078000000	2.310763000000	2.809237000000
H	–4.568975000000	4.290435000000	4.688638000000
H	–3.109830000000	4.798132000000	3.801660000000
H	–2.965146000000	3.690997000000	5.188255000000
H	–5.483023000000	1.943764000000	4.424792000000
H	–3.947402000000	1.336453000000	5.062777000000
H	–4.583576000000	0.714676000000	3.509168000000
H	–4.222830000000	3.987016000000	1.615155000000
H	–5.634930000000	3.453385000000	2.537868000000
H	–4.779577000000	2.292726000000	1.493320000000
H	–2.263403000000	0.496432000000	4.054812000000
H	–2.125170000000	3.983525000000	1.562394000000
H	1.405519000000	3.444842000000	0.478364000000
H	0.108505000000	4.659225000000	0.585645000000
H	1.916703000000	2.782521000000	3.624027000000
H	2.938126000000	3.131360000000	2.212245000000
C	3.640905000000	1.530247000000	3.432904000000
O	3.809532000000	0.345116000000	3.216335000000
C	4.588530000000	2.341154000000	4.280668000000
H	5.147947000000	1.680262000000	4.955396000000
H	4.069979000000	3.124972000000	4.852640000000
H	5.307765000000	2.845144000000	3.611588000000
C	–3.277816000000	–0.096914000000	0.415668000000
N	–4.346481000000	–0.165002000000	0.859026000000
C	–1.946301000000	–0.015932000000	–0.155655000000
H	–1.321263000000	0.652185000000	0.447966000000
H	–1.997090000000	0.374821000000	–1.179678000000
H	–1.490475000000	–1.013282000000	–0.178002000000

Mg**L**$_c$MeCN^{2+}, zero imaginary frequencies
143
–3109.8961597

Mg	2.602053000000	–0.065125000000	0.062287000000
C	–3.034846000000	4.811343000000	0.247097000000
C	–2.996390000000	5.998698000000	1.230921000000
C	–4.089371000000	3.781080000000	0.710433000000
C	–3.440127000000	5.336729000000	–1.138412000000
C	–1.669778000000	4.112059000000	0.221716000000
C	–1.095465000000	3.649843000000	1.417005000000
C	0.112922000000	2.950749000000	1.447154000000
C	0.755957000000	2.706830000000	0.225789000000
C	0.235646000000	3.149605000000	–0.990751000000
C	–0.974705000000	3.857182000000	–0.965198000000
C	0.860326000000	2.761687000000	–2.316026000000
O	2.004720000000	2.039095000000	0.240389000000
C	3.088385000000	2.942521000000	0.443738000000
H	–3.972314000000	6.506709000000	1.246021000000
H	–2.234330000000	6.735232000000	0.933158000000
H	–2.778207000000	5.679533000000	2.260951000000
H	–5.085385000000	4.247699000000	0.751506000000
H	–3.864293000000	3.383136000000	1.710714000000
H	–4.144509000000	2.929291000000	0.013850000000
H	–2.713899000000	6.065339000000	–1.530320000000
H	–4.413536000000	5.843424000000	–1.071225000000
H	–3.549283000000	4.522734000000	–1.871945000000
H	–1.609440000000	3.830641000000	2.361517000000
H	–1.380335000000	4.207565000000	–1.912271000000
H	1.939978000000	2.611254000000	–2.210726000000
H	0.698694000000	3.569126000000	–3.043516000000
H	2.844537000000	3.649820000000	1.256087000000
H	3.290320000000	3.532707000000	–0.464618000000
C	4.315790000000	2.167522000000	0.829490000000
O	4.226617000000	0.950133000000	0.967594000000
C	5.595254000000	2.904398000000	1.010852000000
H	6.285389000000	2.336259000000	1.647680000000
H	5.445395000000	3.923286000000	1.395932000000
H	6.043735000000	2.989001000000	0.004772000000
C	–3.261830000000	0.557297000000	–4.239155000000
C	–3.268388000000	1.442204000000	–5.502817000000
C	–4.118417000000	1.216791000000	–3.134588000000
C	–3.883533000000	–0.804864000000	–4.583100000000
C	–1.824178000000	0.418484000000	–3.722768000000
C	–1.093906000000	1.560664000000	–3.362193000000
C	0.189364000000	1.494723000000	–2.814241000000
C	0.762946000000	0.226757000000	–2.638318000000
C	0.094445000000	–0.940909000000	–3.020503000000
C	–1.196808000000	–0.819007000000	–3.552448000000
C	0.654205000000	–2.328770000000	–2.772087000000
O	2.073170000000	0.142132000000	–2.106590000000
C	3.080235000000	0.095521000000	–3.119645000000
H	–4.294709000000	1.545919000000	–5.886177000000
H	–2.650998000000	0.999025000000	–6.299193000000
H	–2.887643000000	2.454912000000	–5.301244000000
H	–5.151163000000	1.353990000000	–3.489061000000
H	–3.732082000000	2.207541000000	–2.849453000000
H	–4.155825000000	0.586915000000	–2.232237000000
H	–3.325359000000	–1.321151000000	–5.379282000000
H	–4.911933000000	–0.660655000000	–4.944417000000
H	–3.935372000000	–1.466261000000	–3.704372000000
H	–1.546080000000	2.543854000000	–3.493121000000
H	–1.722840000000	–1.731501000000	–3.825297000000
H	1.745560000000	–2.293062000000	–2.679979000000
H	0.419392000000	–2.971676000000	–3.631444000000
H	2.670186000000	0.507740000000	–4.057466000000
H	3.378198000000	–0.945481000000	–3.329200000000
C	4.300029000000	0.915030000000	–2.749412000000
O	4.280269000000	1.640285000000	–1.775626000000
C	5.494668000000	0.763291000000	–3.643512000000
H	6.141245000000	1.646978000000	–3.566987000000
H	5.214335000000	0.580608000000	–4.691722000000
H	6.066182000000	–0.114900000000	–3.295234000000
C	–3.228752000000	–4.761074000000	–0.604886000000
C	–2.901090000000	–6.179960000000	–1.120385000000
C	–4.151559000000	–4.043723000000	–1.613002000000
C	–3.974778000000	–4.869892000000	0.734582000000
C	–1.906720000000	–3.996792000000	–0.458427000000
C	–1.207401000000	–3.573592000000	–1.599751000000
C	0.016015000000	–2.904586000000	–1.524252000000
C	0.557015000000	–2.673491000000	–0.253637000000
C	–0.094395000000	–3.068244000000	0.915136000000
C	–1.329693000000	–3.718656000000	0.786543000000
C	0.452428000000	–2.716909000000	2.283884000000
O	1.850355000000	–2.095016000000	–0.168147000000
C	2.872359000000	–3.080498000000	–0.302260000000
H	–3.827239000000	–6.763247000000	–1.238625000000
H	–2.247436000000	–6.717241000000	–0.415760000000
H	–2.395091000000	–6.149431000000	–2.097965000000
H	–5.101995000000	–4.591471000000	–1.697358000000
H	–3.716706000000	–4.001086000000	–2.623113000000
H	–4.374778000000	–3.020859000000	–1.278005000000
H	–3.411196000000	–5.456292000000	1.476541000000
H	–4.933513000000	–5.385796000000	0.580185000000
H	–4.192011000000	–3.875349000000	1.151738000000
H	–1.628287000000	–3.767314000000	–2.586592000000
H	–1.845813000000	–4.019719000000	1.696188000000
H	1.546066000000	–2.656041000000	2.257686000000
H	0.175320000000	–3.506712000000	2.995507000000
H	2.620352000000	–3.776594000000	–1.121447000000
H	2.973982000000	–3.674994000000	0.620062000000
C	4.178019000000	–2.410767000000	–0.620929000000
O	4.198295000000	–1.190653000000	–0.764414000000
C	5.400563000000	–3.251636000000	–0.730495000000
H	6.167559000000	–2.747922000000	–1.332737000000
H	5.188267000000	–4.258441000000	–1.117846000000
H	5.788320000000	–3.363191000000	0.297947000000

C	–3.485060000000	–0.162767000000	4.257958000000
C	–3.358909000000	0.153025000000	5.764688000000
C	–4.204878000000	–1.509593000000	4.082481000000
C	–4.330631000000	0.929839000000	3.570705000000
C	–2.072949000000	–0.188512000000	3.659340000000
C	–1.446966000000	–1.372662000000	3.254905000000
C	–0.139825000000	–1.400732000000	2.750117000000
C	0.543718000000	–0.186371000000	2.625867000000
C	–0.041459000000	1.030203000000	3.004662000000
C	–1.338950000000	0.999551000000	3.523790000000
C	0.624942000000	2.368995000000	2.751702000000
O	1.887237000000	–0.199464000000	2.171545000000
C	2.825271000000	–0.226269000000	3.250182000000
H	–4.355810000000	0.180975000000	6.230720000000
H	–2.878971000000	1.128980000000	5.937118000000
H	–2.762806000000	–0.614907000000	6.281458000000
H	–5.222200000000	–1.437755000000	4.493624000000
H	–3.697215000000	–2.323103000000	4.623058000000
H	–4.290985000000	–1.782767000000	3.020230000000
H	–3.918178000000	1.937805000000	3.728224000000
H	–5.347597000000	0.930410000000	3.990399000000
H	–4.412223000000	0.748299000000	2.489156000000
H	–1.983847000000	–2.315715000000	3.332911000000
H	–1.792033000000	1.943863000000	3.826025000000
H	1.715121000000	2.260184000000	2.730508000000
H	0.381114000000	3.055815000000	3.573649000000
H	2.321838000000	–0.600383000000	4.157676000000
H	3.189475000000	0.789388000000	3.480381000000
C	3.999849000000	–1.137497000000	2.960136000000
O	4.002572000000	–1.834849000000	1.965921000000
C	5.124262000000	–1.107571000000	3.952175000000
H	5.699718000000	–2.040939000000	3.901226000000
H	4.773854000000	–0.926590000000	4.979296000000
H	5.794323000000	–0.273752000000	3.678430000000
C	–3.012627000000	–0.539001000000	0.202552000000
N	–3.934191000000	–1.195374000000	0.449785000000
C	–1.850382000000	0.280041000000	–0.098463000000
H	–1.309812000000	0.504216000000	0.828860000000
H	–2.153787000000	1.225989000000	–0.565538000000
H	–1.189652000000	–0.257956000000	–0.790426000000

Ca**L**$_{c}$MeCN^{2+}, zero imaginary frequencies
143
–3587.45445169

Ca	2.871483000000	–0.012168000000	0.078650000000
C	–3.264853000000	3.916895000000	–2.168165000000
C	–3.344992000000	5.435930000000	–1.912647000000
C	–4.233309000000	3.175753000000	–1.219403000000
C	–3.692276000000	3.644282000000	–3.618429000000
C	–1.844007000000	3.414280000000	–1.881789000000
C	–1.236665000000	3.689130000000	–0.646454000000
C	0.036461000000	3.220579000000	–0.311707000000
C	0.717980000000	2.447734000000	–1.266171000000
C	0.143965000000	2.110231000000	–2.496256000000
C	–1.128951000000	2.621937000000	–2.786118000000
C	0.788301000000	1.113626000000	–3.446711000000
O	2.034311000000	2.031425000000	–0.984151000000
C	3.019264000000	2.995932000000	–1.333330000000
H	–4.360290000000	5.800425000000	–2.129331000000
H	–2.641866000000	5.984638000000	–2.558068000000
H	–3.122175000000	5.695258000000	–0.866976000000
H	–5.265845000000	3.519893000000	–1.383888000000
H	–3.986228000000	3.351026000000	–0.162647000000
H	–4.209190000000	2.088536000000	–1.393107000000
H	–3.018994000000	4.127698000000	–4.343049000000
H	–4.702702000000	4.043582000000	–3.786848000000
H	–3.730672000000	2.566987000000	–3.841803000000
H	–1.776628000000	4.284707000000	0.089287000000
H	–1.570219000000	2.379817000000	–3.750483000000
H	1.870639000000	1.073244000000	–3.280622000000
H	0.619952000000	1.439983000000	–4.482835000000
H	2.611481000000	4.016072000000	–1.223939000000
H	3.330751000000	2.883996000000	–2.388277000000
C	4.231097000000	2.859881000000	–0.439579000000
O	4.248840000000	1.999090000000	0.429421000000
C	5.381884000000	3.779431000000	–0.691370000000
H	5.999452000000	3.875914000000	0.210976000000
H	5.062262000000	4.766505000000	–1.055664000000
H	6.002998000000	3.319417000000	–1.480982000000
C	–3.256124000000	–1.875822000000	–3.973662000000
C	–3.281016000000	–1.718536000000	–5.507845000000
C	–4.141158000000	–0.789553000000	–3.321197000000
C	–3.831480000000	–3.252562000000	–3.609460000000
C	–1.821515000000	–1.697032000000	–3.460357000000
C	–1.122399000000	–0.512949000000	–3.740613000000
C	0.157436000000	–0.257437000000	–3.243488000000
C	0.762733000000	–1.253094000000	–2.458515000000
C	0.107595000000	–2.447290000000	–2.139212000000
C	–1.179781000000	–2.646604000000	–2.659561000000
C	0.676121000000	–3.450065000000	–1.145759000000
O	2.077012000000	–1.039501000000	–1.999792000000
C	3.079416000000	–1.434877000000	–2.926318000000
H	–4.307024000000	–1.848409000000	–5.883917000000
H	–2.642962000000	–2.472935000000	–5.993259000000
H	–2.936930000000	–0.724021000000	–5.829632000000
H	–5.178749000000	–0.881044000000	–3.676534000000
H	–3.793925000000	0.225264000000	–3.568292000000
H	–4.151250000000	–0.889668000000	–2.224639000000
H	–3.236173000000	–4.072669000000	–4.039782000000
H	–4.852654000000	–3.343135000000	–4.006835000000
H	–3.892601000000	–3.395893000000	–2.520163000000
H	–1.599095000000	0.250197000000	–4.355135000000
H	–1.693454000000	–3.572670000000	–2.412138000000
H	1.762011000000	–3.328303000000	–1.063147000000
H	0.478455000000	–4.469567000000	–1.505906000000
H	2.718118000000	–1.302059000000	–3.961370000000
H	3.333341000000	–2.504365000000	–2.808039000000
C	4.326902000000	–0.601566000000	–2.737415000000
O	4.352924000000	0.262841000000	–1.871990000000
C	5.499675000000	–0.904430000000	–3.612819000000
H	6.160886000000	–0.030928000000	–3.682380000000
H	5.202108000000	–1.251860000000	–4.612745000000
H	6.066286000000	–1.722001000000	–3.131914000000
C	–3.405036000000	–4.106965000000	1.791111000000
C	–3.185862000000	–5.603046000000	2.108097000000
C	–4.263820000000	–3.971035000000	0.516324000000
C	–4.165520000000	–3.451481000000	2.954942000000
C	–2.026746000000	–3.466942000000	1.576776000000
C	–1.281672000000	–3.762090000000	0.424538000000
C	0.000079000000	–3.250194000000	0.203106000000
C	0.562846000000	–2.445406000000	1.206214000000
C	–0.148704000000	–2.088558000000	2.356314000000
C	–1.443001000000	–2.604720000000	2.512211000000
C	0.403000000000	–1.105121000000	3.376130000000
O	1.903670000000	–2.030894000000	1.063937000000
C	2.839897000000	–3.002775000000	1.512138000000
H	–4.153716000000	–6.102131000000	2.269742000000
H	–2.580149000000	–5.730750000000	3.018651000000
H	–2.672850000000	–6.121462000000	1.283069000000
H	–5.254174000000	–4.418398000000	0.687908000000
H	–3.821134000000	–4.495790000000	–0.343577000000
H	–4.410636000000	–2.914183000000	0.251721000000
H	–3.649397000000	–3.590981000000	3.917246000000
H	–5.157140000000	–3.916445000000	3.053188000000
H	–4.316394000000	–2.376109000000	2.779547000000
H	–1.714432000000	–4.413396000000	–0.334302000000
H	–2.000396000000	–2.329805000000	3.405033000000
H	1.497587000000	–1.084524000000	3.323339000000
H	0.123736000000	–1.441154000000	4.384646000000
H	2.431301000000	–4.018968000000	1.372717000000
H	3.050924000000	–2.885127000000	2.590925000000
C	4.132672000000	–2.892087000000	0.737529000000
O	4.247680000000	–2.039297000000	–0.132584000000
C	5.239786000000	–3.826355000000	1.104960000000
H	5.938778000000	–3.940271000000	0.266268000000
H	4.871445000000	–4.805261000000	1.444450000000
H	5.790022000000	–3.368733000000	1.946727000000

C	–3.473009000000	2.171890000000	3.854235000000
C	–3.273985000000	3.208548000000	4.982320000000
C	–4.200081000000	0.942192000000	4.421881000000
C	–4.353682000000	2.780459000000	2.743895000000
C	–2.086936000000	1.810825000000	3.303044000000
C	–1.470252000000	0.582092000000	3.570205000000
C	–0.171924000000	0.282283000000	3.138151000000
C	0.518911000000	1.260630000000	2.410277000000
C	–0.074533000000	2.481462000000	2.059249000000
C	–1.367535000000	2.734056000000	2.529423000000
C	0.584410000000	3.450167000000	1.090761000000
O	1.863976000000	1.020447000000	2.061901000000
C	2.778846000000	1.410760000000	3.078127000000
H	–4.246942000000	3.493852000000	5.411161000000
H	–2.787815000000	4.123952000000	4.610465000000
H	–2.651415000000	2.798432000000	5.792595000000
H	–5.200077000000	1.236424000000	4.771928000000
H	–3.672057000000	0.510822000000	5.286165000000
H	–4.329946000000	0.164387000000	3.654841000000
H	–3.932189000000	3.712994000000	2.339118000000
H	–5.344179000000	3.029374000000	3.152983000000
H	–4.496913000000	2.068584000000	1.918918000000
H	–2.008992000000	–0.173171000000	4.138281000000
H	–1.825396000000	3.692164000000	2.284532000000
H	1.672905000000	3.323504000000	1.107317000000
H	0.362347000000	4.481001000000	1.400733000000
H	2.317565000000	1.287433000000	4.073892000000
H	3.053040000000	2.477466000000	2.981197000000
C	4.031709000000	0.567861000000	3.020083000000
O	4.142468000000	–0.298228000000	2.162825000000
C	5.108871000000	0.862491000000	4.013492000000
H	5.748154000000	–0.018445000000	4.156292000000
H	4.712419000000	1.219449000000	4.974913000000
H	5.733502000000	1.669905000000	3.590764000000
C	–3.068365000000	–0.195422000000	0.475868000000
N	–4.082281000000	–0.394878000000	0.998986000000
C	–1.796935000000	0.044664000000	–0.184130000000
H	–1.125021000000	0.581815000000	0.496542000000
H	–1.943108000000	0.651098000000	–1.087176000000
H	–1.341522000000	–0.912659000000	–0.468651000000

Sr**L**$_c$MeCN^{2+}, zero imaginary frequencies
143
–2940.6447229

Sr	2.857743000000	–0.002262000000	0.075522000000
C	–3.401823000000	3.687066000000	–2.439758000000
C	–3.518054000000	5.214178000000	–2.257880000000
C	–4.358891000000	2.970614000000	–1.461275000000
C	–3.813450000000	3.336827000000	–3.878070000000
C	–1.971833000000	3.230127000000	–2.122567000000
C	–1.366383000000	3.602224000000	–0.912006000000
C	–0.078156000000	3.193890000000	–0.555971000000
C	0.621672000000	2.384487000000	–1.468245000000
C	0.038405000000	1.921489000000	–2.654285000000
C	–1.248448000000	2.382541000000	–2.968413000000
C	0.688531000000	0.851134000000	–3.522315000000
O	1.959776000000	2.051801000000	–1.184691000000
C	2.898677000000	3.020116000000	–1.632534000000
H	–4.538925000000	5.544589000000	–2.501221000000
H	–2.820656000000	5.747749000000	–2.921966000000
H	–3.313071000000	5.528291000000	–1.223691000000
H	–5.397448000000	3.286910000000	–1.643166000000
H	–4.117878000000	3.197840000000	–0.413164000000
H	–4.313221000000	1.877721000000	–1.585742000000
H	–3.140544000000	3.793337000000	–4.620322000000
H	–4.828122000000	3.712263000000	–4.073726000000
H	–3.835494000000	2.249610000000	–4.048686000000
H	–1.919211000000	4.231711000000	–0.215632000000
H	–1.694561000000	2.056862000000	–3.905207000000
H	1.769476000000	0.821373000000	–3.344400000000
H	0.530472000000	1.098867000000	–4.581903000000
H	2.467457000000	4.034782000000	–1.561523000000
H	3.162810000000	2.859272000000	–2.694689000000
C	4.157499000000	2.978666000000	–0.793107000000
O	4.249358000000	2.191404000000	0.139316000000
C	5.264393000000	3.908761000000	–1.177794000000
H	5.874820000000	4.158718000000	–0.299854000000
H	4.900551000000	4.820546000000	–1.672686000000
H	5.912809000000	3.373874000000	–1.894853000000
C	–3.376850000000	–2.150095000000	–3.794384000000
C	–3.410153000000	–2.098541000000	–5.335684000000
C	–4.256807000000	–1.020020000000	–3.214043000000
C	–3.952005000000	–3.497798000000	–3.334079000000
C	–1.938044000000	–1.940444000000	–3.304667000000
C	–1.236613000000	–0.783105000000	–3.675836000000
C	0.055772000000	–0.505447000000	–3.223821000000
C	0.672989000000	–1.461123000000	–2.396675000000
C	0.004046000000	–2.611294000000	–1.958833000000
C	–1.295354000000	–2.831407000000	–2.439763000000
C	0.573495000000	–3.535688000000	–0.888045000000
O	2.009064000000	–1.249871000000	–2.009356000000
C	2.968404000000	–1.733725000000	–2.938477000000
H	–4.438524000000	–2.251682000000	–5.696195000000
H	–2.776227000000	–2.885861000000	–5.771740000000
H	–3.066038000000	–1.129207000000	–5.726838000000
H	–5.296372000000	–1.133796000000	–3.556996000000
H	–3.909458000000	–0.025690000000	–3.533082000000
H	–4.261578000000	–1.043144000000	–2.113266000000
H	–3.359864000000	–4.346198000000	–3.710565000000
H	–4.975436000000	–3.613563000000	–3.718903000000
H	–4.007422000000	–3.566440000000	–2.237436000000
H	–1.721048000000	–0.060305000000	–4.331116000000
H	–1.817575000000	–3.726997000000	–2.112115000000
H	1.659300000000	–3.406211000000	–0.813988000000
H	0.378306000000	–4.580216000000	–1.170182000000
H	2.580267000000	–1.653494000000	–3.969684000000
H	3.191013000000	–2.802950000000	–2.762558000000
C	4.252069000000	–0.935974000000	–2.848295000000
O	4.341437000000	–0.002428000000	–2.062044000000
C	5.383632000000	–1.363083000000	–3.728740000000
H	6.035765000000	–0.508758000000	–3.953877000000
H	5.041518000000	–1.847725000000	–4.654366000000
H	5.981843000000	–2.101690000000	–3.165371000000
C	–3.529831000000	–3.911091000000	2.074657000000
C	–3.327462000000	–5.372507000000	2.533611000000
C	–4.376350000000	–3.890788000000	0.785016000000
C	–4.294310000000	–3.142538000000	3.164558000000
C	–2.142024000000	–3.308645000000	1.816495000000
C	–1.397951000000	–3.700645000000	0.692754000000
C	–0.101797000000	–3.237415000000	0.446300000000
C	0.477358000000	–2.388641000000	1.404867000000
C	–0.241735000000	–1.911773000000	2.507879000000
C	–1.549422000000	–2.385787000000	2.686573000000
C	0.315027000000	–0.848818000000	3.446630000000
O	1.834610000000	–2.039470000000	1.258635000000
C	2.730141000000	–3.000861000000	1.801005000000
H	–4.301327000000	–5.846586000000	2.730656000000
H	–2.732476000000	–5.418489000000	3.458909000000
H	–2.810077000000	–5.969846000000	1.766687000000
H	–5.375403000000	–4.302141000000	0.992247000000
H	–3.937113000000	–4.508725000000	–0.012393000000
H	–4.504142000000	–2.864938000000	0.410744000000
H	–3.789196000000	–3.196900000000	4.141149000000
H	–5.291578000000	–3.586908000000	3.295439000000
H	–4.432276000000	–2.086973000000	2.888326000000
H	–1.842947000000	–4.392147000000	–0.021770000000
H	–2.111832000000	–2.028826000000	3.546273000000
H	1.409008000000	–0.826318000000	3.380913000000
H	0.048920000000	–1.110656000000	4.480737000000
H	2.305345000000	–4.016209000000	1.706247000000
H	2.898162000000	–2.825248000000	2.880253000000
C	4.059967000000	–2.972992000000	1.079405000000
O	4.240776000000	–2.197484000000	0.149778000000
C	5.123493000000	–3.901399000000	1.574924000000
H	5.809858000000	–4.164902000000	0.759228000000
H	4.712420000000	–4.805395000000	2.046530000000
H	5.706504000000	–3.358972000000	2.340789000000

C	–3.591465000000	2.426063000000	3.667068000000
C	–3.394641000000	3.541059000000	4.718539000000
C	–4.312837000000	1.237506000000	4.322699000000
C	–4.477276000000	2.951068000000	2.519110000000
C	–2.202876000000	2.033627000000	3.143798000000
C	–1.577905000000	0.832739000000	3.503038000000
C	–0.268506000000	0.518945000000	3.114528000000
C	0.426774000000	1.462643000000	2.343974000000
C	–0.184189000000	2.635671000000	1.873924000000
C	–1.488523000000	2.902145000000	2.304487000000
C	0.470165000000	3.528912000000	0.828180000000
O	1.788984000000	1.235659000000	2.064339000000
C	2.660166000000	1.723491000000	3.076049000000
H	–4.368014000000	3.851402000000	5.128618000000
H	–2.913728000000	4.429830000000	4.281053000000
H	–2.768176000000	3.192298000000	5.554177000000
H	–5.313871000000	1.552011000000	4.651465000000
H	–3.782592000000	0.871747000000	5.215390000000
H	–4.440246000000	0.405816000000	3.614137000000
H	–4.057837000000	3.850488000000	2.043466000000
H	–5.465404000000	3.230663000000	2.913812000000
H	–4.625061000000	2.178973000000	1.751374000000
H	–2.118445000000	0.113694000000	4.114301000000
H	–1.959207000000	3.828677000000	1.977130000000
H	1.559085000000	3.405850000000	0.851274000000
H	0.247513000000	4.580390000000	1.059255000000
H	2.177225000000	1.646289000000	4.066636000000
H	2.895843000000	2.793039000000	2.919398000000
C	3.948518000000	0.930666000000	3.113933000000
O	4.120849000000	–0.002963000000	2.341558000000
C	4.984325000000	1.361530000000	4.103939000000
H	5.604157000000	0.506077000000	4.403966000000
H	4.550822000000	1.856927000000	4.984357000000
H	5.643093000000	2.090428000000	3.598549000000
C	–3.158388000000	–0.145913000000	0.463665000000
N	–4.188856000000	–0.279328000000	0.975234000000
C	–1.866799000000	0.016947000000	–0.179772000000
H	–1.212139000000	0.621052000000	0.460359000000
H	–1.983103000000	0.523780000000	–1.146048000000
H	–1.410786000000	–0.967017000000	–0.347330000000

Ba$\mathbf{L}_6$MeCN^{2+}, zero imaginary frequencies
143
–2935.38817574

Ba	2.940087000000	–0.018927000000	0.073605000000
C	–3.527513000000	4.150549000000	–1.442944000000
C	–3.655649000000	5.576362000000	–0.868898000000
C	–4.484429000000	3.198655000000	–0.692106000000
C	–3.929124000000	4.187767000000	–2.925687000000
C	–2.095201000000	3.636026000000	–1.247593000000
C	–1.492022000000	3.693420000000	0.018513000000
C	–0.192622000000	3.237071000000	0.257046000000
C	0.521758000000	2.713063000000	–0.836651000000
C	–0.069742000000	2.531279000000	–2.094791000000
C	–1.366875000000	3.032743000000	–2.278275000000
C	0.582793000000	1.706129000000	–3.200440000000
O	1.884293000000	2.408744000000	–0.666152000000
C	2.733946000000	3.524435000000	–0.896078000000
H	–4.677566000000	5.953171000000	–1.024675000000
H	–2.958539000000	6.269093000000	–1.365175000000
H	–3.458869000000	5.610172000000	0.212945000000
H	–5.524503000000	3.544666000000	–0.793661000000
H	–4.251168000000	3.148441000000	0.380665000000
H	–4.428771000000	2.175802000000	–1.094663000000
H	–3.253522000000	4.824232000000	–3.517849000000
H	–4.944233000000	4.598681000000	–3.023167000000
H	–3.945913000000	3.183387000000	–3.375463000000
H	–2.053689000000	4.113129000000	0.851855000000
H	–1.816758000000	2.942084000000	–3.263884000000
H	1.663275000000	1.627106000000	–3.032251000000
H	0.431510000000	2.209834000000	–4.166486000000
H	2.273189000000	4.447963000000	–0.500106000000
H	2.890726000000	3.689731000000	–1.978893000000
C	4.075042000000	3.340320000000	–0.216837000000
O	4.293195000000	2.350948000000	0.467375000000
C	5.101935000000	4.405040000000	–0.450873000000
H	5.790138000000	4.462101000000	0.402971000000
H	4.654225000000	5.387787000000	–0.657490000000
H	5.692220000000	4.113650000000	–1.338120000000
C	–3.507975000000	–1.115890000000	–4.170636000000
C	–3.539807000000	–0.696729000000	–5.654992000000
C	–4.381732000000	–0.152536000000	–3.336859000000
C	–4.091361000000	–2.531049000000	–4.045904000000
C	–2.066321000000	–1.039467000000	–3.650950000000
C	–1.361055000000	0.171107000000	–3.726902000000
C	–0.055965000000	0.317815000000	–3.250157000000
C	0.572051000000	–0.825661000000	–2.720252000000
C	–0.110138000000	–2.039055000000	–2.550885000000
C	–1.422390000000	–2.120023000000	–3.041053000000
C	0.461796000000	–3.203543000000	–1.744601000000
O	1.934766000000	–0.746567000000	–2.383962000000
C	2.802639000000	–1.005837000000	–3.478364000000
H	–4.568983000000	–0.751364000000	–6.040827000000
H	–2.911271000000	–1.361387000000	–6.267451000000
H	–3.187627000000	0.335414000000	–5.802534000000
H	–5.421347000000	–0.174352000000	–3.697393000000
H	–4.028292000000	0.886949000000	–3.409103000000
H	–4.388686000000	–0.437871000000	–2.273720000000
H	–3.505914000000	–3.267801000000	–4.617449000000
H	–5.116310000000	–2.544635000000	–4.443676000000
H	–4.144915000000	–2.860610000000	–2.997825000000
H	–1.851633000000	1.037995000000	–4.166812000000
H	–1.952665000000	–3.062614000000	–2.932722000000
H	1.547720000000	–3.095569000000	–1.638803000000
H	0.268541000000	–4.144723000000	–2.279585000000
H	2.375234000000	–0.602518000000	–4.414479000000
H	2.933780000000	–2.093624000000	–3.633554000000
C	4.157544000000	–0.364140000000	–3.262474000000
O	4.370125000000	0.314749000000	–2.267974000000
C	5.203813000000	–0.628135000000	–4.300954000000
H	5.916085000000	0.206685000000	–4.342303000000
H	4.775034000000	–0.824729000000	–5.294130000000
H	5.762854000000	–1.530226000000	–3.993924000000
C	–3.649612000000	–4.275428000000	1.039142000000
C	–3.446746000000	–5.804133000000	1.138246000000
C	–4.497656000000	–3.950900000000	–0.207589000000
C	–4.413420000000	–3.787778000000	2.280846000000
C	–2.260118000000	–3.631966000000	0.932604000000
C	–1.519759000000	–3.733991000000	–0.255384000000
C	–0.213077000000	–3.250043000000	–0.375367000000
C	0.381129000000	–2.696538000000	0.773293000000
C	–0.343039000000	–2.480542000000	1.953855000000
C	–1.660420000000	–2.959398000000	2.003779000000
C	0.219207000000	–1.681050000000	3.125487000000
O	1.758521000000	–2.407123000000	0.740613000000
C	2.564560000000	–3.533458000000	1.058989000000
H	–4.420336000000	–6.311810000000	1.218451000000
H	–2.850348000000	–6.068130000000	2.025363000000
H	–2.930277000000	–6.201784000000	0.250647000000
H	–5.495523000000	–4.402128000000	–0.103173000000
H	–4.058735000000	–4.360738000000	–1.129450000000
H	–4.627902000000	–2.865788000000	–0.325860000000
H	–3.909048000000	–4.073820000000	3.216582000000
H	–5.411407000000	–4.248954000000	2.302064000000
H	–4.549639000000	–2.696692000000	2.263748000000
H	–1.974696000000	–4.212327000000	–1.121605000000
H	–2.224422000000	–2.817088000000	2.922293000000
H	1.312726000000	–1.634962000000	3.059552000000
H	–0.036149000000	–2.193524000000	4.064486000000
H	2.117288000000	–4.455729000000	0.645238000000
H	2.630246000000	–3.679559000000	2.153921000000
C	3.959822000000	–3.392588000000	0.488063000000
O	4.257019000000	–2.422318000000	–0.194284000000
C	4.938998000000	–4.474722000000	0.824589000000
H	5.693339000000	–4.563504000000	0.031500000000
H	4.452987000000	–5.443196000000	1.011012000000
H	5.461021000000	–4.178869000000	1.752211000000

C	–3.721007000000	1.408411000000	4.112940000000
C	–3.533838000000	2.210626000000	5.420386000000
C	–4.442000000000	0.088512000000	4.430700000000
C	–4.603019000000	2.213079000000	3.137615000000
C	–2.326514000000	1.168840000000	3.517665000000
C	–1.694003000000	–0.079556000000	3.571399000000
C	–0.372603000000	–0.274753000000	3.147105000000
C	0.329161000000	0.843181000000	2.667993000000
C	–0.296225000000	2.086439000000	2.473957000000
C	–1.613025000000	2.223212000000	2.927913000000
C	0.357744000000	3.214662000000	1.682810000000
O	1.710794000000	0.721204000000	2.427861000000
C	2.491075000000	0.961422000000	3.591570000000
H	–4.510426000000	2.401325000000	5.891446000000
H	–3.053434000000	3.183526000000	5.232505000000
H	–2.910534000000	1.657044000000	6.139728000000
H	–5.446949000000	0.303934000000	4.821474000000
H	–3.917134000000	–0.496376000000	5.201535000000
H	–4.559921000000	–0.529273000000	3.528418000000
H	–4.184438000000	3.206144000000	2.914752000000
H	–5.593374000000	2.378864000000	3.587099000000
H	–4.746190000000	1.667583000000	2.194673000000
H	–2.237860000000	–0.932978000000	3.968867000000
H	–2.093445000000	3.195729000000	2.828203000000
H	1.446998000000	3.089910000000	1.670831000000
H	0.136782000000	4.175290000000	2.170279000000
H	1.973105000000	0.574733000000	4.488053000000
H	2.637260000000	2.046045000000	3.756009000000
C	3.842166000000	0.284426000000	3.501079000000
O	4.130798000000	–0.400377000000	2.529859000000
C	4.792608000000	0.520606000000	4.634345000000
H	5.469697000000	–0.336867000000	4.745564000000
H	4.278183000000	0.736588000000	5.581679000000
H	5.408389000000	1.401672000000	4.379325000000
C	–3.249482000000	–0.255000000000	0.383472000000
N	–4.297744000000	–0.506857000000	0.806778000000
C	–1.938872000000	0.057208000000	–0.156979000000
H	–1.301560000000	0.467099000000	0.635463000000
H	–2.026928000000	0.798780000000	–0.960676000000
H	–1.480086000000	–0.853345000000	–0.562049000000

Li**L**$_c$MeCN$^{•+}$, zero imaginary frequencies
143
–2917.62764691

Li	2.088916000000	–0.006374000000	–0.003805000000
C	–3.309672000000	–3.520947000000	–2.943876000000
C	–3.196818000000	–4.105772000000	–4.367464000000
C	–4.302742000000	–2.339353000000	–2.950661000000
C	–3.865318000000	–4.608555000000	–2.011980000000
C	–1.926434000000	–3.025402000000	–2.494651000000
C	–1.284114000000	–2.003608000000	–3.211981000000
C	–0.021240000000	–1.521631000000	–2.859527000000
C	0.623242000000	–2.102985000000	–1.755428000000
C	0.009163000000	–3.095689000000	–0.983863000000
C	–1.258290000000	–3.547525000000	–1.381978000000
C	0.615309000000	–3.588229000000	0.322134000000
O	1.878467000000	–1.626702000000	–1.391930000000
C	2.944752000000	–2.355560000000	–1.963735000000
H	–4.176345000000	–4.474719000000	–4.710364000000
H	–2.486787000000	–4.946763000000	–4.390007000000
H	–2.850334000000	–3.352959000000	–5.091345000000
H	–5.292971000000	–2.672685000000	–3.298248000000
H	–3.973939000000	–1.527402000000	–3.616375000000
H	–4.424444000000	–1.921726000000	–1.939216000000
H	–3.219618000000	–5.499442000000	–1.991301000000
H	–4.858167000000	–4.927852000000	–2.362281000000
H	–3.981322000000	–4.242571000000	–0.979735000000
H	–1.781095000000	–1.561867000000	–4.077136000000
H	–1.726369000000	–4.331543000000	–0.789219000000
H	1.701083000000	–3.448867000000	0.323835000000
H	0.404584000000	–4.662924000000	0.427161000000
H	2.804423000000	–2.456772000000	–3.058200000000
H	3.010633000000	–3.372827000000	–1.545405000000
C	4.251051000000	–1.628897000000	–1.734872000000
O	4.264044000000	–0.490826000000	–1.310247000000
C	5.498744000000	–2.396305000000	–2.071674000000
H	6.346356000000	–1.712275000000	–2.207259000000
H	5.366939000000	–3.030509000000	–2.961773000000
H	5.703040000000	–3.059852000000	–1.215477000000
C	–3.341247000000	–2.845364000000	3.408892000000
C	–3.274723000000	–4.276065000000	3.983538000000
C	–4.311284000000	–2.818710000000	2.208770000000
C	–3.896513000000	–1.909088000000	4.493007000000
C	–1.936860000000	–2.426591000000	2.946684000000
C	–1.281451000000	–3.160992000000	1.946495000000
C	0.006274000000	–2.846970000000	1.502933000000
C	0.665092000000	–1.763050000000	2.105666000000
C	0.032099000000	–0.966941000000	3.069071000000
C	–1.258312000000	–1.327004000000	3.482557000000
C	0.642585000000	0.331040000000	3.578416000000
O	1.949228000000	–1.446698000000	1.676669000000
C	2.991326000000	–1.838235000000	2.547292000000
H	–4.268427000000	–4.595865000000	4.334897000000
H	–2.578458000000	–4.323856000000	4.834908000000
H	–2.935854000000	–5.003126000000	3.230396000000
H	–5.309437000000	–3.169448000000	2.513995000000
H	–3.969580000000	–3.465149000000	1.386290000000
H	–4.421980000000	–1.795770000000	1.816811000000
H	–3.268183000000	–1.911089000000	5.396510000000
H	–4.903715000000	–2.237867000000	4.789567000000
H	–3.980259000000	–0.871563000000	4.133150000000
H	–1.786820000000	–4.013924000000	1.491177000000
H	–1.737221000000	–0.714484000000	4.244477000000
H	1.729886000000	0.312587000000	3.460492000000
H	0.426477000000	0.428124000000	4.652696000000
H	2.629865000000	–2.601760000000	3.261219000000
H	3.355396000000	–0.983816000000	3.138708000000
C	4.160211000000	–2.458721000000	1.799616000000
O	4.025036000000	–2.949427000000	0.699369000000
C	5.478723000000	–2.432727000000	2.524318000000
H	6.146632000000	–3.210289000000	2.132303000000
H	5.357545000000	–2.542186000000	3.613175000000
H	5.924154000000	–1.441746000000	2.334686000000
C	–3.205843000000	3.562337000000	2.914836000000
C	–3.182282000000	3.973180000000	4.401402000000
C	–4.218043000000	2.413922000000	2.712280000000
C	–3.673559000000	4.768880000000	2.086661000000
C	–1.812836000000	3.072844000000	2.489995000000
C	–1.190167000000	2.035380000000	3.201459000000
C	0.066131000000	1.536316000000	2.850576000000
C	0.719598000000	2.108323000000	1.747111000000
C	0.124739000000	3.117564000000	0.981871000000
C	–1.131674000000	3.593621000000	1.385114000000
C	0.731026000000	3.597020000000	–0.329353000000
O	1.964719000000	1.607026000000	1.378715000000
C	3.048072000000	2.322139000000	1.935633000000
H	–4.172931000000	4.340040000000	4.712492000000
H	–2.450098000000	4.776910000000	4.574182000000
H	–2.920067000000	3.130842000000	5.058520000000
H	–5.222288000000	2.718296000000	3.046652000000
H	–3.933526000000	1.512660000000	3.276596000000
H	–4.288622000000	2.142016000000	1.646716000000
H	–2.986012000000	5.622522000000	2.186578000000
H	–4.665228000000	5.096647000000	2.432642000000
H	–3.764658000000	4.522085000000	1.017775000000
H	–1.699040000000	1.592756000000	4.058914000000
H	–1.584947000000	4.390616000000	0.798566000000
H	1.811506000000	3.422917000000	–0.346851000000
H	0.552593000000	4.678049000000	–0.429620000000
H	2.911849000000	2.444703000000	3.028441000000
H	3.134276000000	3.330541000000	1.500101000000
C	4.338911000000	1.564023000000	1.718941000000
O	4.328098000000	0.413233000000	1.330722000000
C	5.602733000000	2.319200000000	2.022409000000
H	6.439195000000	1.624628000000	2.172718000000
H	5.489023000000	2.982102000000	2.893970000000
H	5.811328000000	2.953431000000	1.145298000000

C	–3.333406000000	2.938603000000	–3.283433000000
C	–3.243276000000	4.318888000000	–3.967829000000
C	–4.228138000000	3.046199000000	–2.030162000000
C	–3.990761000000	1.949621000000	–4.258300000000
C	–1.920939000000	2.493821000000	–2.874406000000
C	–1.211729000000	3.222976000000	–1.908911000000
C	0.078173000000	2.875642000000	–1.499108000000
C	0.688916000000	1.769696000000	–2.111555000000
C	0.008067000000	0.986763000000	–3.053623000000
C	–1.287376000000	1.374239000000	–3.425120000000
C	0.573740000000	–0.323849000000	–3.584455000000
O	1.969688000000	1.413145000000	–1.705498000000
C	3.012072000000	1.772547000000	–2.588074000000
H	–4.243891000000	4.664554000000	–4.272359000000
H	–2.609305000000	4.269577000000	–4.866486000000
H	–2.815105000000	5.077300000000	–3.295189000000
H	–5.242021000000	3.371097000000	–2.310816000000
H	–3.837166000000	3.773275000000	–1.303481000000
H	–4.314426000000	2.074520000000	–1.519427000000
H	–3.427765000000	1.866214000000	–5.200145000000
H	–5.005114000000	2.292894000000	–4.510591000000
H	–4.081072000000	0.942742000000	–3.820848000000
H	–1.679657000000	4.092482000000	–1.446153000000
H	–1.806906000000	0.767001000000	–4.164525000000
H	1.663073000000	–0.334052000000	–3.485834000000
H	0.337166000000	–0.404359000000	–4.655796000000
H	2.667192000000	2.548993000000	–3.296332000000
H	3.340347000000	0.908372000000	–3.187175000000
C	4.210866000000	2.352118000000	–1.854017000000
O	4.106878000000	2.842330000000	–0.750549000000
C	5.518048000000	2.287526000000	–2.597412000000
H	6.212617000000	3.046973000000	–2.216299000000
H	5.385340000000	2.398296000000	–3.684755000000
H	5.940570000000	1.285988000000	–2.411001000000
C	–1.242219000000	–0.038034000000	–0.043108000000
N	–0.091711000000	0.026366000000	0.012113000000
C	–2.684519000000	–0.148720000000	–0.132360000000
H	–3.156794000000	0.553778000000	0.567995000000
H	–2.984178000000	–1.174401000000	0.120375000000
H	–3.002230000000	0.082245000000	–1.157926000000

Na**L**$_c$MeCN'$^+$, zero imaginary frequencies
143
–3072.35492475

Na	2.386397000000	–0.012681000000	–0.000092000000
C	–3.365738000000	–3.858720000000	–2.437172000000
C	–3.219260000000	–4.702460000000	–3.721470000000
C	–4.345760000000	–2.696080000000	–2.698627000000
C	–3.960959000000	–4.747092000000	–1.333974000000
C	–1.984519000000	–3.305464000000	–2.052083000000
C	–1.329171000000	–2.403866000000	–2.905934000000
C	–0.051814000000	–1.907342000000	–2.633121000000
C	0.598037000000	–2.366258000000	–1.474451000000
C	–0.042435000000	–3.201623000000	–0.550734000000
C	–1.325352000000	–3.670112000000	–0.872726000000
C	0.559447000000	–3.501721000000	0.817333000000
O	1.882509000000	–1.913448000000	–1.214540000000
C	2.917834000000	–2.740510000000	–1.690318000000
H	–4.193230000000	–5.120799000000	–4.021181000000
H	–2.521022000000	–5.538678000000	–3.562899000000
H	–2.838711000000	–4.100627000000	–4.560523000000
H	–5.329537000000	–3.086989000000	–3.001398000000
H	–3.994828000000	–2.030956000000	–3.501226000000
H	–4.490355000000	–2.088839000000	–1.791986000000
H	–3.335305000000	–5.630379000000	–1.135924000000
H	–4.953495000000	–5.108660000000	–1.641478000000
H	–4.087774000000	–4.195651000000	–0.389090000000
H	–1.826389000000	–2.074231000000	–3.819275000000
H	–1.811622000000	–4.343332000000	–0.169143000000
H	1.645047000000	–3.360271000000	0.796712000000
H	0.352936000000	–4.551955000000	1.073224000000
H	2.733828000000	–3.041523000000	–2.740979000000
H	3.008438000000	–3.665498000000	–1.095257000000
C	4.235289000000	–1.992623000000	–1.651636000000
O	4.272511000000	–0.795137000000	–1.439349000000
C	5.468239000000	–2.819542000000	–1.877782000000
H	6.300267000000	–2.187103000000	–2.213218000000
H	5.295915000000	–3.644519000000	–2.585368000000
H	5.726933000000	–3.261005000000	–0.900813000000
C	–3.413365000000	–2.301266000000	3.742557000000
C	–3.355587000000	–3.637505000000	4.512750000000
C	–4.383146000000	–2.438513000000	2.550335000000
C	–3.964729000000	–1.219373000000	4.683776000000
C	–2.004173000000	–1.960553000000	3.232067000000
C	–1.350638000000	–2.831215000000	2.346058000000
C	–0.050245000000	–2.602448000000	1.887180000000
C	0.623898000000	–1.465188000000	2.365622000000
C	–0.015119000000	–0.522839000000	3.183067000000
C	–1.318061000000	–0.804347000000	3.618134000000
C	0.606974000000	0.831443000000	3.504641000000
O	1.929979000000	–1.249877000000	1.949475000000
C	2.943467000000	–1.585380000000	2.868626000000
H	–4.351551000000	–3.899790000000	4.903295000000
H	–2.660782000000	–3.569030000000	5.363909000000
H	–3.019344000000	–4.464517000000	3.869534000000
H	–5.382571000000	–2.738998000000	2.901547000000
H	–4.044696000000	–3.195747000000	1.827154000000
H	–4.489085000000	–1.480673000000	2.018531000000
H	–3.337787000000	–1.098095000000	5.580052000000
H	–4.974353000000	–1.497662000000	5.021042000000
H	–4.041847000000	–0.242244000000	4.181793000000
H	–1.867573000000	–3.727221000000	1.999882000000
H	–1.800592000000	–0.085566000000	4.277603000000
H	1.692421000000	0.788566000000	3.372323000000
H	0.406262000000	1.074669000000	4.558973000000
H	2.585120000000	–2.344252000000	3.590193000000
H	3.262681000000	–0.705295000000	3.453299000000
C	4.152656000000	–2.179490000000	2.162217000000
O	4.067888000000	–2.651511000000	1.048517000000
C	5.441818000000	–2.136866000000	2.936545000000
H	6.142447000000	–2.891448000000	2.556906000000
H	5.282772000000	–2.266870000000	4.018142000000
H	5.873931000000	–1.134089000000	2.779042000000
C	–3.279503000000	3.884769000000	2.356604000000
C	–3.293740000000	4.466589000000	3.785047000000
C	–4.278456000000	2.710785000000	2.267910000000
C	–3.737647000000	4.982691000000	1.384307000000
C	–1.873011000000	3.364018000000	2.021756000000
C	–1.247968000000	2.442650000000	2.876551000000
C	0.027235000000	1.932162000000	2.623662000000
C	0.699809000000	2.383497000000	1.475280000000
C	0.089960000000	3.241022000000	0.551174000000
C	–1.184668000000	3.736813000000	0.862536000000
C	0.704335000000	3.522630000000	–0.816060000000
O	1.972607000000	1.894842000000	1.221132000000
C	3.034397000000	2.689791000000	1.689846000000
H	–4.293802000000	4.858469000000	4.028080000000
H	–2.570911000000	5.291550000000	3.879504000000
H	–3.042533000000	3.709441000000	4.542431000000
H	–5.291182000000	3.040195000000	2.548909000000
H	–3.995584000000	1.882223000000	2.935105000000
H	–4.326711000000	2.320113000000	1.238914000000
H	–3.054497000000	5.845643000000	1.394341000000
H	–4.736380000000	5.343167000000	1.672762000000
H	–3.810207000000	4.611479000000	0.350817000000
H	–1.770706000000	2.104861000000	3.772112000000
H	–1.647590000000	4.428033000000	0.161060000000
H	1.783458000000	3.338353000000	–0.798459000000
H	0.538258000000	4.580260000000	–1.071290000000
H	2.864147000000	3.002197000000	2.739581000000
H	3.155067000000	3.608390000000	1.089970000000
C	4.324823000000	1.895637000000	1.650262000000
O	4.318172000000	0.696056000000	1.446093000000
C	5.587717000000	2.679588000000	1.863671000000
H	6.398750000000	2.020561000000	2.199340000000
H	5.448638000000	3.515267000000	2.566002000000
H	5.856479000000	3.104638000000	0.882127000000

C	–3.381162000000	2.431687000000	–3.612484000000
C	–3.293859000000	3.670076000000	–4.529544000000
C	–4.257248000000	2.765617000000	–2.386548000000
C	–4.059140000000	1.291842000000	–4.388414000000
C	–1.962679000000	2.055871000000	–3.156713000000
C	–1.249635000000	2.918005000000	–2.311219000000
C	0.051580000000	2.646416000000	–1.880231000000
C	0.672511000000	1.481678000000	–2.363549000000
C	–0.018456000000	0.557125000000	–3.160058000000
C	–1.326676000000	0.873975000000	–3.552943000000
C	0.556089000000	–0.814005000000	–3.503343000000
O	1.974197000000	1.215088000000	–1.964215000000
C	2.993122000000	1.527258000000	–2.884819000000
H	–4.297111000000	3.968007000000	–4.873611000000
H	–2.676124000000	3.458089000000	–5.415784000000
H	–2.847299000000	4.528855000000	–4.005827000000
H	–5.276145000000	3.032621000000	–2.707010000000
H	–3.859949000000	3.614746000000	–1.811981000000
H	–4.332020000000	1.904996000000	–1.704128000000
H	–3.516835000000	1.044277000000	–5.313383000000
H	–5.078415000000	1.590302000000	–4.675529000000
H	–4.139948000000	0.376299000000	–3.781604000000
H	–1.724942000000	3.834879000000	–1.962534000000
H	–1.854747000000	0.162702000000	–4.185256000000
H	1.643350000000	–0.805734000000	–3.379222000000
H	0.339956000000	–1.036418000000	–4.559217000000
H	2.657133000000	2.306523000000	–3.595373000000
H	3.278743000000	0.643860000000	–3.482221000000
C	4.225844000000	2.071487000000	–2.178180000000
O	4.162665000000	2.534601000000	–1.059489000000
C	5.509275000000	1.992261000000	–2.959362000000
H	6.237347000000	2.718075000000	–2.575298000000
H	5.349970000000	2.139289000000	–4.038744000000
H	5.907978000000	0.973822000000	–2.814669000000
C	–1.353415000000	–0.053249000000	–0.048234000000
N	–0.205114000000	0.045201000000	0.015930000000
C	–2.791505000000	–0.214426000000	–0.146893000000
H	–3.297103000000	0.531317000000	0.481452000000
H	–3.066036000000	–1.222184000000	0.190809000000
H	–3.100947000000	–0.085541000000	–1.192427000000

K**L**$_e$MeCN$^{•+}$, zero imaginary frequencies
143
–3509.95085004

K	2.860605000000	–0.021971000000	0.001038000000
C	–3.479817000000	–4.304645000000	–1.092554000000
C	–3.391219000000	–5.467455000000	–2.103778000000
C	–4.445738000000	–3.229959000000	–1.632867000000
C	–4.056062000000	–4.847705000000	0.223987000000
C	–2.076727000000	–3.704940000000	–0.906885000000
C	–1.412606000000	–3.125933000000	–1.999835000000
C	–0.108451000000	–2.629719000000	–1.911038000000
C	0.560356000000	–2.768782000000	–0.680107000000
C	–0.101909000000	–3.219627000000	0.471706000000
C	–1.408936000000	–3.708151000000	0.322566000000
C	0.502154000000	–3.080496000000	1.868606000000
O	1.887241000000	–2.378863000000	–0.608262000000
C	2.843024000000	–3.409087000000	–0.680267000000
H	–4.381646000000	–5.926761000000	–2.250295000000
H	–2.701526000000	–6.246783000000	–1.744646000000
H	–3.030969000000	–5.125656000000	–3.085740000000
H	–5.442215000000	–3.664403000000	–1.808135000000
H	–4.098507000000	–2.805156000000	–2.586103000000
H	–4.561020000000	–2.404280000000	–0.914427000000
H	–3.433570000000	–5.652904000000	0.642576000000
H	–5.059785000000	–5.262680000000	0.048535000000
H	–4.154562000000	–4.057283000000	0.984224000000
H	–1.923650000000	–3.063838000000	–2.961156000000
H	–1.908130000000	–4.104353000000	1.204497000000
H	1.586303000000	–2.943577000000	1.800897000000
H	0.309144000000	–4.004821000000	2.434794000000
H	2.521664000000	–4.206019000000	–1.379882000000
H	3.002265000000	–3.886287000000	0.303989000000
C	4.173772000000	–2.872704000000	–1.188705000000
O	4.257967000000	–1.786038000000	–1.728326000000
C	5.365731000000	–3.754445000000	–0.943343000000
H	6.178241000000	–3.503180000000	–1.637006000000
H	5.116775000000	–4.824755000000	–1.004196000000
H	5.699414000000	–3.545329000000	0.088122000000
C	–3.485770000000	–1.009015000000	4.244462000000
C	–3.464099000000	–2.077536000000	5.357767000000
C	–4.453092000000	–1.452345000000	3.127058000000
C	–4.015489000000	0.303608000000	4.841850000000
C	–2.067004000000	–0.854328000000	3.673126000000
C	–1.418511000000	–1.960389000000	3.101383000000
C	–0.106887000000	–1.898061000000	2.621817000000
C	0.583575000000	–0.679291000000	2.762993000000
C	–0.059740000000	0.484342000000	3.210473000000
C	–1.373212000000	0.360176000000	3.687634000000
C	0.569852000000	1.870232000000	3.075081000000
O	1.910859000000	–0.630062000000	2.370095000000
C	2.872316000000	–0.717041000000	3.392803000000
H	–4.467632000000	–2.196579000000	5.795911000000
H	–2.770506000000	–1.790051000000	6.162918000000
H	–3.147608000000	–3.059555000000	4.975534000000
H	–5.461217000000	–1.621965000000	3.536397000000
H	–4.128145000000	–2.388158000000	2.647825000000
H	–4.533037000000	–0.679335000000	2.347673000000
H	–3.381880000000	0.662397000000	5.667066000000
H	–5.027996000000	0.148553000000	5.243524000000
H	–4.080482000000	1.100581000000	4.084995000000
H	–1.949017000000	–2.910446000000	3.030867000000
H	–1.858824000000	1.250850000000	4.080448000000
H	1.651651000000	1.782842000000	2.930694000000
H	0.393950000000	2.434817000000	4.003872000000
H	2.549027000000	–1.415146000000	4.190402000000
H	3.048021000000	0.263535000000	3.872088000000
C	4.191636000000	–1.240071000000	2.842211000000
O	4.258313000000	–1.775843000000	1.752279000000
C	5.395256000000	–1.013776000000	3.712971000000
H	6.195320000000	–1.718609000000	3.452946000000
H	5.155639000000	–1.072645000000	4.785501000000
H	5.741978000000	0.013003000000	3.501914000000
C	–3.396647000000	4.308990000000	1.039320000000
C	–3.421717000000	5.304278000000	2.217828000000
C	–4.378560000000	3.152242000000	1.322766000000
C	–3.870963000000	5.046183000000	–0.222634000000
C	–1.978242000000	3.738584000000	0.878113000000
C	–1.334825000000	3.144286000000	1.974773000000
C	–0.032799000000	2.641759000000	1.901266000000
C	0.650977000000	2.776579000000	0.678458000000
C	0.008796000000	3.243522000000	–0.478230000000
C	–1.292034000000	3.750410000000	–0.340698000000
C	0.620743000000	3.090782000000	–1.870946000000
O	1.970890000000	2.360402000000	0.617552000000
C	2.949878000000	3.366280000000	0.699861000000
H	–4.427341000000	5.739038000000	2.329735000000
H	–2.709681000000	6.127218000000	2.051204000000
H	–3.162143000000	4.821603000000	3.171591000000
H	–5.393054000000	3.540230000000	1.505292000000
H	–4.077502000000	2.567341000000	2.205154000000
H	–4.433151000000	2.468203000000	0.461170000000
H	–3.202120000000	5.881198000000	–0.481259000000
H	–4.876258000000	5.461396000000	–0.056435000000
H	–3.935476000000	4.373800000000	–1.091434000000
H	–1.862683000000	3.072978000000	2.925793000000
H	–1.776005000000	4.160939000000	–1.224250000000
H	1.699272000000	2.917621000000	–1.795639000000
H	0.462504000000	4.021979000000	–2.436831000000
H	2.647491000000	4.165272000000	1.405747000000
H	3.123188000000	3.848433000000	–0.279829000000
C	4.265192000000	2.790714000000	1.205663000000
O	4.319329000000	1.697010000000	1.735122000000
C	5.480946000000	3.642356000000	0.970872000000
H	6.285084000000	3.363049000000	1.663575000000
H	5.260505000000	4.718320000000	1.041235000000
H	5.811573000000	3.434340000000	–0.061842000000

C	–3.460462000000	1.157376000000	–4.186098000000
C	–3.401530000000	2.139399000000	–5.375315000000
C	–4.362204000000	1.746785000000	–3.080879000000
C	–4.091134000000	–0.157032000000	–4.669957000000
C	–2.037733000000	0.951577000000	–3.642570000000
C	–1.337819000000	2.038472000000	–3.097833000000
C	–0.026750000000	1.930364000000	–2.626807000000
C	0.616017000000	0.685780000000	–2.765160000000
C	–0.073903000000	–0.456557000000	–3.199816000000
C	–1.390702000000	–0.289143000000	–3.655032000000
C	0.514181000000	–1.862887000000	–3.077006000000
O	1.941899000000	0.587384000000	–2.377323000000
C	2.905051000000	0.666428000000	–3.398693000000
H	–4.408814000000	2.303965000000	–5.789791000000
H	–2.760802000000	1.743493000000	–6.178267000000
H	–2.997833000000	3.117795000000	–5.074198000000
H	–5.379567000000	1.916582000000	–3.466335000000
H	–3.983593000000	2.709978000000	–2.709114000000
H	–4.436712000000	1.062108000000	–2.222083000000
H	–3.515962000000	–0.609241000000	–5.492064000000
H	–5.108485000000	0.032410000000	–5.043549000000
H	–4.170158000000	–0.895722000000	–3.857510000000
H	–1.831003000000	3.008186000000	–3.032753000000
H	–1.916570000000	–1.165401000000	–4.028418000000
H	1.598218000000	–1.806077000000	–2.933593000000
H	0.321361000000	–2.412938000000	–4.011109000000
H	2.597112000000	1.381109000000	–4.187627000000
H	3.058924000000	–0.312033000000	–3.889843000000
C	4.235425000000	1.154729000000	–2.842197000000
O	4.312444000000	1.682463000000	–1.749119000000
C	5.435112000000	0.906332000000	–3.712544000000
H	6.252058000000	1.588227000000	–3.444038000000
H	5.199323000000	0.981927000000	–4.784913000000
H	5.755743000000	–0.130929000000	–3.511739000000
C	–1.278429000000	–0.053248000000	–0.016486000000
N	–0.127859000000	0.041392000000	0.004258000000
C	–2.720071000000	–0.206518000000	–0.046634000000
H	–3.201813000000	0.716530000000	0.301591000000
H	–3.009229000000	–1.037973000000	0.608383000000
H	–3.041074000000	–0.424964000000	–1.073295000000

Rb**L**$_c$MeCN$^{\prime+}$, zero imaginary frequencies
143
–2934.18195619

Rb	3.076209000000	–0.004549000000	–0.006331000000
C	–3.586346000000	–4.271013000000	–0.995476000000
C	–3.546910000000	–5.400840000000	–2.046022000000
C	–4.548553000000	–3.162628000000	–1.471277000000
C	–4.133496000000	–4.850040000000	0.318122000000
C	–2.171129000000	–3.694441000000	–0.829427000000
C	–1.508679000000	–3.137623000000	–1.934578000000
C	–0.197229000000	–2.657573000000	–1.863665000000
C	0.479458000000	–2.789745000000	–0.635905000000
C	–0.182452000000	–3.208848000000	0.528362000000
C	–1.495791000000	–3.685159000000	0.395546000000
C	0.433670000000	–3.043983000000	1.917429000000
O	1.822613000000	–2.452694000000	–0.583145000000
C	2.715277000000	–3.536719000000	–0.684349000000
H	–4.548034000000	–5.842031000000	–2.173741000000
H	–2.857071000000	–6.200575000000	–1.735184000000
H	–3.215713000000	–5.033542000000	–3.028868000000
H	–5.554984000000	–3.575311000000	–1.642793000000
H	–4.212778000000	–2.703851000000	–2.413009000000
H	–4.636073000000	–2.365158000000	–0.717914000000
H	–3.502370000000	–5.667226000000	0.699095000000
H	–5.142203000000	–5.257683000000	0.154349000000
H	–4.213547000000	–4.081762000000	1.102526000000
H	–2.027209000000	–3.082387000000	–2.891994000000
H	–1.993943000000	–4.062577000000	1.285949000000
H	1.514476000000	–2.890679000000	1.832580000000
H	0.260540000000	–3.962043000000	2.500442000000
H	2.272642000000	–4.364520000000	–1.271051000000
H	2.966121000000	–3.948493000000	0.312204000000
C	4.008679000000	–3.133790000000	–1.375837000000
O	4.145475000000	–2.045448000000	–1.900157000000
C	5.109740000000	–4.161516000000	–1.332688000000
H	5.811244000000	–4.001119000000	–2.161714000000
H	4.722092000000	–5.191291000000	–1.348826000000
H	5.656334000000	–4.028766000000	–0.382694000000
C	–3.582636000000	–0.954417000000	4.232031000000
C	–3.572017000000	–2.017333000000	5.350742000000
C	–4.539650000000	–1.402773000000	3.107654000000
C	–4.116937000000	0.361187000000	4.818633000000
C	–2.159183000000	–0.802904000000	3.671609000000
C	–1.500943000000	–1.915132000000	3.123877000000
C	–0.185120000000	–1.855744000000	2.654709000000
C	0.500041000000	–0.632697000000	2.787260000000
C	–0.155032000000	0.537165000000	3.200780000000
C	–1.472956000000	0.415789000000	3.666561000000
C	0.472919000000	1.921030000000	3.037812000000
O	1.843886000000	–0.589970000000	2.450798000000
C	2.737479000000	–0.712453000000	3.531087000000
H	–4.579527000000	–2.133077000000	5.780538000000
H	–2.885386000000	–1.726383000000	6.160641000000
H	–3.253513000000	–3.001812000000	4.976674000000
H	–5.552115000000	–1.568886000000	3.507668000000
H	–4.210564000000	–2.341570000000	2.637276000000
H	–4.610586000000	–0.634736000000	2.322430000000
H	–3.487064000000	0.726350000000	5.643954000000
H	–5.130975000000	0.207282000000	5.216878000000
H	–4.180282000000	1.153081000000	4.056510000000
H	–2.027145000000	–2.868107000000	3.065600000000
H	–1.967215000000	1.310535000000	4.038061000000
H	1.552381000000	1.826011000000	2.880573000000
H	0.309114000000	2.502543000000	3.958621000000
H	2.293837000000	–1.309700000000	4.350919000000
H	2.994332000000	0.276257000000	3.958073000000
C	4.025754000000	–1.404915000000	3.113467000000
O	4.154743000000	–1.915418000000	2.017525000000
C	5.131559000000	–1.381840000000	4.136639000000
H	5.827137000000	–2.213121000000	3.962629000000
H	4.748235000000	–1.408637000000	5.167811000000
H	5.683748000000	–0.433767000000	4.013648000000
C	–3.535550000000	4.266652000000	0.968483000000
C	–3.570380000000	5.280956000000	2.130394000000
C	–4.504053000000	3.104051000000	1.273218000000
C	–4.020409000000	4.977697000000	–0.304485000000
C	–2.110162000000	3.711463000000	0.814201000000
C	–1.455432000000	3.147116000000	1.919889000000
C	–0.143474000000	2.668688000000	1.854778000000
C	0.538677000000	2.802552000000	0.630523000000
C	–0.116120000000	3.226786000000	–0.535697000000
C	–1.427549000000	3.708389000000	–0.406567000000
C	0.502303000000	3.053998000000	–1.923299000000
O	1.881214000000	2.459817000000	0.581718000000
C	2.779049000000	3.534560000000	0.718504000000
H	–4.580718000000	5.706116000000	2.236607000000
H	–2.867910000000	6.109057000000	1.949202000000
H	–3.304224000000	4.817056000000	3.091643000000
H	–5.521963000000	3.484226000000	1.453249000000
H	–4.193933000000	2.537216000000	2.164159000000
H	–4.554790000000	2.404975000000	0.423648000000
H	–3.360981000000	5.815227000000	–0.578674000000
H	–5.029801000000	5.384742000000	–0.142986000000
H	–4.079734000000	4.290432000000	–1.161898000000
H	–1.981575000000	3.083722000000	2.872142000000
H	–1.921056000000	4.091961000000	–1.296605000000
H	1.579629000000	2.878052000000	–1.835211000000
H	0.350113000000	3.976244000000	–2.505699000000
H	2.336806000000	4.350263000000	1.322361000000
H	3.040502000000	3.971527000000	–0.264949000000
C	4.063310000000	3.104810000000	1.411129000000
O	4.189080000000	2.000334000000	1.903699000000
C	5.169795000000	4.127836000000	1.412744000000
H	5.854836000000	3.943870000000	2.250665000000
H	4.786957000000	5.158927000000	1.446581000000
H	5.734907000000	4.015398000000	0.471004000000

C	–3.581316000000	1.057504000000	–4.186474000000
C	–3.553527000000	2.079667000000	–5.342357000000
C	–4.493932000000	1.585026000000	–3.059021000000
C	–4.178164000000	–0.255592000000	–4.714802000000
C	–2.153154000000	0.870454000000	–3.650199000000
C	–1.459814000000	1.969192000000	–3.120941000000
C	–0.142817000000	1.880058000000	–2.661424000000
C	0.510759000000	0.640248000000	–2.795283000000
C	–0.176432000000	–0.515598000000	–3.197793000000
C	–1.497865000000	–0.365723000000	–3.645937000000
C	0.425748000000	–1.912854000000	–3.044578000000
O	1.855538000000	0.565029000000	–2.469339000000
C	2.744031000000	0.663431000000	–3.556308000000
H	–4.565883000000	2.225859000000	–5.751111000000
H	–2.901764000000	1.730408000000	–6.158004000000
H	–3.181543000000	3.060627000000	–5.010715000000
H	–5.513662000000	1.755847000000	–3.438001000000
H	–4.128233000000	2.536260000000	–2.645441000000
H	–4.559167000000	0.862505000000	–2.230946000000
H	–3.584938000000	–0.669620000000	–5.544247000000
H	–5.196601000000	–0.077957000000	–5.091277000000
H	–4.248172000000	–1.020247000000	–3.926080000000
H	–1.961913000000	2.934699000000	–3.064391000000
H	–2.019431000000	–1.250727000000	–4.003870000000
H	1.507468000000	–1.838040000000	–2.892107000000
H	0.246766000000	–2.486088000000	–3.967694000000
H	2.309357000000	1.270579000000	–4.373671000000
H	2.972755000000	–0.332030000000	–3.983455000000
C	4.052092000000	1.324292000000	–3.149269000000
O	4.200392000000	1.836544000000	–2.056778000000
C	5.150788000000	1.268351000000	–4.178915000000
H	5.867643000000	2.083181000000	–4.013665000000
H	4.762070000000	1.298941000000	–5.207970000000
H	5.680105000000	0.307639000000	–4.053583000000
C	–1.263273000000	–0.046756000000	–0.005899000000
N	–0.111421000000	0.035385000000	0.001207000000
C	–2.706942000000	–0.179782000000	–0.016057000000
H	–3.169819000000	0.753648000000	0.329119000000
H	–2.998873000000	–1.000152000000	0.651188000000
H	–3.044701000000	–0.403618000000	–1.035886000000

Cs**L**$_c$MeCN'$^+$, zero imaginary frequencies
143
–2930.22037408

Cs	3.323129000000	–0.007241000000	–0.003729000000
C	–3.714631000000	–3.967251000000	–1.730016000000
C	–3.722406000000	–4.844450000000	–2.999348000000
C	–4.658133000000	–2.762898000000	–1.933115000000
C	–4.250274000000	–4.806015000000	–0.559654000000
C	–2.285896000000	–3.461645000000	–1.473795000000
C	–1.619283000000	–2.719904000000	–2.460997000000
C	–0.301906000000	–2.275382000000	–2.310629000000
C	0.376703000000	–2.637540000000	–1.131010000000
C	–0.288621000000	–3.252891000000	–0.058985000000
C	–1.607084000000	–3.684280000000	–0.271453000000
C	0.329419000000	–3.344515000000	1.336274000000
O	1.729025000000	–2.348292000000	–1.027127000000
C	2.586739000000	–3.428128000000	–1.317459000000
H	–4.736094000000	–5.228699000000	–3.194034000000
H	–3.045867000000	–5.705429000000	–2.884818000000
H	–3.403291000000	–4.281197000000	–3.888921000000
H	–5.677888000000	–3.107340000000	–2.166092000000
H	–4.326363000000	–2.118489000000	–2.760755000000
H	–4.710601000000	–2.145142000000	–1.023772000000
H	–3.623088000000	–5.690449000000	–0.370489000000
H	–5.266318000000	–5.160395000000	–0.788995000000
H	–4.310140000000	–4.220471000000	0.370491000000
H	–2.140473000000	–2.484226000000	–3.388811000000
H	–2.107289000000	–4.213003000000	0.536824000000
H	1.409886000000	–3.177270000000	1.282058000000
H	0.156825000000	–4.353605000000	1.742446000000
H	2.057844000000	–4.216511000000	–1.883926000000
H	2.961204000000	–3.898584000000	–0.386904000000
C	3.792501000000	–2.996893000000	–2.138851000000
O	3.937456000000	–1.852300000000	–2.521962000000
C	4.817053000000	–4.074117000000	–2.395207000000
H	5.405831000000	–3.828408000000	–3.288677000000
H	4.361147000000	–5.070266000000	–2.496558000000
H	5.500857000000	–4.111135000000	–1.529249000000
C	–3.706483000000	–1.705141000000	3.956702000000
C	–3.736422000000	–2.986636000000	4.815279000000
C	–4.648992000000	–1.875685000000	2.746382000000
C	–4.227993000000	–0.539148000000	4.810517000000
C	–2.273100000000	–1.461055000000	3.457886000000
C	–1.610393000000	–2.453529000000	2.719485000000
C	–0.291306000000	–2.310918000000	2.277204000000
C	0.392901000000	–1.134165000000	2.638176000000
C	–0.267373000000	–0.058178000000	3.251533000000
C	–1.587454000000	–0.262804000000	3.680739000000
C	0.356620000000	1.334409000000	3.340247000000
O	1.745712000000	–1.036835000000	2.347533000000
C	2.603201000000	–1.340240000000	3.423363000000
H	–4.753895000000	–3.170394000000	5.194825000000
H	–3.059989000000	–2.895462000000	5.679111000000
H	–3.430918000000	–3.873471000000	4.240318000000
H	–5.673165000000	–2.100315000000	3.083307000000
H	–4.326664000000	–2.697665000000	2.089525000000
H	–4.686685000000	–0.954415000000	2.145100000000
H	–3.597806000000	–0.368561000000	5.696600000000
H	–5.246498000000	–0.761024000000	5.162684000000
H	–4.277289000000	0.398811000000	4.236557000000
H	–2.136441000000	–3.378556000000	2.483777000000
H	–2.084586000000	0.548846000000	4.206845000000
H	1.436033000000	1.275612000000	3.167335000000
H	0.191437000000	1.740928000000	4.350506000000
H	2.075025000000	–1.917886000000	4.204273000000
H	2.976463000000	–0.415826000000	3.906953000000
C	3.809281000000	–2.155359000000	2.981545000000
O	3.950823000000	–2.529043000000	1.833404000000
C	4.837508000000	–2.419493000000	4.053275000000
H	5.424846000000	–3.311691000000	3.799619000000
H	4.385218000000	–2.526955000000	5.050441000000
H	5.522019000000	–1.554225000000	4.093280000000
C	–3.676156000000	3.975633000000	1.726047000000
C	–3.717907000000	4.771703000000	3.046875000000
C	–4.629993000000	2.766038000000	1.826179000000
C	–4.174525000000	4.890983000000	0.597049000000
C	–2.244895000000	3.473618000000	1.474643000000
C	–1.582669000000	2.725224000000	2.459749000000
C	–0.265922000000	2.278648000000	2.310724000000
C	0.414572000000	2.640968000000	1.132551000000
C	–0.247590000000	3.260034000000	0.060964000000
C	–1.563980000000	3.696769000000	0.273611000000
C	0.370685000000	3.346845000000	–1.334715000000
O	1.767018000000	2.349161000000	1.029903000000
C	2.625264000000	3.423468000000	1.336019000000
H	–4.733329000000	5.157051000000	3.229378000000
H	–3.028041000000	5.628904000000	3.009998000000
H	–3.439054000000	4.151397000000	3.911487000000
H	–5.652085000000	3.097525000000	2.068125000000
H	–4.312156000000	2.058862000000	2.607191000000
H	–4.673861000000	2.220899000000	0.870171000000
H	–3.527087000000	5.772299000000	0.472122000000
H	–5.188607000000	5.250114000000	0.827946000000
H	–4.227442000000	4.362606000000	–0.366822000000
H	–2.106895000000	2.486566000000	3.384738000000
H	–2.061878000000	4.229586000000	–0.533237000000
H	1.449015000000	3.165287000000	–1.281797000000
H	0.210947000000	4.358560000000	–1.739752000000
H	2.100154000000	4.198738000000	1.924079000000
H	2.991124000000	3.916010000000	0.413326000000
C	3.836941000000	2.976446000000	2.139800000000
O	3.980664000000	1.825969000000	2.505332000000
C	4.866810000000	4.046468000000	2.404418000000
H	5.459717000000	3.787012000000	3.291251000000
H	4.415108000000	5.042842000000	2.521272000000
H	5.545952000000	4.092246000000	1.535218000000

C	–3.701159000000	1.770912000000	–3.928740000000
C	–3.698430000000	3.019688000000	–4.835169000000
C	–4.621644000000	2.021286000000	–2.715114000000
C	–4.271084000000	0.591664000000	–4.731310000000
C	–2.269576000000	1.500510000000	–3.439080000000
C	–1.585303000000	2.485070000000	–2.710762000000
C	–0.267029000000	2.322556000000	–2.274479000000
C	0.396202000000	1.134405000000	–2.636352000000
C	–0.284508000000	0.066783000000	–3.242729000000
C	–1.605464000000	0.289665000000	–3.661788000000
C	0.323139000000	–1.332964000000	–3.339906000000
O	1.749237000000	1.016587000000	–2.354990000000
C	2.603949000000	1.308567000000	–3.436466000000
H	–4.714441000000	3.227050000000	–5.206586000000
H	–3.039560000000	2.871038000000	–5.704576000000
H	–3.350408000000	3.914523000000	–4.297884000000
H	–5.644064000000	2.257576000000	–3.049269000000
H	–4.268349000000	2.861417000000	–2.099272000000
H	–4.676286000000	1.131418000000	–2.069024000000
H	–3.664510000000	0.374012000000	–5.623524000000
H	–5.290214000000	0.829174000000	–5.071133000000
H	–4.333036000000	–0.324739000000	–4.124811000000
H	–2.095039000000	3.419106000000	–2.475762000000
H	–2.119422000000	–0.516458000000	–4.180514000000
H	1.404130000000	–1.285767000000	–3.173711000000
H	0.147023000000	–1.733919000000	–4.350491000000
H	2.076398000000	1.886957000000	–4.217119000000
H	2.967175000000	0.378749000000	–3.917185000000
C	3.819498000000	2.114892000000	–3.004483000000
O	3.972542000000	2.488547000000	–1.857877000000
C	4.841674000000	2.370116000000	–4.084246000000
H	5.438091000000	3.257661000000	–3.835482000000
H	4.382703000000	2.480891000000	–5.077986000000
H	5.518899000000	1.499381000000	–4.129039000000
C	–1.244030000000	–0.036430000000	–0.009649000000
N	–0.090895000000	0.017989000000	0.014208000000
C	–2.690300000000	–0.127514000000	–0.042702000000
H	–3.127999000000	0.740236000000	0.466932000000
H	–3.006677000000	–1.047186000000	0.464534000000
H	–3.029391000000	–0.151955000000	–1.085913000000

Mg**L**$_c$MeCN'$^{2+}$, zero imaginary frequencies
143
–3109.92680002

Mg	2.272746000000	–0.001860000000	0.000313000000
C	–3.333157000000	–3.035239000000	–3.420728000000
C	–3.265814000000	–3.320058000000	–4.935928000000
C	–4.312258000000	–1.870972000000	–3.154723000000
C	–3.866934000000	–4.288267000000	–2.710262000000
C	–1.938109000000	–2.636095000000	–2.918204000000
C	–1.291545000000	–1.517529000000	–3.471345000000
C	–0.028211000000	–1.096972000000	–3.049130000000
C	0.602780000000	–1.851866000000	–2.049845000000
C	–0.004344000000	–2.949009000000	–1.436984000000
C	–1.273016000000	–3.329501000000	–1.900437000000
C	0.603787000000	–3.627186000000	–0.217132000000
O	1.868880000000	–1.439490000000	–1.603088000000
C	2.950448000000	–1.973520000000	–2.348538000000
H	–4.257279000000	–3.616055000000	–5.311083000000
H	–2.561890000000	–4.138202000000	–5.151764000000
H	–2.945208000000	–2.436781000000	–5.508387000000
H	–5.316455000000	–2.123913000000	–3.527198000000
H	–3.997480000000	–0.943267000000	–3.656446000000
H	–4.397337000000	–1.665559000000	–2.075699000000
H	–3.218270000000	–5.161641000000	–2.876319000000
H	–4.863684000000	–4.539762000000	–3.100454000000
H	–3.970726000000	–4.133240000000	–1.624748000000
H	–1.789225000000	–0.948237000000	–4.257270000000
H	–1.746310000000	–4.194828000000	–1.440225000000
H	1.691149000000	–3.498051000000	–0.206881000000
H	0.390957000000	–4.704627000000	–0.265146000000
H	2.753594000000	–1.889755000000	–3.432763000000
H	3.116801000000	–3.037198000000	–2.115734000000
C	4.177647000000	–1.170951000000	–2.014549000000
O	4.055203000000	–0.191775000000	–1.287746000000
C	5.487776000000	–1.607275000000	–2.573363000000
H	6.208285000000	–0.779475000000	–2.573749000000
H	5.389782000000	–2.046895000000	–3.576722000000
H	5.861499000000	–2.397181000000	–1.898450000000
C	–3.378924000000	–3.338297000000	2.899427000000
C	–3.322456000000	–4.842727000000	3.239668000000
C	–4.328256000000	–3.114531000000	1.702445000000
C	–3.943789000000	–2.581775000000	4.111217000000
C	–1.969731000000	–2.856884000000	2.524077000000
C	–1.294090000000	–3.443842000000	1.442776000000
C	–0.006838000000	–3.058243000000	1.056681000000
C	0.625081000000	–2.057785000000	1.810317000000
C	–0.018444000000	–1.406431000000	2.868338000000
C	–1.309767000000	–1.832349000000	3.211441000000
C	0.574799000000	–0.195465000000	3.575442000000
O	1.933034000000	–1.671316000000	1.452422000000
C	2.946136000000	–2.319662000000	2.218186000000
H	–4.321361000000	–5.205381000000	3.526188000000
H	–2.637682000000	–5.030585000000	4.080640000000
H	–2.982806000000	–5.446166000000	2.384576000000
H	–5.332934000000	–3.498424000000	1.935387000000
H	–3.980379000000	–3.630220000000	0.794347000000
H	–4.428759000000	–2.041562000000	1.473692000000
H	–3.323609000000	–2.724142000000	5.009121000000
H	–4.951384000000	–2.954706000000	4.345191000000
H	–4.031771000000	–1.501080000000	3.916795000000
H	–1.784881000000	–4.233673000000	0.873131000000
H	–1.803915000000	–1.339082000000	4.046236000000
H	1.664968000000	–0.194068000000	3.472142000000
H	0.352218000000	–0.269987000000	4.649516000000
H	2.532713000000	–3.237806000000	2.670248000000
H	3.281176000000	–1.675854000000	3.048087000000
C	4.138462000000	–2.730611000000	1.373117000000
O	4.075186000000	–2.735792000000	0.161930000000
C	5.368792000000	–3.129379000000	2.136959000000
H	6.013112000000	–3.763983000000	1.515182000000
H	5.126933000000	–3.638184000000	3.082528000000
H	5.922677000000	–2.208672000000	2.389811000000
C	–3.306693000000	3.065486000000	3.395671000000
C	–3.298588000000	3.233928000000	4.929157000000
C	–4.298185000000	1.948524000000	3.001096000000
C	–3.782517000000	4.382362000000	2.763355000000
C	–1.905126000000	2.664999000000	2.912081000000
C	–1.272737000000	1.536747000000	3.461804000000
C	–0.011230000000	1.106434000000	3.043830000000
C	0.629061000000	1.856906000000	2.047441000000
C	0.035585000000	2.963103000000	1.437679000000
C	–1.228509000000	3.357176000000	1.901289000000
C	0.651151000000	3.635242000000	0.218302000000
O	1.892179000000	1.433177000000	1.602060000000
C	2.979412000000	1.956731000000	2.346213000000
H	–4.295724000000	3.537856000000	5.281870000000
H	–2.578707000000	4.007712000000	5.236507000000
H	–3.036681000000	2.300413000000	5.449022000000
H	–5.308863000000	2.184440000000	3.367604000000
H	–4.009302000000	0.973243000000	3.422570000000
H	–4.358597000000	1.847598000000	1.904903000000
H	–3.108061000000	5.218152000000	3.003812000000
H	–4.779536000000	4.640131000000	3.148844000000
H	–3.867000000000	4.307325000000	1.668037000000
H	–1.780023000000	0.967695000000	4.241564000000
H	–1.690999000000	4.229964000000	1.444429000000
H	1.736871000000	3.493207000000	0.205787000000
H	0.451003000000	4.715117000000	0.266349000000
H	2.782025000000	1.876942000000	3.430633000000
H	3.157473000000	3.018124000000	2.111541000000
C	4.197216000000	1.139583000000	2.012436000000
O	4.062542000000	0.160749000000	1.287295000000
C	5.512920000000	1.562009000000	2.568816000000
H	6.224104000000	0.726185000000	2.569800000000
H	5.421134000000	2.004436000000	3.571532000000
H	5.894725000000	2.346506000000	1.892108000000

C	–3.365362000000	3.362408000000	–2.847972000000
C	–3.297312000000	4.850607000000	–3.251920000000
C	–4.281522000000	3.203846000000	–1.615068000000
C	–3.975461000000	2.564688000000	–4.010361000000
C	–1.952273000000	2.877079000000	–2.494248000000
C	–1.258723000000	3.465865000000	–1.426095000000
C	0.029008000000	3.071774000000	–1.052247000000
C	0.645468000000	2.064014000000	–1.808487000000
C	–0.012510000000	1.415685000000	–2.859889000000
C	–1.305925000000	1.847861000000	–3.188231000000
C	0.567817000000	0.202584000000	–3.575266000000
O	1.951082000000	1.663635000000	–1.457442000000
C	2.968922000000	2.302922000000	–2.224088000000
H	–4.300193000000	5.219867000000	–3.515477000000
H	–2.641836000000	4.991772000000	–4.124773000000
H	–2.914185000000	5.480420000000	–2.435004000000
H	–5.295282000000	3.564527000000	–1.845512000000
H	–3.915876000000	3.775868000000	–0.749275000000
H	–4.363815000000	2.147337000000	–1.313386000000
H	–3.385496000000	2.667767000000	–4.933631000000
H	–4.987722000000	2.936376000000	–4.225260000000
H	–4.063604000000	1.492715000000	–3.772267000000
H	–1.738341000000	4.259987000000	–0.853376000000
H	–1.813025000000	1.355439000000	–4.015815000000
H	1.658195000000	0.191522000000	–3.474970000000
H	0.342875000000	0.285007000000	–4.648296000000
H	2.563988000000	3.225966000000	–2.673836000000
H	3.295843000000	1.657110000000	–3.055798000000
C	4.167177000000	2.699682000000	–1.380501000000
O	4.107138000000	2.699899000000	–0.169148000000
C	5.399171000000	3.090405000000	–2.145838000000
H	6.051154000000	3.715858000000	–1.522778000000
H	5.159541000000	3.606098000000	–3.088237000000
H	5.943821000000	2.165878000000	–2.404812000000
C	–1.084553000000	–0.030606000000	–0.026539000000
N	0.067109000000	0.008138000000	0.001054000000
C	–2.527561000000	–0.100867000000	–0.072336000000
H	–2.950825000000	0.496802000000	0.747249000000
H	–2.838126000000	–1.148907000000	0.036834000000
H	–2.876999000000	0.289899000000	–1.037709000000

$Ca\mathbf{L_c}MeCN'^{2+}$, zero imaginary frequencies
143
–3587.47794509

Ca	2.639512000000	–0.001527000000	–0.000647000000
C	–3.437399000000	–3.654151000000	–2.475738000000
C	–3.410992000000	–4.318340000000	–3.868536000000
C	–4.383536000000	–2.434371000000	–2.502159000000
C	–3.985076000000	–4.669422000000	–1.461316000000
C	–2.020264000000	–3.183967000000	–2.115459000000
C	–1.352000000000	–2.274909000000	–2.951827000000
C	–0.056116000000	–1.821676000000	–2.689537000000
C	0.593887000000	–2.342262000000	–1.557334000000
C	–0.052254000000	–3.184046000000	–0.645520000000
C	–1.353510000000	–3.604993000000	–0.959849000000
C	0.554083000000	–3.542938000000	0.708228000000
O	1.914751000000	–1.947703000000	–1.304188000000
C	2.899329000000	–2.727301000000	–1.960604000000
H	–4.416879000000	–4.673766000000	–4.139310000000
H	–2.729885000000	–5.182945000000	–3.879336000000
H	–3.084284000000	–3.619369000000	–4.652981000000
H	–5.398318000000	–2.744387000000	–2.794233000000
H	–4.051377000000	–1.669879000000	–3.220693000000
H	–4.450325000000	–1.964109000000	–1.508552000000
H	–3.359604000000	–5.573367000000	–1.407506000000
H	–4.995238000000	–4.984754000000	–1.759986000000
H	–4.063330000000	–4.240045000000	–0.450286000000
H	–1.858872000000	–1.901788000000	–3.841954000000
H	–1.850410000000	–4.286350000000	–0.272523000000
H	1.641162000000	–3.412739000000	0.674753000000
H	0.351962000000	–4.603245000000	0.919569000000
H	2.519223000000	–3.099762000000	–2.928725000000
H	3.176490000000	–3.613251000000	–1.360190000000
C	4.132144000000	–1.889869000000	–2.218741000000
O	4.142774000000	–0.710834000000	–1.900238000000
C	5.309958000000	–2.573475000000	–2.838073000000
H	5.955131000000	–1.842581000000	–3.342541000000
H	5.017090000000	–3.378596000000	–3.527520000000
H	5.890698000000	–3.032141000000	–2.018047000000
C	–3.435050000000	–2.471481000000	3.652581000000
C	–3.421091000000	–3.874368000000	4.295200000000
C	–4.382974000000	–2.469797000000	2.433675000000
C	–3.972691000000	–1.467660000000	4.683753000000
C	–2.015872000000	–2.115232000000	3.184910000000
C	–1.347605000000	–2.953505000000	2.277407000000
C	–0.051932000000	–2.691458000000	1.822732000000
C	0.597672000000	–1.558007000000	2.340962000000
C	–0.048213000000	–0.645349000000	3.181778000000
C	–1.349160000000	–0.958784000000	3.603491000000
C	0.558729000000	0.708064000000	3.539346000000
O	1.918353000000	–1.305185000000	1.945360000000
C	2.904003000000	–1.960868000000	2.724097000000
H	–4.428441000000	–4.139533000000	4.650620000000
H	–2.736821000000	–3.905702000000	5.156790000000
H	–3.106191000000	–4.652152000000	3.583511000000
H	–5.398235000000	–2.766361000000	2.737956000000
H	–4.053302000000	–3.172349000000	1.653060000000
H	–4.449213000000	–1.464936000000	1.987318000000
H	–3.342707000000	–1.429609000000	5.585366000000
H	–4.983259000000	–1.764778000000	4.999348000000
H	–4.046986000000	–0.450613000000	4.268075000000
H	–1.854146000000	–3.844790000000	1.906539000000
H	–1.846325000000	–0.269846000000	4.282939000000
H	1.645821000000	0.673854000000	3.409161000000
H	0.357036000000	0.920129000000	4.599591000000
H	2.523965000000	–2.927883000000	3.099456000000
H	3.183819000000	–1.358828000000	3.608106000000
C	4.134587000000	–2.221395000000	1.884038000000
O	4.142255000000	–1.904962000000	0.704414000000
C	5.313853000000	–2.840102000000	2.565674000000
H	5.956759000000	–3.346395000000	1.834041000000
H	5.022774000000	–3.527756000000	3.372971000000
H	5.896286000000	–2.019464000000	3.021087000000
C	–3.423560000000	3.663953000000	2.472725000000
C	–3.427416000000	4.251122000000	3.899636000000
C	–4.380560000000	2.453608000000	2.408966000000
C	–3.936977000000	4.739264000000	1.502999000000
C	–2.004165000000	3.195613000000	2.117320000000
C	–1.340598000000	2.280311000000	2.950761000000
C	–0.046164000000	1.823023000000	2.687989000000
C	0.605016000000	2.342094000000	1.556051000000
C	–0.037471000000	3.188408000000	0.646101000000
C	–1.335770000000	3.616310000000	0.962524000000
C	0.570488000000	3.545896000000	–0.707122000000
O	1.925119000000	1.944321000000	1.302952000000
C	2.911844000000	2.721094000000	1.959079000000
H	–4.434893000000	4.609982000000	4.159347000000
H	–2.732764000000	5.101398000000	3.977218000000
H	–3.138017000000	3.506277000000	4.655847000000
H	–5.396067000000	2.750886000000	2.711968000000
H	–4.059183000000	1.637839000000	3.074470000000
H	–4.445374000000	2.056198000000	1.382992000000
H	–3.292470000000	5.631408000000	1.503616000000
H	–4.945404000000	5.058981000000	1.803031000000
H	–4.009153000000	4.362900000000	0.470816000000
H	–1.849721000000	1.906265000000	3.839081000000
H	–1.829633000000	4.301929000000	0.277301000000
H	1.656986000000	3.410568000000	–0.674305000000
H	0.373177000000	4.607193000000	–0.918041000000
H	2.533245000000	3.094363000000	2.927490000000
H	3.191297000000	3.606482000000	1.358857000000
C	4.142312000000	1.879904000000	2.216162000000
O	4.149118000000	0.700974000000	1.896974000000
C	5.322561000000	2.559496000000	2.835253000000
H	5.965961000000	1.826314000000	3.338661000000
H	5.032455000000	3.364856000000	3.525591000000
H	5.904088000000	3.017274000000	2.015286000000

C	–3.432317000000	2.495483000000	–3.633877000000
C	–3.395718000000	3.873673000000	–4.327612000000
C	–4.365229000000	2.560435000000	–2.405146000000
C	–4.002757000000	1.466532000000	–4.621525000000
C	–2.014331000000	2.128643000000	–3.171458000000
C	–1.337042000000	2.965452000000	–2.269883000000
C	–0.041362000000	2.697356000000	–1.820335000000
C	0.600381000000	1.559934000000	–2.339226000000
C	–0.053169000000	0.649172000000	–3.176501000000
C	–1.355389000000	0.968021000000	–3.591913000000
C	0.549135000000	–0.705535000000	–3.539134000000
O	1.921132000000	1.300712000000	–1.947959000000
C	2.907645000000	1.954708000000	–2.727010000000
H	–4.402431000000	4.151445000000	–4.675332000000
H	–2.726241000000	3.856662000000	–5.201147000000
H	–3.048009000000	4.667252000000	–3.649354000000
H	–5.381716000000	2.849088000000	–2.712734000000
H	–4.021597000000	3.296660000000	–1.663108000000
H	–4.433055000000	1.580809000000	–1.905572000000
H	–3.390225000000	1.389270000000	–5.532601000000
H	–5.014233000000	1.768372000000	–4.929545000000
H	–4.084032000000	0.464808000000	–4.171388000000
H	–1.837736000000	3.859377000000	–1.897888000000
H	–1.858807000000	0.281012000000	–4.268846000000
H	1.636624000000	–0.673999000000	–3.411731000000
H	0.344151000000	–0.914147000000	–4.599443000000
H	2.529315000000	2.922601000000	–3.101761000000
H	3.185648000000	1.352390000000	–3.611411000000
C	4.139384000000	2.212427000000	–1.887754000000
O	4.147450000000	1.895423000000	–0.708282000000
C	5.319202000000	2.829477000000	–2.569989000000
H	5.963766000000	3.333865000000	–1.838502000000
H	5.028587000000	3.518496000000	–3.376291000000
H	5.899682000000	2.008267000000	–3.026858000000
C	–1.034998000000	–0.033386000000	–0.014511000000
N	0.117902000000	0.001811000000	0.002081000000
C	–2.479762000000	–0.094551000000	–0.043019000000
H	–2.893513000000	0.696673000000	0.596546000000
H	–2.805922000000	–1.075375000000	0.327469000000
H	–2.826367000000	0.043710000000	–1.075868000000

Sr**L$_c$**MeCN$'^{2+}$, zero imaginary frequencies
143
–2940.6669847

Sr	2.667679000000	–0.002749000000	–0.001285000000
C	–3.551845000000	–3.716093000000	–2.326821000000
C	–3.526994000000	–4.425976000000	–3.697109000000
C	–4.499220000000	–2.499183000000	–2.393305000000
C	–4.098127000000	–4.697851000000	–1.279176000000
C	–2.132562000000	–3.237305000000	–1.985628000000
C	–1.462052000000	–2.362122000000	–2.855624000000
C	–0.155884000000	–1.921296000000	–2.623495000000
C	0.502279000000	–2.426937000000	–1.487713000000
C	–0.153368000000	–3.210385000000	–0.529757000000
C	–1.464205000000	–3.620838000000	–0.817863000000
C	0.454211000000	–3.515578000000	0.839085000000
O	1.843400000000	–2.076687000000	–1.281575000000
C	2.783614000000	–2.918633000000	–1.925971000000
H	–4.532747000000	–4.792015000000	–3.953920000000
H	–2.844151000000	–5.289126000000	–3.680986000000
H	–3.203264000000	–3.752500000000	–4.504748000000
H	–5.511529000000	–2.819115000000	–2.683193000000
H	–4.163708000000	–1.754502000000	–3.130734000000
H	–4.573842000000	–2.001394000000	–1.414021000000
H	–3.472262000000	–5.599336000000	–1.196602000000
H	–5.108673000000	–5.022856000000	–1.565950000000
H	–4.175245000000	–4.235672000000	–0.282711000000
H	–1.974338000000	–2.010439000000	–3.751109000000
H	–1.966878000000	–4.266555000000	–0.101225000000
H	1.540974000000	–3.382277000000	0.801130000000
H	0.256796000000	–4.568222000000	1.090447000000
H	2.373512000000	–3.304531000000	–2.876822000000
H	3.028288000000	–3.798697000000	–1.301980000000
C	4.055373000000	–2.155147000000	–2.233944000000
O	4.138756000000	–0.964937000000	–1.968941000000
C	5.183229000000	–2.931339000000	–2.839775000000
H	5.828769000000	–2.268758000000	–3.431174000000
H	4.835467000000	–3.779924000000	–3.446461000000
H	5.788167000000	–3.339239000000	–2.010373000000
C	–3.551984000000	–2.309354000000	3.713090000000
C	–3.542386000000	–3.686762000000	4.409100000000
C	–4.499335000000	–2.352687000000	2.494883000000
C	–4.088412000000	–1.265664000000	4.704509000000
C	–2.129412000000	–1.976837000000	3.237518000000
C	–1.461083000000	–2.851133000000	2.364806000000
C	–0.154067000000	–2.623626000000	1.923614000000
C	0.507178000000	–1.488522000000	2.426715000000
C	–0.145579000000	–0.527692000000	3.208754000000
C	–1.457412000000	–0.810583000000	3.618867000000
C	0.466003000000	0.839252000000	3.512532000000
O	1.848325000000	–1.285797000000	2.074186000000
C	2.789011000000	–1.936169000000	2.910303000000
H	–4.550604000000	–3.935429000000	4.773937000000
H	–2.858426000000	–3.687210000000	5.271510000000
H	–3.229145000000	–4.491781000000	3.727592000000
H	–5.513925000000	–2.640411000000	2.809601000000
H	–4.168620000000	–3.082082000000	1.739781000000
H	–4.567642000000	–1.364731000000	2.013174000000
H	–3.459802000000	–1.195088000000	5.605091000000
H	–5.100396000000	–1.547645000000	5.029332000000
H	–4.159102000000	–0.265028000000	4.250309000000
H	–1.975671000000	–3.746094000000	2.015067000000
H	–1.958352000000	–0.090950000000	4.262424000000
H	1.552548000000	0.798294000000	3.378156000000
H	0.270602000000	1.091410000000	4.565378000000
H	2.376792000000	–2.886096000000	3.296200000000
H	3.040570000000	–1.315442000000	3.790839000000
C	4.056053000000	–2.247549000000	2.140399000000
O	4.134852000000	–1.980956000000	0.950135000000
C	5.185298000000	–2.859099000000	2.910095000000
H	5.824878000000	–3.452657000000	2.243651000000
H	4.839315000000	–3.465015000000	3.759942000000
H	5.796055000000	–2.032746000000	3.315505000000
C	–3.532280000000	3.726728000000	2.310811000000
C	–3.553843000000	4.339092000000	3.726963000000
C	–4.489383000000	2.516134000000	2.257555000000
C	–4.033585000000	4.785512000000	1.316796000000
C	–2.107995000000	3.253396000000	1.981407000000
C	–1.445324000000	2.370863000000	2.849996000000
C	–0.140000000000	1.925658000000	2.621555000000
C	0.523038000000	2.429237000000	1.488003000000
C	–0.125616000000	3.218738000000	0.530475000000
C	–1.434002000000	3.637294000000	0.817077000000
C	0.486097000000	3.520920000000	–0.837118000000
O	1.862996000000	2.072295000000	1.284220000000
C	2.807045000000	2.905555000000	1.933419000000
H	–4.563602000000	4.705739000000	3.966086000000
H	–2.857422000000	5.188371000000	3.799452000000
H	–3.278101000000	3.606884000000	4.500376000000
H	–5.506436000000	2.817486000000	2.551239000000
H	–4.171541000000	1.709454000000	2.935777000000
H	–4.548803000000	2.105796000000	1.236582000000
H	–3.385478000000	5.674972000000	1.306316000000
H	–5.043454000000	5.114156000000	1.602015000000
H	–4.098786000000	4.390646000000	0.291230000000
H	–1.962831000000	2.017553000000	3.741628000000
H	–1.931233000000	4.288419000000	0.101703000000
H	1.571647000000	3.377926000000	–0.798894000000
H	0.298066000000	4.575447000000	–1.087871000000
H	2.399128000000	3.288272000000	2.886526000000
H	3.056545000000	3.788054000000	1.314702000000
C	4.074552000000	2.133154000000	2.236612000000
O	4.150687000000	0.943759000000	1.965378000000
C	5.207437000000	2.899407000000	2.845670000000
H	5.849964000000	2.229874000000	3.432496000000
H	4.864993000000	3.746381000000	3.457617000000
H	5.813710000000	3.308883000000	2.018016000000

C	–3.541656000000	2.349011000000	–3.686952000000
C	–3.511576000000	3.706118000000	–4.421722000000
C	–4.471803000000	2.448839000000	–2.458697000000
C	–4.111790000000	1.289769000000	–4.642108000000
C	–2.119737000000	2.001737000000	–3.220719000000
C	–1.438114000000	2.872711000000	–2.355752000000
C	–0.131555000000	2.634456000000	–1.920600000000
C	0.517271000000	1.492963000000	–2.424927000000
C	–0.147599000000	0.535716000000	–3.201635000000
C	–1.460309000000	0.828467000000	–3.603753000000
C	0.454453000000	–0.834806000000	–3.511335000000
O	1.858346000000	1.279317000000	–2.078565000000
C	2.801461000000	1.925904000000	–2.914850000000
H	–4.519910000000	3.969429000000	–4.775947000000
H	–2.843201000000	3.665780000000	–5.295309000000
H	–3.166168000000	4.520905000000	–3.767850000000
H	–5.487699000000	2.733849000000	–2.771590000000
H	–4.123575000000	3.201806000000	–1.736022000000
H	–4.543672000000	1.482706000000	–1.934297000000
H	–3.501171000000	1.187179000000	–5.551956000000
H	–5.125059000000	1.579275000000	–4.956024000000
H	–4.188979000000	0.301781000000	–4.162193000000
H	–1.943262000000	3.772535000000	–2.005459000000
H	–1.971230000000	0.111886000000	–4.243037000000
H	1.541522000000	–0.800654000000	–3.379248000000
H	0.255213000000	–1.081947000000	–4.564644000000
H	2.393803000000	2.878557000000	–3.298880000000
H	3.048627000000	1.304891000000	–3.796387000000
C	4.070950000000	2.229830000000	–2.145897000000
O	4.149867000000	1.960742000000	–0.956202000000
C	5.202403000000	2.836684000000	–2.916088000000
H	5.845679000000	3.426208000000	–2.249629000000
H	4.858441000000	3.445433000000	–3.764731000000
H	5.808529000000	2.007814000000	–3.323309000000
C	–1.178335000000	–0.037819000000	–0.011160000000
N	–0.025047000000	0.005321000000	0.003589000000
C	–2.622869000000	–0.114943000000	–0.035334000000
H	–3.044699000000	0.668236000000	0.608318000000
H	–2.936419000000	–1.100642000000	0.331857000000
H	–2.975772000000	0.022562000000	–1.065799000000

Ba**L**$_c$MeCN'$^{2+}$, zero imaginary frequencies
143
–2935.40769207

Ba	–2.803818000000	0.002627000000	0.000725000000
C	3.676862000000	3.761153000000	–2.160012000000
C	3.672252000000	4.501292000000	–3.514401000000
C	4.619868000000	2.541936000000	–2.242237000000
C	4.214567000000	4.717759000000	–1.085035000000
C	2.251158000000	3.282216000000	–1.846277000000
C	1.579391000000	2.444170000000	–2.750702000000
C	0.262839000000	2.018239000000	–2.550616000000
C	–0.404755000000	2.505391000000	–1.410835000000
C	0.257533000000	3.228735000000	–0.409644000000
C	1.578264000000	3.627063000000	–0.669645000000
C	–0.352337000000	3.482289000000	0.970562000000
O	–1.769552000000	2.222595000000	–1.264662000000
C	–2.618016000000	3.157453000000	–1.909836000000
H	4.682326000000	4.870012000000	–3.749421000000
H	2.991500000000	5.365862000000	–3.488304000000
H	3.358135000000	3.847044000000	–4.341389000000
H	5.635941000000	2.862896000000	–2.517556000000
H	4.286756000000	1.813767000000	–2.996980000000
H	4.684309000000	2.024339000000	–1.272681000000
H	3.588196000000	5.617348000000	–0.986896000000
H	5.227962000000	5.048270000000	–1.355012000000
H	4.282918000000	4.232595000000	–0.099066000000
H	2.097862000000	2.115712000000	–3.650977000000
H	2.084466000000	4.236616000000	0.075130000000
H	–1.438991000000	3.345766000000	0.930095000000
H	–0.159559000000	4.526051000000	1.260793000000
H	–2.159135000000	3.516784000000	–2.849252000000
H	–2.783833000000	4.050655000000	–1.277716000000
C	–3.955266000000	2.532063000000	–2.252276000000
O	–4.165195000000	1.349960000000	–2.026240000000
C	–4.989390000000	3.438868000000	–2.847575000000
H	–5.680865000000	2.863759000000	–3.477504000000
H	–4.546763000000	4.270952000000	–3.414016000000
H	–5.573505000000	3.874078000000	–2.017156000000
C	3.677559000000	2.143597000000	3.757241000000
C	3.687464000000	3.504343000000	4.485234000000
C	4.619932000000	2.204798000000	2.536152000000
C	4.206562000000	1.071653000000	4.722258000000
C	2.248699000000	1.838114000000	3.281849000000
C	1.578475000000	2.746822000000	2.446934000000
C	0.261171000000	2.550667000000	2.020794000000
C	–0.408887000000	1.411384000000	2.505781000000
C	0.251059000000	0.407553000000	3.227186000000
C	1.572806000000	0.662680000000	3.624347000000
C	–0.362421000000	–0.970941000000	3.479080000000
O	–1.773802000000	1.268427000000	2.221672000000
C	–2.622590000000	1.917021000000	3.153636000000
H	4.700030000000	3.731955000000	4.851723000000
H	3.006759000000	3.493060000000	5.350153000000
H	3.382152000000	4.329142000000	3.824095000000
H	5.638719000000	2.475796000000	2.852255000000
H	4.292418000000	2.954285000000	1.799618000000
H	4.676273000000	1.227700000000	2.031554000000
H	3.577996000000	0.984234000000	5.621415000000
H	5.221295000000	1.337133000000	5.052408000000
H	4.268918000000	0.081867000000	4.243986000000
H	2.098792000000	3.646840000000	2.120558000000
H	2.077844000000	–0.085078000000	4.230981000000
H	–1.448899000000	–0.927654000000	3.341754000000
H	–0.171532000000	–1.262216000000	4.522921000000
H	–2.162216000000	2.855700000000	3.513009000000
H	–2.792338000000	1.286490000000	4.047237000000
C	–3.957320000000	2.261989000000	2.524175000000
O	–4.164512000000	2.034696000000	1.341786000000
C	–4.992487000000	2.861198000000	3.427170000000
H	–5.681116000000	3.491565000000	2.849139000000
H	–4.550739000000	3.428177000000	4.259358000000
H	–5.579511000000	2.032863000000	3.862444000000
C	3.659925000000	–3.770722000000	2.144334000000
C	3.697412000000	–4.422100000000	3.542657000000
C	4.611352000000	–2.555042000000	2.116499000000
C	4.156768000000	–4.799882000000	1.117380000000
C	2.229905000000	–3.296664000000	1.840972000000
C	1.565001000000	–2.451952000000	2.744298000000
C	0.248863000000	–2.022725000000	2.548029000000
C	–0.423171000000	–2.508856000000	1.410775000000
C	0.233121000000	–3.237186000000	0.409487000000
C	1.551969000000	–3.642145000000	0.667457000000
C	–0.381002000000	–3.487426000000	–0.969316000000
O	–1.787353000000	–2.220722000000	1.267305000000
C	–2.638962000000	–3.150102000000	1.915672000000
H	4.710752000000	–4.791330000000	3.761776000000
H	3.004772000000	–5.275755000000	3.598242000000
H	3.426830000000	–3.712814000000	4.338914000000
H	5.631812000000	–2.859346000000	2.395037000000
H	4.294325000000	–1.768762000000	2.818571000000
H	4.661670000000	–2.116862000000	1.106802000000
H	3.510237000000	–5.690015000000	1.086110000000
H	5.169380000000	–5.134007000000	1.386058000000
H	4.214271000000	–4.376341000000	0.102955000000
H	2.088435000000	–2.121487000000	3.640711000000
H	2.053464000000	–4.256479000000	–0.076444000000
H	–1.466530000000	–3.342072000000	–0.927999000000
H	–0.196930000000	–4.532735000000	–1.259709000000
H	–2.181525000000	–3.507916000000	2.856408000000
H	–2.808292000000	–4.044970000000	1.286817000000
C	–3.973571000000	–2.517757000000	2.255600000000
O	–4.178046000000	–1.335459000000	2.025248000000
C	–5.012022000000	–3.417652000000	2.853780000000
H	–5.702051000000	–2.836948000000	3.480137000000
H	–4.573254000000	–4.248777000000	3.424626000000
H	–5.596719000000	–3.854633000000	2.024697000000

C	3.669003000000	–2.181152000000	–3.732820000000
C	3.662594000000	–3.525590000000	–4.490943000000
C	4.595082000000	–2.288840000000	–2.502205000000
C	4.227824000000	–1.098161000000	–4.667856000000
C	2.239992000000	–1.861902000000	–3.267658000000
C	1.557656000000	–2.766808000000	–2.439285000000
C	0.240710000000	–2.560649000000	–2.018675000000
C	–0.417868000000	–1.415279000000	–2.504356000000
C	0.253027000000	–0.415535000000	–3.221876000000
C	1.575400000000	–0.680252000000	–3.612469000000
C	–0.351980000000	0.966311000000	–3.479099000000
O	–1.782455000000	–1.261055000000	–2.224532000000
C	–2.634297000000	–1.903723000000	–3.157753000000
H	4.675778000000	–3.765851000000	–4.847620000000
H	2.995258000000	–3.481181000000	–5.365157000000
H	3.330160000000	–4.357736000000	–3.852455000000
H	5.614493000000	–2.562193000000	–2.814199000000
H	4.248815000000	–3.053333000000	–1.790756000000
H	4.657370000000	–1.329053000000	–1.965390000000
H	3.615485000000	–0.984928000000	–5.575309000000
H	5.244214000000	–1.370630000000	–4.986937000000
H	4.294921000000	–0.118683000000	–4.169662000000
H	2.069686000000	–3.670766000000	–2.111515000000
H	2.089525000000	0.064324000000	–4.215628000000
H	–1.438918000000	0.928956000000	–3.343880000000
H	–0.157345000000	1.253255000000	–4.523439000000
H	–2.179018000000	–2.844597000000	–3.517828000000
H	–2.799882000000	–1.271159000000	–4.050696000000
C	–3.971404000000	–2.241779000000	–2.529573000000
O	–4.177805000000	–2.015426000000	–1.346880000000
C	–5.009733000000	–2.833038000000	–3.434180000000
H	–5.702731000000	–3.459910000000	–2.857580000000
H	–4.570954000000	–3.401569000000	–4.266887000000
H	–5.591149000000	–2.000354000000	–3.868676000000
C	1.242373000000	0.036892000000	–0.010953000000
N	0.088945000000	–0.007905000000	0.003633000000
C	2.686886000000	0.114687000000	–0.034516000000
H	3.109934000000	–0.698372000000	0.569432000000
H	3.001735000000	1.081110000000	0.378660000000
H	3.037265000000	0.026802000000	–1.070794000000

L$_c$MeOH, zero imaginary frequencies
142
–2893.11033334

C	2.661491000000	–5.244036000000	–0.776138000000
C	2.416362000000	–6.617046000000	–0.118329000000
C	3.840056000000	–4.534348000000	–0.073456000000
C	3.046758000000	–5.472172000000	–2.245745000000
C	1.409867000000	–4.366047000000	–0.634551000000
C	0.915398000000	–4.055806000000	0.641548000000
C	–0.171522000000	–3.203653000000	0.835126000000
C	–0.787639000000	–2.621199000000	–0.292155000000
C	–0.331808000000	–2.914672000000	–1.590629000000
C	0.748704000000	–3.801756000000	–1.728013000000
C	–0.832497000000	–2.195208000000	–2.837755000000
O	–1.757675000000	–1.688044000000	–0.049877000000
C	–3.062352000000	–1.842829000000	–0.540008000000
H	3.313939000000	–7.251472000000	–0.201121000000
H	1.579229000000	–7.140535000000	–0.606239000000
H	2.173500000000	–6.519671000000	0.950289000000
H	4.763025000000	–5.129964000000	–0.166083000000
H	3.643406000000	–4.382581000000	0.998278000000
H	4.021367000000	–3.544604000000	–0.521157000000
H	2.244871000000	–5.979246000000	–2.804304000000
H	3.945379000000	–6.105823000000	–2.302086000000
H	3.277048000000	–4.525178000000	–2.757817000000
H	1.391203000000	–4.486927000000	1.525070000000
H	1.092080000000	–4.018660000000	–2.739126000000
H	–1.903621000000	–1.980263000000	–2.791796000000
H	–0.664710000000	–2.852769000000	–3.704416000000
H	–3.132622000000	–2.639897000000	–1.305141000000
H	–3.366765000000	–0.915728000000	–1.044785000000
C	–4.093085000000	–2.216213000000	0.510615000000
O	–3.812788000000	–2.711492000000	1.584553000000
C	–5.523008000000	–1.916948000000	0.126409000000
H	–6.192390000000	–2.683395000000	0.540889000000
H	–5.661377000000	–1.803254000000	–0.957090000000
H	–5.775039000000	–0.952785000000	0.601049000000
C	3.582694000000	0.133130000000	–3.647395000000
C	3.798770000000	–0.450350000000	–5.057861000000
C	4.230695000000	–0.790108000000	–2.591630000000
C	4.276796000000	1.501188000000	–3.562698000000
C	2.077607000000	0.238150000000	–3.364012000000
C	1.285832000000	–0.917892000000	–3.350154000000
C	–0.074949000000	–0.892645000000	–3.033612000000
C	–0.681828000000	0.347172000000	–2.767136000000
C	0.087601000000	1.520610000000	–2.713023000000
C	1.451274000000	1.440704000000	–3.026434000000
C	–0.477074000000	2.846718000000	–2.232682000000
O	–2.047100000000	0.381655000000	–2.553410000000
C	–2.786484000000	0.867358000000	–3.639672000000
H	4.875106000000	–0.541094000000	–5.277480000000
H	3.344779000000	0.196394000000	–5.825447000000
H	3.351656000000	–1.451443000000	–5.155293000000
H	5.309606000000	–0.897720000000	–2.790639000000
H	3.786044000000	–1.796911000000	–2.600021000000
H	4.098643000000	–0.362516000000	–1.587548000000
H	3.871751000000	2.213412000000	–4.298639000000
H	5.352774000000	1.386828000000	–3.766049000000
H	4.169960000000	1.935486000000	–2.557470000000
H	1.748582000000	–1.886663000000	–3.543017000000
H	2.034045000000	2.359023000000	–2.972072000000
H	–1.570523000000	2.827054000000	–2.213829000000
H	–0.152417000000	3.644156000000	–2.918847000000
H	–2.578526000000	0.286034000000	–4.564260000000
H	–2.542852000000	1.919986000000	–3.867604000000
C	–4.277693000000	0.760004000000	–3.381391000000
O	–4.741099000000	0.026072000000	–2.533579000000
C	–5.141852000000	1.641443000000	–4.246606000000
H	–6.171771000000	1.262228000000	–4.266490000000
H	–4.744554000000	1.744341000000	–5.268250000000
H	–5.129493000000	2.639625000000	–3.777112000000
C	2.954413000000	5.069640000000	0.773568000000
C	2.767927000000	6.469165000000	0.153709000000
C	4.084897000000	4.317880000000	0.035951000000
C	3.371799000000	5.237640000000	2.242431000000
C	1.656505000000	4.262396000000	0.631599000000
C	1.146952000000	3.978120000000	–0.645889000000
C	0.014937000000	3.182798000000	–0.838626000000
C	–0.632693000000	2.635350000000	0.288549000000
C	–0.160170000000	2.903890000000	1.586272000000
C	0.965388000000	3.732708000000	1.723964000000
C	–0.695946000000	2.203040000000	2.827256000000
O	–1.647229000000	1.753449000000	0.046424000000
C	–2.943517000000	1.970808000000	0.536996000000
H	3.699183000000	7.053540000000	0.232105000000
H	1.969527000000	7.023943000000	0.671228000000
H	2.498897000000	6.409897000000	–0.911619000000
H	5.041434000000	4.853662000000	0.149269000000
H	3.879251000000	4.229163000000	–1.041572000000
H	4.204445000000	3.299784000000	0.436575000000
H	2.607927000000	5.773749000000	2.826658000000
H	4.304388000000	5.819833000000	2.300209000000
H	3.557120000000	4.265419000000	2.724203000000
H	1.643855000000	4.384307000000	–1.529492000000
H	1.323535000000	3.925220000000	2.734525000000
H	–1.777788000000	2.050628000000	2.784627000000
H	–0.486763000000	2.841085000000	3.699558000000
H	–2.972962000000	2.759682000000	1.312810000000
H	–3.295693000000	1.054542000000	1.030081000000
C	–3.950100000000	2.412086000000	–0.510197000000
O	–3.640367000000	2.896969000000	–1.580904000000
C	–5.394855000000	2.197466000000	–0.125823000000
H	–6.016751000000	3.006167000000	–0.533960000000
H	–5.538185000000	2.084909000000	0.957131000000
H	–5.705735000000	1.253756000000	–0.606512000000
C	3.570385000000	–0.404878000000	3.649431000000

C	3.749402000000	–0.365990000000	5.181531000000
C	4.261653000000	0.822304000000	3.018124000000
C	4.254480000000	–1.669141000000	3.098796000000
C	2.067124000000	–0.396033000000	3.331906000000
C	1.352450000000	0.806137000000	3.294836000000
C	–0.014469000000	0.856904000000	3.007915000000
C	–0.702570000000	–0.345348000000	2.772708000000
C	–0.007972000000	–1.565837000000	2.727221000000
C	1.358406000000	–1.564529000000	3.033352000000
C	–0.648082000000	–2.851973000000	2.232585000000
O	–2.068264000000	–0.293572000000	2.564326000000
C	–2.837083000000	–0.745635000000	3.644069000000
H	4.819017000000	–0.355543000000	5.449951000000
H	3.283834000000	–1.244622000000	5.655534000000
H	3.280519000000	0.534182000000	5.608825000000
H	5.353143000000	0.745830000000	3.146066000000
H	3.948146000000	1.761156000000	3.499745000000
H	4.026350000000	0.899267000000	1.946138000000
H	3.882585000000	–2.586793000000	3.579092000000
H	5.337938000000	–1.619936000000	3.287946000000
H	4.103979000000	–1.765845000000	2.012536000000
H	1.874647000000	1.747568000000	3.457945000000
H	1.882615000000	–2.518248000000	2.984282000000
H	–1.738477000000	–2.769156000000	2.212569000000
H	–0.372421000000	–3.674530000000	2.910639000000
H	–2.594821000000	–0.189525000000	4.575793000000
H	–2.660793000000	–1.814156000000	3.859296000000
C	–4.317972000000	–0.542478000000	3.384390000000
O	–4.732377000000	0.222088000000	2.538108000000
C	–5.237999000000	–1.370420000000	4.244901000000
H	–6.241578000000	–0.926146000000	4.265232000000
H	–4.849531000000	–1.502874000000	5.266523000000
H	–5.288824000000	–2.365288000000	3.771050000000
O	2.675237000000	0.979062000000	–0.023266000000
H	2.159784000000	1.751541000000	–0.295422000000
C	1.797378000000	–0.135190000000	0.034383000000
H	2.341172000000	–0.966377000000	0.500217000000
H	1.473951000000	–0.460947000000	–0.961591000000
H	0.902335000000	0.069613000000	0.637342000000

Li**L**$_c$MeOH$^+$, zero imaginary frequencies
143
–2900.60196067

Li	2.578681000000	–0.024635000000	–0.003472000000
C	–3.063676000000	4.595754000000	1.309375000000
C	–3.032274000000	5.512082000000	2.549060000000
C	–4.106599000000	3.474745000000	1.514292000000
C	–3.487867000000	5.437075000000	0.095715000000
C	–1.690801000000	3.939625000000	1.106363000000
C	–1.099448000000	3.214632000000	2.152152000000
C	0.117545000000	2.545414000000	2.003693000000
C	0.758380000000	2.607934000000	0.756794000000
C	0.204613000000	3.308982000000	–0.318768000000
C	–1.011193000000	3.976494000000	–0.114659000000
C	0.799344000000	3.222963000000	–1.713271000000
O	1.991947000000	1.972428000000	0.605374000000
C	3.079599000000	2.830268000000	0.887610000000
H	–4.015027000000	5.986279000000	2.696583000000
H	–2.282441000000	6.309716000000	2.430707000000
H	–2.793762000000	4.956929000000	3.468390000000
H	–5.110243000000	3.904528000000	1.659681000000
H	–3.873394000000	2.861002000000	2.397345000000
H	–4.141863000000	2.806054000000	0.640459000000
H	–2.767088000000	6.241921000000	–0.115842000000
H	–4.463615000000	5.905776000000	0.291906000000
H	–3.597993000000	4.822719000000	–0.810744000000
H	–1.602779000000	3.160323000000	3.118277000000
H	–1.436877000000	4.524528000000	–0.953069000000
H	1.878931000000	3.045745000000	–1.671918000000
H	0.619944000000	4.169194000000	–2.243918000000
H	2.920959000000	3.358539000000	1.848087000000
H	3.205710000000	3.598480000000	0.107210000000
C	4.345839000000	2.020835000000	1.009635000000
O	4.294771000000	0.803533000000	1.014320000000
C	5.630839000000	2.785583000000	1.114981000000
H	6.416376000000	2.162401000000	1.561495000000
H	5.512855000000	3.725602000000	1.674540000000
H	5.923468000000	3.043170000000	0.083182000000
C	–3.430186000000	1.414383000000	–3.793671000000
C	–3.531151000000	2.505515000000	–4.877892000000
C	–4.189648000000	1.857553000000	–2.522366000000
C	–4.089398000000	0.130516000000	–4.319607000000
C	–1.959032000000	1.191218000000	–3.420833000000
C	–1.199743000000	2.250513000000	–2.906156000000
C	0.111514000000	2.088198000000	–2.451941000000
C	0.698498000000	0.816713000000	–2.554165000000
C	–0.015528000000	–0.266967000000	–3.083101000000
C	–1.335137000000	–0.056208000000	–3.505789000000
C	0.551255000000	–1.675768000000	–3.124226000000
O	2.007519000000	0.644074000000	–2.105092000000
C	2.997535000000	0.688774000000	–3.111052000000
H	–4.585073000000	2.682515000000	–5.143868000000
H	–2.993488000000	2.206722000000	–5.791401000000
H	–3.110008000000	3.463165000000	–4.536325000000
H	–5.256303000000	2.010650000000	–2.750619000000
H	–3.795322000000	2.804748000000	–2.124013000000
H	–4.101021000000	1.091004000000	–1.737928000000
H	–3.594844000000	–0.243219000000	–5.229775000000
H	–5.141260000000	0.330104000000	–4.572450000000
H	–4.078771000000	–0.666480000000	–3.560814000000
H	–1.655883000000	3.236386000000	–2.814900000000
H	–1.884801000000	–0.907983000000	–3.902057000000
H	1.642538000000	–1.646972000000	–3.048363000000
H	0.295376000000	–2.137083000000	–4.089203000000
H	2.615141000000	1.214706000000	–4.006204000000
H	3.284838000000	–0.326232000000	–3.435096000000
C	4.241814000000	1.438428000000	–2.662041000000
O	4.216202000000	2.223524000000	–1.738965000000
C	5.490616000000	1.146681000000	–3.450456000000
H	6.210604000000	1.967702000000	–3.341329000000
H	5.277436000000	0.960558000000	–4.514431000000
H	5.933135000000	0.226634000000	–3.032581000000
C	–3.235368000000	–4.535200000000	–1.326921000000
C	–3.082417000000	–5.628214000000	–2.405181000000
C	–4.257233000000	–3.477143000000	–1.797207000000
C	–3.773554000000	–5.183841000000	–0.042716000000
C	–1.875248000000	–3.859246000000	–1.102408000000
C	–1.261376000000	–3.157532000000	–2.153264000000
C	–0.027929000000	–2.515541000000	–2.002913000000
C	0.611860000000	–2.592559000000	–0.756823000000
C	0.033053000000	–3.265019000000	0.323620000000
C	–1.205740000000	–3.891201000000	0.125016000000
C	0.648953000000	–3.203962000000	1.709391000000
O	1.870348000000	–2.008620000000	–0.614252000000
C	2.916561000000	–2.913684000000	–0.908333000000
H	–4.049298000000	–6.123152000000	–2.587100000000
H	–2.359260000000	–6.396017000000	–2.088789000000
H	–2.735358000000	–5.211414000000	–3.362841000000
H	–5.246275000000	–3.941054000000	–1.935073000000
H	–3.970159000000	–3.030134000000	–2.761391000000
H	–4.352328000000	–2.665345000000	–1.060920000000
H	–3.103795000000	–5.972859000000	0.332347000000
H	–4.749904000000	–5.649011000000	–0.243908000000
H	–3.919607000000	–4.440789000000	0.756027000000
H	–1.751684000000	–3.112078000000	–3.127061000000
H	–1.651354000000	–4.414674000000	0.968566000000
H	1.735499000000	–3.082054000000	1.650841000000
H	0.430604000000	–4.139641000000	2.244073000000
H	2.725351000000	–3.432712000000	–1.867893000000
H	3.015443000000	–3.688126000000	–0.130231000000
C	4.217471000000	–2.163496000000	–1.041506000000
O	4.224055000000	–0.945267000000	–1.039389000000
C	5.463519000000	–2.987432000000	–1.166964000000
H	6.272275000000	–2.398809000000	–1.618879000000
H	5.294897000000	–3.917045000000	–1.730912000000
H	5.755613000000	–3.265341000000	–0.140316000000

C	–3.423042000000	–1.211701000000	3.925035000000
C	–3.504085000000	–2.214349000000	5.094330000000
C	–4.236358000000	–1.744121000000	2.723630000000
C	–4.040056000000	0.120949000000	4.375102000000
C	–1.958797000000	–1.049468000000	3.496639000000
C	–1.273285000000	–2.136540000000	2.939672000000
C	0.034430000000	–2.036914000000	2.460717000000
C	0.691100000000	–0.799968000000	2.567611000000
C	0.047207000000	0.312998000000	3.124422000000
C	–1.267211000000	0.161318000000	3.586825000000
C	0.674632000000	1.696170000000	3.131821000000
O	1.998800000000	–0.691686000000	2.093041000000
C	3.001411000000	–0.777599000000	3.083990000000
H	–4.551780000000	–2.363312000000	5.400019000000
H	–2.941364000000	–1.848871000000	5.967510000000
H	–3.093585000000	–3.196806000000	4.815599000000
H	–5.305771000000	–1.793331000000	2.981891000000
H	–3.920620000000	–2.758485000000	2.437547000000
H	–4.115436000000	–1.104782000000	1.836455000000
H	–3.520544000000	0.541383000000	5.249989000000
H	–5.091392000000	–0.033459000000	4.659703000000
H	–4.022216000000	0.869047000000	3.567692000000
H	–1.783143000000	–3.095169000000	2.843393000000
H	–1.762101000000	1.035599000000	4.005365000000
H	1.762851000000	1.620323000000	3.043154000000
H	0.452270000000	2.185667000000	4.091210000000
H	2.607070000000	–1.275732000000	3.989720000000
H	3.344853000000	0.224083000000	3.393883000000
C	4.198574000000	–1.592925000000	2.622738000000
O	4.119705000000	–2.380302000000	1.704382000000
C	5.471726000000	–1.362256000000	3.392051000000
H	6.145301000000	–2.221516000000	3.280830000000
H	5.283484000000	–1.155869000000	4.456956000000
H	5.956435000000	–0.470734000000	2.959438000000
O	–2.877814000000	–0.657494000000	–0.484981000000
H	–2.420061000000	–1.212159000000	–1.131673000000
C	–1.923496000000	0.198815000000	0.111685000000
H	–2.416434000000	0.731647000000	0.935165000000
H	–1.535796000000	0.951090000000	–0.593538000000
H	–1.074498000000	–0.359446000000	0.538644000000

Na**L**$_6$MeOH$^+$, zero imaginary frequencies
143
–3055.32968398

Na	2.645520000000	–0.032254000000	–0.001396000000
C	–3.164401000000	4.580092000000	0.985420000000
C	–3.184511000000	5.556729000000	2.178320000000
C	–4.181630000000	3.442567000000	1.224825000000
C	–3.583981000000	5.350683000000	–0.276124000000
C	–1.770561000000	3.952500000000	0.842404000000
C	–1.169696000000	3.321723000000	1.942221000000
C	0.074545000000	2.692087000000	1.857327000000
C	0.739777000000	2.710871000000	0.619872000000
C	0.157856000000	3.279642000000	–0.519820000000
C	–1.084283000000	3.913853000000	–0.374914000000
C	0.754377000000	3.088628000000	–1.907212000000
O	2.007098000000	2.140546000000	0.537946000000
C	3.061915000000	3.058582000000	0.726811000000
H	–4.181647000000	6.012656000000	2.279286000000
H	–2.451375000000	6.366223000000	2.037882000000
H	–2.955882000000	5.054052000000	3.129712000000
H	–5.198906000000	3.851183000000	1.331546000000
H	–3.948468000000	2.878481000000	2.140299000000
H	–4.182270000000	2.731619000000	0.384642000000
H	–2.873994000000	6.156936000000	–0.516944000000
H	–4.571318000000	5.809870000000	–0.119343000000
H	–3.668901000000	4.690108000000	–1.152056000000
H	–1.688171000000	3.311355000000	2.901486000000
H	–1.526823000000	4.373509000000	–1.256263000000
H	1.831833000000	2.903586000000	–1.845458000000
H	0.591169000000	4.000363000000	–2.500617000000
H	2.861216000000	3.710897000000	1.599639000000
H	3.188862000000	3.717258000000	–0.149617000000
C	4.363689000000	2.331765000000	0.991530000000
O	4.385205000000	1.133344000000	1.206211000000
C	5.606065000000	3.172865000000	0.954932000000
H	6.410352000000	2.696348000000	1.530090000000
H	5.430180000000	4.200507000000	1.306313000000
H	5.910607000000	3.224460000000	–0.104082000000
C	–3.480963000000	1.153442000000	–3.876623000000
C	–3.580774000000	2.176129000000	–5.025882000000
C	–4.241791000000	1.673709000000	–2.636155000000
C	–4.140206000000	–0.159622000000	–4.324595000000
C	–2.009399000000	0.953728000000	–3.491021000000
C	–1.253129000000	2.043392000000	–3.037321000000
C	0.062959000000	1.913479000000	–2.586055000000
C	0.656665000000	0.640688000000	–2.643043000000
C	–0.060319000000	–0.480167000000	–3.085117000000
C	–1.385144000000	–0.296758000000	–3.504764000000
C	0.513858000000	–1.889357000000	–3.030999000000
O	1.973418000000	0.500307000000	–2.217468000000
C	2.956761000000	0.540734000000	–3.227348000000
H	–4.634520000000	2.338087000000	–5.302026000000
H	–3.043827000000	1.820925000000	–5.919403000000
H	–3.157883000000	3.152224000000	–4.743867000000
H	–5.310035000000	1.803465000000	–2.871223000000
H	–3.854479000000	2.648330000000	–2.303084000000
H	–4.144743000000	0.962706000000	–1.802016000000
H	–3.643959000000	–0.589037000000	–5.208886000000
H	–5.191196000000	0.025212000000	–4.591825000000
H	–4.132684000000	–0.908909000000	–3.518740000000
H	–1.716280000000	3.028951000000	–2.995848000000
H	–1.938757000000	–1.170529000000	–3.842700000000
H	1.604506000000	–1.848386000000	–2.947178000000
H	0.265025000000	–2.413721000000	–3.965390000000
H	2.597227000000	1.117630000000	–4.100812000000
H	3.204519000000	–0.473167000000	–3.588106000000
C	4.224923000000	1.219588000000	–2.735358000000
O	4.222637000000	1.923407000000	–1.747359000000
C	5.462081000000	0.954444000000	–3.548060000000
H	6.203762000000	1.746922000000	–3.385829000000
H	5.241997000000	0.843818000000	–4.620895000000
H	5.879658000000	–0.002084000000	–3.190253000000
C	–3.316731000000	–4.548312000000	–1.020743000000
C	–3.179468000000	–5.717606000000	–2.018235000000
C	–4.328678000000	–3.516164000000	–1.563483000000
C	–3.858159000000	–5.098098000000	0.307492000000
C	–1.946261000000	–3.877284000000	–0.848980000000
C	–1.323316000000	–3.266572000000	–1.950206000000
C	–0.067421000000	–2.657278000000	–1.856544000000
C	0.591243000000	–2.689000000000	–0.616461000000
C	–0.016157000000	–3.227462000000	0.524279000000
C	–1.276608000000	–3.824673000000	0.377788000000
C	0.596171000000	–3.060026000000	1.906656000000
O	1.881009000000	–2.172953000000	–0.536613000000
C	2.890273000000	–3.141430000000	–0.731660000000
H	–4.152257000000	–6.213660000000	–2.161551000000
H	–2.463460000000	–6.468624000000	–1.649588000000
H	–2.831195000000	–5.374213000000	–3.004154000000
H	–5.322213000000	–3.979131000000	–1.668201000000
H	–4.038407000000	–3.141385000000	–2.556968000000
H	–4.415019000000	–2.654004000000	–0.885923000000
H	–3.194885000000	–5.864602000000	0.736876000000
H	–4.839409000000	–5.566916000000	0.141750000000
H	–3.995587000000	–4.298735000000	1.051357000000
H	–1.824260000000	–3.270991000000	–2.919545000000
H	–1.739281000000	–4.259181000000	1.261426000000
H	1.681242000000	–2.930719000000	1.835514000000
H	0.392049000000	–3.961471000000	2.503047000000
H	2.650068000000	–3.785780000000	–1.600303000000
H	2.992104000000	–3.802370000000	0.146139000000
C	4.224292000000	–2.481823000000	–1.011081000000
O	4.304415000000	–1.287178000000	–1.231746000000
C	5.422429000000	–3.385141000000	–0.982207000000
H	6.244427000000	–2.952246000000	–1.566718000000
H	5.191296000000	–4.403809000000	–1.327692000000
H	5.733591000000	–3.448126000000	0.074243000000

C	–3.485737000000	–0.930146000000	3.991403000000
C	–3.576978000000	–1.895045000000	5.191370000000
C	–4.304896000000	–1.491225000000	2.807457000000
C	–4.089394000000	0.421216000000	4.401612000000
C	–2.019674000000	–0.796486000000	3.558233000000
C	–1.339203000000	–1.915647000000	3.060096000000
C	–0.024817000000	–1.851586000000	2.593088000000
C	0.640855000000	–0.615140000000	2.660834000000
C	–0.005074000000	0.537273000000	3.129328000000
C	–1.325372000000	0.416365000000	3.584683000000
C	0.630991000000	1.917558000000	3.042314000000
O	1.956948000000	–0.543277000000	2.214577000000
C	2.949815000000	–0.620015000000	3.212911000000
H	–4.625950000000	–2.021553000000	5.502616000000
H	–3.008658000000	–1.509343000000	6.052129000000
H	–3.179326000000	–2.890884000000	4.943440000000
H	–5.374132000000	–1.526626000000	3.068852000000
H	–3.995083000000	–2.514621000000	2.548950000000
H	–4.181767000000	–0.878606000000	1.902135000000
H	–3.560711000000	0.865452000000	5.259070000000
H	–5.140236000000	0.284677000000	4.696919000000
H	–4.071268000000	1.142777000000	3.570657000000
H	–1.858496000000	–2.872216000000	3.006187000000
H	–1.823111000000	1.313848000000	3.946408000000
H	1.718341000000	1.828487000000	2.951535000000
H	0.413862000000	2.471526000000	3.967498000000
H	2.573930000000	–1.169433000000	4.097104000000
H	3.250992000000	0.384285000000	3.559508000000
C	4.177621000000	–1.363745000000	2.713366000000
O	4.131831000000	–2.070133000000	1.728049000000
C	5.433623000000	–1.157840000000	3.514202000000
H	6.131559000000	–1.989153000000	3.352097000000
H	5.228769000000	–1.027911000000	4.587849000000
H	5.897445000000	–0.227219000000	3.145249000000
O	–2.896807000000	–0.672881000000	–0.430240000000
H	–2.456973000000	–1.265857000000	–1.054821000000
C	–1.923277000000	0.204675000000	0.100163000000
H	–2.388869000000	0.770671000000	0.916640000000
H	–1.558204000000	0.926717000000	–0.646861000000
H	–1.061133000000	–0.337709000000	0.521650000000

K$\mathbf{L}_c$MeOH$^+$, zero imaginary frequencies
143
–3492.92817945

K	3.044843000000	–0.053192000000	–0.007603000000
C	–3.262637000000	4.526363000000	0.643029000000
C	–3.323903000000	5.558994000000	1.786284000000
C	–4.248146000000	3.372359000000	0.930265000000
C	–3.692338000000	5.223241000000	–0.657578000000
C	–1.849295000000	3.934799000000	0.541187000000
C	–1.226359000000	3.404740000000	1.680638000000
C	0.044780000000	2.824041000000	1.642443000000
C	0.719312000000	2.806273000000	0.407845000000
C	0.100174000000	3.233373000000	–0.776371000000
C	–1.168072000000	3.822517000000	–0.674524000000
C	0.688774000000	2.938951000000	–2.153264000000
O	2.029437000000	2.343254000000	0.378344000000
C	3.018721000000	3.347648000000	0.372969000000
H	–4.334283000000	5.991358000000	1.853227000000
H	–2.611295000000	6.380815000000	1.614994000000
H	–3.094820000000	5.110158000000	2.764089000000
H	–5.277854000000	3.755058000000	1.011187000000
H	–4.004331000000	2.860998000000	1.873317000000
H	–4.221304000000	2.621037000000	0.126922000000
H	–2.998503000000	6.031826000000	–0.935123000000
H	–4.690637000000	5.667153000000	–0.528575000000
H	–3.758353000000	4.518533000000	–1.499838000000
H	–1.748350000000	3.440522000000	2.636664000000
H	–1.635678000000	4.187840000000	–1.586208000000
H	1.766473000000	2.758306000000	–2.082678000000
H	0.527053000000	3.806382000000	–2.810795000000
H	2.705181000000	4.215479000000	0.985835000000
H	3.207509000000	3.724826000000	–0.648691000000
C	4.327600000000	2.836321000000	0.954478000000
O	4.387288000000	1.790445000000	1.573487000000
C	5.535142000000	3.687041000000	0.677637000000
H	6.327386000000	3.475986000000	1.407168000000
H	5.297123000000	4.761337000000	0.659170000000
H	5.893744000000	3.406981000000	–0.328441000000
C	–3.543034000000	0.840343000000	–3.968299000000
C	–3.629309000000	1.751983000000	–5.208841000000
C	–4.313709000000	1.475698000000	–2.789668000000
C	–4.202291000000	–0.506654000000	–4.299818000000
C	–2.073877000000	0.674711000000	–3.557113000000
C	–1.324056000000	1.800398000000	–3.188064000000
C	0.000072000000	1.714530000000	–2.750616000000
C	0.609018000000	0.445492000000	–2.743663000000
C	–0.113323000000	–0.716819000000	–3.055285000000
C	–1.445658000000	–0.570988000000	–3.469242000000
C	0.464851000000	–2.116805000000	–2.865638000000
O	1.947011000000	0.356686000000	–2.381592000000
C	2.873874000000	0.350572000000	–3.442021000000
H	–4.680191000000	1.893015000000	–5.506734000000
H	–3.088063000000	1.312378000000	–6.061307000000
H	–3.202212000000	2.747624000000	–5.015248000000
H	–5.381512000000	1.574340000000	–3.041172000000
H	–3.935198000000	2.481559000000	–2.554414000000
H	–4.214603000000	0.853368000000	–1.887754000000
H	–3.699597000000	–1.016991000000	–5.136142000000
H	–5.250281000000	–0.344922000000	–4.592612000000
H	–4.204770000000	–1.178767000000	–3.428840000000
H	–1.797524000000	2.781308000000	–3.210825000000
H	–2.001961000000	–1.470011000000	–3.725312000000
H	1.553258000000	–2.064471000000	–2.756561000000
H	0.237314000000	–2.721571000000	–3.756552000000
H	2.531417000000	0.996704000000	–4.274219000000
H	3.008399000000	–0.665714000000	–3.855949000000
C	4.226152000000	0.878832000000	–2.987270000000
O	4.357112000000	1.472509000000	–1.933218000000
C	5.380395000000	0.584846000000	–3.903437000000
H	6.203565000000	1.287005000000	–3.719546000000
H	5.088058000000	0.594602000000	–4.964372000000
H	5.723351000000	–0.436190000000	–3.660459000000
C	–3.445850000000	–4.505390000000	–0.657407000000
C	–3.324154000000	–5.759485000000	–1.548712000000
C	–4.439080000000	–3.510344000000	–1.294071000000
C	–4.003208000000	–4.928641000000	0.710061000000
C	–2.061303000000	–3.851126000000	–0.536383000000
C	–1.422455000000	–3.342512000000	–1.679350000000
C	–0.137890000000	–2.786789000000	–1.637907000000
C	0.535896000000	–2.785472000000	–0.403520000000
C	–0.101220000000	–3.178527000000	0.782460000000
C	–1.389176000000	–3.724213000000	0.683759000000
C	0.511399000000	–2.913320000000	2.153366000000
O	1.865314000000	–2.383493000000	–0.378365000000
C	2.802284000000	–3.438582000000	–0.372626000000
H	–4.304324000000	–6.250770000000	–1.653491000000
H	–2.621719000000	–6.486710000000	–1.112414000000
H	–2.966020000000	–5.508227000000	–2.558646000000
H	–5.437766000000	–3.967956000000	–1.368672000000
H	–4.136636000000	–3.225508000000	–2.313281000000
H	–4.520186000000	–2.593713000000	–0.691746000000
H	–3.357910000000	–5.669382000000	1.206741000000
H	–4.993642000000	–5.389301000000	0.579922000000
H	–4.126018000000	–4.065995000000	1.382269000000
H	–1.931140000000	–3.395621000000	–2.642960000000
H	–1.870558000000	–4.062860000000	1.598463000000
H	1.597758000000	–2.801005000000	2.074602000000
H	0.301103000000	–3.767639000000	2.814203000000
H	2.436804000000	–4.294576000000	–0.973044000000
H	2.981228000000	–3.814007000000	0.651121000000
C	4.130776000000	–3.005421000000	–0.972900000000
O	4.239819000000	–1.974416000000	–1.609649000000
C	5.293912000000	–3.914178000000	–0.691085000000
H	6.091010000000	–3.755407000000	–1.428506000000
H	5.000191000000	–4.974184000000	–0.655356000000
H	5.672556000000	–3.638365000000	0.308779000000

C	–3.537455000000	–0.560350000000	4.066092000000
C	–3.654312000000	–1.482147000000	5.297292000000
C	–4.376426000000	–1.134983000000	2.902973000000
C	–4.099588000000	0.820153000000	4.435879000000
C	–2.069789000000	–0.485044000000	3.623708000000
C	–1.411521000000	–1.650919000000	3.209327000000
C	–0.090720000000	–1.648330000000	2.756385000000
C	0.604001000000	–0.424017000000	2.757361000000
C	–0.030863000000	0.779865000000	3.098803000000
C	–1.356824000000	0.715084000000	3.552010000000
C	0.623979000000	2.140526000000	2.876862000000
O	1.939232000000	–0.422742000000	2.371920000000
C	2.880284000000	–0.455186000000	3.419278000000
H	–4.705140000000	–1.561699000000	5.617456000000
H	–3.068041000000	–1.088284000000	6.142187000000
H	–3.293884000000	–2.499288000000	5.081487000000
H	–5.443604000000	–1.149859000000	3.175023000000
H	–4.081113000000	–2.166811000000	2.661855000000
H	–4.254461000000	–0.544013000000	1.983460000000
H	–3.545208000000	1.280488000000	5.268292000000
H	–5.149079000000	0.722242000000	4.750735000000
H	–4.076930000000	1.510925000000	3.579681000000
H	–1.953043000000	–2.596357000000	3.218122000000
H	–1.844436000000	1.645084000000	3.835418000000
H	1.706600000000	2.027005000000	2.756916000000
H	0.441994000000	2.774267000000	3.758082000000
H	2.517333000000	–1.076425000000	4.261763000000
H	3.072744000000	0.555742000000	3.823296000000
C	4.197286000000	–1.055107000000	2.950920000000
O	4.284095000000	–1.660761000000	1.898888000000
C	5.377134000000	–0.814955000000	3.849738000000
H	6.159544000000	–1.561438000000	3.662463000000
H	5.098712000000	–0.799052000000	4.914294000000
H	5.770060000000	0.184095000000	3.591433000000
O	–2.985426000000	–0.631092000000	–0.377682000000
H	–2.599773000000	–1.278506000000	–0.983265000000
C	–1.939754000000	0.197907000000	0.091238000000
H	–2.352097000000	0.851188000000	0.869368000000
H	–1.525865000000	0.837507000000	–0.703609000000
H	–1.119960000000	–0.383677000000	0.543073000000

Rb**L**$_{\mathbf{c}}$MeOH$^+$, zero imaginary frequencies
143
–2917.16075189

Rb	3.251043000000	–0.059663000000	–0.011623000000
C	–3.360114000000	4.543713000000	0.492120000000
C	–3.417776000000	5.609982000000	1.604304000000
C	–4.349416000000	3.402356000000	0.814015000000
C	–3.788190000000	5.203473000000	–0.828251000000
C	–1.948204000000	3.945882000000	0.407335000000
C	–1.324143000000	3.451559000000	1.562042000000
C	–0.049700000000	2.876537000000	1.542727000000
C	0.627205000000	2.829105000000	0.310016000000
C	0.002895000000	3.208884000000	–0.887667000000
C	–1.268413000000	3.794279000000	–0.804865000000
C	0.596108000000	2.867098000000	–2.251199000000
O	1.955780000000	2.417128000000	0.295678000000
C	2.889083000000	3.475357000000	0.304033000000
H	–4.426727000000	6.047468000000	1.658574000000
H	–2.702551000000	6.424081000000	1.408635000000
H	–3.189924000000	5.189539000000	2.594929000000
H	–5.377718000000	3.790894000000	0.884415000000
H	–4.106498000000	2.918439000000	1.771579000000
H	–4.325911000000	2.627481000000	0.033458000000
H	–3.091661000000	6.001076000000	–1.129804000000
H	–4.784932000000	5.654370000000	–0.711957000000
H	–3.857041000000	4.474609000000	–1.649385000000
H	–1.846460000000	3.515555000000	2.516229000000
H	–1.737649000000	4.128975000000	–1.727260000000
H	1.671461000000	2.678935000000	–2.165424000000
H	0.444424000000	3.711975000000	–2.940011000000
H	2.444466000000	4.388836000000	0.741363000000
H	3.209190000000	3.736093000000	–0.723522000000
C	4.131130000000	3.139442000000	1.113673000000
O	4.233394000000	2.099255000000	1.736039000000
C	5.236456000000	4.162096000000	1.053978000000
H	5.865010000000	4.091066000000	1.951377000000
H	4.855401000000	5.186706000000	0.930439000000
H	5.863346000000	3.933166000000	0.174453000000
C	–3.652125000000	0.713192000000	–3.966241000000
C	–3.738727000000	1.576204000000	–5.241131000000
C	–4.418893000000	1.395881000000	–2.812081000000
C	–4.315637000000	–0.643855000000	–4.243483000000
C	–2.182000000000	0.559681000000	–3.553697000000
C	–1.428467000000	1.696434000000	–3.229044000000
C	–0.099373000000	1.625213000000	–2.803761000000
C	0.511108000000	0.357119000000	–2.766266000000
C	–0.217693000000	–0.814835000000	–3.021786000000
C	–1.555221000000	–0.682906000000	–3.423586000000
C	0.367214000000	–2.205144000000	–2.787841000000
O	1.866504000000	0.281215000000	–2.467873000000
C	2.716541000000	0.265644000000	–3.592134000000
H	–4.789866000000	1.708670000000	–5.542142000000
H	–3.200615000000	1.102163000000	–6.077003000000
H	–3.308173000000	2.577304000000	–5.087235000000
H	–5.486423000000	1.490107000000	–3.066480000000
H	–4.035460000000	2.407942000000	–2.614856000000
H	–4.322042000000	0.806520000000	–1.888152000000
H	–3.816814000000	–1.187370000000	–5.061022000000
H	–5.364235000000	–0.491077000000	–4.538982000000
H	–4.316369000000	–1.281245000000	–3.346965000000
H	–1.902075000000	2.675950000000	–3.281692000000
H	–2.115429000000	–1.589528000000	–3.640467000000
H	1.454020000000	–2.139187000000	–2.671561000000
H	0.149619000000	–2.839965000000	–3.660415000000
H	2.252071000000	0.794651000000	–4.446180000000
H	2.920031000000	–0.769178000000	–3.928525000000
C	4.045532000000	0.945156000000	–3.306928000000
O	4.255586000000	1.534999000000	–2.263963000000
C	5.086810000000	0.806285000000	–4.386630000000
H	5.808613000000	1.631190000000	–4.325153000000
H	4.643642000000	0.755353000000	–5.392454000000
H	5.626758000000	–0.141224000000	–4.214660000000
C	–3.553529000000	–4.514578000000	–0.510338000000
C	–3.425956000000	–5.801631000000	–1.352572000000
C	–4.545603000000	–3.546810000000	–1.189142000000
C	–4.116495000000	–4.886250000000	0.869765000000
C	–2.169717000000	–3.855375000000	–0.408232000000
C	–1.527713000000	–3.385276000000	–1.565804000000
C	–0.239680000000	–2.835641000000	–1.541595000000
C	0.434818000000	–2.803513000000	–0.307869000000
C	–0.208366000000	–3.148804000000	0.889808000000
C	–1.499786000000	–3.688857000000	0.808359000000
C	0.408896000000	–2.838598000000	2.248636000000
O	1.778663000000	–2.449223000000	–0.294130000000
C	2.663464000000	–3.549922000000	–0.275286000000
H	–4.404948000000	–6.298063000000	–1.443424000000
H	–2.724743000000	–6.510659000000	–0.885380000000
H	–3.062975000000	–5.588197000000	–2.369463000000
H	–5.543200000000	–4.008844000000	–1.249948000000
H	–4.239583000000	–3.301379000000	–2.217400000000
H	–4.630927000000	–2.607632000000	–0.623220000000
H	–3.473369000000	–5.607752000000	1.396650000000
H	–5.106440000000	–5.351578000000	0.753028000000
H	–4.241822000000	–3.998903000000	1.508459000000
H	–2.035307000000	–3.468246000000	–2.527785000000
H	–1.984010000000	–3.994574000000	1.733028000000
H	1.493951000000	–2.723262000000	2.158372000000
H	0.204734000000	–3.670018000000	2.940142000000
H	2.171869000000	–4.456261000000	–0.674962000000
H	2.984289000000	–3.786669000000	0.757341000000
C	3.911109000000	–3.301649000000	–1.107638000000
O	4.057278000000	–2.289121000000	–1.765504000000
C	4.966611000000	–4.373765000000	–1.020774000000
H	5.593005000000	–4.360701000000	–1.922352000000
H	4.537649000000	–5.374616000000	–0.863907000000
H	5.607701000000	–4.147966000000	–0.150717000000

C	–3.646092000000	–0.415463000000	4.062585000000
C	–3.763273000000	–1.295681000000	5.323921000000
C	–4.487306000000	–1.027262000000	2.920482000000
C	–4.205795000000	0.977247000000	4.387502000000
C	–2.178267000000	–0.357071000000	3.618070000000
C	–1.520698000000	–1.537192000000	3.245410000000
C	–0.195532000000	–1.553580000000	2.805231000000
C	0.504283000000	–0.332335000000	2.775857000000
C	–0.133011000000	0.884414000000	3.063324000000
C	–1.463241000000	0.838683000000	3.506343000000
C	0.532089000000	2.231772000000	2.796794000000
O	1.856389000000	–0.349704000000	2.451092000000
C	2.725339000000	–0.377470000000	3.560426000000
H	–4.813928000000	–1.362783000000	5.647567000000
H	–3.175515000000	–0.874894000000	6.154719000000
H	–3.404716000000	–2.320033000000	5.141688000000
H	–5.554061000000	–1.032383000000	3.194614000000
H	–4.192968000000	–2.066624000000	2.713076000000
H	–4.366190000000	–0.467485000000	1.981699000000
H	–3.649615000000	1.463834000000	5.203633000000
H	–5.255078000000	0.891356000000	4.706564000000
H	–4.183159000000	1.639519000000	3.509180000000
H	–2.065031000000	–2.480219000000	3.281866000000
H	–1.951262000000	1.778641000000	3.753165000000
H	1.612037000000	2.100540000000	2.671887000000
H	0.362516000000	2.896044000000	3.658047000000
H	2.243666000000	–0.868236000000	4.427669000000
H	3.000017000000	0.645168000000	3.883405000000
C	4.003944000000	–1.141402000000	3.259672000000
O	4.160671000000	–1.748765000000	2.217099000000
C	5.068968000000	–1.062114000000	4.321957000000
H	5.735002000000	–1.932401000000	4.256580000000
H	4.646311000000	–0.974596000000	5.334032000000
H	5.666086000000	–0.152903000000	4.132918000000
O	–3.092786000000	–0.643359000000	–0.352098000000
H	–2.698762000000	–1.314277000000	–0.925891000000
C	–2.054622000000	0.210727000000	0.088419000000
H	–2.476167000000	0.894225000000	0.834781000000
H	–1.638913000000	0.818315000000	–0.730161000000
H	–1.235008000000	–0.346328000000	0.569924000000

$\text{Cs}\mathbf{L}_{e}\text{MeOH}^{+}$, zero imaginary frequencies
143
–2913.2015456

Cs	3.510576000000	–0.106533000000	0.010440000000
C	–3.423979000000	4.621208000000	0.299438000000
C	–3.464636000000	5.730128000000	1.369880000000
C	–4.435813000000	3.512204000000	0.661090000000
C	–3.835996000000	5.237589000000	–1.046763000000
C	–2.023025000000	3.994933000000	0.242618000000
C	–1.412440000000	3.529561000000	1.416193000000
C	–0.146377000000	2.935014000000	1.424490000000
C	0.536486000000	2.835272000000	0.198506000000
C	–0.077114000000	3.184136000000	–1.014470000000
C	–1.339324000000	3.791568000000	–0.959640000000
C	0.515822000000	2.785647000000	–2.361987000000
O	1.865056000000	2.419790000000	0.201608000000
C	2.796522000000	3.479144000000	0.127966000000
H	–4.465533000000	6.187708000000	1.404811000000
H	–2.734208000000	6.522978000000	1.145179000000
H	–3.246201000000	5.343543000000	2.376295000000
H	–5.456759000000	3.922494000000	0.712986000000
H	–4.204914000000	3.060684000000	1.637140000000
H	–4.424801000000	2.707893000000	–0.089312000000
H	–3.124648000000	6.010914000000	–1.375722000000
H	–4.825100000000	5.709835000000	–0.951736000000
H	–3.914602000000	4.479161000000	–1.839830000000
H	–1.937150000000	3.636195000000	2.365199000000
H	–1.797497000000	4.102092000000	–1.896031000000
H	1.590115000000	2.595684000000	–2.271692000000
H	0.365719000000	3.601445000000	–3.085356000000
H	2.305448000000	4.449611000000	0.320344000000
H	3.240799000000	3.540827000000	–0.885212000000
C	3.939334000000	3.325320000000	1.120087000000
O	4.024396000000	2.368264000000	1.865998000000
C	4.990538000000	4.405717000000	1.066690000000
H	5.538087000000	4.443865000000	2.017460000000
H	4.565871000000	5.391864000000	0.827349000000
H	5.706295000000	4.152447000000	0.265077000000
C	–3.751303000000	0.589710000000	–3.968218000000
C	–3.832124000000	1.390724000000	–5.283351000000
C	–4.511364000000	1.334065000000	–2.848336000000
C	–4.426697000000	–0.773504000000	–4.177516000000
C	–2.281689000000	0.443529000000	–3.551342000000
C	–1.518632000000	1.587197000000	–3.278806000000
C	–0.187777000000	1.525186000000	–2.857209000000
C	0.415107000000	0.256362000000	–2.767632000000
C	–0.324820000000	–0.919820000000	–2.966385000000
C	–1.663325000000	–0.796284000000	–3.367430000000
C	0.254200000000	–2.301988000000	–2.678725000000
O	1.777946000000	0.182363000000	–2.496969000000
C	2.592023000000	0.099869000000	–3.645851000000
H	–4.882396000000	1.516879000000	–5.590183000000
H	–3.298356000000	0.872509000000	–6.095487000000
H	–3.393262000000	2.394556000000	–5.178088000000
H	–5.576960000000	1.430527000000	–3.110048000000
H	–4.115274000000	2.348948000000	–2.695519000000
H	–4.425243000000	0.784329000000	–1.899342000000
H	–3.935687000000	–1.359762000000	–4.969912000000
H	–5.475387000000	–0.626801000000	–4.475877000000
H	–4.427513000000	–1.365891000000	–3.250912000000
H	–1.985076000000	2.567138000000	–3.371828000000
H	–2.231106000000	–1.707422000000	–3.540626000000
H	1.339653000000	–2.233622000000	–2.556827000000
H	0.041524000000	–2.967343000000	–3.529722000000
H	2.049020000000	0.453815000000	–4.541524000000
H	2.890552000000	–0.948091000000	–3.845811000000
C	3.860089000000	0.927549000000	–3.517926000000
O	4.094631000000	1.603508000000	–2.533955000000
C	4.827164000000	0.810455000000	–4.668910000000
H	5.467936000000	1.700817000000	–4.713836000000
H	4.317056000000	0.654006000000	–5.630947000000
H	5.469910000000	–0.069150000000	–4.489766000000
C	–3.693560000000	–4.524880000000	–0.365282000000
C	–3.557255000000	–5.849701000000	–1.145208000000
C	–4.670151000000	–3.586160000000	–1.104567000000
C	–4.281006000000	–4.830158000000	1.020945000000
C	–2.308988000000	–3.866335000000	–0.270232000000
C	–1.651954000000	–3.437949000000	–1.435340000000
C	–0.363856000000	–2.886525000000	–1.416492000000
C	0.295450000000	–2.810069000000	–0.177060000000
C	–0.362470000000	–3.116691000000	1.023955000000
C	–1.652744000000	–3.659256000000	0.947699000000
C	0.240138000000	–2.758554000000	2.377086000000
O	1.638878000000	–2.457042000000	–0.146719000000
C	2.525629000000	–3.554057000000	–0.020150000000
H	–4.536822000000	–6.345873000000	–1.231620000000
H	–2.868258000000	–6.539440000000	–0.633077000000
H	–3.174295000000	–5.684069000000	–2.163720000000
H	–5.670132000000	–4.044297000000	–1.155067000000
H	–4.349646000000	–3.394335000000	–2.139639000000
H	–4.756305000000	–2.619180000000	–0.587538000000
H	–3.651497000000	–5.530943000000	1.590486000000
H	–5.272059000000	–5.294442000000	0.909312000000
H	–4.410126000000	–3.913798000000	1.616551000000
H	–2.147115000000	–3.556539000000	–2.400003000000
H	–2.147676000000	–3.931272000000	1.877377000000
H	1.328278000000	–2.664708000000	2.304502000000
H	0.008288000000	–3.554322000000	3.100959000000
H	1.973498000000	–4.508340000000	0.013467000000
H	3.087031000000	–3.472258000000	0.931715000000
C	3.562040000000	–3.605893000000	–1.135925000000
O	3.766615000000	–2.653233000000	–1.863975000000
C	4.329697000000	–4.897499000000	–1.243905000000
H	5.254010000000	–4.746881000000	–1.816178000000
H	3.701702000000	–5.636038000000	–1.771948000000
H	4.555881000000	–5.317566000000	–0.251300000000

C	–3.803113000000	–0.213031000000	4.034692000000
C	–3.934762000000	–1.026454000000	5.338884000000
C	–4.643152000000	–0.876236000000	2.920871000000
C	–4.352937000000	1.198881000000	4.284555000000
C	–2.331789000000	–0.189178000000	3.598790000000
C	–1.684176000000	–1.390423000000	3.281954000000
C	–0.352797000000	–1.441004000000	2.863205000000
C	0.363627000000	–0.231228000000	2.792140000000
C	–0.264775000000	1.003583000000	3.017470000000
C	–1.600789000000	0.992044000000	3.445178000000
C	0.419560000000	2.331038000000	2.705096000000
O	1.726328000000	–0.278299000000	2.512720000000
C	2.549683000000	–0.207618000000	3.655694000000
H	–4.987890000000	–1.069535000000	5.658753000000
H	–3.349030000000	–0.567677000000	6.150817000000
H	–3.582159000000	–2.061128000000	5.211439000000
H	–5.711963000000	–0.852913000000	3.185913000000
H	–4.361216000000	–1.929318000000	2.774158000000
H	–4.506977000000	–0.371305000000	1.953576000000
H	–3.799157000000	1.721079000000	5.080057000000
H	–5.405524000000	1.138242000000	4.598518000000
H	–4.316685000000	1.815899000000	3.374419000000
H	–2.239628000000	–2.325277000000	3.349598000000
H	–2.080063000000	1.947148000000	3.646479000000
H	1.498290000000	2.183932000000	2.591229000000
H	0.252805000000	3.029772000000	3.539382000000
H	1.992796000000	–0.505125000000	4.563440000000
H	2.909183000000	0.826231000000	3.825463000000
C	3.767340000000	–1.108707000000	3.546545000000
O	3.954914000000	–1.829671000000	2.584560000000
C	4.750784000000	–1.007012000000	4.684740000000
H	5.341095000000	–1.929939000000	4.755780000000
H	4.260085000000	–0.788153000000	5.644686000000
H	5.439377000000	–0.171537000000	4.468441000000
O	–3.207353000000	–0.647729000000	–0.335321000000
H	–2.792569000000	–1.351342000000	–0.852731000000
C	–2.188101000000	0.243479000000	0.076063000000
H	–2.632705000000	0.962446000000	0.774267000000
H	–1.762792000000	0.809491000000	–0.766972000000
H	–1.371858000000	–0.275039000000	0.603375000000

Mg**L**$_c$MeOH^{2+}, zero imaginary frequencies
143
–3092.87572841

Mg	2.546935000000	–0.034225000000	0.012070000000
C	–3.109179000000	4.718142000000	0.951820000000
C	–3.048679000000	5.771288000000	2.076984000000
C	–4.146192000000	3.628645000000	1.307091000000
C	–3.554569000000	5.411947000000	–0.344401000000
C	–1.742888000000	4.037700000000	0.801699000000
C	–1.147781000000	3.411583000000	1.908675000000
C	0.062356000000	2.721023000000	1.818331000000
C	0.683742000000	2.659677000000	0.564208000000
C	0.141318000000	3.266595000000	–0.568615000000
C	–1.069080000000	3.958574000000	–0.421309000000
C	0.734960000000	3.056969000000	–1.948312000000
O	1.940086000000	2.012142000000	0.465022000000
C	3.015358000000	2.901066000000	0.756483000000
H	–4.027802000000	6.261075000000	2.188236000000
H	–2.303179000000	6.549226000000	1.851236000000
H	–2.792597000000	5.326107000000	3.049993000000
H	–5.146792000000	4.075152000000	1.411693000000
H	–3.903613000000	3.127983000000	2.256716000000
H	–4.194977000000	2.858088000000	0.521701000000
H	–2.842962000000	6.190813000000	–0.658812000000
H	–4.528234000000	5.898108000000	–0.187859000000
H	–3.678630000000	4.696808000000	–1.172374000000
H	–1.644461000000	3.454833000000	2.878516000000
H	–1.494150000000	4.435339000000	–1.302400000000
H	1.818093000000	2.905796000000	–1.891366000000
H	0.546654000000	3.946814000000	–2.564688000000
H	2.785329000000	3.488785000000	1.662916000000
H	3.179737000000	3.611354000000	–0.069641000000
C	4.265428000000	2.106210000000	1.001635000000
O	4.197861000000	0.879959000000	0.986935000000
C	5.539791000000	2.837775000000	1.234961000000
H	6.256921000000	2.208499000000	1.777583000000
H	5.387786000000	3.799390000000	1.745810000000
H	5.955788000000	3.051776000000	0.234180000000
C	–3.458348000000	1.065800000000	–3.927714000000
C	–3.558383000000	2.116322000000	–5.051922000000
C	–4.236832000000	1.547672000000	–2.681025000000
C	–4.090482000000	–0.245378000000	–4.418291000000
C	–1.992191000000	0.878738000000	–3.520445000000
C	–1.245352000000	1.976632000000	–3.069039000000
C	0.056876000000	1.854179000000	–2.579904000000
C	0.634849000000	0.576438000000	–2.566991000000
C	–0.046657000000	–0.543995000000	–3.051833000000
C	–1.358898000000	–0.368450000000	–3.515228000000
C	0.533818000000	–1.945567000000	–3.011289000000
O	1.962069000000	0.436685000000	–2.092957000000
C	2.938665000000	0.529647000000	–3.132322000000
H	–4.610509000000	2.261068000000	–5.340230000000
H	–3.001933000000	1.794978000000	–5.945821000000
H	–3.165217000000	3.095647000000	–4.740298000000
H	–5.304585000000	1.662635000000	–2.921818000000
H	–3.869749000000	2.522106000000	–2.323904000000
H	–4.141691000000	0.824704000000	–1.856411000000
H	–3.571289000000	–0.647155000000	–5.302122000000
H	–5.136992000000	–0.067018000000	–4.704090000000
H	–4.096023000000	–1.017415000000	–3.633176000000
H	–1.703479000000	2.965455000000	–3.066790000000
H	–1.889984000000	–1.244444000000	–3.884172000000
H	1.627848000000	–1.905430000000	–2.963724000000
H	0.267337000000	–2.470089000000	–3.939247000000
H	2.493573000000	1.041897000000	–4.002295000000
H	3.245923000000	–0.473546000000	–3.472248000000
C	4.158506000000	1.321444000000	–2.703781000000
O	4.160041000000	1.929547000000	–1.653045000000
C	5.326775000000	1.292895000000	–3.644541000000
H	5.968248000000	2.168150000000	–3.479645000000
H	5.015432000000	1.236745000000	–4.698466000000
H	5.915563000000	0.385246000000	–3.424307000000
C	–3.241295000000	–4.662221000000	–1.009015000000
C	–3.107325000000	–5.818998000000	–2.020919000000
C	–4.248772000000	–3.615163000000	–1.535297000000
C	–3.777357000000	–5.224870000000	0.315931000000
C	–1.879452000000	–3.979823000000	–0.833674000000
C	–1.249684000000	–3.383267000000	–1.938393000000
C	–0.035326000000	–2.702446000000	–1.828531000000
C	0.566894000000	–2.642271000000	–0.565669000000
C	–0.010195000000	–3.220966000000	0.563425000000
C	–1.235827000000	–3.881676000000	0.403341000000
C	0.583313000000	–3.028827000000	1.944254000000
O	1.847003000000	–2.040565000000	–0.459527000000
C	2.885862000000	–2.973155000000	–0.749079000000
H	–4.081420000000	–6.310819000000	–2.164464000000
H	–2.392960000000	–6.576833000000	–1.663885000000
H	–2.765924000000	–5.467876000000	–3.006539000000
H	–5.247536000000	–4.067237000000	–1.632016000000
H	–3.964269000000	–3.239083000000	–2.530498000000
H	–4.317131000000	–2.756897000000	–0.850385000000
H	–3.106724000000	–5.987088000000	0.741582000000
H	–4.752213000000	–5.704228000000	0.146224000000
H	–3.927951000000	–4.431660000000	1.064545000000
H	–1.721335000000	–3.441973000000	–2.920538000000
H	–1.692482000000	–4.327647000000	1.284619000000
H	1.672634000000	–2.926793000000	1.889932000000
H	0.355837000000	–3.908464000000	2.561998000000
H	2.629734000000	–3.555563000000	–1.651838000000
H	3.022411000000	–3.686021000000	0.079949000000
C	4.167513000000	–2.232673000000	–1.000644000000
O	4.153184000000	–1.004581000000	–0.985723000000
C	5.408086000000	–3.018503000000	–1.240224000000
H	6.148147000000	–2.421298000000	–1.788326000000
H	5.212375000000	–3.973693000000	–1.748179000000
H	5.821106000000	–3.248090000000	–0.241658000000

C	–3.472507000000	–0.884380000000	4.044124000000
C	–3.552101000000	–1.878486000000	5.221121000000
C	–4.305052000000	–1.412446000000	2.853317000000
C	–4.060958000000	0.461356000000	4.492987000000
C	–2.015099000000	–0.749118000000	3.588692000000
C	–1.337528000000	–1.867705000000	3.084227000000
C	–0.038231000000	–1.798394000000	2.578657000000
C	0.599610000000	–0.550098000000	2.582709000000
C	–0.020185000000	0.592156000000	3.097350000000
C	–1.322368000000	0.465530000000	3.600200000000
C	0.607631000000	1.970776000000	3.019021000000
O	1.931313000000	–0.465064000000	2.100248000000
C	2.903251000000	–0.599775000000	3.139243000000
H	–4.596934000000	–1.998454000000	5.545814000000
H	–2.966864000000	–1.520734000000	6.082272000000
H	–3.174652000000	–2.874431000000	4.944039000000
H	–5.368235000000	–1.468201000000	3.132441000000
H	–3.989056000000	–2.421070000000	2.547851000000
H	–4.208363000000	–0.760035000000	1.972346000000
H	–3.516942000000	0.883771000000	5.351875000000
H	–5.106496000000	0.323073000000	4.803804000000
H	–4.056489000000	1.201119000000	3.677196000000
H	–1.844707000000	–2.832096000000	3.064212000000
H	–1.807037000000	1.358981000000	3.989303000000
H	1.699817000000	1.894790000000	2.968685000000
H	0.362717000000	2.529955000000	3.932483000000
H	2.435956000000	–1.091550000000	4.009210000000
H	3.254262000000	0.389095000000	3.479496000000
C	4.086741000000	–1.442897000000	2.709409000000
O	4.067705000000	–2.032996000000	1.648298000000
C	5.247833000000	–1.485857000000	3.658303000000
H	5.845221000000	–2.390447000000	3.486043000000
H	4.932238000000	–1.427295000000	4.710746000000
H	5.884033000000	–0.606991000000	3.453602000000
O	–2.670518000000	–0.785362000000	–0.392448000000
H	–2.428703000000	–1.029229000000	–1.295870000000
C	–1.805912000000	0.233961000000	0.043584000000
H	–2.068433000000	0.477044000000	1.079145000000
H	–1.888210000000	1.154306000000	–0.556443000000
H	–0.744946000000	–0.087223000000	0.039019000000

Ca**L**$_e$MeOH^{2+}, zero imaginary frequencies
143
–3570.43288106

Ca	2.822050000000	–0.041416000000	–0.000389000000
C	–3.228646000000	4.607714000000	0.339099000000
C	–3.258982000000	5.753277000000	1.370866000000
C	–4.209741000000	3.492064000000	0.765008000000
C	–3.679338000000	5.165195000000	–1.019870000000
C	–1.823694000000	3.995286000000	0.273931000000
C	–1.195283000000	3.555887000000	1.449275000000
C	0.057747000000	2.938005000000	1.447573000000
C	0.698999000000	2.773003000000	0.209359000000
C	0.099535000000	3.151131000000	–0.996175000000
C	–1.152606000000	3.778343000000	–0.933519000000
C	0.685434000000	2.769502000000	–2.346966000000
O	2.008709000000	2.251226000000	0.194203000000
C	3.006157000000	3.250441000000	0.364935000000
H	–4.265357000000	6.196371000000	1.412067000000
H	–2.548135000000	6.549254000000	1.100427000000
H	–3.013658000000	5.407443000000	2.386016000000
H	–5.236012000000	3.886356000000	0.820178000000
H	–3.954179000000	3.083384000000	1.754317000000
H	–4.199785000000	2.659361000000	0.044932000000
H	–2.993819000000	5.942382000000	–1.391470000000
H	–4.675253000000	5.620486000000	–0.921071000000
H	–3.759976000000	4.376499000000	–1.783359000000
H	–1.701555000000	3.691256000000	2.404836000000
H	–1.614797000000	4.091966000000	–1.867029000000
H	1.769566000000	2.631955000000	–2.267795000000
H	0.501306000000	3.580561000000	–3.065799000000
H	2.627550000000	4.060107000000	1.013428000000
H	3.277571000000	3.712782000000	–0.601906000000
C	4.243835000000	2.661883000000	1.000761000000
O	4.273659000000	1.472152000000	1.284002000000
C	5.405224000000	3.575599000000	1.226187000000
H	6.047397000000	3.186601000000	2.027090000000
H	5.095941000000	4.609174000000	1.438350000000
H	5.997356000000	3.589846000000	0.293543000000
C	–3.514492000000	0.534601000000	–4.043054000000
C	–3.610652000000	1.415865000000	–5.304804000000
C	–4.290840000000	1.190835000000	–2.877828000000
C	–4.153010000000	–0.829427000000	–4.344387000000
C	–2.047174000000	0.400637000000	–3.617679000000
C	–1.300301000000	1.550573000000	–3.319213000000
C	0.010641000000	1.495756000000	–2.841717000000
C	0.601881000000	0.228458000000	–2.705523000000
C	–0.093602000000	–0.950638000000	–2.992670000000
C	–1.416722000000	–0.836003000000	–3.445730000000
C	0.491832000000	–2.332273000000	–2.738021000000
O	1.940738000000	0.153105000000	–2.277423000000
C	2.895779000000	0.310972000000	–3.317113000000
H	–4.662621000000	1.526774000000	–5.608487000000
H	–3.059349000000	0.966853000000	–6.145357000000
H	–3.208757000000	2.426209000000	–5.135657000000
H	–5.359656000000	1.266702000000	–3.129410000000
H	–3.926729000000	2.208354000000	–2.670146000000
H	–4.188602000000	0.598195000000	–1.955969000000
H	–3.635134000000	–1.355185000000	–5.161454000000
H	–5.198278000000	–0.687846000000	–4.654451000000
H	–4.163616000000	–1.481640000000	–3.457750000000
H	–1.767765000000	2.528127000000	–3.432447000000
H	–1.959638000000	–1.751253000000	–3.673512000000
H	1.584207000000	–2.271522000000	–2.673151000000
H	0.242840000000	–2.988672000000	–3.583934000000
H	2.492417000000	0.959786000000	–4.114332000000
H	3.140768000000	–0.660520000000	–3.784717000000
C	4.161428000000	0.936412000000	–2.775423000000
O	4.235193000000	1.223888000000	–1.588909000000
C	5.292403000000	1.145197000000	–3.730346000000
H	5.960473000000	1.935714000000	–3.364531000000
H	4.948548000000	1.363812000000	–4.751663000000
H	5.869313000000	0.203711000000	–3.768086000000
C	–3.384429000000	–4.598690000000	–0.382286000000
C	–3.261909000000	–5.912607000000	–1.182456000000
C	–4.363570000000	–3.640243000000	–1.094980000000
C	–3.951546000000	–4.918708000000	1.008913000000
C	–2.003756000000	–3.935150000000	–0.300289000000
C	–1.352560000000	–3.523910000000	–1.474793000000
C	–0.095276000000	–2.912553000000	–1.461632000000
C	0.536944000000	–2.749754000000	–0.219149000000
C	–0.087811000000	–3.093212000000	0.982678000000
C	–1.355839000000	–3.686309000000	0.913270000000
C	0.511585000000	–2.733583000000	2.331836000000
O	1.868229000000	–2.282966000000	–0.199901000000
C	2.815549000000	–3.332785000000	–0.358930000000
H	–4.244232000000	–6.403394000000	–1.257930000000
H	–2.568562000000	–6.612466000000	–0.690880000000
H	–2.898606000000	–5.737767000000	–2.206625000000
H	–5.366676000000	–4.090791000000	–1.138776000000
H	–4.055453000000	–3.436839000000	–2.132259000000
H	–4.434982000000	–2.681639000000	–0.560357000000
H	–3.310604000000	–5.620276000000	1.564675000000
H	–4.939529000000	–5.390051000000	0.905666000000
H	–4.083540000000	–4.008231000000	1.613591000000
H	–1.838289000000	–3.684546000000	–2.438257000000
H	–1.840732000000	–3.964992000000	1.846248000000
H	1.602157000000	–2.658031000000	2.254033000000
H	0.282612000000	–3.530833000000	3.053141000000
H	2.398012000000	–4.127578000000	–1.001880000000
H	3.059114000000	–3.800535000000	0.612643000000
C	4.084644000000	–2.817118000000	–0.995136000000
O	4.180431000000	–1.633110000000	–1.287972000000
C	5.195288000000	–3.794462000000	–1.209311000000
H	5.858487000000	–3.448772000000	–2.012958000000
H	4.831404000000	–4.812150000000	–1.411252000000
H	5.784529000000	–3.830189000000	–0.275421000000

C	–3.504482000000	–0.267260000000	4.150699000000
C	–3.608340000000	–1.148391000000	5.412680000000
C	–4.357460000000	–0.875623000000	3.014232000000
C	–4.052372000000	1.128932000000	4.480897000000
C	–2.045593000000	–0.215994000000	3.681314000000
C	–1.391207000000	–1.403211000000	3.321089000000
C	–0.085507000000	–1.423991000000	2.828480000000
C	0.585589000000	–0.195926000000	2.710308000000
C	–0.025895000000	1.019405000000	3.032986000000
C	–1.335972000000	0.979589000000	3.531927000000
C	0.625538000000	2.361833000000	2.737382000000
O	1.924348000000	–0.199123000000	2.268548000000
C	2.870764000000	–0.404489000000	3.307784000000
H	–4.654231000000	–1.203295000000	5.751051000000
H	–3.005558000000	–0.734331000000	6.235677000000
H	–3.267121000000	–2.177824000000	5.226033000000
H	–5.419309000000	–0.886907000000	3.303954000000
H	–4.061914000000	–1.911482000000	2.791013000000
H	–4.255919000000	–0.302149000000	2.080755000000
H	–3.483520000000	1.614458000000	5.288826000000
H	–5.096178000000	1.047416000000	4.816601000000
H	–4.043640000000	1.790141000000	3.600772000000
H	–1.924184000000	–2.349294000000	3.410512000000
H	–1.813197000000	1.922474000000	3.790397000000
H	1.713338000000	2.247913000000	2.665971000000
H	0.416001000000	3.055061000000	3.564357000000
H	2.434305000000	–1.030750000000	4.105664000000
H	3.165251000000	0.553253000000	3.775429000000
C	4.102463000000	–1.094965000000	2.768436000000
O	4.164108000000	–1.384356000000	1.581416000000
C	5.218734000000	–1.365053000000	3.725289000000
H	5.843822000000	–2.190318000000	3.360197000000
H	4.861712000000	–1.565253000000	4.745827000000
H	5.845626000000	–0.456153000000	3.764462000000
O	–2.809464000000	–0.750904000000	–0.277647000000
H	–2.532134000000	–1.199149000000	–1.087606000000
C	–1.853564000000	0.234384000000	0.035262000000
H	–2.139757000000	0.687613000000	0.991185000000
H	–1.804810000000	1.033968000000	–0.720359000000
H	–0.839803000000	–0.191371000000	0.160680000000

Sr**L**$_c$MeOH^{2+}, zero imaginary frequencies
143
–2923.62312286

Sr	2.813730000000	–0.040943000000	–0.006624000000
C	–3.352546000000	4.539592000000	0.588015000000
C	–3.404727000000	5.594237000000	1.711822000000
C	–4.333014000000	3.388135000000	0.904949000000
C	–3.788130000000	5.208849000000	–0.724902000000
C	–1.941816000000	3.943928000000	0.490544000000
C	–1.311610000000	3.438623000000	1.638266000000
C	–0.043770000000	2.851430000000	1.605734000000
C	0.613165000000	2.799658000000	0.364202000000
C	0.000682000000	3.216725000000	–0.823741000000
C	–1.267037000000	3.807417000000	–0.726685000000
C	0.591579000000	2.915247000000	–2.196233000000
O	1.945996000000	2.345317000000	0.328819000000
C	2.900484000000	3.372895000000	0.558398000000
H	–4.413184000000	6.030324000000	1.771618000000
H	–2.691904000000	6.411592000000	1.522431000000
H	–3.177482000000	5.165079000000	2.698974000000
H	–5.361462000000	3.772714000000	0.983339000000
H	–4.085691000000	2.897458000000	1.858342000000
H	–4.312441000000	2.620866000000	0.116238000000
H	–3.098085000000	6.013474000000	–1.022384000000
H	–4.785186000000	5.655012000000	–0.599465000000
H	–3.860986000000	4.487283000000	–1.552552000000
H	–1.827029000000	3.500765000000	2.596187000000
H	–1.737880000000	4.162259000000	–1.640595000000
H	1.675117000000	2.768898000000	–2.121342000000
H	0.413743000000	3.769942000000	–2.864637000000
H	2.495972000000	4.120916000000	1.263654000000
H	3.139037000000	3.912876000000	–0.377191000000
C	4.175179000000	2.806933000000	1.145489000000
O	4.269514000000	1.608070000000	1.372386000000
C	5.294442000000	3.767472000000	1.397173000000
H	5.930193000000	3.406142000000	2.216393000000
H	4.940347000000	4.788476000000	1.599172000000
H	5.913814000000	3.802129000000	0.482932000000
C	–3.622190000000	0.783300000000	–3.996872000000
C	–3.699967000000	1.688820000000	–5.242960000000
C	–4.396302000000	1.426446000000	–2.823900000000
C	–4.277069000000	–0.566722000000	–4.324518000000
C	–2.156605000000	0.622414000000	–3.575186000000
C	–1.404553000000	1.753642000000	–3.223320000000
C	–0.084912000000	1.673627000000	–2.772690000000
C	0.511894000000	0.400525000000	–2.734804000000
C	–0.200151000000	–0.765083000000	–3.044677000000
C	–1.531152000000	–0.623265000000	–3.464860000000
C	0.379508000000	–2.162497000000	–2.852867000000
O	1.868668000000	0.304784000000	–2.374631000000
C	2.779310000000	0.524072000000	–3.441357000000
H	–4.748941000000	1.822543000000	–5.548096000000
H	–3.152698000000	1.246774000000	–6.089917000000
H	–3.281430000000	2.688775000000	–5.052920000000
H	–5.462002000000	1.522569000000	–3.081901000000
H	–4.021080000000	2.434940000000	–2.593957000000
H	–4.306056000000	0.811029000000	–1.916165000000
H	–3.769429000000	–1.081324000000	–5.155097000000
H	–5.322010000000	–0.406978000000	–4.626818000000
H	–4.289762000000	–1.234937000000	–3.450148000000
H	–1.874793000000	2.734851000000	–3.272686000000
H	–2.086416000000	–1.523534000000	–3.718623000000
H	1.471659000000	–2.108735000000	–2.778195000000
H	0.134568000000	–2.775271000000	–3.732407000000
H	2.355781000000	1.238463000000	–4.169799000000
H	2.981200000000	–0.413267000000	–3.993290000000
C	4.085667000000	1.087816000000	–2.923122000000
O	4.228528000000	1.309759000000	–1.728258000000
C	5.172346000000	1.321921000000	–3.924608000000
H	5.838169000000	2.126124000000	–3.584406000000
H	4.783247000000	1.536626000000	–4.930218000000
H	5.770979000000	0.395484000000	–3.987907000000
C	–3.514741000000	–4.529254000000	–0.610912000000
C	–3.375832000000	–5.804410000000	–1.470184000000
C	–4.503939000000	–3.552875000000	–1.282801000000
C	–4.081024000000	–4.919148000000	0.762631000000
C	–2.135497000000	–3.865844000000	–0.495555000000
C	–1.490036000000	–3.382146000000	–1.646249000000
C	–0.212605000000	–2.811996000000	–1.609044000000
C	0.442516000000	–2.770679000000	–0.366984000000
C	–0.188250000000	–3.154183000000	0.821977000000
C	–1.473512000000	–3.706233000000	0.726563000000
C	0.424331000000	–2.878492000000	2.188920000000
O	1.793349000000	–2.373874000000	–0.335369000000
C	2.695832000000	–3.451245000000	–0.553839000000
H	–4.352162000000	–6.303110000000	–1.568148000000
H	–2.673882000000	–6.517304000000	–1.010575000000
H	–3.014494000000	–5.577768000000	–2.484876000000
H	–5.498305000000	–4.018271000000	–1.356980000000
H	–4.193946000000	–3.292217000000	–2.306439000000
H	–4.599878000000	–2.623940000000	–0.701697000000
H	–3.439777000000	–5.647362000000	1.282537000000
H	–5.068415000000	–5.385838000000	0.635914000000
H	–4.213991000000	–4.040350000000	1.411983000000
H	–1.991099000000	–3.464456000000	–2.611495000000
H	–1.959403000000	–4.032026000000	1.643320000000
H	1.514020000000	–2.795126000000	2.106018000000
H	0.203059000000	–3.720210000000	2.860755000000
H	2.250358000000	–4.188283000000	–1.245721000000
H	2.911408000000	–3.988434000000	0.388799000000
C	3.995178000000	–2.962040000000	–1.155676000000
O	4.152848000000	–1.773426000000	–1.399842000000
C	5.059420000000	–3.985204000000	–1.399364000000
H	5.708467000000	–3.670368000000	–2.227307000000
H	4.649959000000	–4.988567000000	–1.584323000000
H	5.681693000000	–4.039483000000	–0.488057000000

C	–3.609395000000	–0.511704000000	4.103832000000
C	–3.702568000000	–1.420491000000	5.347221000000
C	–4.456402000000	–1.105749000000	2.955922000000
C	–4.173214000000	0.870721000000	4.463512000000
C	–2.148918000000	–0.434296000000	3.642232000000
C	–1.486249000000	–1.602770000000	3.238575000000
C	–0.170787000000	–1.600181000000	2.771251000000
C	0.506957000000	–0.368226000000	2.744053000000
C	–0.121349000000	0.835406000000	3.086191000000
C	–1.443649000000	0.769223000000	3.549182000000
C	0.528883000000	2.195265000000	2.858173000000
O	1.862160000000	–0.354904000000	2.361488000000
C	2.770234000000	–0.619401000000	3.419987000000
H	–4.748106000000	–1.497968000000	5.682370000000
H	–3.107144000000	–1.015168000000	6.179882000000
H	–3.345164000000	–2.440331000000	5.138971000000
H	–5.518871000000	–1.124759000000	3.242759000000
H	–4.158840000000	–2.138804000000	2.722450000000
H	–4.353415000000	–0.521664000000	2.029481000000
H	–3.613851000000	1.343342000000	5.285571000000
H	–5.217735000000	0.770784000000	4.791839000000
H	–4.166814000000	1.551887000000	3.598917000000
H	–2.021389000000	–2.550998000000	3.271836000000
H	–1.933290000000	1.698175000000	3.832084000000
H	1.616100000000	2.084423000000	2.772859000000
H	0.327131000000	2.840030000000	3.725720000000
H	2.317295000000	–1.310997000000	4.152856000000
H	3.024368000000	0.306319000000	3.970060000000
C	4.041287000000	–1.249853000000	2.891761000000
O	4.163068000000	–1.479278000000	1.695726000000
C	5.122597000000	–1.539705000000	3.884370000000
H	5.742645000000	–2.377911000000	3.539734000000
H	4.731395000000	–1.732651000000	4.893546000000
H	5.769338000000	–0.645738000000	3.941113000000
O	–3.055779000000	–0.660217000000	–0.339672000000
H	–2.697398000000	–1.256550000000	–1.010281000000
C	–2.025498000000	0.205562000000	0.080801000000
H	–2.419773000000	0.826058000000	0.894143000000
H	–1.687107000000	0.878946000000	–0.722519000000
H	–1.154249000000	–0.344919000000	0.477824000000

Ba**L**$_c$MeOH^{2+}, zero imaginary frequencies
143
–2918.36639159

Ba	–2.892851000000	0.037857000000	–0.013026000000
C	3.490520000000	–4.465724000000	0.670140000000
C	3.568005000000	–5.476708000000	1.831894000000
C	4.456132000000	–3.289319000000	0.936359000000
C	3.925199000000	–5.178566000000	–0.620134000000
C	2.070112000000	–3.895192000000	0.561467000000
C	1.430355000000	–3.379214000000	1.698707000000
C	0.147185000000	–2.826365000000	1.657758000000
C	–0.518093000000	–2.830629000000	0.417946000000
C	0.112694000000	–3.234019000000	–0.767034000000
C	1.396190000000	–3.787907000000	–0.658992000000
C	–0.478411000000	–2.958443000000	–2.147690000000
O	–1.878643000000	–2.471032000000	0.385986000000
C	–2.744219000000	–3.568389000000	0.642736000000
H	4.582793000000	–5.896984000000	1.896968000000
H	2.864336000000	–6.309899000000	1.681101000000
H	3.345985000000	–5.014116000000	2.805021000000
H	5.490876000000	–3.655410000000	1.020759000000
H	4.207861000000	–2.768350000000	1.873189000000
H	4.418008000000	–2.551248000000	0.121308000000
H	3.240387000000	–5.999295000000	–0.883904000000
H	4.927013000000	–5.610865000000	–0.484398000000
H	3.987466000000	–4.487664000000	–1.474163000000
H	1.949636000000	–3.409260000000	2.655597000000
H	1.879476000000	–4.142320000000	–1.566166000000
H	–1.562114000000	–2.807722000000	–2.076967000000
H	–0.302870000000	–3.826912000000	–2.799252000000
H	–2.302580000000	–4.236718000000	1.404384000000
H	–2.891267000000	–4.181177000000	–0.266897000000
C	–4.090246000000	–3.099309000000	1.153284000000
O	–4.304943000000	–1.909582000000	1.336552000000
C	–5.127540000000	–4.153824000000	1.389107000000
H	–5.824625000000	–3.830715000000	2.173730000000
H	–4.689546000000	–5.131811000000	1.635138000000
H	–5.705858000000	–4.271895000000	0.455253000000
C	3.743947000000	–0.851014000000	–3.967648000000
C	3.811312000000	–1.757763000000	–5.213767000000
C	4.521793000000	–1.496767000000	–2.799315000000
C	4.402895000000	0.495779000000	–4.299876000000
C	2.279524000000	–0.683898000000	–3.544042000000
C	1.528257000000	–1.809832000000	–3.174668000000
C	0.200233000000	–1.727263000000	–2.749595000000
C	–0.406972000000	–0.456900000000	–2.764111000000
C	0.313508000000	0.708630000000	–3.060580000000
C	1.652336000000	0.562257000000	–3.454402000000
C	–0.266598000000	2.110408000000	–2.885354000000
O	–1.784539000000	–0.369339000000	–2.496610000000
C	–2.602798000000	–0.611206000000	–3.631395000000
H	4.858194000000	–1.898286000000	–5.523168000000
H	3.263177000000	–1.312993000000	–6.058770000000
H	3.386825000000	–2.754685000000	–5.020743000000
H	5.586567000000	–1.592452000000	–3.061195000000
H	4.148120000000	–2.506082000000	–2.571303000000
H	4.433111000000	–0.883681000000	–1.890070000000
H	3.895623000000	1.010573000000	–5.130563000000
H	5.446656000000	0.331766000000	–4.603933000000
H	4.419506000000	1.165450000000	–3.426845000000
H	2.004380000000	–2.788824000000	–3.196265000000
H	2.211804000000	1.459006000000	–3.710002000000
H	–1.358891000000	2.058891000000	–2.806526000000
H	–0.024582000000	2.712045000000	–3.773758000000
H	–2.147150000000	–1.381416000000	–4.280096000000
H	–2.707005000000	0.300826000000	–4.249482000000
C	–3.977182000000	–1.095810000000	–3.218004000000
O	–4.241561000000	–1.277729000000	–2.038237000000
C	–4.976694000000	–1.307425000000	–4.313248000000
H	–5.705069000000	–2.074680000000	–4.018847000000
H	–4.505360000000	–1.564780000000	–5.272713000000
H	–5.526690000000	–0.359852000000	–4.454970000000
C	3.645963000000	4.471752000000	–0.660324000000
C	3.518257000000	5.724348000000	–1.554126000000
C	4.639936000000	3.477412000000	–1.297023000000
C	4.200104000000	4.897793000000	0.707434000000
C	2.262288000000	3.817598000000	–0.540753000000
C	1.619756000000	3.315637000000	–1.685190000000
C	0.327606000000	2.778516000000	–1.649429000000
C	–0.345123000000	2.794785000000	–0.414615000000
C	0.291727000000	3.168981000000	0.775858000000
C	1.590379000000	3.689155000000	0.679518000000
C	–0.328909000000	2.916947000000	2.146339000000
O	–1.718340000000	2.488158000000	–0.396382000000
C	–2.531896000000	3.629402000000	–0.636714000000
H	4.496311000000	6.219200000000	–1.654421000000
H	2.812393000000	6.449834000000	–1.120950000000
H	3.167711000000	5.471761000000	–2.566464000000
H	5.634459000000	3.941264000000	–1.378398000000
H	4.336703000000	3.187606000000	–2.314788000000
H	4.732853000000	2.565470000000	–0.689321000000
H	3.554513000000	5.639654000000	1.202039000000
H	5.188972000000	5.360375000000	0.577302000000
H	4.327029000000	4.036875000000	1.381319000000
H	2.132598000000	3.365175000000	–2.646301000000
H	2.077878000000	4.020691000000	1.593039000000
H	–1.418278000000	2.827726000000	2.060486000000
H	–0.114331000000	3.771834000000	2.804085000000
H	–2.052184000000	4.293043000000	–1.379118000000
H	–2.659596000000	4.228286000000	0.284962000000
C	–3.893608000000	3.237597000000	–1.170229000000
O	–4.165006000000	2.064543000000	–1.382040000000
C	–4.874939000000	4.347384000000	–1.391941000000
H	–5.575544000000	4.078615000000	–2.193779000000
H	–4.387082000000	5.308990000000	–1.606582000000
H	–5.460347000000	4.468858000000	–0.462963000000

C	3.718318000000	0.571895000000	4.067388000000
C	3.827998000000	1.528161000000	5.273254000000
C	4.564286000000	1.111692000000	2.892514000000
C	4.271035000000	–0.800217000000	4.479799000000
C	2.254347000000	0.490477000000	3.617284000000
C	1.591452000000	1.653012000000	3.197796000000
C	0.267400000000	1.648566000000	2.754929000000
C	–0.420453000000	0.420367000000	2.776285000000
C	0.216258000000	–0.783615000000	3.107643000000
C	1.546965000000	–0.712894000000	3.546022000000
C	–0.434931000000	–2.148557000000	2.897177000000
O	–1.795749000000	0.416266000000	2.479301000000
C	–2.618535000000	0.707076000000	3.598847000000
H	4.875854000000	1.605491000000	5.601007000000
H	3.231245000000	1.163809000000	6.123725000000
H	3.483166000000	2.543702000000	5.026558000000
H	5.627382000000	1.142057000000	3.176275000000
H	4.266789000000	2.133113000000	2.612753000000
H	4.458861000000	0.487012000000	1.993431000000
H	3.706595000000	–1.237060000000	5.318041000000
H	5.316094000000	–0.696223000000	4.805147000000
H	4.260729000000	–1.513408000000	3.641702000000
H	2.132785000000	2.597846000000	3.202592000000
H	2.041232000000	–1.637961000000	3.832038000000
H	–1.521771000000	–2.039292000000	2.801313000000
H	–0.241078000000	–2.779389000000	3.777111000000
H	–2.132125000000	1.453338000000	4.253293000000
H	–2.783560000000	–0.195072000000	4.218339000000
C	–3.956688000000	1.265367000000	3.161028000000
O	–4.192199000000	1.454450000000	1.976021000000
C	–4.960004000000	1.539708000000	4.238728000000
H	–5.638175000000	2.346084000000	3.929281000000
H	–4.490418000000	1.774166000000	5.204864000000
H	–5.566045000000	0.626120000000	4.374971000000
O	3.183027000000	0.607741000000	–0.366437000000
H	2.846454000000	1.220595000000	–1.033248000000
C	2.107637000000	–0.182817000000	0.088835000000
H	2.483562000000	–0.830235000000	0.889337000000
H	1.698245000000	–0.830474000000	–0.702188000000
H	1.290822000000	0.427564000000	0.511061000000

Li**L**$_c$MeOH^{+}, zero imaginary frequencies
143
–2900.60279901

Li	2.062485000000	0.072514000000	–0.271942000000
C	–3.612046000000	–3.449813000000	–2.768705000000
C	–3.722125000000	–3.837127000000	–4.257401000000
C	–4.558403000000	–2.266627000000	–2.466525000000
C	–4.054919000000	–4.651157000000	–1.919706000000
C	–2.174746000000	–3.013858000000	–2.449738000000
C	–1.597768000000	–1.947896000000	–3.158384000000
C	–0.318670000000	–1.465546000000	–2.875014000000
C	0.404558000000	–2.087144000000	–1.844464000000
C	–0.129520000000	–3.142902000000	–1.097260000000
C	–1.413517000000	–3.599486000000	–1.433053000000
C	0.574460000000	–3.683087000000	0.142314000000
O	1.657652000000	–1.585200000000	–1.511688000000
C	2.746233000000	–2.219589000000	–2.143735000000
H	–4.751367000000	–4.147033000000	–4.497005000000
H	–3.048527000000	–4.674656000000	–4.496191000000
H	–3.465645000000	–2.997977000000	–4.921089000000
H	–5.602838000000	–2.544517000000	–2.678811000000
H	–4.315843000000	–1.383281000000	–3.076704000000
H	–4.490660000000	–1.972149000000	–1.407136000000
H	–3.413020000000	–5.529571000000	–2.087172000000
H	–5.083737000000	–4.935734000000	–2.186026000000
H	–4.047715000000	–4.417197000000	–0.843861000000
H	–2.166164000000	–1.462996000000	–3.953674000000
H	–1.825001000000	–4.428727000000	–0.860101000000
H	1.660026000000	–3.559765000000	0.069549000000
H	0.355260000000	–4.756790000000	0.236362000000
H	2.614728000000	–2.232833000000	–3.243528000000
H	2.867954000000	–3.263541000000	–1.811239000000
C	4.000325000000	–1.441725000000	–1.820180000000
O	3.920669000000	–0.395268000000	–1.201205000000
C	5.301827000000	–2.014522000000	–2.294744000000
H	6.088030000000	–1.248885000000	–2.277531000000
H	5.214959000000	–2.463064000000	–3.296090000000
H	5.569276000000	–2.817613000000	–1.588449000000
C	–3.260115000000	–2.747058000000	3.308179000000
C	–3.415200000000	–4.233918000000	3.686233000000
C	–4.181035000000	–2.414752000000	2.112642000000
C	–3.692840000000	–1.892533000000	4.509212000000
C	–1.810766000000	–2.463811000000	2.889497000000
C	–1.228129000000	–3.207221000000	1.854893000000
C	0.066015000000	–2.961219000000	1.384267000000
C	0.809205000000	–1.934482000000	2.000519000000
C	0.242407000000	–1.122263000000	2.999427000000
C	–1.051446000000	–1.426127000000	3.441897000000
C	0.922211000000	0.149935000000	3.489565000000
O	2.079266000000	–1.661765000000	1.522245000000
C	3.144670000000	–2.116188000000	2.332613000000
H	–4.453902000000	–4.444582000000	3.984815000000
H	–2.757528000000	–4.496389000000	4.529268000000
H	–3.170908000000	–4.901411000000	2.846572000000
H	–5.234772000000	–2.598356000000	2.375216000000
H	–3.941063000000	–3.029035000000	1.231554000000
H	–4.078948000000	–1.357471000000	1.821415000000
H	–3.054997000000	–2.071832000000	5.388345000000
H	–4.726538000000	–2.142581000000	4.790474000000
H	–3.668783000000	–0.816910000000	4.277599000000
H	–1.805641000000	–3.996845000000	1.374368000000
H	–1.482028000000	–0.801639000000	4.221838000000
H	1.991751000000	0.117602000000	3.262715000000
H	0.809057000000	0.217064000000	4.581887000000
H	2.801175000000	–2.926352000000	3.003445000000
H	3.537250000000	–1.304011000000	2.962370000000
C	4.276208000000	–2.690795000000	1.497683000000
O	4.088612000000	–3.109935000000	0.374382000000
C	5.624199000000	–2.709738000000	2.162998000000
H	6.289000000000	–3.427709000000	1.666291000000
H	5.549551000000	–2.934107000000	3.238595000000
H	6.037310000000	–1.691515000000	2.065919000000
C	–2.948839000000	3.415677000000	3.219847000000
C	–2.856627000000	3.743428000000	4.723575000000
C	–3.960631000000	2.269661000000	2.997901000000
C	–3.460555000000	4.663255000000	2.483313000000
C	–1.581395000000	2.954538000000	2.695587000000
C	–0.912318000000	1.892890000000	3.324253000000
C	0.300142000000	1.385902000000	2.851008000000
C	0.867788000000	1.986330000000	1.714753000000
C	0.228954000000	3.031854000000	1.037914000000
C	–0.985613000000	3.504168000000	1.557193000000
C	0.736341000000	3.577452000000	–0.292063000000
O	2.093930000000	1.503501000000	1.254942000000
C	3.188863000000	2.252865000000	1.756677000000
H	–3.835247000000	4.077602000000	5.102032000000
H	–2.126787000000	4.547159000000	4.906663000000
H	–2.553959000000	2.869974000000	5.319976000000
H	–4.959684000000	2.558210000000	3.361178000000
H	–3.659649000000	1.355075000000	3.530872000000
H	–4.042665000000	2.022718000000	1.927557000000
H	–2.764806000000	5.510276000000	2.584630000000
H	–4.428553000000	4.973234000000	2.904404000000
H	–3.617748000000	4.471196000000	1.410989000000
H	–1.353634000000	1.433135000000	4.209314000000
H	–1.477557000000	4.320755000000	1.031824000000
H	1.809526000000	3.394251000000	–0.404105000000
H	0.567518000000	4.664105000000	–0.316219000000
H	3.045995000000	2.452523000000	2.836767000000
H	3.266739000000	3.226348000000	1.249931000000
C	4.496643000000	1.498555000000	1.623871000000
O	4.523928000000	0.297594000000	1.457191000000
C	5.735773000000	2.344067000000	1.739696000000
H	6.611304000000	1.715635000000	1.946550000000
H	5.629074000000	3.121839000000	2.512240000000
H	5.867253000000	2.857748000000	0.773206000000

C	–3.552281000000	3.115484000000	–2.952779000000
C	–3.550518000000	4.579677000000	–3.436677000000
C	–4.342610000000	3.005810000000	–1.629835000000
C	–4.258711000000	2.252866000000	–4.009701000000
C	–2.110772000000	2.652670000000	–2.698035000000
C	–1.310517000000	3.344294000000	–1.775668000000
C	–0.016458000000	2.930494000000	–1.445101000000
C	0.503525000000	1.812092000000	–2.114526000000
C	–0.266933000000	1.065128000000	–3.013991000000
C	–1.565450000000	1.511507000000	–3.296535000000
C	0.223388000000	–0.247773000000	–3.608988000000
O	1.806848000000	1.417970000000	–1.820858000000
C	2.786327000000	1.773893000000	–2.772266000000
H	–4.580844000000	4.925068000000	–3.615459000000
H	–2.987581000000	4.681799000000	–4.377358000000
H	–3.097015000000	5.256416000000	–2.697210000000
H	–5.386357000000	3.325298000000	–1.776711000000
H	–3.907217000000	3.636532000000	–0.840330000000
H	–4.350785000000	1.967041000000	–1.263225000000
H	–3.737992000000	2.286566000000	–4.979002000000
H	–5.282119000000	2.623832000000	–4.169176000000
H	–4.336150000000	1.200568000000	–3.694688000000
H	–1.715112000000	4.220112000000	–1.266829000000
H	–2.162244000000	0.929342000000	–3.996703000000
H	1.317428000000	–0.274759000000	–3.609480000000
H	–0.102986000000	–0.300640000000	–4.658111000000
H	2.400862000000	2.562546000000	–3.444620000000
H	3.051190000000	0.913956000000	–3.411499000000
C	4.051403000000	2.323057000000	–2.128197000000
O	4.055604000000	2.768874000000	–1.002847000000
C	5.280739000000	2.289757000000	–2.999224000000
H	6.016160000000	3.023560000000	–2.645642000000
H	5.043368000000	2.465809000000	–4.059905000000
H	5.721276000000	1.281576000000	–2.920423000000
O	–0.158411000000	–0.070412000000	0.035053000000
H	–0.086200000000	–0.898907000000	0.528714000000
C	–1.544114000000	0.243443000000	0.029830000000
H	–1.674038000000	1.224784000000	–0.432156000000
H	–1.939150000000	0.287002000000	1.057791000000
H	–2.113252000000	–0.501635000000	–0.550872000000

NaL$_e$MeOH$^+$, zero imaginary frequencies
143
–3055.33099501

Na	2.376844000000	0.043056000000	–0.024724000000
C	–3.405292000000	–3.229647000000	–3.017688000000
C	–3.492011000000	–3.483564000000	–4.536283000000
C	–4.352446000000	–2.072590000000	–2.628942000000
C	–3.866805000000	–4.500658000000	–2.288337000000
C	–1.972363000000	–2.830594000000	–2.635902000000
C	–1.361285000000	–1.739477000000	–3.273651000000
C	–0.079737000000	–1.297316000000	–2.940631000000
C	0.616711000000	–1.999380000000	–1.942081000000
C	0.030349000000	–3.055034000000	–1.232459000000
C	–1.255202000000	–3.466871000000	–1.617962000000
C	0.678454000000	–3.641248000000	0.020418000000
O	1.894799000000	–1.576611000000	–1.603437000000
C	2.942176000000	–2.242476000000	–2.269687000000
H	–4.516918000000	–3.772257000000	–4.817162000000
H	–2.813078000000	–4.295991000000	–4.838357000000
H	–3.229167000000	–2.589154000000	–5.120306000000
H	–5.392876000000	–2.319749000000	–2.892900000000
H	–4.086805000000	–1.138068000000	–3.146208000000
H	–4.310670000000	–1.879509000000	–1.545427000000
H	–3.215186000000	–5.359121000000	–2.512504000000
H	–4.886579000000	–4.763296000000	–2.606879000000
H	–3.892120000000	–4.361510000000	–1.196831000000
H	–1.906551000000	–1.201188000000	–4.049704000000
H	–1.704146000000	–4.303347000000	–1.086001000000
H	1.759348000000	–3.465903000000	0.017045000000
H	0.503681000000	–4.727227000000	0.041156000000
H	2.744932000000	–2.297561000000	–3.358689000000
H	3.065838000000	–3.277158000000	–1.906449000000
C	4.241490000000	–1.487821000000	–2.075286000000
O	4.253478000000	–0.373536000000	–1.586365000000
C	5.489116000000	–2.201861000000	–2.508829000000
H	6.298195000000	–1.484291000000	–2.696514000000
H	5.322202000000	–2.840044000000	–3.389611000000
H	5.779475000000	–2.855062000000	–1.668975000000
C	–3.383208000000	–3.071102000000	2.989213000000
C	–3.478698000000	–4.581499000000	3.284394000000
C	–4.249282000000	–2.725121000000	1.757430000000
C	–3.932405000000	–2.303303000000	4.201258000000
C	–1.929374000000	–2.691537000000	2.674961000000
C	–1.242924000000	–3.352987000000	1.648108000000
C	0.062012000000	–3.015958000000	1.270340000000
C	0.708107000000	–1.990592000000	1.990665000000
C	0.036343000000	–1.252480000000	2.981101000000
C	–1.265850000000	–1.642217000000	3.319938000000
C	0.614302000000	0.021764000000	3.587284000000
O	1.997962000000	–1.639220000000	1.621345000000
C	3.044303000000	–2.131090000000	2.430188000000
H	–4.519974000000	–4.860610000000	3.508541000000
H	–2.857914000000	–4.853128000000	4.152170000000
H	–3.150208000000	–5.191330000000	2.429695000000
H	–5.304935000000	–2.976749000000	1.945190000000
H	–3.924515000000	–3.279308000000	0.864228000000
H	–4.188305000000	–1.650524000000	1.524340000000
H	–3.337798000000	–2.492685000000	5.108140000000
H	–4.965028000000	–2.622192000000	4.406557000000
H	–3.955875000000	–1.217532000000	4.022818000000
H	–1.744910000000	–4.149730000000	1.100110000000
H	–1.778415000000	–1.079669000000	4.097184000000
H	1.698897000000	0.047410000000	3.444048000000
H	0.418494000000	0.018293000000	4.670115000000
H	2.722837000000	–3.037352000000	2.977472000000
H	3.350630000000	–1.383941000000	3.181663000000
C	4.250519000000	–2.520452000000	1.587849000000
O	4.144313000000	–2.753966000000	0.402892000000
C	5.560236000000	–2.590343000000	2.323458000000
H	6.274922000000	–3.217911000000	1.776038000000
H	5.439097000000	–2.953269000000	3.355819000000
H	5.950778000000	–1.559751000000	2.373992000000
C	–3.406181000000	3.082743000000	3.118926000000
C	–3.483187000000	3.313419000000	4.641408000000
C	–4.320826000000	1.901733000000	2.724864000000
C	–3.917126000000	4.348099000000	2.412710000000
C	–1.967840000000	2.732522000000	2.710115000000
C	–1.300290000000	1.672985000000	3.344399000000
C	–0.015877000000	1.268907000000	2.973663000000
C	0.625340000000	1.981621000000	1.946490000000
C	–0.016676000000	3.008918000000	1.245077000000
C	–1.303461000000	3.378708000000	1.663403000000
C	0.578728000000	3.624073000000	–0.018288000000
O	1.909518000000	1.599569000000	1.585287000000
C	2.950844000000	2.302843000000	2.219229000000
H	–4.512704000000	3.568966000000	4.937120000000
H	–2.824379000000	4.140775000000	4.947435000000
H	–3.189526000000	2.419427000000	5.211280000000
H	–5.366152000000	2.111199000000	3.002077000000
H	–4.018777000000	0.970734000000	3.228170000000
H	–4.282637000000	1.723178000000	1.638810000000
H	–3.283759000000	5.221138000000	2.632723000000
H	–4.936999000000	4.579331000000	2.754577000000
H	–3.961009000000	4.219435000000	1.320645000000
H	–1.801764000000	1.131789000000	4.147348000000
H	–1.798817000000	4.187341000000	1.129469000000
H	1.662841000000	3.472218000000	–0.044396000000
H	0.381236000000	4.706555000000	–0.014590000000
H	2.776702000000	2.369714000000	3.311782000000
H	3.039396000000	3.334704000000	1.838145000000
C	4.264607000000	1.578275000000	2.006816000000
O	4.293477000000	0.453738000000	1.541834000000
C	5.503805000000	2.333741000000	2.391171000000
H	6.335733000000	1.642110000000	2.576473000000
H	5.342160000000	2.990473000000	3.259187000000
H	5.756171000000	2.971657000000	1.527607000000

C	–3.489496000000	3.111208000000	–2.996323000000
C	–3.547905000000	4.612153000000	–3.344152000000
C	–4.365080000000	2.830541000000	–1.754713000000
C	–4.061174000000	2.315068000000	–4.179436000000
C	–2.044493000000	2.706574000000	–2.670777000000
C	–1.346287000000	3.373940000000	–1.653244000000
C	–0.055920000000	3.006040000000	–1.259200000000
C	0.570721000000	1.964331000000	–1.963967000000
C	–0.104512000000	1.230051000000	–2.948137000000
C	–1.402789000000	1.630650000000	–3.293046000000
C	0.487997000000	–0.030457000000	–3.568485000000
O	1.880271000000	1.638152000000	–1.635096000000
C	2.878529000000	2.171413000000	–2.474548000000
H	–4.582331000000	4.911920000000	–3.574282000000
H	–2.923423000000	4.836234000000	–4.222804000000
H	–3.197984000000	5.241616000000	–2.512499000000
H	–5.413174000000	3.107488000000	–1.949879000000
H	–4.024116000000	3.402317000000	–0.878678000000
H	–4.336774000000	1.761923000000	–1.489157000000
H	–3.466903000000	2.460680000000	–5.094562000000
H	–5.087853000000	2.648837000000	–4.391634000000
H	–4.105764000000	1.236292000000	–3.963960000000
H	–1.831388000000	4.193744000000	–1.122314000000
H	–1.921485000000	1.067252000000	–4.066445000000
H	1.577023000000	–0.024180000000	–3.459579000000
H	0.259725000000	–0.035992000000	–4.644929000000
H	2.512228000000	3.077889000000	–2.993158000000
H	3.174302000000	1.448896000000	–3.255282000000
C	4.110007000000	2.581730000000	–1.682015000000
O	4.058885000000	2.777753000000	–0.486554000000
C	5.377271000000	2.724275000000	–2.480450000000
H	6.090373000000	3.372015000000	–1.954765000000
H	5.189382000000	3.102054000000	–3.497266000000
H	5.812000000000	1.714491000000	–2.572501000000
O	–0.333088000000	–0.063015000000	0.037894000000
H	–0.254134000000	–0.952805000000	0.403454000000
C	–1.722746000000	0.206531000000	–0.030757000000
H	–1.851650000000	1.242815000000	–0.355306000000
H	–2.200049000000	0.091477000000	0.956824000000
H	–2.224623000000	–0.454895000000	–0.756952000000

K**L**$_{c}$MeOH$'^{+}$, zero imaginary frequencies
143
–3492.92817817

K	2.870680000000	0.022034000000	–0.026217000000
C	–3.520784000000	–3.468608000000	–2.510886000000
C	–3.639457000000	–3.920170000000	–3.980619000000
C	–4.423935000000	–2.238267000000	–2.271731000000
C	–4.013064000000	–4.614267000000	–1.613144000000
C	–2.068710000000	–3.074279000000	–2.202636000000
C	–1.428883000000	–2.105019000000	–2.990193000000
C	–0.120635000000	–1.680257000000	–2.747786000000
C	0.577062000000	–2.291731000000	–1.689659000000
C	–0.050189000000	–3.185548000000	–0.808891000000
C	–1.361709000000	–3.585457000000	–1.110224000000
C	0.587284000000	–3.603798000000	0.518200000000
O	1.901841000000	–1.937076000000	–1.482272000000
C	2.867692000000	–2.828645000000	–1.985976000000
H	–4.677765000000	–4.206980000000	–4.209534000000
H	–2.993119000000	–4.789365000000	–4.178229000000
H	–3.355068000000	–3.121780000000	–4.681920000000
H	–5.475329000000	–2.481967000000	–2.492007000000
H	–4.133551000000	–1.390754000000	–2.910686000000
H	–4.362240000000	–1.903440000000	–1.224477000000
H	–3.391584000000	–5.515996000000	–1.725036000000
H	–5.044721000000	–4.883372000000	–1.884645000000
H	–4.020286000000	–4.329176000000	–0.550176000000
H	–1.972719000000	–1.652199000000	–3.818973000000
H	–1.839188000000	–4.311228000000	–0.455466000000
H	1.669224000000	–3.436638000000	0.493602000000
H	0.410263000000	–4.677537000000	0.682620000000
H	2.532449000000	–3.283173000000	–2.939000000000
H	3.061700000000	–3.658779000000	–1.282095000000
C	4.177302000000	–2.105585000000	–2.266464000000
O	4.239023000000	–0.891696000000	–2.308985000000
C	5.380442000000	–2.988127000000	–2.446281000000
H	6.163907000000	–2.459022000000	–3.003738000000
H	5.132597000000	–3.943781000000	–2.932495000000
H	5.756343000000	–3.214494000000	–1.433122000000
C	–3.471474000000	–2.564313000000	3.370790000000
C	–3.619138000000	–4.038089000000	3.798598000000
C	–4.333026000000	–2.297901000000	2.116146000000
C	–3.984623000000	–1.675485000000	4.514317000000
C	–2.008288000000	–2.263203000000	3.016453000000
C	–1.338406000000	–3.063600000000	2.081549000000
C	–0.021431000000	–2.814960000000	1.679761000000
C	0.652782000000	–1.733127000000	2.286348000000
C	–0.011450000000	–0.837113000000	3.145435000000
C	–1.325229000000	–1.150952000000	3.519567000000
C	0.587378000000	0.507875000000	3.557129000000
O	1.972248000000	–1.504430000000	1.932127000000
C	2.959046000000	–1.961195000000	2.827399000000
H	–4.667418000000	–4.256077000000	4.055244000000
H	–2.998891000000	–4.256408000000	4.681728000000
H	–3.325441000000	–4.733698000000	2.998574000000
H	–5.395682000000	–2.495629000000	2.327833000000
H	–4.030618000000	–2.939939000000	1.275531000000
H	–4.236069000000	–1.251395000000	1.788166000000
H	–3.380360000000	–1.795717000000	5.426657000000
H	–5.021432000000	–1.948448000000	4.760722000000
H	–3.986571000000	–0.610349000000	4.238435000000
H	–1.862794000000	–3.905524000000	1.632008000000
H	–1.831809000000	–0.480873000000	4.209833000000
H	1.667818000000	0.512371000000	3.379321000000
H	0.418002000000	0.656600000000	4.634698000000
H	2.664172000000	–2.923690000000	3.289648000000
H	3.125537000000	–1.240445000000	3.648269000000
C	4.273820000000	–2.189825000000	2.093975000000
O	4.320321000000	–2.246714000000	0.880190000000
C	5.493860000000	–2.296662000000	2.963879000000
H	6.301623000000	–2.813589000000	2.430304000000
H	5.282853000000	–2.788830000000	3.925418000000
H	5.813322000000	–1.261949000000	3.179307000000
C	–3.506591000000	3.366978000000	2.622889000000
C	–3.623868000000	3.775896000000	4.104704000000
C	–4.386698000000	2.125881000000	2.356013000000
C	–4.028782000000	4.526781000000	1.760513000000
C	–2.051721000000	3.006109000000	2.287416000000
C	–1.365937000000	2.064755000000	3.069699000000
C	–0.058764000000	1.659711000000	2.784783000000
C	0.590671000000	2.270145000000	1.696260000000
C	–0.081837000000	3.142027000000	0.827981000000
C	–1.390917000000	3.514320000000	1.165127000000
C	0.507936000000	3.580762000000	–0.512315000000
O	1.916293000000	1.940552000000	1.459557000000
C	2.878490000000	2.854850000000	1.925482000000
H	–4.664960000000	4.041541000000	4.345946000000
H	–2.988989000000	4.648413000000	4.323533000000
H	–3.326566000000	2.963169000000	4.783910000000
H	–5.442056000000	2.340804000000	2.587533000000
H	–4.073627000000	1.269955000000	2.972516000000
H	–4.321994000000	1.819445000000	1.300408000000
H	–3.414356000000	5.432211000000	1.881019000000
H	–5.058209000000	4.777531000000	2.057088000000
H	–4.053211000000	4.265801000000	0.691779000000
H	–1.870874000000	1.620099000000	3.926970000000
H	–1.906796000000	4.213149000000	0.510165000000
H	1.591959000000	3.426348000000	–0.518241000000
H	0.314541000000	4.655171000000	–0.654051000000
H	2.563441000000	3.315759000000	2.882624000000
H	3.037946000000	3.679185000000	1.206475000000
C	4.207158000000	2.156499000000	2.175507000000
O	4.286901000000	0.943974000000	2.238365000000
C	5.403345000000	3.057664000000	2.296549000000
H	6.215156000000	2.548840000000	2.831845000000
H	5.159791000000	4.017515000000	2.776641000000
H	5.735726000000	3.272945000000	1.265879000000

C	–3.562031000000	2.604709000000	–3.381599000000
C	–3.671167000000	4.065885000000	–3.860951000000
C	–4.436383000000	2.403689000000	–2.123685000000
C	–4.097526000000	1.689849000000	–4.493980000000
C	–2.106756000000	2.278953000000	–3.014174000000
C	–1.425525000000	3.081096000000	–2.086142000000
C	–0.121250000000	2.802025000000	–1.666032000000
C	0.533404000000	1.706263000000	–2.256614000000
C	–0.134403000000	0.819684000000	–3.113607000000
C	–1.443589000000	1.145616000000	–3.496367000000
C	0.475468000000	–0.515877000000	–3.536145000000
O	1.864745000000	1.486306000000	–1.935689000000
C	2.806748000000	1.982285000000	–2.855129000000
H	–4.713157000000	4.304721000000	–4.125562000000
H	–3.045049000000	4.235329000000	–4.750643000000
H	–3.355886000000	4.779876000000	–3.085719000000
H	–5.491752000000	2.629114000000	–2.344869000000
H	–4.117403000000	3.059520000000	–1.299751000000
H	–4.375200000000	1.363649000000	–1.766510000000
H	–3.492661000000	1.765868000000	–5.410623000000
H	–5.128796000000	1.976888000000	–4.748269000000
H	–4.120855000000	0.634588000000	–4.181972000000
H	–1.933696000000	3.943207000000	–1.653710000000
H	–1.955728000000	0.477906000000	–4.185901000000
H	1.560227000000	–0.498345000000	–3.388120000000
H	0.280214000000	–0.673655000000	–4.608141000000
H	2.470313000000	2.942039000000	–3.295330000000
H	2.964656000000	1.280627000000	–3.694985000000
C	4.142468000000	2.244637000000	–2.175694000000
O	4.244233000000	2.289686000000	–0.964109000000
C	5.319523000000	2.404653000000	–3.096336000000
H	6.129155000000	2.948328000000	–2.592934000000
H	5.049097000000	2.895375000000	–4.043534000000
H	5.669812000000	1.385098000000	–3.334710000000
O	–0.328162000000	–0.023145000000	0.006075000000
H	–0.161730000000	–0.795035000000	0.559892000000
C	–1.733675000000	0.143219000000	–0.025221000000
H	–1.951316000000	1.058632000000	–0.583984000000
H	–2.150358000000	0.250581000000	0.989882000000
H	–2.230583000000	–0.701775000000	–0.530727000000

Rb**L**$_{c}$MeOH$^{\prime+}$, zero imaginary frequencies
143
–2917.15986034

Rb	–3.100190000000	–0.055520000000	0.010661000000
C	3.573721000000	2.656290000000	–3.263209000000
C	3.744716000000	2.655326000000	–4.795257000000
C	4.431105000000	1.530122000000	–2.643942000000
C	4.071997000000	4.006208000000	–2.723593000000
C	2.107187000000	2.392911000000	–2.893351000000
C	1.432855000000	1.293904000000	–3.444555000000
C	0.113602000000	0.971376000000	–3.114466000000
C	–0.563461000000	1.832998000000	–2.223148000000
C	0.105386000000	2.877237000000	–1.555514000000
C	1.426732000000	3.152022000000	–1.937577000000
C	–0.496998000000	3.602957000000	–0.351536000000
O	–1.903582000000	1.592070000000	–1.962699000000
C	–2.812151000000	2.412988000000	–2.661777000000
H	4.794928000000	2.853481000000	–5.059605000000
H	3.122185000000	3.434057000000	–5.262559000000
H	3.471497000000	1.688779000000	–5.243748000000
H	5.496409000000	1.679793000000	–2.880660000000
H	4.134596000000	0.542644000000	–3.027565000000
H	4.320677000000	1.509665000000	–1.548924000000
H	3.461836000000	4.843444000000	–3.096132000000
H	5.109505000000	4.176211000000	–3.047493000000
H	4.067572000000	4.037581000000	–1.623912000000
H	1.958729000000	0.651039000000	–4.148245000000
H	1.936329000000	3.979628000000	–1.450762000000
H	–1.576003000000	3.422924000000	–0.303413000000
H	–0.332168000000	4.685439000000	–0.464803000000
H	–2.407786000000	2.695452000000	–3.652634000000
H	–3.012668000000	3.351953000000	–2.112547000000
C	–4.133799000000	1.697559000000	–2.893900000000
O	–4.274602000000	0.516955000000	–2.642122000000
C	–5.250370000000	2.560470000000	–3.419674000000
H	–5.998402000000	1.942861000000	–3.933484000000
H	–4.885489000000	3.358060000000	–4.084392000000
H	–5.732720000000	3.046226000000	–2.553507000000
C	3.608517000000	3.378372000000	2.635596000000
C	3.696459000000	4.914376000000	2.736226000000
C	4.492591000000	2.883275000000	1.469958000000
C	4.148801000000	2.777196000000	3.942496000000
C	2.158880000000	2.952477000000	2.358926000000
C	1.464613000000	3.498510000000	1.269228000000
C	0.156522000000	3.122889000000	0.945914000000
C	–0.484083000000	2.197767000000	1.790701000000
C	0.200555000000	1.547166000000	2.827379000000
C	1.509324000000	1.964795000000	3.106012000000
C	–0.387654000000	0.343381000000	3.559823000000
O	–1.822946000000	1.914052000000	1.558769000000
C	–2.731382000000	2.636728000000	2.356872000000
H	4.733449000000	5.225373000000	2.937832000000
H	3.061467000000	5.291882000000	3.552664000000
H	3.379407000000	5.408764000000	1.805961000000
H	5.543282000000	3.173414000000	1.629208000000
H	4.167853000000	3.307972000000	0.508484000000
H	4.448856000000	1.786312000000	1.383190000000
H	3.537439000000	3.071068000000	4.809498000000
H	5.174797000000	3.132813000000	4.119728000000
H	4.187512000000	1.678062000000	3.903619000000
H	1.960797000000	4.239354000000	0.642672000000
H	2.032289000000	1.487135000000	3.931471000000
H	–1.469648000000	0.299347000000	3.398001000000
H	–0.205173000000	0.454846000000	4.639974000000
H	–2.296886000000	3.602364000000	2.678742000000
H	–2.990778000000	2.078837000000	3.277050000000
C	–4.018126000000	2.945895000000	1.608314000000
O	–4.145704000000	2.707224000000	0.422270000000
C	–5.126968000000	3.528887000000	2.444930000000
H	–5.822074000000	4.096104000000	1.812257000000
H	–4.747157000000	4.159383000000	3.262848000000
H	–5.678861000000	2.689814000000	2.903560000000
C	3.681172000000	–2.684246000000	3.281464000000
C	3.801314000000	–2.770446000000	4.816150000000
C	4.561882000000	–1.528620000000	2.756028000000
C	4.200160000000	–4.000371000000	2.681912000000
C	2.224599000000	–2.401872000000	2.884457000000
C	1.558128000000	–1.290783000000	3.423370000000
C	0.245805000000	–0.959131000000	3.074681000000
C	–0.426398000000	–1.811409000000	2.180454000000
C	0.231557000000	–2.862299000000	1.525890000000
C	1.543962000000	–3.158358000000	1.924726000000
C	–0.389734000000	–3.583817000000	0.331891000000
O	–1.775939000000	–1.587192000000	1.941756000000
C	–2.645645000000	–2.374560000000	2.720212000000
H	4.843841000000	–2.973153000000	5.107517000000
H	3.171151000000	–3.580685000000	5.214601000000
H	3.497409000000	–1.834239000000	5.307419000000
H	5.617392000000	–1.692283000000	3.025684000000
H	4.252319000000	–0.560074000000	3.176718000000
H	4.496042000000	–1.452991000000	1.659261000000
H	3.590613000000	–4.860587000000	2.998666000000
H	5.233003000000	–4.180390000000	3.015741000000
H	4.213691000000	–3.973276000000	1.581766000000
H	2.081630000000	–0.651393000000	4.134024000000
H	2.044634000000	–4.000279000000	1.451460000000
H	–1.472585000000	–3.421731000000	0.318511000000
H	–0.204343000000	–4.665244000000	0.425068000000
H	–2.179181000000	–2.644769000000	3.687089000000
H	–2.895787000000	–3.325586000000	2.211107000000
C	–3.941891000000	–1.646052000000	3.035581000000
O	–4.108878000000	–0.474504000000	2.753349000000
C	–5.010264000000	–2.484456000000	3.688193000000
H	–5.699074000000	–1.845713000000	4.256331000000
H	–4.590472000000	–3.269911000000	4.334356000000
H	–5.581543000000	–2.985114000000	2.887031000000

C	3.631735000000	–3.223824000000	–2.739715000000
C	3.764039000000	–4.756024000000	–2.852602000000
C	4.516992000000	–2.711270000000	–1.582033000000
C	4.134184000000	–2.598880000000	–4.050326000000
C	2.173465000000	–2.847111000000	–2.439774000000
C	1.528679000000	–3.398237000000	–1.322819000000
C	0.211819000000	–3.082222000000	–0.980528000000
C	–0.486317000000	–2.200777000000	–1.825975000000
C	0.147417000000	–1.532979000000	–2.883751000000
C	1.465312000000	–1.904793000000	–3.191520000000
C	–0.500819000000	–0.344203000000	–3.596009000000
O	–1.835302000000	–1.974932000000	–1.582224000000
C	–2.711051000000	–2.789173000000	–2.326270000000
H	4.807717000000	–5.035355000000	–3.066143000000
H	3.132303000000	–5.146112000000	–3.665696000000
H	3.469206000000	–5.264718000000	–1.922835000000
H	5.574033000000	–2.963849000000	–1.761736000000
H	4.223126000000	–3.156477000000	–0.619794000000
H	4.437490000000	–1.617761000000	–1.480747000000
H	3.524872000000	–2.913183000000	–4.911613000000
H	5.170724000000	–2.914852000000	–4.240783000000
H	4.131658000000	–1.499162000000	–4.008729000000
H	2.073922000000	–4.095364000000	–0.687130000000
H	1.947176000000	–1.428819000000	–4.042439000000
H	–1.579893000000	–0.330956000000	–3.407873000000
H	–0.342303000000	–0.438571000000	–4.681431000000
H	–2.229911000000	–3.749806000000	–2.592014000000
H	–3.000380000000	–2.306020000000	–3.280296000000
C	–3.976972000000	–3.118438000000	–1.550618000000
O	–4.116974000000	–2.804559000000	–0.384169000000
C	–5.049861000000	–3.828015000000	–2.335956000000
H	–5.694387000000	–4.404455000000	–1.659435000000
H	–4.635286000000	–4.478306000000	–3.120722000000
H	–5.669291000000	–3.061435000000	–2.833488000000
O	0.372372000000	–0.012620000000	–0.009382000000
H	0.143127000000	0.669156000000	–0.652582000000
C	1.786317000000	–0.062031000000	0.028174000000
H	2.076373000000	–0.855819000000	0.723643000000
H	2.216679000000	0.888941000000	0.383885000000
H	2.209230000000	–0.297546000000	–0.962572000000

Cs$\mathbf{L}_{c}$MeOH$'^{+}$, zero imaginary frequencies
143
–2913.19886577

Cs	–3.374694000000	–0.032843000000	0.046101000000
C	3.676244000000	2.285525000000	–3.538381000000
C	3.831312000000	2.125618000000	–5.063815000000
C	4.539216000000	1.227833000000	–2.815196000000
C	4.181978000000	3.682934000000	–3.147112000000
C	2.212996000000	2.065977000000	–3.129037000000
C	1.530527000000	0.918352000000	–3.556757000000
C	0.211684000000	0.638873000000	–3.187381000000
C	–0.456553000000	1.591370000000	–2.387291000000
C	0.221308000000	2.696392000000	–1.837696000000
C	1.541685000000	2.923168000000	–2.253378000000
C	–0.372591000000	3.549812000000	–0.717544000000
O	–1.805740000000	1.406287000000	–2.120161000000
C	–2.678132000000	2.168335000000	–2.926914000000
H	4.879034000000	2.292723000000	–5.357993000000
H	3.205129000000	2.853415000000	–5.602879000000
H	3.552057000000	1.118548000000	–5.407077000000
H	5.601775000000	1.349025000000	–3.079014000000
H	4.236563000000	0.206539000000	–3.090072000000
H	4.442029000000	1.322024000000	–1.722908000000
H	3.569618000000	4.478387000000	–3.599157000000
H	5.216623000000	3.815514000000	–3.496691000000
H	4.188017000000	3.828867000000	–2.056729000000
H	2.048059000000	0.203230000000	–4.193610000000
H	2.056665000000	3.795567000000	–1.859475000000
H	–1.452331000000	3.383752000000	–0.645129000000
H	–0.200464000000	4.613162000000	–0.944552000000
H	–2.170864000000	2.503717000000	–3.849928000000
H	–3.016327000000	3.077931000000	–2.393807000000
C	–3.912244000000	1.377571000000	–3.332645000000
O	–4.065745000000	0.215244000000	–3.011930000000
C	–4.948293000000	2.150868000000	–4.108756000000
H	–5.571990000000	1.462175000000	–4.693519000000
H	–4.499294000000	2.913309000000	–4.762627000000
H	–5.596417000000	2.676818000000	–3.386107000000
C	3.751461000000	3.625931000000	2.248503000000
C	3.840085000000	5.164009000000	2.186993000000
C	4.630060000000	3.011053000000	1.137131000000
C	4.298557000000	3.165019000000	3.608488000000
C	2.300214000000	3.174538000000	2.026041000000
C	1.599010000000	3.604573000000	0.890213000000
C	0.287308000000	3.201991000000	0.617162000000
C	–0.349863000000	2.372254000000	1.557513000000
C	0.341755000000	1.832409000000	2.651639000000
C	1.653560000000	2.272200000000	2.876117000000
C	–0.243611000000	0.715532000000	3.511274000000
O	–1.698643000000	2.087214000000	1.382639000000
C	–2.569543000000	2.886243000000	2.152433000000
H	4.878084000000	5.493791000000	2.350031000000
H	3.209024000000	5.625408000000	2.962367000000
H	3.518732000000	5.558408000000	1.211506000000
H	5.681045000000	3.317859000000	1.258937000000
H	4.299391000000	3.330767000000	0.137801000000
H	4.587945000000	1.911091000000	1.167476000000
H	3.691864000000	3.548823000000	4.443028000000
H	5.325626000000	3.536342000000	3.742032000000
H	4.336515000000	2.067860000000	3.685028000000
H	2.091116000000	4.275766000000	0.186748000000
H	2.180321000000	1.881850000000	3.743902000000
H	–1.327396000000	0.657836000000	3.369464000000
H	–0.046220000000	0.936278000000	4.571868000000
H	–2.050444000000	3.783700000000	2.535388000000
H	–2.939791000000	2.328273000000	3.034819000000
C	–3.780633000000	3.352239000000	1.360296000000
O	–3.933526000000	3.072524000000	0.186341000000
C	–4.804656000000	4.132003000000	2.146594000000
H	–5.393749000000	4.766801000000	1.471756000000
H	–4.349523000000	4.736712000000	2.944897000000
H	–5.488647000000	3.411421000000	2.628137000000
C	3.815362000000	–2.333824000000	3.517825000000
C	3.941864000000	–2.260883000000	5.052808000000
C	4.698634000000	–1.242838000000	2.872543000000
C	4.327100000000	–3.707090000000	3.056094000000
C	2.357728000000	–2.089029000000	3.100159000000
C	1.697010000000	–0.926138000000	3.523188000000
C	0.381849000000	–0.630070000000	3.153390000000
C	–0.299400000000	–1.568276000000	2.358337000000
C	0.352078000000	–2.684461000000	1.815059000000
C	1.667368000000	–2.940266000000	2.231429000000
C	–0.279421000000	–3.528925000000	0.711378000000
O	–1.656590000000	–1.379437000000	2.121503000000
C	–2.497692000000	–2.126551000000	2.971089000000
H	4.984746000000	–2.437346000000	5.359738000000
H	3.309752000000	–3.022338000000	5.535515000000
H	3.644150000000	–1.277236000000	5.445326000000
H	5.754031000000	–1.380322000000	3.156949000000
H	4.392354000000	–0.234320000000	3.188671000000
H	4.631931000000	–1.283104000000	1.774027000000
H	3.716857000000	–4.527486000000	3.464042000000
H	5.361365000000	–3.855057000000	3.401164000000
H	4.334017000000	–3.794702000000	1.959058000000
H	2.225960000000	–0.217765000000	4.160513000000
H	2.161161000000	–3.829962000000	1.846636000000
H	–1.362857000000	–3.374101000000	0.688284000000
H	–0.088020000000	–4.594058000000	0.914975000000
H	–1.948169000000	–2.480368000000	3.862523000000
H	–2.882573000000	–3.029037000000	2.455869000000
C	–3.696511000000	–1.326775000000	3.454970000000
O	–3.869302000000	–0.163772000000	3.142920000000
C	–4.683995000000	–2.092334000000	4.300068000000
H	–5.262851000000	–1.398358000000	4.923451000000
H	–4.197508000000	–2.856415000000	4.924343000000
H	–5.382651000000	–2.615306000000	3.623843000000

C	3.715843000000	–3.504807000000	–2.411072000000
C	3.837870000000	–5.041450000000	–2.366530000000
C	4.615494000000	–2.882200000000	–1.320533000000
C	4.209501000000	–3.021510000000	–3.783436000000
C	2.262518000000	–3.089752000000	–2.139141000000
C	1.625110000000	–3.517038000000	–0.965664000000
C	0.311273000000	–3.163831000000	–0.648812000000
C	–0.392086000000	–2.373305000000	–1.575010000000
C	0.235893000000	–1.823206000000	–2.701825000000
C	1.550076000000	–2.229773000000	–2.979798000000
C	–0.412246000000	–0.713948000000	–3.530976000000
O	–1.745900000000	–2.136749000000	–1.359119000000
C	–2.601155000000	–3.006980000000	–2.065443000000
H	4.877869000000	–5.348682000000	–2.558824000000
H	3.196404000000	–5.508722000000	–3.129808000000
H	3.548114000000	–5.449403000000	–1.386804000000
H	5.668969000000	–3.160620000000	–1.482165000000
H	4.327508000000	–3.222805000000	–0.314834000000
H	4.544697000000	–1.783581000000	–1.332404000000
H	3.591069000000	–3.419556000000	–4.602577000000
H	5.242824000000	–3.361203000000	–3.949024000000
H	4.212215000000	–1.923363000000	–3.855425000000
H	2.172714000000	–4.146437000000	–0.264830000000
H	2.024966000000	–1.848282000000	–3.880740000000
H	–1.490341000000	–0.674357000000	–3.342676000000
H	–0.257504000000	–0.920061000000	–4.601443000000
H	–2.061203000000	–3.915441000000	–2.390033000000
H	–2.990130000000	–2.525166000000	–2.984771000000
C	–3.792641000000	–3.453977000000	–1.233138000000
O	–3.939341000000	–3.110606000000	–0.075834000000
C	–4.802327000000	–4.308247000000	–1.958479000000
H	–5.359872000000	–4.922844000000	–1.239540000000
H	–4.339898000000	–4.942089000000	–2.729540000000
H	–5.517647000000	–3.638188000000	–2.466651000000
O	0.565855000000	–0.030311000000	0.002449000000
H	0.298963000000	0.579262000000	–0.695983000000
C	1.978740000000	–0.054880000000	0.005840000000
H	2.297950000000	–0.779434000000	0.762667000000
H	2.405063000000	0.929412000000	0.263197000000
H	2.383375000000	–0.373197000000	–0.969547000000

Mg**L**$_{c}$MeOH'$^{2+}$, zero imaginary frequencies

143

–3092.89408188

Mg	2.208671000000	0.067249000000	–0.032246000000
C	–3.366373000000	–2.800062000000	–3.586601000000
C	–3.371425000000	–2.871372000000	–5.127569000000
C	–4.340918000000	–1.699556000000	–3.110215000000
C	–3.848254000000	–4.149789000000	–3.033579000000
C	–1.958807000000	–2.441917000000	–3.090413000000
C	–1.341457000000	–1.262526000000	–3.540809000000
C	–0.085069000000	–0.853738000000	–3.088121000000
C	0.568269000000	–1.684374000000	–2.166975000000
C	–0.003078000000	–2.855007000000	–1.663780000000
C	–1.266439000000	–3.219145000000	–2.154517000000
C	0.637493000000	–3.635441000000	–0.519400000000
O	1.832836000000	–1.291056000000	–1.694356000000
C	2.912531000000	–1.751710000000	–2.492350000000
H	–4.377718000000	–3.128754000000	–5.491243000000
H	–2.672013000000	–3.640476000000	–5.489489000000
H	–3.090341000000	–1.912616000000	–5.588352000000
H	–5.365062000000	–1.925123000000	–3.444613000000
H	–4.070345000000	–0.710254000000	–3.510292000000
H	–4.349106000000	–1.630812000000	–2.010465000000
H	–3.186127000000	–4.976211000000	–3.333978000000
H	–4.851869000000	–4.372887000000	–3.423363000000
H	–3.920587000000	–4.142195000000	–1.934837000000
H	–1.861439000000	–0.630534000000	–4.261635000000
H	–1.716551000000	–4.136617000000	–1.780083000000
H	1.722761000000	–3.488976000000	–0.508889000000
H	0.440613000000	–4.707140000000	–0.662650000000
H	2.697303000000	–1.587174000000	–3.563652000000
H	3.095184000000	–2.827323000000	–2.344383000000
C	4.132784000000	–0.958333000000	–2.115651000000
O	4.003340000000	–0.032272000000	–1.323412000000
C	5.442080000000	–1.336579000000	–2.716637000000
H	6.154328000000	–0.503657000000	–2.659673000000
H	5.338399000000	–1.698038000000	–3.750277000000
H	5.829288000000	–2.173230000000	–2.109156000000
C	–3.381194000000	–3.570593000000	2.549452000000
C	–3.415834000000	–5.107293000000	2.680785000000
C	–4.268812000000	–3.125832000000	1.365099000000
C	–3.943017000000	–2.954546000000	3.839661000000
C	–1.944791000000	–3.111373000000	2.269114000000
C	–1.256850000000	–3.612667000000	1.155215000000
C	0.034371000000	–3.196856000000	0.810996000000
C	0.660134000000	–2.258145000000	1.654731000000
C	–0.004033000000	–1.686127000000	2.753673000000
C	–1.293616000000	–2.146092000000	3.047334000000
C	0.563406000000	–0.511012000000	3.538950000000
O	1.950458000000	–1.806350000000	1.316759000000
C	2.997108000000	–2.477609000000	2.016276000000
H	–4.443515000000	–5.445722000000	2.881379000000
H	–2.777825000000	–5.447705000000	3.510651000000
H	–3.076954000000	–5.610243000000	1.762762000000
H	–5.311893000000	–3.433070000000	1.535010000000
H	–3.939796000000	–3.572825000000	0.414789000000
H	–4.250915000000	–2.030357000000	1.248473000000
H	–3.342144000000	–3.228384000000	4.720397000000
H	–4.964903000000	–3.322359000000	4.010590000000
H	–3.998443000000	–1.856285000000	3.781914000000
H	–1.747085000000	–4.342672000000	0.511387000000
H	–1.806260000000	–1.712528000000	3.903553000000
H	1.652643000000	–0.479293000000	3.431690000000
H	0.348483000000	–0.667192000000	4.605790000000
H	2.618945000000	–3.437786000000	2.408091000000
H	3.328863000000	–1.883324000000	2.883182000000
C	4.184019000000	–2.790482000000	1.121754000000
O	4.093475000000	–2.720120000000	–0.085274000000
C	5.443021000000	–3.194827000000	1.834119000000
H	6.094966000000	–3.764631000000	1.159772000000
H	5.238589000000	–3.772811000000	2.748315000000
H	5.970853000000	–2.273949000000	2.136985000000
C	–3.377403000000	2.683883000000	3.625060000000
C	–3.377148000000	2.752883000000	5.166066000000
C	–4.327886000000	1.561287000000	3.151786000000
C	–3.894086000000	4.022428000000	3.076346000000
C	–1.964706000000	2.359454000000	3.120132000000
C	–1.313904000000	1.198227000000	3.571972000000
C	–0.052889000000	0.817365000000	3.108512000000
C	0.568392000000	1.658351000000	2.173426000000
C	–0.033018000000	2.814739000000	1.675013000000
C	–1.299910000000	3.149806000000	2.176256000000
C	0.582429000000	3.618876000000	0.534505000000
O	1.837390000000	1.294778000000	1.692745000000
C	2.918528000000	1.769457000000	2.479339000000
H	–4.387170000000	2.987787000000	5.534596000000
H	–2.693145000000	3.536640000000	5.525931000000
H	–3.073424000000	1.800011000000	5.624823000000
H	–5.355543000000	1.761254000000	3.491641000000
H	–4.031055000000	0.578475000000	3.549207000000
H	–4.339808000000	1.493872000000	2.052026000000
H	–3.249184000000	4.863385000000	3.373749000000
H	–4.900179000000	4.221712000000	3.472668000000
H	–3.972720000000	4.014269000000	1.978162000000
H	–1.810347000000	0.561895000000	4.305356000000
H	–1.776092000000	4.052977000000	1.799275000000
H	1.670019000000	3.491240000000	0.516105000000
H	0.369665000000	4.684942000000	0.696154000000
H	2.722458000000	1.593517000000	3.552315000000
H	3.079320000000	2.849574000000	2.336648000000
C	4.146004000000	1.001208000000	2.073133000000
O	4.019424000000	0.091484000000	1.261784000000
C	5.457289000000	1.386269000000	2.665537000000
H	6.179712000000	0.563937000000	2.586495000000
H	5.362004000000	1.730252000000	3.705855000000
H	5.827438000000	2.236584000000	2.066293000000

C	–3.414006000000	3.625682000000	–2.565662000000
C	–3.387173000000	5.152765000000	–2.782871000000
C	–4.318798000000	3.284961000000	–1.360472000000
C	–4.003726000000	2.960931000000	–3.818720000000
C	–1.994904000000	3.127484000000	–2.262256000000
C	–1.296287000000	3.639929000000	–1.157614000000
C	–0.024480000000	3.186729000000	–0.793591000000
C	0.575059000000	2.216331000000	–1.611455000000
C	–0.084911000000	1.649656000000	–2.707522000000
C	–1.366733000000	2.128253000000	–3.013967000000
C	0.491472000000	0.491403000000	–3.511726000000
O	1.886127000000	1.795524000000	–1.302025000000
C	2.891592000000	2.508642000000	–2.022959000000
H	–4.401073000000	5.523011000000	–2.997729000000
H	–2.740460000000	5.419105000000	–3.632872000000
H	–3.020142000000	5.690212000000	–1.895669000000
H	–5.349306000000	3.621873000000	–1.550018000000
H	–3.974461000000	3.773383000000	–0.436413000000
H	–4.343628000000	2.198046000000	–1.181103000000
H	–3.401573000000	3.169993000000	–4.716131000000
H	–5.015280000000	3.350371000000	–4.003290000000
H	–4.090350000000	1.869028000000	–3.702875000000
H	–1.767374000000	4.400966000000	–0.534695000000
H	–1.882270000000	1.693998000000	–3.868443000000
H	1.583277000000	0.481108000000	–3.423587000000
H	0.256923000000	0.653486000000	–4.573462000000
H	2.466888000000	3.457816000000	–2.392166000000
H	3.217271000000	1.935237000000	–2.906036000000
C	4.091412000000	2.853166000000	–1.159639000000
O	4.042961000000	2.748065000000	0.047688000000
C	5.307876000000	3.330411000000	–1.899607000000
H	5.956945000000	3.909214000000	–1.230158000000
H	5.049051000000	3.923348000000	–2.790077000000
H	5.864018000000	2.441740000000	–2.245271000000
O	0.022695000000	–0.131608000000	0.043177000000
H	–0.023355000000	–1.046012000000	0.369076000000
C	–1.365186000000	0.241758000000	–0.046357000000
H	–1.434847000000	1.314306000000	–0.218365000000
H	–1.865388000000	–0.000574000000	0.901971000000
H	–1.839718000000	–0.299369000000	–0.876663000000

Ca**L**$_c$MeOH'$^{2+}$, zero imaginary frequencies
143
–3570.44697861

Ca	2.572195000000	0.028623000000	–0.009603000000
C	–3.510065000000	–1.550891000000	–4.018208000000
C	–3.602853000000	–1.161777000000	–5.507719000000
C	–4.402162000000	–0.610999000000	–3.176075000000
C	–4.027382000000	–2.988581000000	–3.858173000000
C	–2.063144000000	–1.399737000000	–3.528842000000
C	–1.399678000000	–0.171879000000	–3.684308000000
C	–0.102716000000	0.049246000000	–3.214103000000
C	0.554411000000	–1.032791000000	–2.604610000000
C	–0.078178000000	–2.258177000000	–2.365011000000
C	–1.382546000000	–2.420878000000	–2.856839000000
C	0.547743000000	–3.341890000000	–1.485598000000
O	1.877243000000	–0.856142000000	–2.173690000000
C	2.859416000000	–1.097569000000	–3.167485000000
H	–4.638359000000	–1.271845000000	–5.863706000000
H	–2.960428000000	–1.807846000000	–6.125480000000
H	–3.304772000000	–0.117576000000	–5.684319000000
H	–5.452735000000	–0.692049000000	–3.494745000000
H	–4.100567000000	0.442234000000	–3.282827000000
H	–4.348978000000	–0.871281000000	–2.106741000000
H	–3.413600000000	–3.709275000000	–4.419974000000
H	–5.055131000000	–3.058034000000	–4.242779000000
H	–4.054919000000	–3.302022000000	–2.803183000000
H	–1.918485000000	0.651831000000	–4.174443000000
H	–1.874956000000	–3.378147000000	–2.699716000000
H	1.635456000000	–3.215232000000	–1.452251000000
H	0.340636000000	–4.326930000000	–1.928083000000
H	2.461568000000	–0.860469000000	–4.170216000000
H	3.162593000000	–2.160332000000	–3.182606000000
C	4.073429000000	–0.231407000000	–2.914113000000
O	4.072813000000	0.554260000000	–1.979295000000
C	5.245833000000	–0.407236000000	–3.826473000000
H	5.873198000000	0.493446000000	–3.821594000000
H	4.947102000000	–0.670066000000	–4.851736000000
H	5.847371000000	–1.244740000000	–3.430549000000
C	–3.467122000000	–4.056316000000	1.513798000000
C	–3.578945000000	–5.542007000000	1.115378000000
C	–4.344964000000	–3.195384000000	0.577221000000
C	–3.982322000000	–3.897368000000	2.952522000000
C	–2.015082000000	–3.583647000000	1.365367000000
C	–1.344666000000	–3.759592000000	0.145839000000
C	–0.040720000000	–3.303034000000	–0.073397000000
C	0.616133000000	–2.683264000000	1.010831000000
C	–0.037119000000	–2.405668000000	2.223545000000
C	–1.340853000000	–2.894131000000	2.380201000000
C	0.559240000000	–1.484704000000	3.284693000000
O	1.927017000000	–2.223446000000	0.823623000000
C	2.944495000000	–3.182333000000	1.062227000000
H	–4.618870000000	–5.884187000000	1.225929000000
H	–2.943931000000	–6.172621000000	1.756300000000
H	–3.287000000000	–5.716750000000	0.069136000000
H	–5.400271000000	–3.497715000000	0.658041000000
H	–4.044923000000	–3.303553000000	–0.476013000000
H	–4.273923000000	–2.128159000000	0.840999000000
H	–3.370572000000	–4.463908000000	3.671242000000
H	–5.011644000000	–4.277652000000	3.020977000000
H	–4.006107000000	–2.843299000000	3.268909000000
H	–1.861314000000	–4.253056000000	–0.676517000000
H	–1.846320000000	–2.707029000000	3.324764000000
H	1.645411000000	–1.425792000000	3.156621000000
H	0.364767000000	–1.917447000000	4.276986000000
H	2.592734000000	–4.195473000000	0.797814000000
H	3.229904000000	–3.203527000000	2.129511000000
C	4.159060000000	–2.856191000000	0.221104000000
O	4.125986000000	–1.908921000000	–0.547931000000
C	5.370404000000	–3.714541000000	0.403305000000
H	6.007966000000	–3.671754000000	–0.489221000000
H	5.118044000000	–4.755066000000	0.654414000000
H	5.941916000000	–3.296440000000	1.251046000000
C	–3.487936000000	1.480270000000	4.025585000000
C	–3.589043000000	1.062037000000	5.506439000000
C	–4.373007000000	0.555073000000	3.160193000000
C	–4.007655000000	2.919815000000	3.891220000000
C	–2.039169000000	1.341656000000	3.537612000000
C	–1.362060000000	0.121717000000	3.698576000000
C	–0.064724000000	–0.088307000000	3.223998000000
C	0.576638000000	0.996907000000	2.601964000000
C	–0.067880000000	2.215337000000	2.363324000000
C	–1.371127000000	2.366297000000	2.859875000000
C	0.544770000000	3.311234000000	1.491948000000
O	1.900069000000	0.830609000000	2.170477000000
C	2.885813000000	1.089567000000	3.154990000000
H	–4.625968000000	1.168306000000	5.859334000000
H	–2.947972000000	1.693867000000	6.140095000000
H	–3.295910000000	0.013497000000	5.664970000000
H	–5.425642000000	0.625318000000	3.474770000000
H	–4.066907000000	–0.498645000000	3.248052000000
H	–4.314926000000	0.837168000000	2.096741000000
H	–3.393771000000	3.630788000000	4.465100000000
H	–5.035290000000	2.980907000000	4.277621000000
H	–4.036354000000	3.252056000000	2.842271000000
H	–1.869245000000	–0.701474000000	4.201269000000
H	–1.874554000000	3.316668000000	2.695561000000
H	1.632714000000	3.188683000000	1.449796000000
H	0.337822000000	4.288501000000	1.951714000000
H	2.504935000000	0.840111000000	4.161480000000
H	3.166662000000	2.158542000000	3.171252000000
C	4.113248000000	0.249577000000	2.879867000000
O	4.112481000000	–0.530963000000	1.940487000000
C	5.296417000000	0.444476000000	3.774062000000
H	5.937827000000	–0.446238000000	3.760988000000
H	5.009640000000	0.704922000000	4.803291000000
H	5.878739000000	1.290126000000	3.366810000000

C	–3.460575000000	4.134026000000	–1.494835000000
C	–3.506813000000	5.640038000000	–1.166085000000
C	–4.359355000000	3.355259000000	–0.508119000000
C	–4.005690000000	3.927968000000	–2.916373000000
C	–2.025130000000	3.611948000000	–1.345257000000
C	–1.338479000000	3.794291000000	–0.133699000000
C	–0.054827000000	3.285890000000	0.087086000000
C	0.570818000000	2.626253000000	–0.985153000000
C	–0.088266000000	2.359823000000	–2.190648000000
C	–1.381481000000	2.879453000000	–2.348777000000
C	0.503665000000	1.450772000000	–3.264441000000
O	1.892673000000	2.190316000000	–0.818903000000
C	2.876702000000	3.177757000000	–1.077389000000
H	–4.534348000000	6.019419000000	–1.272787000000
H	–2.860246000000	6.213062000000	–1.848238000000
H	–3.184676000000	5.850864000000	–0.135432000000
H	–5.402738000000	3.696923000000	–0.588138000000
H	–4.039855000000	3.498448000000	0.535156000000
H	–4.336171000000	2.274950000000	–0.723783000000
H	–3.389085000000	4.441705000000	–3.669751000000
H	–5.023655000000	4.337794000000	–2.985924000000
H	–4.066683000000	2.861677000000	–3.183925000000
H	–1.830514000000	4.325021000000	0.681473000000
H	–1.895785000000	2.694656000000	–3.289418000000
H	1.591490000000	1.400628000000	–3.146196000000
H	0.297786000000	1.889996000000	–4.251604000000
H	2.497138000000	4.181132000000	–0.814075000000
H	3.146122000000	3.203702000000	–2.149036000000
C	4.113062000000	2.896250000000	–0.253727000000
O	4.124846000000	1.945834000000	0.513817000000
C	5.290592000000	3.797801000000	–0.447762000000
H	5.942723000000	3.771631000000	0.434818000000
H	4.996847000000	4.830240000000	–0.686852000000
H	5.863948000000	3.407709000000	–1.307521000000
O	0.045777000000	–0.119590000000	0.006008000000
H	0.042001000000	–1.084023000000	0.100214000000
C	–1.352143000000	0.204262000000	0.005725000000
H	–1.462678000000	1.287536000000	0.030886000000
H	–1.834031000000	–0.221031000000	0.898220000000
H	–1.831461000000	–0.189706000000	–0.901551000000

Sr**L**$_c$MeOH'$^{2+}$, zero imaginary frequencies
143
–2923.63712044

Sr	2.595818000000	0.026732000000	–0.009564000000
C	–3.624236000000	–1.510988000000	–3.973532000000
C	–3.745014000000	–1.087283000000	–5.451489000000
C	–4.500270000000	–0.590806000000	–3.093599000000
C	–4.138809000000	–2.952338000000	–3.838654000000
C	–2.168372000000	–1.371243000000	–3.507337000000
C	–1.501880000000	–0.145181000000	–3.662101000000
C	–0.192921000000	0.063850000000	–3.219741000000
C	0.473856000000	–1.034499000000	–2.647721000000
C	–0.168646000000	–2.251486000000	–2.384365000000
C	–1.484473000000	–2.399690000000	–2.850044000000
C	0.458707000000	–3.337757000000	–1.504751000000
O	1.815896000000	–0.881110000000	–2.272680000000
C	2.758468000000	–1.128307000000	–3.301898000000
H	–4.786698000000	–1.191304000000	–5.790819000000
H	–3.112952000000	–1.717257000000	–6.095991000000
H	–3.452505000000	–0.038542000000	–5.609353000000
H	–5.556330000000	–0.662081000000	–3.396159000000
H	–4.197718000000	0.463958000000	–3.179878000000
H	–4.429663000000	–0.876645000000	–2.031953000000
H	–3.531634000000	–3.659618000000	–4.424172000000
H	–5.171553000000	–3.014097000000	–4.211025000000
H	–4.152803000000	–3.289656000000	–2.790925000000
H	–2.027908000000	0.686021000000	–4.130564000000
H	–1.983864000000	–3.351815000000	–2.685606000000
H	1.546266000000	–3.208715000000	–1.468860000000
H	0.255559000000	–4.322382000000	–1.950408000000
H	2.333976000000	–0.867396000000	–4.288073000000
H	3.034647000000	–2.198479000000	–3.344398000000
C	4.007246000000	–0.297335000000	–3.087167000000
O	4.070576000000	0.487543000000	–2.152483000000
C	5.137161000000	–0.509984000000	–4.045861000000
H	5.762132000000	0.390643000000	–4.106372000000
H	4.792287000000	–0.812778000000	–5.045166000000
H	5.762009000000	–1.328680000000	–3.646453000000
C	–3.577387000000	–4.011461000000	1.479650000000
C	–3.712358000000	–5.488812000000	1.058230000000
C	–4.441786000000	–3.123018000000	0.556500000000
C	–4.089901000000	–3.867947000000	2.921032000000
C	–2.118067000000	–3.559000000000	1.338467000000
C	–1.446792000000	–3.735870000000	0.119721000000
C	–0.131957000000	–3.305925000000	–0.090234000000
C	0.535003000000	–2.718274000000	1.007513000000
C	–0.124164000000	–2.419028000000	2.213813000000
C	–1.438481000000	–2.883084000000	2.358654000000
C	0.476309000000	–1.499775000000	3.278455000000
O	1.866208000000	–2.315307000000	0.841686000000
C	2.842506000000	–3.313400000000	1.090439000000
H	–4.757312000000	–5.816686000000	1.164551000000
H	–3.086561000000	–6.139091000000	1.688488000000
H	–3.424258000000	–5.651837000000	0.009038000000
H	–5.501795000000	–3.409744000000	0.632790000000
H	–4.142947000000	–3.220812000000	–0.497943000000
H	–4.353866000000	–2.060975000000	0.835183000000
H	–3.484172000000	–4.452243000000	3.630572000000
H	–5.123637000000	–4.236956000000	2.984616000000
H	–4.101420000000	–2.818768000000	3.253551000000
H	–1.970957000000	–4.210872000000	–0.708266000000
H	–1.948239000000	–2.689249000000	3.299155000000
H	1.561453000000	–1.435050000000	3.142733000000
H	0.292321000000	–1.940709000000	4.269403000000
H	2.461040000000	–4.310355000000	0.805209000000
H	3.099991000000	–3.359847000000	2.164781000000
C	4.094924000000	–3.031739000000	0.284775000000
O	4.129165000000	–2.083533000000	–0.484693000000
C	5.261888000000	–3.942853000000	0.504736000000
H	5.902446000000	–3.966086000000	–0.386649000000
H	4.957135000000	–4.959498000000	0.792483000000
H	5.856098000000	–3.524384000000	1.336677000000
C	–3.603300000000	1.435912000000	3.982355000000
C	–3.735800000000	0.973052000000	5.447202000000
C	–4.471441000000	0.538831000000	3.071483000000
C	–4.118512000000	2.880086000000	3.882400000000
C	–2.145280000000	1.309992000000	3.518523000000
C	–1.462490000000	0.093546000000	3.680701000000
C	–0.153070000000	–0.102151000000	3.232923000000
C	0.495733000000	1.000683000000	2.647516000000
C	–0.162099000000	2.208090000000	2.381989000000
C	–1.476921000000	2.341862000000	2.852418000000
C	0.448833000000	3.307509000000	1.509498000000
O	1.838621000000	0.860309000000	2.271436000000
C	2.784524000000	1.120564000000	3.292917000000
H	–4.779125000000	1.073744000000	5.782311000000
H	–3.104861000000	1.581820000000	6.112809000000
H	–3.451357000000	–0.081448000000	5.579751000000
H	–5.530079000000	0.598859000000	3.367661000000
H	–4.165487000000	–0.516695000000	3.132623000000
H	–4.393007000000	0.853548000000	2.018688000000
H	–3.509642000000	3.572861000000	4.483234000000
H	–5.150764000000	2.932723000000	4.257610000000
H	–4.134378000000	3.242361000000	2.843292000000
H	–1.975136000000	–0.737633000000	4.163464000000
H	–1.989876000000	3.285042000000	2.678251000000
H	1.536388000000	3.182375000000	1.461726000000
H	0.246751000000	4.283974000000	1.973648000000
H	2.376749000000	0.844840000000	4.282302000000
H	3.039592000000	2.195893000000	3.339712000000
C	4.046027000000	0.315606000000	3.055154000000
O	4.109597000000	–0.460667000000	2.112873000000
C	5.186010000000	0.542286000000	3.998285000000
H	5.826312000000	–0.348297000000	4.046422000000
H	4.851023000000	0.836413000000	5.003492000000
H	5.791905000000	1.372071000000	3.592507000000

C	–3.586262000000	4.076898000000	–1.457794000000
C	–3.672397000000	5.570532000000	–1.084402000000
C	–4.468095000000	3.246475000000	–0.498112000000
C	–4.121400000000	3.901053000000	–2.887302000000
C	–2.139156000000	3.584755000000	–1.317958000000
C	–1.452421000000	3.768887000000	–0.106860000000
C	–0.155617000000	3.289393000000	0.103633000000
C	0.483365000000	2.666746000000	–0.984435000000
C	–0.182830000000	2.374878000000	–2.181968000000
C	–1.488245000000	2.867339000000	–2.327696000000
C	0.416221000000	1.467619000000	–3.257306000000
O	1.826300000000	2.291922000000	–0.842170000000
C	2.766070000000	3.318910000000	–1.106884000000
H	–4.708597000000	5.926821000000	–1.186455000000
H	–3.036726000000	6.180031000000	–1.744835000000
H	–3.362345000000	5.758895000000	–0.045768000000
H	–5.519616000000	3.564172000000	–0.570747000000
H	–4.153851000000	3.366353000000	0.549642000000
H	–4.417356000000	2.174006000000	–0.745637000000
H	–3.509420000000	4.445272000000	–3.622840000000
H	–5.145840000000	4.295466000000	–2.950769000000
H	–4.163990000000	2.841992000000	–3.184888000000
H	–1.954463000000	4.278626000000	0.715188000000
H	–2.007232000000	2.674356000000	–3.263741000000
H	1.502943000000	1.413525000000	–3.129192000000
H	0.221222000000	1.912468000000	–4.244439000000
H	2.357453000000	4.304077000000	–0.817897000000
H	3.005760000000	3.374268000000	–2.185304000000
C	4.039246000000	3.081382000000	–0.321200000000
O	4.120459000000	2.131219000000	0.443851000000
C	5.169295000000	4.035328000000	–0.552410000000
H	5.821903000000	4.076432000000	0.329538000000
H	4.823067000000	5.041811000000	–0.828570000000
H	5.765896000000	3.644657000000	–1.396047000000
O	–0.090163000000	–0.126321000000	0.008837000000
H	–0.083743000000	–1.090916000000	0.102093000000
C	–1.486921000000	0.194904000000	0.007314000000
H	–1.595443000000	1.278932000000	0.017552000000
H	–1.969162000000	–0.216737000000	0.906120000000
H	–1.970119000000	–0.209616000000	–0.893377000000

Ba**L**$_c$MeOH'$^{2+}$, zero imaginary frequencies
143
–2918.37883139

Ba	2.713778000000	0.034057000000	–0.002377000000
C	–3.737199000000	–1.460836000000	–3.949039000000
C	–3.873139000000	–1.006373000000	–5.416489000000
C	–4.606268000000	–0.560661000000	–3.042123000000
C	–4.247910000000	–2.905888000000	–3.840752000000
C	–2.276962000000	–1.328776000000	–3.494247000000
C	–1.609439000000	–0.102494000000	–3.639956000000
C	–0.291448000000	0.095467000000	–3.219388000000
C	0.383847000000	–1.017562000000	–2.683889000000
C	–0.265786000000	–2.228927000000	–2.406741000000
C	–1.590162000000	–2.364470000000	–2.852253000000
C	0.363968000000	–3.321811000000	–1.534320000000
O	1.748331000000	–0.893755000000	–2.388779000000
C	2.606773000000	–1.159568000000	–3.485471000000
H	–4.917760000000	–1.105952000000	–5.748032000000
H	–3.245716000000	–1.620857000000	–6.080217000000
H	–3.584990000000	0.046290000000	–5.555055000000
H	–5.665170000000	–0.627288000000	–3.335792000000
H	–4.305572000000	0.495814000000	–3.110317000000
H	–4.524767000000	–0.867007000000	–1.987171000000
H	–3.642409000000	–3.599364000000	–4.444262000000
H	–5.282807000000	–2.962421000000	–4.207976000000
H	–4.255124000000	–3.265233000000	–2.800424000000
H	–2.140823000000	0.735946000000	–4.088126000000
H	–2.093460000000	–3.314271000000	–2.688411000000
H	1.451092000000	–3.187980000000	–1.491270000000
H	0.168298000000	–4.304070000000	–1.989244000000
H	2.134354000000	–0.845318000000	–4.434069000000
H	2.814376000000	–2.242513000000	–3.578219000000
C	3.916169000000	–0.409157000000	–3.341750000000
O	4.093625000000	0.356705000000	–2.406650000000
C	4.963520000000	–0.678156000000	–4.379112000000
H	5.625708000000	0.191350000000	–4.484381000000
H	4.533295000000	–0.960027000000	–5.351066000000
H	5.575874000000	–1.527339000000	–4.026814000000
C	–3.697457000000	–3.975243000000	1.424067000000
C	–3.847677000000	–5.443512000000	0.976837000000
C	–4.551087000000	–3.062420000000	0.515144000000
C	–4.210103000000	–3.852369000000	2.867367000000
C	–2.232923000000	–3.536823000000	1.292946000000
C	–1.558870000000	–3.706132000000	0.075057000000
C	–0.233511000000	–3.302582000000	–0.120420000000
C	0.441619000000	–2.753544000000	0.993785000000
C	–0.225481000000	–2.439094000000	2.193380000000
C	–1.550141000000	–2.878818000000	2.321980000000
C	0.377131000000	–1.530237000000	3.268519000000
O	1.798672000000	–2.435790000000	0.862365000000
C	2.687680000000	–3.512002000000	1.115537000000
H	–4.896219000000	–5.762038000000	1.076098000000
H	–3.229717000000	–6.111389000000	1.596364000000
H	–3.559923000000	–5.591306000000	–0.074713000000
H	–5.614390000000	–3.338327000000	0.585602000000
H	–4.251785000000	–3.147122000000	–0.540111000000
H	–4.451397000000	–2.005990000000	0.810054000000
H	–3.609780000000	–4.454009000000	3.566949000000
H	–5.247054000000	–4.213473000000	2.923841000000
H	–4.213152000000	–2.809323000000	3.218475000000
H	–2.088108000000	–4.158645000000	–0.761695000000
H	–2.064577000000	–2.684521000000	3.259361000000
H	1.462016000000	–1.459504000000	3.130295000000
H	0.198726000000	–1.983364000000	4.255315000000
H	2.251806000000	–4.467241000000	0.770243000000
H	2.880941000000	–3.621334000000	2.199243000000
C	4.001770000000	–3.301203000000	0.388282000000
O	4.152087000000	–2.342922000000	–0.353628000000
C	5.084400000000	–4.302773000000	0.651699000000
H	5.765697000000	–4.360634000000	–0.207405000000
H	4.688331000000	–5.297491000000	0.902727000000
H	5.666768000000	–3.947316000000	1.520510000000
C	–3.730016000000	1.379367000000	3.942817000000
C	–3.888462000000	0.875923000000	5.391522000000
C	–4.584464000000	0.509836000000	2.993385000000
C	–4.240428000000	2.827344000000	3.876120000000
C	–2.264714000000	1.263538000000	3.499465000000
C	–1.578571000000	0.048951000000	3.658041000000
C	–0.257580000000	–0.132404000000	3.238548000000
C	0.400493000000	0.987053000000	2.693535000000
C	–0.266583000000	2.186095000000	2.407620000000
C	–1.592963000000	2.303067000000	2.849694000000
C	0.345778000000	3.293778000000	1.543435000000
O	1.767755000000	0.879066000000	2.404753000000
C	2.622325000000	1.139884000000	3.504565000000
H	–4.936701000000	0.972165000000	5.712241000000
H	–3.265893000000	1.462361000000	6.084522000000
H	–3.611530000000	–0.183425000000	5.498926000000
H	–5.647907000000	0.563305000000	3.273339000000
H	–4.280112000000	–0.547056000000	3.031418000000
H	–4.487783000000	0.852135000000	1.950974000000
H	–3.635777000000	3.501750000000	4.501628000000
H	–5.276628000000	2.872993000000	4.241283000000
H	–4.244551000000	3.217936000000	2.847347000000
H	–2.097102000000	–0.790532000000	4.118580000000
H	–2.111950000000	3.241536000000	2.670392000000
H	1.433254000000	3.166950000000	1.489567000000
H	0.148406000000	4.266917000000	2.017253000000
H	2.160390000000	0.796803000000	4.448492000000
H	2.807840000000	2.224824000000	3.619076000000
C	3.945313000000	0.418690000000	3.337256000000
O	4.130429000000	–0.322561000000	2.383437000000
C	4.994629000000	0.684706000000	4.373142000000
H	5.676336000000	–0.172349000000	4.452654000000
H	4.565669000000	0.933974000000	5.354534000000
H	5.585782000000	1.555468000000	4.037566000000

C	–3.714517000000	4.031268000000	–1.403791000000
C	–3.828183000000	5.513132000000	–0.993056000000
C	–4.584335000000	3.161984000000	–0.468085000000
C	–4.242427000000	3.883498000000	–2.839270000000
C	–2.259459000000	3.560598000000	–1.272201000000
C	–1.572776000000	3.739711000000	–0.060625000000
C	–0.263747000000	3.288354000000	0.137452000000
C	0.387873000000	2.706652000000	–0.967235000000
C	–0.284205000000	2.396951000000	–2.158339000000
C	–1.601473000000	2.861690000000	–2.289780000000
C	0.320742000000	1.500214000000	–3.241797000000
O	1.755070000000	2.418814000000	–0.857100000000
C	2.604106000000	3.517563000000	–1.142533000000
H	–4.870033000000	5.853854000000	–1.090575000000
H	–3.200663000000	6.150271000000	–1.634960000000
H	–3.525930000000	5.680630000000	0.051428000000
H	–5.641249000000	3.462589000000	–0.535745000000
H	–4.274310000000	3.261973000000	0.582856000000
H	–4.513662000000	2.096956000000	–0.741064000000
H	–3.634212000000	4.452678000000	–3.558881000000
H	–5.271520000000	4.266520000000	–2.897224000000
H	–4.271209000000	2.831766000000	–3.162922000000
H	–2.083239000000	4.226541000000	0.769632000000
H	–2.123777000000	2.665282000000	–3.222839000000
H	1.407261000000	1.441727000000	–3.110300000000
H	0.131602000000	1.955219000000	–4.225773000000
H	2.149706000000	4.462413000000	–0.792070000000
H	2.763535000000	3.626898000000	–2.232100000000
C	3.943855000000	3.354089000000	–0.453050000000
O	4.149174000000	2.402246000000	0.285361000000
C	4.984442000000	4.391341000000	–0.746440000000
H	5.686313000000	4.473613000000	0.093917000000
H	4.547806000000	5.371468000000	–0.987126000000
H	5.554931000000	4.054737000000	–1.630461000000
O	–0.172639000000	–0.117902000000	0.015215000000
H	–0.146339000000	–1.081727000000	0.110111000000
C	–1.573801000000	0.176901000000	0.007613000000
H	–1.700729000000	1.258949000000	0.026299000000
H	–2.056272000000	–0.249138000000	0.899582000000
H	–2.046951000000	–0.226992000000	–0.898549000000

References

(1) Arnaud-Neu, F.; Collins, E. M.; Deasy, M.; Ferguson, G.; Harris, S. J.; Kaitner, B.; Lough, A. J.; McKervey, M. A.; Marques, E. Synthesis, x-Ray Crystal Structures, and Cation-Binding Properties of Alkyl Calixaryl Esters and Ketones, a New Family of Macrocyclic Molecular Receptors. *J. Am. Chem. Soc.* **1989**, *111* (23), 8681–8691. https://doi.org/10.1021/ja00205a018.

(2) Požar, J.; Nikšić-Franjić, I.; Cvetnić, M.; Leko, K.; Cindro, N.; Pičuljan, K.; Borilović, I.; Frkanec, L.; Tomišić, V. Solvation Effect on Complexation of Alkali Metal Cations by a Calix[4]arene Ketone Derivative. *J. Phys. Chem. B* **2017**, *121* (36), 8539–8550. https://doi.org/10.1021/acs.jpcb.7b05093.
